# SARS-CoV-2 spike variants differ in their allosteric responses to linoleic acid

**DOI:** 10.1093/jmcb/mjad021

**Published:** 2023-03-29

**Authors:** A Sofia F Oliveira, Deborah K Shoemark, Andrew D Davidson, Imre Berger, Christiane Schaffitzel, Adrian J Mulholland

**Affiliations:** School of Chemistry, Centre for Computational Chemistry, University of Bristol, Bristol BS8 1TS, UK; School of Chemistry, University of Bristol, Bristol BS8 1TS, UK; School of Biochemistry, University of Bristol, Bristol BS8 1TD, UK; School of Biochemistry, University of Bristol, Bristol BS8 1TD, UK; School of Cellular and Molecular Medicine, University of Bristol, University Walk, Bristol BS8 1TD, UK; School of Chemistry, University of Bristol, Bristol BS8 1TS, UK; School of Biochemistry, University of Bristol, Bristol BS8 1TD, UK; School of Chemistry, Max Planck Bristol Centre for Minimal Biology, Bristol BS8 1TS, UK; School of Biochemistry, University of Bristol, Bristol BS8 1TD, UK; School of Chemistry, Centre for Computational Chemistry, University of Bristol, Bristol BS8 1TS, UK

**Keywords:** SARS-CoV-2 spike, allosteric modulation, D-NEMD simulations, fatty acid binding site, SARS-CoV-2 variants

## Abstract

The SARS-CoV-2 spike protein contains a functionally important fatty acid (FA) binding site, which is also found in some other coronaviruses, e.g. SARS-CoV and MERS-CoV. The occupancy of the FA site by linoleic acid (LA) reduces infectivity by ‘locking’ the spike in a less infectious conformation. Here, we use dynamical-nonequilibrium molecular dynamics (D-NEMD) simulations to compare the allosteric responses of spike variants to LA removal. D-NEMD simulations show that the FA site is coupled to other functional regions of the protein, e.g. the receptor-binding motif (RBM), N-terminal domain (NTD), furin cleavage site, and regions surrounding the fusion peptide. D-NEMD simulations also identify the allosteric networks connecting the FA site to these functional regions. The comparison between the wild-type spike and four variants (Alpha, Delta, Delta plus, and Omicron BA.1) shows that the variants differ significantly in their responses to LA removal. The allosteric connections to the FA site on Alpha are generally similar to those on the wild-type protein, with the exception of the RBM and the S71–R78 region, which show a weaker link to the FA site. In contrast, Omicron is the most different variant, exhibiting significant differences in the RBM, NTD, V622–L629, and furin cleavage site. These differences in the allosteric modulation may be of functional relevance, potentially affecting transmissibility and virulence. Experimental comparison of the effects of LA on SARS-CoV-2 variants, including emerging variants, is warranted.

## Introduction

**Figure 1:**
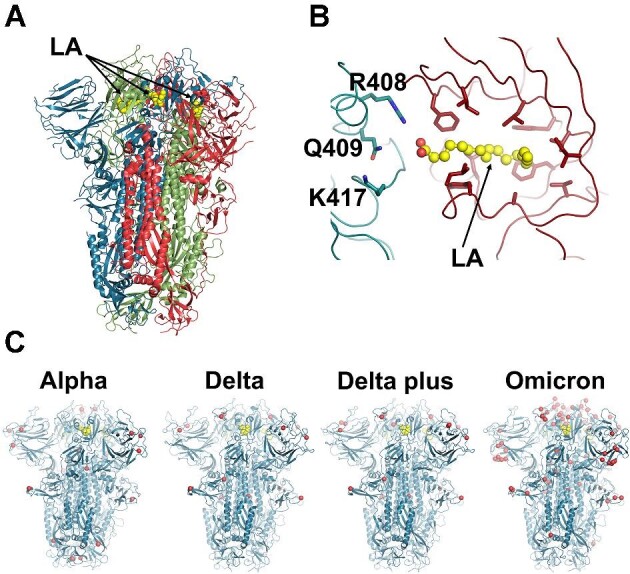
The ectodomain of SARS-CoV-2 spike trimer with LA bound to the FA binding site. (**A**) Three-dimensional structure of the ectodomain of SARS-CoV-2 spike trimer with LA bound in the locked conformation (PDB code 6ZB5; Toelzer et al., 2020). The spike protein is a homotrimer (Walls et al., 2020), with each monomer shown in a different colour: green, red, and blue. LA molecules are highlighted with yellow spheres. Note that each FA binding site is located at the interface between two neighbouring monomers and is formed by residues from two adjacent RBDs. (**B**) Detailed view of the FA binding site. The pocket is lined by the hydrophobic and aromatic residues, and the LA acidic headgroup is located near R408, Q409, and K417. (**C**) Models used as the starting points for the equilibrium MD simulations of the Alpha, Delta, Delta plus, and Omicron variants (Shoemark et al., 2022). Yellow spheres represent LA molecules. Red spheres highlight the positions of mutations, deletions, and insertions in the four simulated variants.

The spike glycoprotein, which is located on the surface of the virion, mediates SARS-CoV-2 entry into host cells by binding primarily to the receptor angiotensin-converting enzyme 2 (ACE2) (Wang et al., 2020b; Yan et al., 2020) as well as to other receptors, such as neuropilin-1 (Cantuti-Castelvetri et al., 2020; Daly et al., 2020) and potentially nicotinic acetylcholine receptors (Oliveira et al., 2021b; Chrestia et al., 2022). The spike ectodomain (Figure 1A; Supplementary Figure S14) contains the receptor-binding domain (RBD), which directly binds to the human receptors, as well as all the machinery needed to fuse the host and viral membranes, including the fusion peptide (FP) (Cai et al., 2020; Walls et al., 2020; Wrapp et al., 2020). The SARS-CoV-2 spike contains two proteolytic cleavage sites (Walls et al., 2020): a furin cleavage site located at the S1/S2 junction (residues N679–R685), which distinguishes SARS-CoV-2 from other β-coronavirus spike proteins, affects the overall stability of the protein, and modulates infectivity (Davidson et al., 2020; Wrobel et al., 2020; Takeda, 2022), and a cleavage site in the S2 subunit that releases the FP (Takeda, 2022; Supplementary Figure S14). A free fatty acid (FA) binding site was also discovered in the SARS-CoV-2 spike in late 2020 (Toelzer et al., 2020). Subsequently, equivalent FA sites have been identified in other closely related spikes, including those in pagolin coronavirus and wild-type SARS-CoV (Bangaru et al., 2020; Toelzer et al., 2020; Zhang et al., 2021b). The FA pocket is located at the interface between two neighbouring RBDs on adjacent monomers in the homotrimeric spike (Figure 1A; Toelzer et al., 2020). This hydrophobic pocket is formed by two RBDs, one providing the aromatic and hydrophobic residues to accommodate the FA hydrocarbon tail and the other one from the adjacent chain providing the polar (Q409) and positively charged (R408 and K417) residues that bind the FA carboxylate headgroup (Figure 1B; Toelzer et al., 2020). The presence of linoleic acid (LA) in the FA pocket stabilizes the locked conformation of the spike, in which the receptor-binding motifs (RBMs) are buried inside the RBD trimer, preventing them from binding to ACE2, and thus reduces infectivity (Toelzer et al., 2020).

Biomolecular simulations have provided molecular-level insights into SARS-CoV-2 spike structure and dynamics, uncovering the effects of mutations, predicting interactions, and revealing allosteric connections in the protein ([Bibr bib2][Bibr bib2]; Barros et al., 2020; Casalino et al., 2020, 2021; Daly et al., 2020; Toelzer et al., 2020; Yu et al., 2020; Ray et al., 2021; Shoemark et al., 2021; Sztain et al., 2021; Triveri et al., 2021; Verkhivker and Di Paola, 2021; Zimmerman et al., 2021; Gupta et al., 2022; Oliveira et al., 2022; Dommer et al., 2023). Equilibrium molecular dynamics (MD) simulations indicated persistent and stable interactions between LA and the spike trimer (Toelzer et al., 2020; Shoemark et al., 2021). Specifically, binding of LA rigidifies the FA binding site (Shoemark et al., 2021). Dynamical-nonequilibrium molecular dynamics (D-NEMD) simulations (Oliveira et al., 2021a) are emerging as a practical technique for identifying structural communication pathways in biomolecular systems (Oliveira et al., 2019a, b; Abreu et al., 2020; Galdadas et al., 2021). Recently, using D-NEMD simulations, we showed that the FA site is allosterically coupled to functional motifs involved in membrane fusion and to epitopes (Gupta et al., 2022; Oliveira et al., 2022). These simulations revealed that the removal of LA from the FA site induces a long-range structural response in the RBM, N-terminal domain (NTD), furin cleavage site, and FP-surrounding regions (Gupta et al., 2022; Oliveira et al., 2022). D-NEMD simulations have also highlighted different allosteric and dynamical behaviours among the wild-type spike (also known as ‘early 2020’), the D614G variant, and the BriSΔ variant (a variant containing an 8-amino acid deletion encompassing the furin recognition motif and S1/S2 cleavage site) (Gupta et al., 2022; Oliveira et al., 2022).

Here, we use D-NEMD simulations (Ciccotti et al., 1979; Ciccotti, 1991; Ciccotti and Ferrario, 2016; Oliveira et al., 2021a) to analyse the responses of four spike variants to LA removal. We compare the structural and dynamical responses of the Alpha (B.1.1.7), Delta (B.1.617.2), Delta plus (B.1.617.2-AY.1), and Omicron BA.1 (B.1.1.529) variants with the wild-type spike. Alpha was first detected in the UK in late 2020 and was largely responsible for the surge of cases in the winter of 2020/2021 (Davies et al., 2021; Tang et al., 2021; Zhang et al., 2021a). This variant contains seven spike mutations and three deletions, notably L18F, H69Δ, V70Δ, Y144Δ, N501Y, A570D, P681H, T716I, S982A, and D1118H (Figure 1C), and is more transmissible than the wild-type virus (Davies et al., 2021; Tang et al., 2021; Zhang et al., 2021a). Delta was initially identified in India in late 2020 and was, until recently, the dominant strain globally (https://cov-lineages.org/global_report_B.1.617.2.html). It harbours six mutations and three deletions in the spike (T19R, E156Δ, F157Δ, R158Δ, L452R, T478K, D614G, P681R, and D950N) (Figure 1C) and shows enhanced transmissibility compared to previous variants (https://stacks.cdc.gov/view/cdc/108671). By the middle of 2021, a variant of Delta with the K417N mutation (nicknamed as ‘Delta plus’) was identified in Nepal and quickly spread to the rest of the world (Figure 1C; https://cov-lineages.org/lineage.html?lineage=AY.100). Note that although Delta plus spread worldwide, it did not displace Delta in the same way as the Omicron did (for more details, see https://ourworldindata.org/grapher/covid-variants-bar?time=2022-01-31). The Omicron BA.1 variant (hereafter referred to as Omicron) was initially detected in South Africa in November 2021 (Karim and Karim, 2021; Vaughan, 2021). It spread rapidly globally and became the dominant variant in many countries (see https://ourworldindata.org/grapher/covid-variants-bar?time=2022-01-31). This variant includes up to 40 mutations, deletions, and insertions on the spike, e.g. A67V, H69Δ–V70Δ, T95I, G142D, V143Δ, Y144Δ, Y145Δ, N211I, L212Δ, D215E, PED insertion in position 215, G339D, S371L, S373P, S375F, K417N, N440K, G446S, S477N, T478K, E484A, Q493R, G496S, Q498R, N501Y, Y505H, T547K, D614G, H655Y, N679K, P681H, N764K, D796Y, N856K, Q954H, N969K, and L981F (Figure 1C; Majumdar and Sarkar, 2021). Omicron is more transmissible than Delta and any other preceding variants, but it apparently causes less severe disease than previous strains (Zhang et al., 2021c; Nealon and Cowling, 2022; Yang and Shaman, 2022).

## Results

### Structural responses of the wild-type, Alpha, Delta, Delta plus, and Omicron spikes

Hundreds of D-NEMD simulations were performed to analyse the responses of the wild-type spike and the Alpha, Delta, Delta plus, and Omicron variants to LA removal. The models used here were taken from Shoemark et al. (2022) and were built using the cryo-EM structures of the locked ectodomain of spike with LA bound in the locked conformation (Toelzer et al., 2020) and the NTD of the NOVAVAX structure, which has better-defined loops for the NTD than previous structures (Bangaru et al., 2020; Toelzer et al., 2020). We compare the structural and dynamical responses of the variants to the wild-type protein. These simulations investigate how mutations, insertions, and deletions affect the allosteric pathways connecting the FA site to the rest of the protein. Several cryo-EM structures for Omicron BA.1 were recently solved (Cerutti et al., 2022; Gobeil et al., 2022; Zhang et al., 2022). The overall architecture of the FA binding site in the Omicron model, used here as the starting point for the equilibrium MD simulations, is similar to the cryo-EM structures 7TNW (Zhang et al., 2022) and 7TF8 (Gobeil et al., 2022), two experimental structures of the closed Omicron BA.1 spike, except for the region containing the gating helix (containing Y365 and Y369). In our model, the gating helix rotates outward to accommodate LA (Supplementary Figure S1). It is worth noting that in Omicron BA.1, despite the substitution of lysine in position 417 by asparagine, the key anchoring residues directly interacting with the carboxylate headgroup of LA, namely R408 and Q409, are still present (Supplementary Figure S1). Additionally, in Omicron, the region in the FA pocket accommodating the hydrophobic tail of LA is also slightly modified compared to the wild-type, due to the substitution of serine in position 373 by proline (Supplementary Figure S1). Overall, our Omicron model, which is based on the cryo-EM structure 6ZB5 with LA bound (Toelzer et al., 2020), has a more compact trimer architecture than 7TNW (Zhang et al., 2022) and 7TF8 (Gobeil et al., 2022) due to extra interactions between the LA molecule and the residues forming the FA site, mainly R408 and Q409 (Supplementary Figure S1).

We have also compared our Omicron model with the cryo-EM structure of Omicron sub-lineage BA.2 (PDB code 7UB0; Stalls et al., 2022) to identify variations within the FA binding site and understand how they may impact FA binding (Supplementary Figure S2A). Omicron BA.2, besides the K417N and S373P mutations (also present in Omicron BA.1), harbours an additional amino acid substitution, R408S, within the FA site (Supplementary Figure S2B). In this variant, the two positively charged residues (R408 and K417) providing the most dominant charge interactions with the carboxylate headgroup of LA are missing, replaced by two residues with hydrogen bonding potential. The combination of the K417N and R408S mutations may affect the ability of Omicron BA.2 to bind and stabilize FA molecules. As shown in Supplementary Figure S2A, the cryo-EM structure of Omicron BA.2 shows a highly contracted FA binding site compared to our Omicron model, mainly due to the changes in several residues of the hydrophobic and aromatic side chains that line the pocket, such as L368 and F377. Interestingly, Omicron BA.4 also contains the K417N and R408S mutations (Supplementary Figure S2B), suggesting a reduced ability of this sub-lineage to bind FAs such as LA.

In the D-NEMD approach, multiple configurations extracted from equilibrium MD simulations are used as starting points for nonequilibrium simulations, through which the effect of a perturbation can be studied (Oliveira et al., 2021a). Equilibrium trajectories for the locked, non-glycosylated and uncleaved (no cleavage at the furin recognition site) ectodomains of the wild-type, Alpha, Delta, Delta plus, and Omicron spikes bound with LA were taken from Shoemark et al. (2022). To probe how the substitutions, deletions, and insertions in each variant affect the dynamics of the spike, the Cα root-mean-square fluctuation (RMSF) differences between the wild-type and each variant were calculated using the equilibrium trajectories (Supplementary Figures S8–S11). Positive values correspond to a greater flexibility of the wild-type spike during the simulations, whereas negative values correspond to an increased flexibility of the variant. Although the profiles of the Cα RMSF differences between the wild-type and variants are generally similar (thus indicating similar dynamics), there are some discernible differences in key functional regions: Alpha shows higher fluctuations predominantly in two distinct regions, notably L249–D253 in the NTD and A829–F833 in the fusion peptide proximal region (FPPR); Delta shows enhanced dynamics in the segments V143–S161 and S247–D253 in the NTD, N679–V687 in the furin cleavage site, and A942–A944 in the heptad repeat 1; Delta plus has higher fluctuations in two main regions, namely W633–G639 in the C-terminal domain 2 of the S1 subunit and T678–V687 in the furin cleavage site; Omicron shows increased motions mainly in the regions Y248–S255 in the NTD, N481–V483 in the RBM, and T678–A688 in the furin cleavage site. Unsurprisingly, most of these differences are not statistically significant, e.g. the RMSF difference between Omicron and wild-type in Y248–S255 is associated with a high *P*-value. Nonetheless, a few regions, such as the furin cleavage site regions, still show statistically significant differences in dynamical behaviour between the wild-type and variants (Supplementary Figures S8–S11). To further identify the associated motions of the LA molecule when bound to the FA site, the statistical correlations between LA and all the Cα atoms of the protein were determined for the wild-type, Alpha, Delta, Delta plus, and Omicron systems (Supplementary Figure S12). As expected, the motions of LA are tightly coupled to the regions surrounding the FA site. The correlation values between LA and the rest of the protein are higher for Omicron (Supplementary Figure S12). Also, all variants show the increased motions between LA and NTD regions compared to the wild-type (Supplementary Figure S12).

In the D-NEMD approach, the time-dependent response of the protein to the perturbation is extracted by directly comparing equilibrium and nonequilibrium trajectories at equivalent points in time (Supplementary Figure S13). For each system, 87 short (10 ns) D-NEMD simulations were carried out starting from conformations extracted from the equilibrated part of the equilibrium MD simulations (Supplementary Figures S3–S7). Glycans are crucial to the biological functions of the spike, e.g. participating in shielding and infection by altering the dynamics of RBD opening (Casalino et al., 2020; Sztain et al., 2021). However, the cryo-EM structure of the wild-type spike in a locked conformation with LA bound (Toelzer et al., 2020) only contains glycans on the exterior. Thus, the allosteric communication networks and the response of the spike protein to an internal structural perturbation (here, LA removal) are unlikely to be qualitatively altered by the presence of glycans.

In D-NEMD simulations, the instantaneous deletion of the LA molecules from the FA site prompts the structural response of the protein as it adapts to LA removal. Such perturbation forces the system out of equilibrium and, in this way, creates the driving force necessary for the conformational changes of interest to occur. This driving force disappears once the simulations reach a new equilibrium state. Note that D-NEMD simulations are not intended to model the physical process of LA binding or dissociation. It should also be noted that, due to their short length (10 ns), the D-NEMD simulations performed here do not attempt to sample state transitions, such as the opening or closing of the RBD. Instead, these simulations allow for the identification of the first steps involved in the propagation of the structural changes within the protein and the order of the events associated with this process. Conformational rearrangements taking >10 ns are not be sampled, meaning that additional regions of the spikes may also respond to LA removal.

The perturbation used here, as well as in our previous work (Gupta et al., 2022; Oliveira et al., 2022), is designed to induce a rapid response and force signal transmission within the protein, hence allowing the mapping of the mechanical and dynamical couplings between the structural elements involved in this process. The time evolution of the response is extracted using the Kubo–Onsager relation (Ciccotti et al., 1979; Ciccotti, 1991; Ciccotti and Ferrario, 2016; Oliveira et al., 2021a). Multiple D-NEMD simulations are performed and compared with equilibrium trajectories to identify the protein's structural response. The response is averaged over multiple trajectories to remove the noise (Supplementary Figure S15; Ciccotti et al., 1979; Ciccotti, 1991; Ciccotti and Ferrario, 2016; Oliveira et al., 2021a). Given that the same perturbation (LA removal) is used for all systems, the structural responses of different variants can be directly and meaningfully compared. Note that the D-NEMD approach could determine the statistical significance of structural rearrangements (Supplementary Figures S16–S20; Oliveira et al., 2021a). We thus focus on the significant differences among the variants.

**Figure 2:**
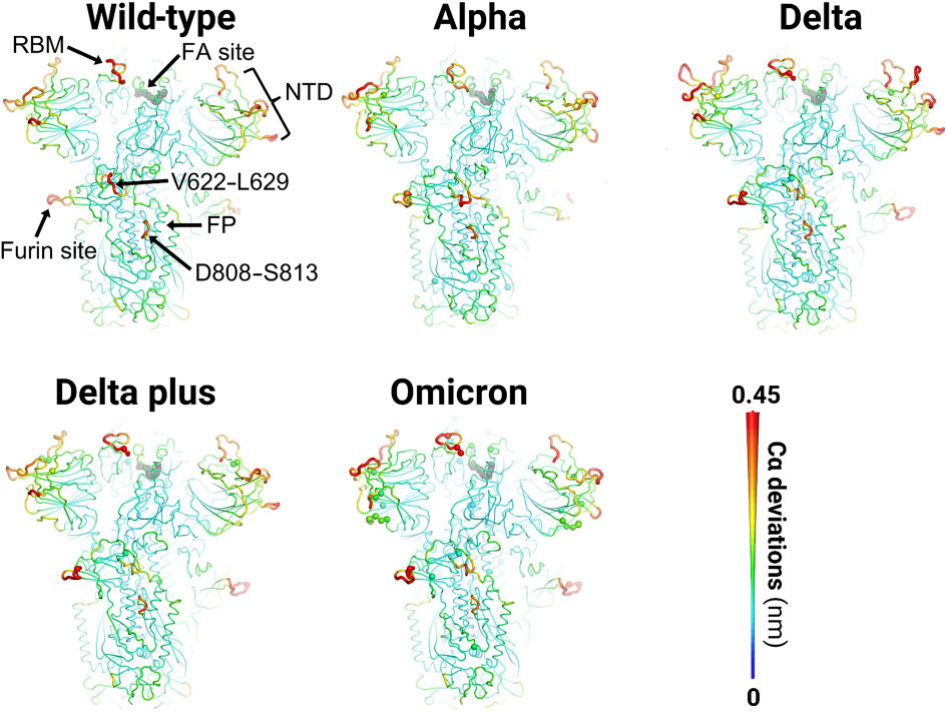
Structural responses of the wild-type, Alpha, Delta, Delta plus, and Omicron spikes at 10 ns after LA removal from the FA binding site. The Cα-positional deviation between the LA-free D-NEMD and LA-bound equilibrium MD simulations was calculated for each residue. The average values were obtained from the three chains of the trimer and from 87 pairs of simulations (Supplementary Figure S15). The average Cα deviations are mapped onto the starting structures for the equilibrium MD simulations, indicated by different colours (according to the scale on the right). Dark grey spheres highlight the FA binding sites. Other spheres pinpoint the positions of mutations.

**Figure 3:**
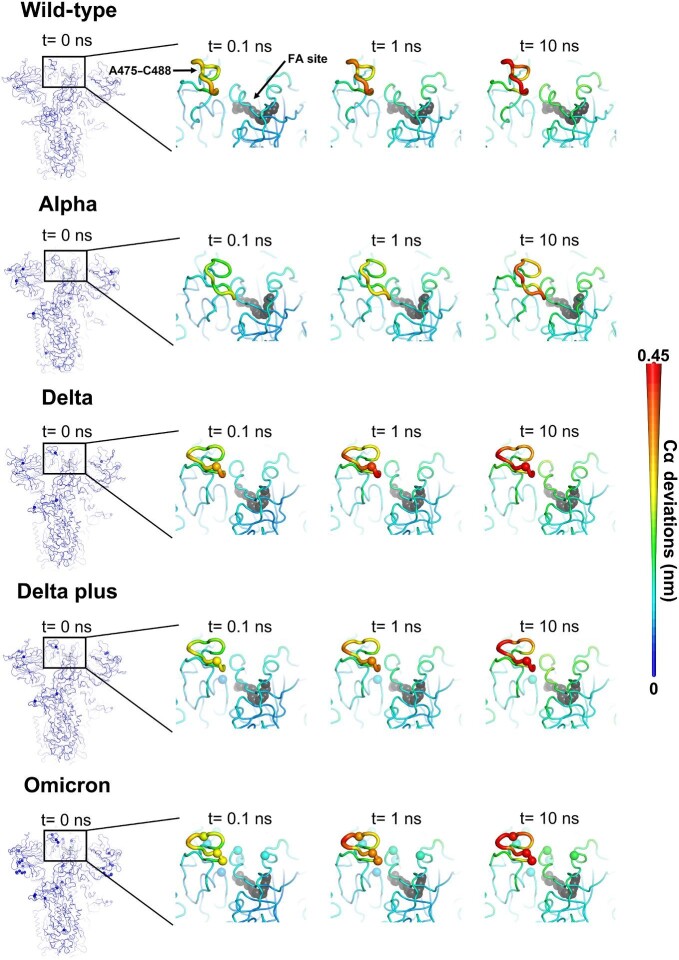
Structural responses of the RBM in the wild-type, Alpha, Delta, Delta plus, and Omicron spikes. The average Cα deviations are mapped onto the starting structures for the equilibrium MD simulations, indicated by different colours and cartoon thicknesses. Dark grey spheres highlight the FA binding sites. Other spheres show the positions of mutations.

**Figure 4:**
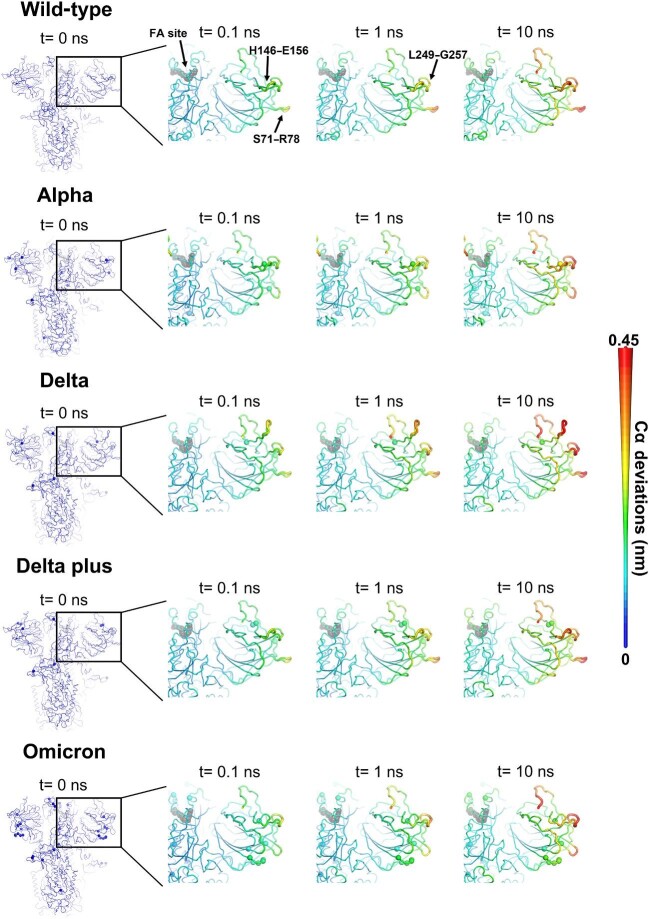
Structural responses of the NTD in the wild-type, Alpha, Delta, Delta plus, and Omicron spikes. The average Cα deviations are mapped onto the starting structures for the equilibrium MD simulations, indicated by different colours and cartoon thicknesses. Dark grey spheres highlight the FA binding sites. Other spheres pinpoint the positions of mutations.

D-NEMD simulations reveal the complex cascade of conformational changes induced by LA removal and identify the pathways through which these changes propagate within the protein. The structural response quickly propagates from the FA site to the specific and well-defined regions of the spike in all variants (Figure 2; Supplementary Figure S21). Several functional motifs, including the regions surrounding the FP, show significant structural responses in all variants (Figure 2). In the systems simulated, LA removal induces a conformational response in the FA pocket, which contracts due to the movements of the hydrophobic and aromatic sidechains that line it (Supplementary Figure S22). Structural changes are rapidly transmitted to the RBD and NTD, and then to V622–L629, furin cleavage site, and residues surrounding the FP (Supplementary Figure S21). As shown in Figures 2–4 and Supplementary Figures S28 and S29, at 0.1 ns after LA removal, significant structural rearrangements can be observed in the RBD, mainly in the RBM, and in specific regions of the NTD, such as S71–R78, H146–E156, and L249–G257. The RBM contains the residues that directly interact with the host ACE2 receptor (Lan et al., 2020; Wrapp et al., 2020) and is a known target for neutralizing antibodies (Robbiani et al., 2020; Rogers et al., 2020; Wec et al., 2020). The NTD is also a major target for neutralizing antibodies (Chi et al., 2020; Soh et al., 2020; Wang et al., 2020a; Cerutti et al., 2021). In particular, the H146–E156 and L249–G257 segments were shown to directly mediate the interactions between spikes and specific antibodies (e.g. 4A8 monoclonal antibody; Chi et al., 2020). The S71–R78 region is an antigenic epitope and has also been suggested to be involved in binding to other receptors besides ACE2 (Behloul et al., 2020).

**Figure 5:**
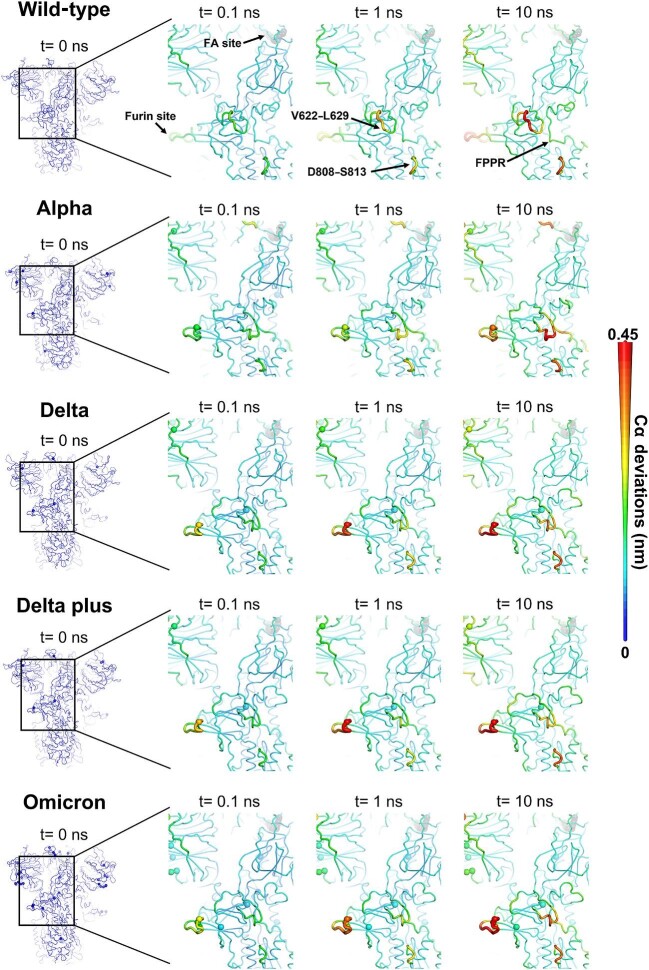
Structural responses of the furin cleavage site and FP-surrounding regions in the wild-type, Alpha, Delta, Delta plus, and Omicron spikes. The average Cα deviations are mapped onto the starting structures for the equilibrium MD simulations, indicated by different colours and cartoon thicknesses. Dark grey spheres highlight the FA binding sites. Other spheres pinpoint the positions of mutations.

The networks connecting the FA site to functional regions of the spike are similar in all variants, thus apparently conserved (Supplementary Figures S22–S24). Similar to that observed in the wild-type spike (Oliveira et al., 2022), the S366–A372 and R454–K458 segments transmit structural changes from the FA site to the RBM (Supplementary Figure S23), while the P337–A348, W353–I358, and C166–P174 regions mediate signal propagation to the NTD (Supplementary Figure S24) in all variants. Signal transmission to the S1/S2 interface and S2 subunit occurs via the C525–K537, F318–I326, and L629–Q644 regions (Supplementary Figure S25). However, there are significant differences among different variants in their allosteric responses to LA removal, in particular the propagation of structural changes in functionally important regions of the protein (Figures 3–5; Supplementary Figure S21).

Cross-correlation matrices were determined and mapped for the wild-type, Alpha, Delta, Delta plus, and Omicron systems in D-NEMD simulations (Supplementary Figure S26) and equilibrium MD simulations (Supplementary Figure S27). In the cross-correlation maps, cyan and blue regions represent moderately and significantly negative correlations, indicating residues moving in opposite directions (moving towards or away from each other) with the correlation values <0, while orange and red regions correspond to moderately and significantly positive correlations, indicating residues moving in similar directions with the correlation values >0. The cross-correlation maps from the LA-free D-NEMD trajectories (Supplementary Figure S26) demonstrate that all variants show the higher overall correlations (more extensive red and blue regions in the maps) compared to the wild-type, with Omicron showing the highest among all the simulated systems. Specifically, all systems show significantly positive correlations between the FA site and the RBD, as well as moderately negative correlations between the FA site and the furin cleavage site (Supplementary Figure S26). Additionally, while the wild-type shows moderately negative correlations between the FA site and the NTD, all variants show the tighter negative couplings (Supplementary Figure S26). Interestingly, the signal propagation pathway involving the segment C525–K537 (Supplementary Figure S25) can also be identified from the cross-correlation matrices, showing high correlations with the FA site (Supplementary Figure S26).

The cross-correlation maps computed from the LA-bound equilibrium trajectories (Supplementary Figure S27) demonstrate the slightly increased dynamic correlations of these regions, suggesting that the presence of LA in the FA site strengthens the dynamic communications between the FA site and the RBD (in particular the RBM), NTD, and furin cleavage site.

### Structural responses of the RBM in the wild-type, Alpha, Delta, Delta plus, and Omicron spikes

Significant variations in the responses of the RBM (mainly the A475–C488 segment) were observed among the five virus spike proteins (Figure 3; Supplementary Figures S21, S28, and S31–S34). In the Alpha spike, the response of A475–C488, the RBD region showing the strongest response to LA removal, is notably weaker and more diffuse (lower amplitude of the structural changes) than that in the wild-type protein (Figure 3; Supplementary Figures S21 and S28). In contrast, Delta, Delta plus, and Omicron, which all have mutations in the A475–C488 segment, show a stronger response than the wild-type spike (Figure 3; Supplementary Figures S21 and S28). Delta and Delta plus have a threonine-to-lysine substitution in position 478 (T478K). Omicron also contains the S477N and E484A mutations, in addition to T478K. The D-NEMD simulations indicate that these mutations alter the dynamics of signal transmission from the FA site to the RBM, and thus amplify the allosteric coupling between the FA site and the RBM (Figure 3; Supplementary Figures S21 and S28).

### Structural responses of the NTD in the wild-type, Alpha, Delta, Delta plus, and Omicron spikes

The responses of the NTD differ significantly among variants, particularly in the S71–R78, H146–E156, and L249–G257 segments (Figure 4; Supplementary Figures S21, S29, and S31–S34). For example, Delta shows enhanced structural rearrangements whereas Omicron shows reduced responses in the region H146–E156 compared to the wild-type protein, and Delta plus variant shows weaker responses than Delta (Figure 4; Supplementary Figure S29). These variants contain deletions and mutations either in, or in direct contact with, the H146–E156 region: Delta has a three-residue deletion in positions 156–158 (E156Δ, F157Δ, and R158Δ); Omicron harbours a three-residue deletion in positions 143–145 (V143Δ, Y144Δ, and Y145Δ); and Delta plus contains the E156Δ–F157Δ deletion plus an arginine-to-glycine mutation in position 158 (R158G). Therefore, R158G may mitigate the effects of the E156Δ–F157Δ deletion.

All variants show weaker structural responses of the S71–R78 segment than the wild-type spike (Figure 4; Supplementary Figures S21 and S29), probably due to mutations close to or in direct contact with S71–R78: Alpha includes a two-residue deletion in positions 69 and 70 (H69Δ–V70Δ); Delta and Delta plus contain a threonine-to-arginine substitution in position 19, which is located close to S71–R78; and Omicron contains an alanine-to-valine mutation in position 67 (A67V) in addition to the H69Δ–V70Δ deletion. It was recently found that in the Omicron spike, mutations in the RBD (e.g. E484A) may be compensated for by stabilizing mutations in the NTD (e.g. H69Δ–V70Δ and G142D) and the S2 domain (Javanmardi et al., 2022).

Alpha, Delta, and Omicron show the enhanced structural rearrangements of the L249–G257 region in the NTD relative to the wild-type spike (Figure 4; SupplementaryFigures S21 and S29), which is modulated by the deletions and substitutions in NTD regions adjacent to this loop: L18F and Y144Δ in Alpha, T19R in Delta, and G142D and V143Δ–Y145Δ in Omicron.

### Structural responses of the furin cleavage site and FP-surrounding regions in the wild-type, Alpha, Delta, Delta plus, and Omicron spikes

The rearrangements induced by LA removal are not restricted to the regions near the FA site, but propagate as far as the V622–L629 segment, furin cleavage site, and regions surrounding the FP (Figure 5; Supplementary Figures S21 and S29–S35). The furin cleavage site, which is located at the S1/S2 interface, shows significant differences in response to LA removal among variants, i.e. less impacted in Alpha than in the wild-type protein, wherease more affected in Delta, Delta plus, and Omicron (Figure 5; Supplementary Figures S21 and S30). These variants contain residue substitutions close to the furin cleavage site. The proline residue in position 681 is mutated to histidine in Alpha and Omicron (P681H) and to arginine in Delta and Delta plus (P681R). In addition to P681H, in Omicron, asparagine 679 is also replaced by lysine (N679K). The extra positively charged residues near the cleavage site (P681R in Delta and Delta plus and N679K in Omicron) strengthen the allosteric connection to the FA pocket (Supplementary Figures S31–S35). The addition of flanking positively charged residues to the P681–R685 stretch has been suggested to improve proteolytic processing (Whittaker, 2021).

The allosteric coupling between the FA site and V622–L629 is substantially weaker in the spike variants containing the D614G mutation (Delta, Delta plus, and Omicron) compared to the wild-type spike (Figure 5; Supplementary Figures S21 and S30–S35). D614G significantly reduces signal propagation and allosteric coupling between the FA site and V622–L629. The D614G mutation is located at the interface between two monomers, where it disrupts the trans-interface salt-bridge and hydrogen bond networks (Yurkovetskiy et al., 2020; Gobeil et al., 2021) and alters the dynamics of this region (Oliveira et al., 2022). The D614G substitution has been shown to increase transmission, infectivity, and viral fitness (Hou et al., 2020; Korber et al., 2020; Yurkovetskiy et al., 2020; Ozono et al., 2021; Plante et al., 2021; Volz et al., 2021; Zhou et al., 2021). Our results here indicate that it may play a role in limiting the allosteric effects of the FA site.

The regions surrounding the FP, notably D808–S813 and the FPPR, are also affected by LA removal (Figure 5; SupplementaryFigures S31–S35). The responses of D808–S813 among simulated variants are generally similar, showing only a slight decrease in Omicron (SupplementaryFigures S31–S35). This segment is located upstream of the FP and immediately preceding the S2′ protease cleavage site. The S2′ site is essential for infection (Takeda, 2022), and its cleavage is mediated by transmembrane protease serine 2 after the spike binding to ACE2 (Hoffmann et al., 2020; Takeda, 2022). Finally, responses of the FPPR are reduced in Delta, Delta plus, and Omicron compared to the wild-type protein (Supplementary Figures S31–S35). This diminished response indicates a weakened allosteric connection of the FPPR to the FA site. Thus, mutations in or close to the furin cleavage site and V622–L629, such as D614G (in Delta, Delta plus, and Omicron), H655Y and N679K (in Omicron), P681H (in Alpha and Omicron), and P681R (in Delta and Delta plus), alter the allosteric networks connecting the FA site to the regions surrounding the FP, particularly the FPPR.

## Discussion

In summary, our findings show that SARS-CoV-2 variants differ significantly in their allosteric responses to FA binding (Table 1). These differences are of potential functional importance in the regulation of viral infectivity by LA. It may also have implications for the efforts to target the FA site with natural, repurposed, or specifically designed ligands (Shoemark et al., 2021).

**Table 1:** Summary of structural responses of functional regions to LA removal in the Alpha, Delta, Delta plus, and Omicron spikes compared to the wild-type SARS-CoV-2 spike.

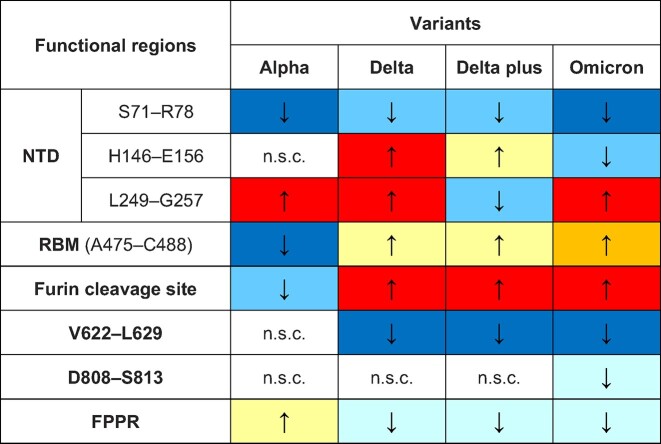

The **↑** and **↓** arrows indicate stronger and weaker responses, respectively, relative to the wild-type protein. Different colours represent different changes in the amplitude of the response relative to the wild-type protein. The light blue, sky blue, and dark blue colours represent a decrease of <5%, 5%–10%, and >10%, respectively. The yellow, orange, and red colours represent an increase of <5%, 5%–10%, and >10%, respectively. The white colour indicates no significant change (n.s.c.).

The allosteric connections in Alpha are generally similar to those in the wild-type spike protein, except for the RBM, S71–R78, and L249–G257 (Table 1). Delta and Delta plus exhibit significantly different responses of the NTD, furin cleavage site, and V622–L629, but not of the RBM (Table 1). Omicron, the most infectious variant simulated, displays significant changes in the responses of the NTD, RBM, furin cleavage site, and V622–L629 compared to the wild-type spike protein (Table 1). In Omicron, S71–R78, H146–E156, and V622–L629 exhibit weaker connections to the FA site, whereas the L249–G257 region, RBM, and furin cleavage site show stronger couplings to the FA site.

In Delta, Delta plus, and Omicron, the allosteric connection between the FA site and the furin cleavage site is increased compared to the wild-type spike, whereas the link to V622–L629 is diminished and the connection to the FPPR is weaker (Table 1). This indicates that mutations determine how the structural rearrangements are propagated in that region.

While all variants show similar networks connecting the FA site to functional regions of the protein, there are statistically significant differences in their responses. Substitutions, insertions, and deletions affect the amplitude of the structural responses of specific regions in the S1 and S2 subunits and alter the rates at which these rearrangements propagate to them. While some mutations (such as L18F, T19R, G142D, E156Δ–F157Δ, T478K, and P681H/R) strengthen the links to the FA pocket, others (such as H69Δ–V70Δ, Y144Δ, and D614G) abate them.

The coupling of the FA site to the NTD, in particular to the S71–R78, H146–E156, and L249–G257 segments, is greatly affected by mutations in and around these regions. While deletions around position 156 (E156Δ–F157Δ in Delta) enhance the allosteric connection to the FA site, deletions around position 144 (V143Δ–Y145Δ in Omicron) diminish that connection. Deletions near position 71 (H69Δ–V70Δ in Alpha) weaken the response of S71–R78 and the allosteric link to the FA site. Mutations in the regions next to L249–G257 (e.g. L18F in Alpha, T19R in Delta, and G142D in Omicron) can amplify the structural rearrangements induced by LA removal, reinforcing the connection to the FA pocket.

The D-NEMD simulations also reveal that substitutions leading to an increased positive charge in the region preceding the furin cleavage site (i.e. P681R in Delta and Delta plus and N679K in Omicron) apparently reinforce the allosteric link to the FA pocket. Conversely, the D614G mutation in Delta, Delta plus, and Omicron significantly reduces the coupling of V622–L629 to the FA site.

Our results demonstrate that D-NEMD simulations are a valuable tool to study allostery, identify and explore the allosteric networks operating within the spike, and predict the impact of future mutations in these pathways. D-NEMD simulations provide an effective approach to characterize allosteric effects (Gupta et al., 2022; Oliveira et al., 2022), effects of pH changes (Dommer et al., 2023), and other functionally important properties, and will help to investigate further emerging variants. We note that the simulations here compare spike proteins without glycans, as discussed above and elsewhere (Oliveira et al., 2022), considering that the presence of glycans (covering the outside of the protein) is unlikely to qualitatively alter the internal mechanical response of the protein. The differences in allosteric response to LA among variants revealed here by D-NEMD simulations may have functional relevance and should be investigated by experiments.

## Supplementary Material

mjad021_Supplemental_Files

## Materials and methods

### Equilibrium MD simulations and D-*NEMD simulations*

Details are described in Supplementary material.

### Data Availability

All D-NEMD simulation data (including input and trajectory files) are openly available from the MolSSI/BioExcel COVID-19 public data repository for biomolecular simulations of COVID proteins (https://covid.molssi.org/simulations/).
