# Supplementary material for: SARS-CoV-2 spike variants differ in their allosteric responses to linoleic acid
Source: J Mol Cell Biol. 2023 Mar 29;15(3):mjad021. doi: 10.1093/jmcb/mjad021 (PMC10563148; doi:10.1093/jmcb/mjad021)
Supplement: mjad021_Supplemental_Files [file mjad021_supplemental_files.zip › JMCB-2022-0155.R2_Supplementary Figures and Text.pdf]

Supplementary Material

SARS-CoV-2 spike variants differ in their allosteric response to linoleic acid

A. Sofia F. Oliveira, Deborah K. Shoemark, Andrew D. Davidson, Imre Berger, Christiane Schaffitzel and Adrian J. Mulholland

Materials and methods

Equilibrium simulations

The sequence for the early 2020 spike (hereafter named wild type) was taken from Uniprot P0DTC2 (https://www.uniprot.org/uniprot/P0DTC2). The sequences for Alpha (B.1.1.1.7), Delta (B.1.617.2) and Delta Plus (B1.617.2-AY1) were taken from UK-COG (https://www.cogconsortium.uk/). The sequences for Omicron BA.1 (hereafter named Omicron) were taken from https://www.gisaid.org as EPI-ISL-6640916. The Alpha variant comprises seven mutations and three deletions in each spike monomer, namely L18F, H69Δ-V70Δ, Y144Δ, N501Y, A570D, P681H, T716I, S982A, D1118H. The Delta variant contains six mutations and three deletions in the spike (T19R, E156Δ-R158Δ, L452R, T478K, D614G, P681R and D950N), whereas the Delta Plus has eight mutations and two deletions (T19R, E156Δ-F157Δ, R158G, K417N, L452R, T478K, D614G, P681R and D950N). The Omicron variant bears 40 substitutions, deletions and insertions (A67V, H69Δ-V70Δ, T95I, G142D, V143Δ-Y145Δ, N211I, L212Δ, D215E, PED insertion, G339D, S371L, S373P, S375F, K417N, N440K, G446S, S477N, T478K, E484A, Q493R, G496S, Q498R, N501Y, Y505H, T547K, D614G, H655Y, N679K, P681H, N764K, D796Y, N856K, Q954H, N969K and L981F), 15 of them located in the RBD.

The models for the non-glycosylated and uncleaved (no cleavage at the furin site located in the S1/S2 interface) locked ectodomain of the wild type, Alpha, Delta, Delta plus and Omicron with linoleic acid (LA) bound were taken from (Shoemark et al., 2022). The model for the wild-type spike with LA was based on the cryo-EM structures with PDB codes 7JJI (Bangaru et al., 2020) and 6ZB5 (Toelzer et al., 2020). Note that both cryo-EM structures used to build the model for the wild-type spike contains glycans on the exterior (Bangaru et al., 2020; Toelzer et al., 2020): limited glycosylation occurs in the structure 6ZB5 because it was expressed in insect cells (Toelzer et al., 2020); site-specific glycosylation analysis detected glycosylation in all 22 N-linked glycan sites in the structure 7JJI (Bangaru et al., 2020). Both cryo-EM structures (pdb codes: 6ZB5 and 7JJI) contain 2-acetamido-2-deoxy-beta-D-glucopyranose-(1-4)-2-acetamido-2-deoxy-beta-D-glucopyranose oligosaccharides and 2-acetamido-2-deoxy-beta-D-glucopyranose monosaccharides (Bangaru et al., 2020; Toelzer et al., 2020). The models for the Alpha, Delta, Delta plus and Omicron spikes with LA bound were constructed using as template the model for the wild-type spike described above (for more details, see (Shoemark et al., 2022)).

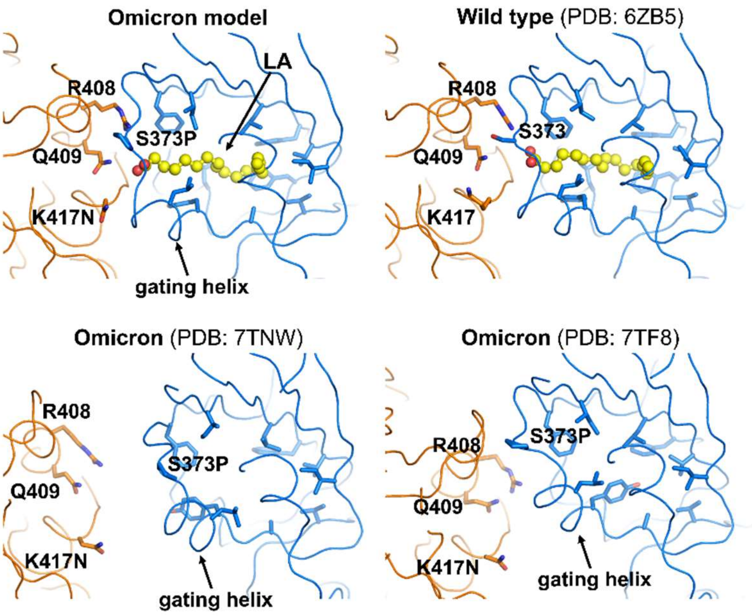

Supplementary Figure 1. The fatty acid binding site shown for comparison in: the Omicron model used as the starting point for equilibrium simulations; the cryo-EM structure of the wild-type spike with LA bound that was used as the template for building the Omicron model (PDB code: 6ZB5 (Toelzer et al., 2020)); and two recently released cryo-EM structures of Omicron BA.1 in the closed state (without LA bound) (PDB codes: 7TNW (Zhang et al., 2022) and 7TF8 (Gobeil et al., 2022)). Note that the overall structure of the FA binding site between the model and the experimental Omicron structures 7TNW and 7TF8 is similar, except for the shift in the gating helix (containing Y365 and Y369). In our model, this helix moves outward to accommodate LA. It is also worth noting that in Omicron BA.1, although K417 is mutated to an asparagine, the important anchoring residues for LA binding, namely R408 and Q409, are still present. Our Omicron model has a more compact trimer architecture because of the extra interactions between LA and the residues forming the FA site. In the 7TNW (Zhang et al., 2022) and 7TF8 (Gobeil et al., 2022) structures, a gap between adjacent RBDs (coloured in orange and blue) is observed, thus placing the anchoring residues, R408 and Q409, several angstroms away from the LA headgroup.

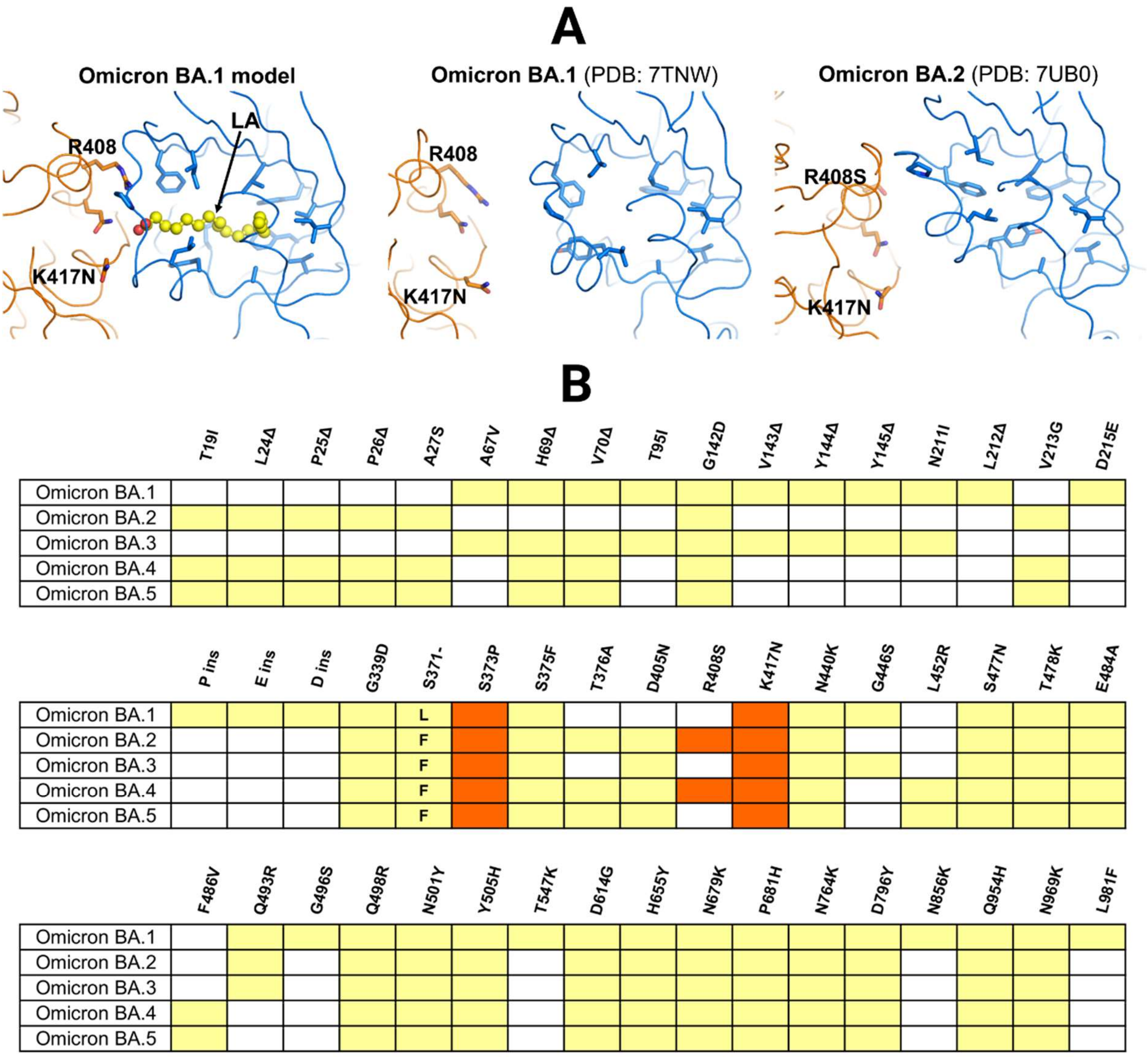

Supplementary Figure 2. A. The Omicron BA.1 model with LA bound used for the equilibrium simulations and the cryo-EM structures of Omicron BA.1 (PDB code: 7TNW (Zhang et al., 2022)) and BA.2 in the closed (with the three RBDs down) state (PDB code: 7UB0 (Stalls et al., 2022)). **B.** Residue changes between Omicron sub-lineages. The yellow colour highlights the position of changes relative to the wild type spike whereas white indicated no changes. Changes in the FA site are highlighted in dark orange. Note that in both Omicron BA.2 and BA.4, R408 and K1417 were replaced by polar residues, serine and asparagine respectively. Please zoom in to the image for detailed visualisation of the differences between sub-lineages.

The equilibrium simulations of the wild type, Alpha, Delta, Delta plus and Omicron used here as the starting point for the dynamical-nonequilibrium (D-NEMD) simulations were taken from (Shoemark et al., 2022). All MD simulations here used the same conditions and protocols as applied successfully previously (Gupta et al., 2022; Oliveira et al., 2022; Shoemark et al., 2021; Toelzer et al., 2020). Each spike trimer was simulated in a box of explicit waters with 150 mM NaCl, under periodic boundary conditions as an NPT ensemble at 310 K and pH 7, as described in (Shoemark et al., 2022). Three replicate simulations, each 200 ns, were performed for each spike system using GROMACS (Abraham et al., 2015).

All systems remained stable over the simulation time (200 ns), and the average Cα root mean square deviation (RMSD) profiles reached a plateau after 50 ns (panel A in Supplementary Figures 3-7). The stability of the systems is further demonstrated by the analysis of the secondary structure of the proteins. In all systems, the protein secondary structure remained intact (panel B in Supplementary Figures 3-7). Principal component analysis (PCA) was also used to check the equilibration/relaxation and sampling of the replicates (Garton and Laughton, 2013; Ng et al., 2013; Roy and Laughton, 2010). All replicates were considered equilibrated after 50 ns (panel C in Supplementary Figures 3-7). As expected, PCA showed that the different replicates explore different regions of conformational space, thus improving the overall sampling for each system.

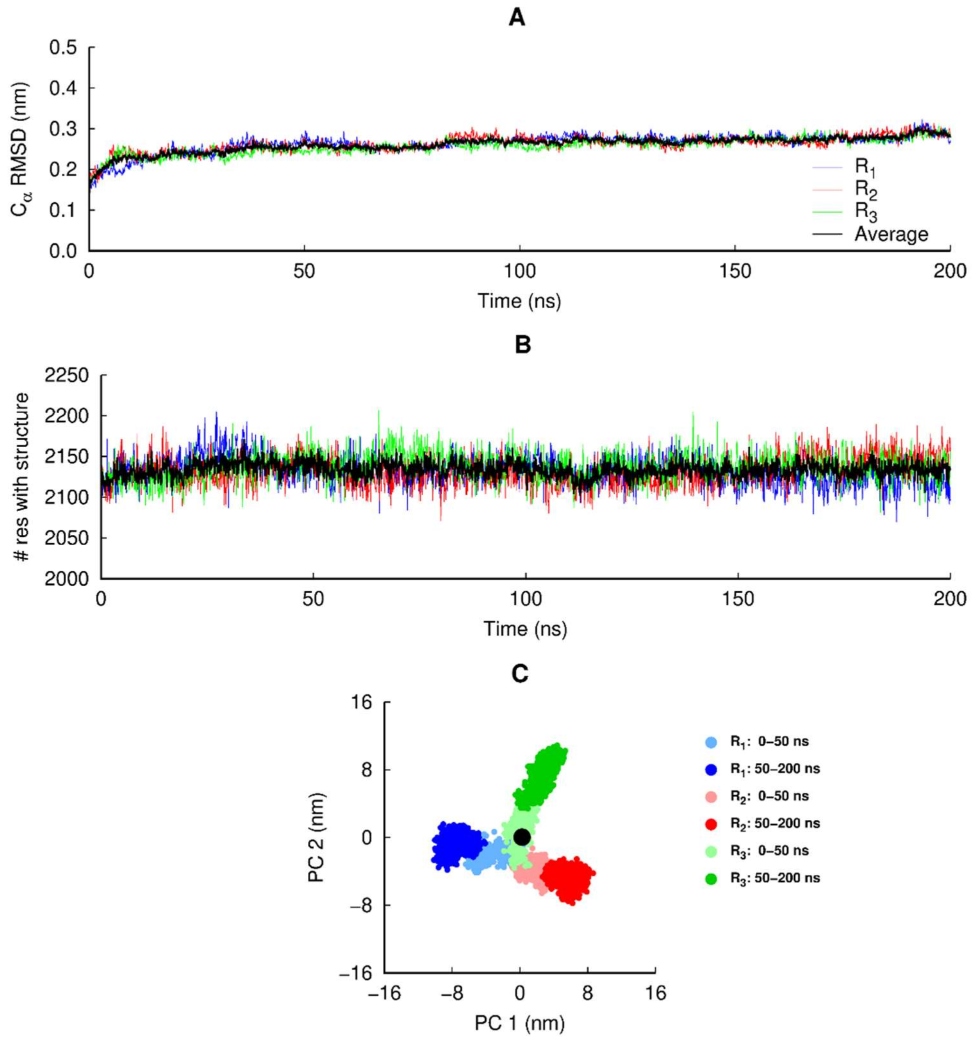

Supplementary Figure 3. Structural stability, equilibration and sampling of the equilibrium simulations of the wild-type spike. **A.** Temporal evolution of the C_α RMSD for each replicate individually and averaged C_α RMSD for the wild-type spike. The RMSD is relative to the starting structure. The black line corresponds to the average C_α RMSD, averaged over all 3 replicates. These plots indicate that the systems are stable over the simulation time. **B.** Secondary structure (assigned by DSSP (Kabsch and Sander, 1983)) during simulations, showing numbers of residues assigned in total to α-helix, 3_{10}- helix, 5-helix, β-sheet and β-bridge secondary structure classes. The black line corresponds to the average number of residues with secondary structure, averaged over all 3 replicates. The blue, red and green lines

correspond to number of residues with secondary structure in replicate 1, 2 and 3, respectively. **C.** PCA of all replicates for the wild-type spike. All 3 replicates were combined for analysis, with one conformation per 100 ps per replicate (totalling 6001 frames) for all the Cα atoms. The black dot corresponds to the structure used as the starting point.

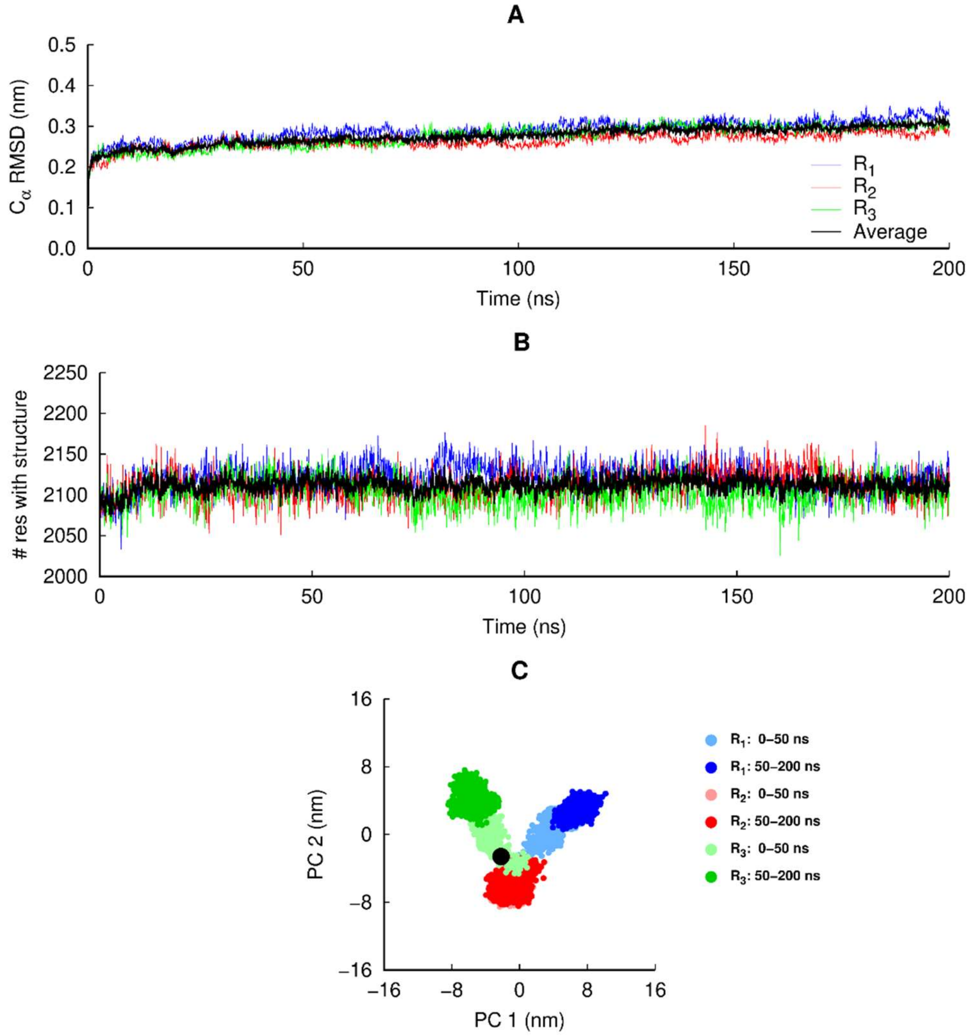

Supplementary Figure 4. Structural stability, equilibration and sampling of the equilibrium simulations of the Alpha spike. **A.** Temporal evolution of the C_α RMSD for each replicate individually and average C_α RMSD. **B.** Secondary structure during simulations. **C.** PCA for the Alpha spike. For more information, see the caption of Supplementary Figure 3.

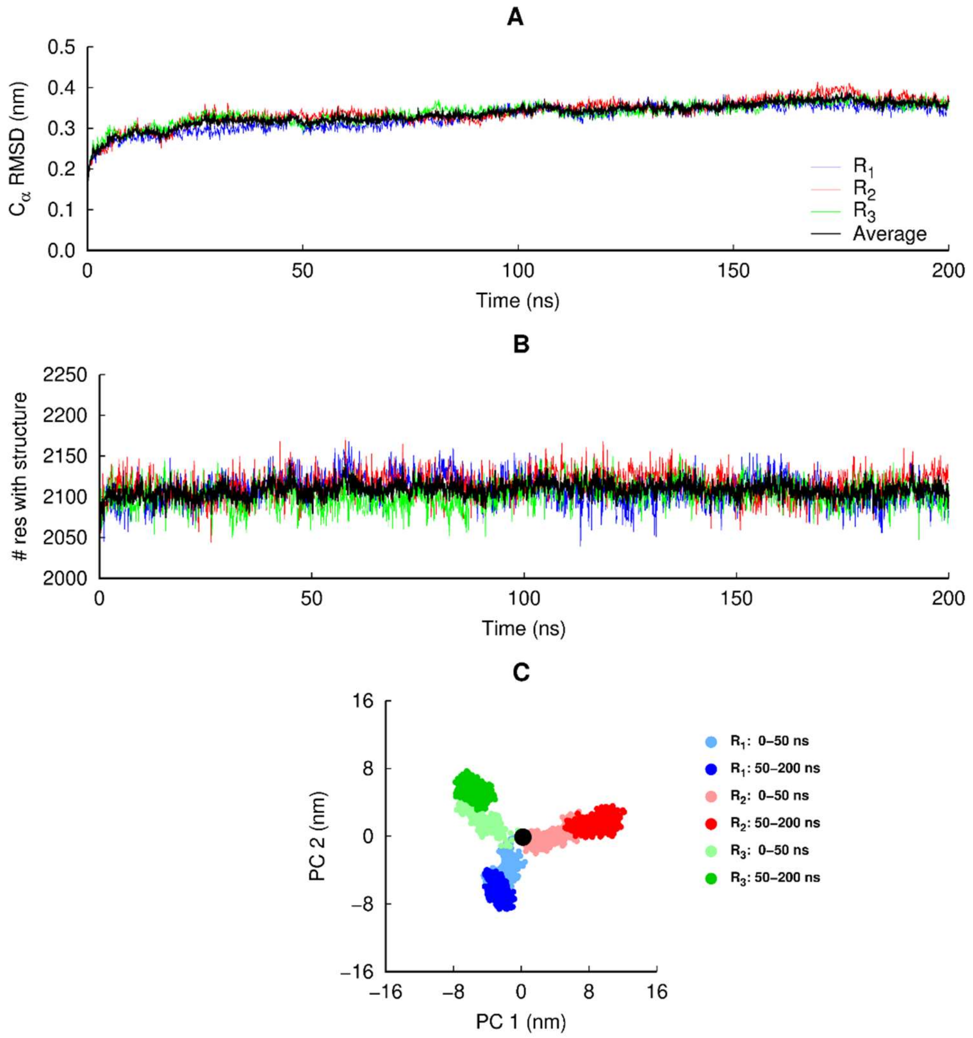

Supplementary Figure 5. Structural stability, equilibration and sampling of the equilibrium simulations of the Delta spike. **A.** Temporal evolution of the C_α RMSD for each replicate individually and for averaged C_α RMSD. **B.** Secondary structure during simulations. **C.** PCA for the Delta spike. For more information, see the caption of Supplementary Figure 3.

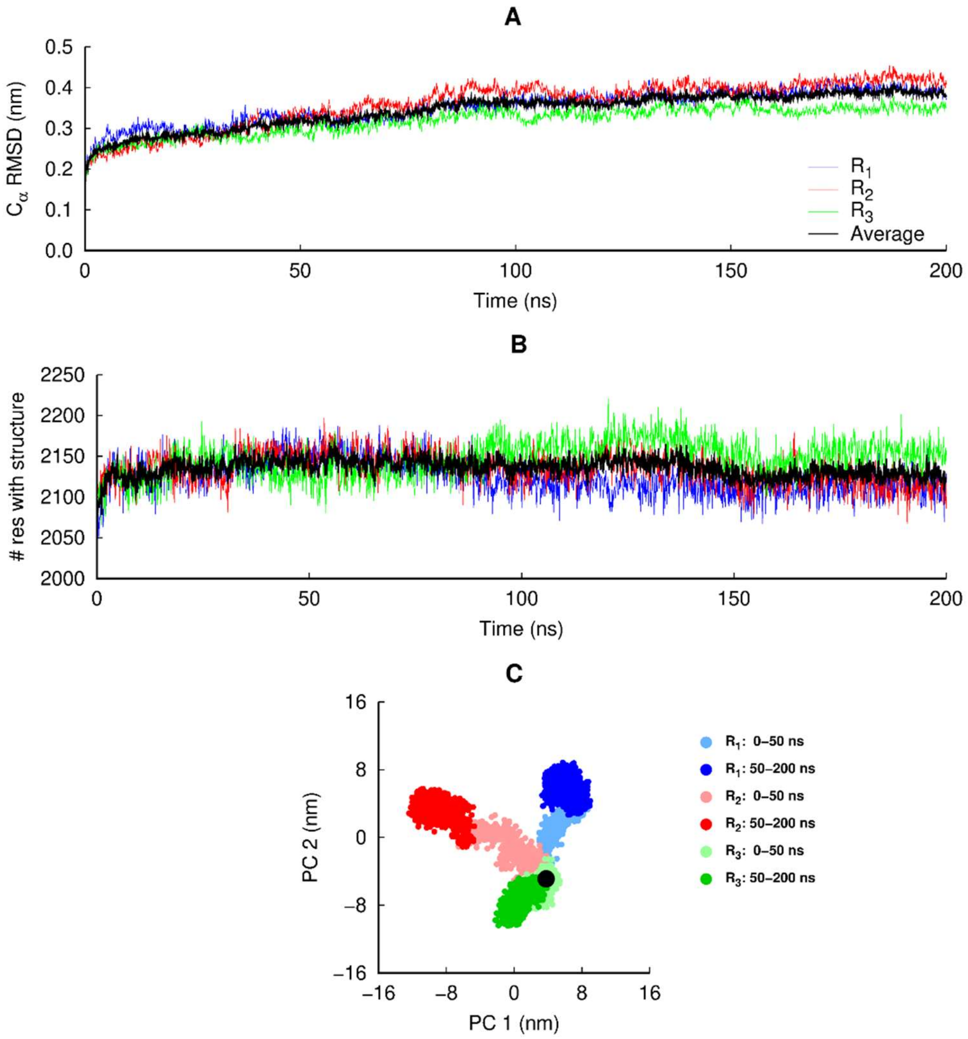

Supplementary Figure 6. Structural stability, equilibration and sampling of the equilibrium simulations of the Delta plus spike. **A.** Temporal evolution of the C_α RMSD for each replicate individually and averaged C_α RMSD. **B.** Secondary structure during simulations. **C.** PCA for the Delta plus spike. For more information, see the caption of Supplementary Figure 3.

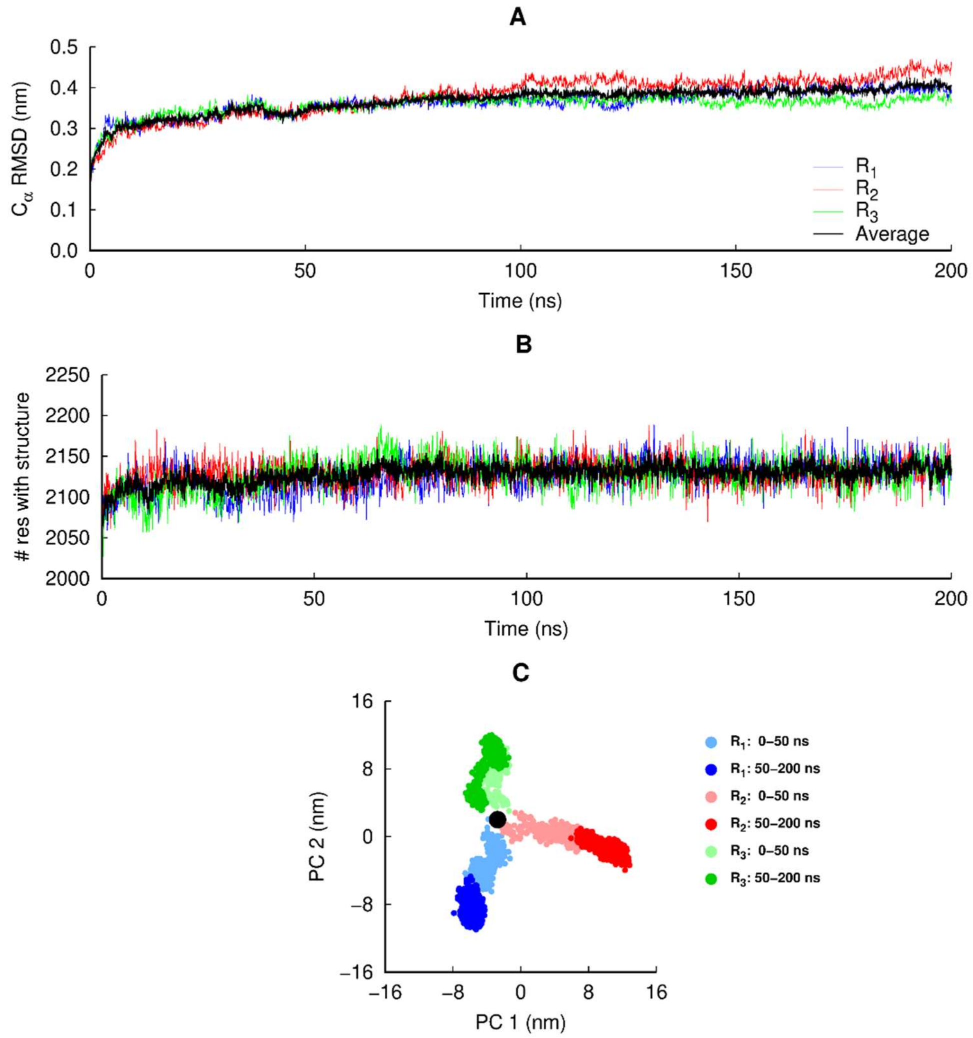

Supplementary Figure 7. Structural stability, equilibration and sampling of the equilibrium simulations of the Omicron BA.1 spike. **A.** Temporal evolution of the C_α RMSD for each replicate individually and averaged C_α RMSD. **B.** Secondary structure during simulations. **C.** PCA for the Omicron BA.1 spike. For more information, see the caption of Supplementary Figure 3.

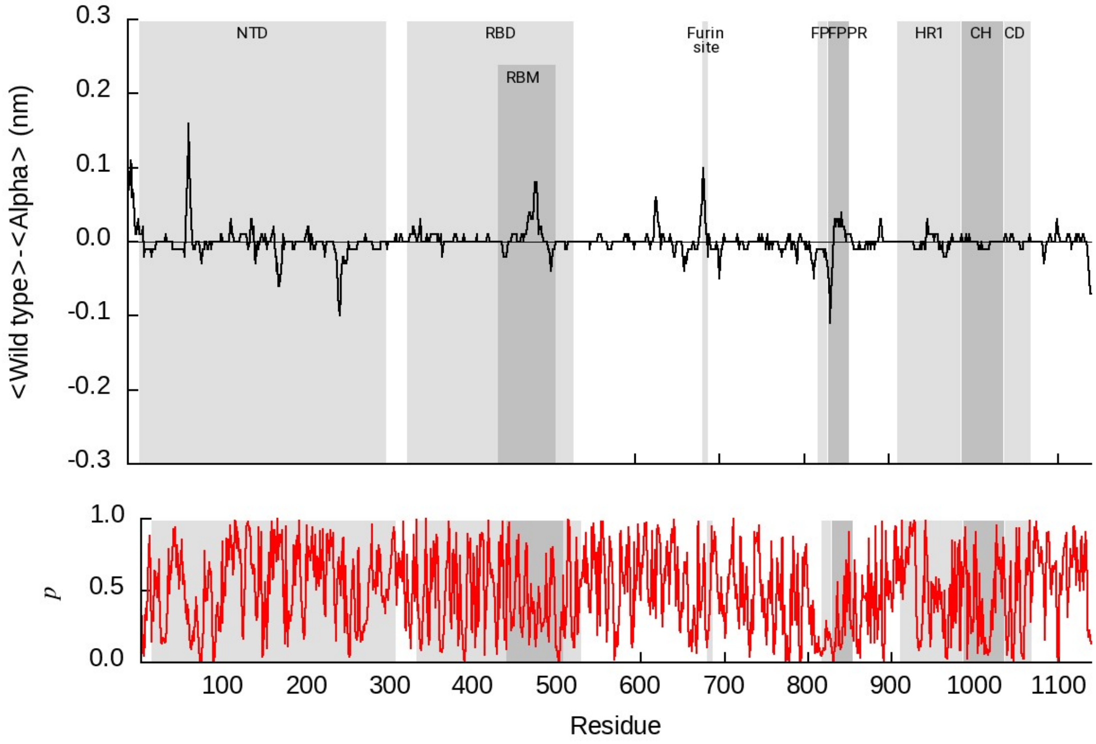

Supplementary Figure 8. Average change in RMSF between the wild-type and Alpha spike and associated p-values (Roy and Laughton, 2010). The positions of some important structural motifs are highlighted in grey, namely the N-terminal domain (NTD), receptor-binding domain (RBD), receptor-binding motif (RBM), fusion peptide (FP), fusion-peptide proximal region (FPPR), heptad repeat 1 (HR1), central helix (CH), connector domain (CD). Please zoom in to the image for detailed visualisation.

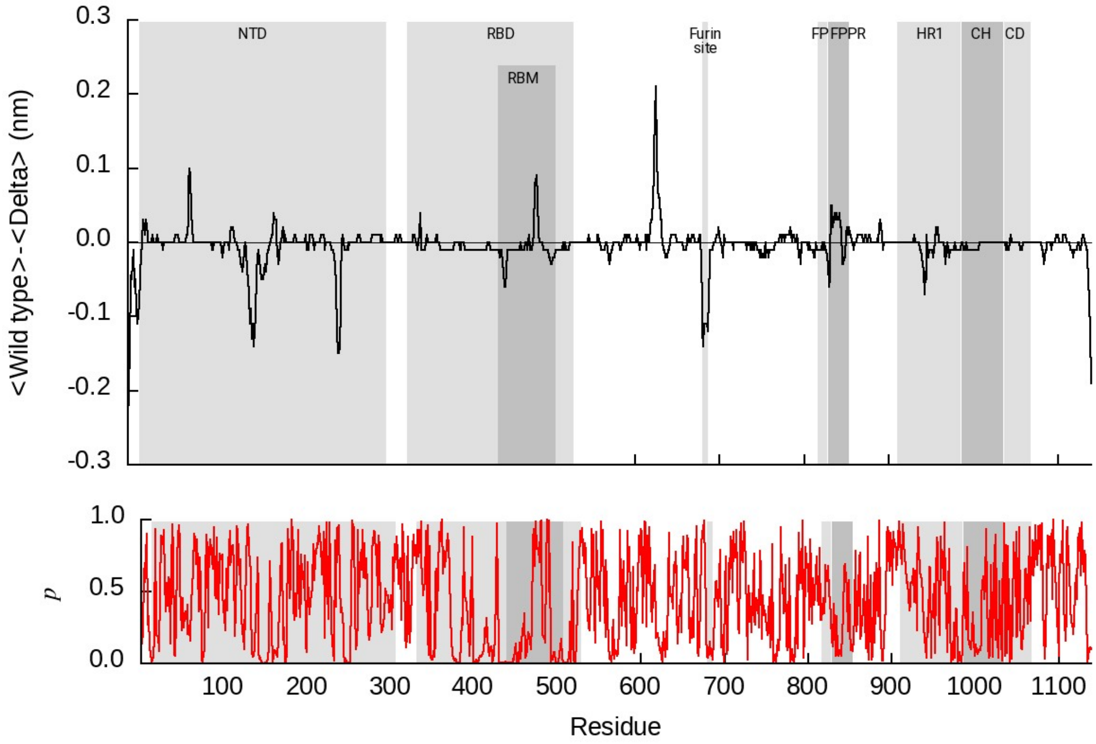

Supplementary Figure 9. Average change in RMSF between the wild-type and Delta spike and associated *p*-values (Roy and Laughton, 2010). For more details, see legend of Supplementary Figure 8. Please zoom in to the image for detailed visualisation.

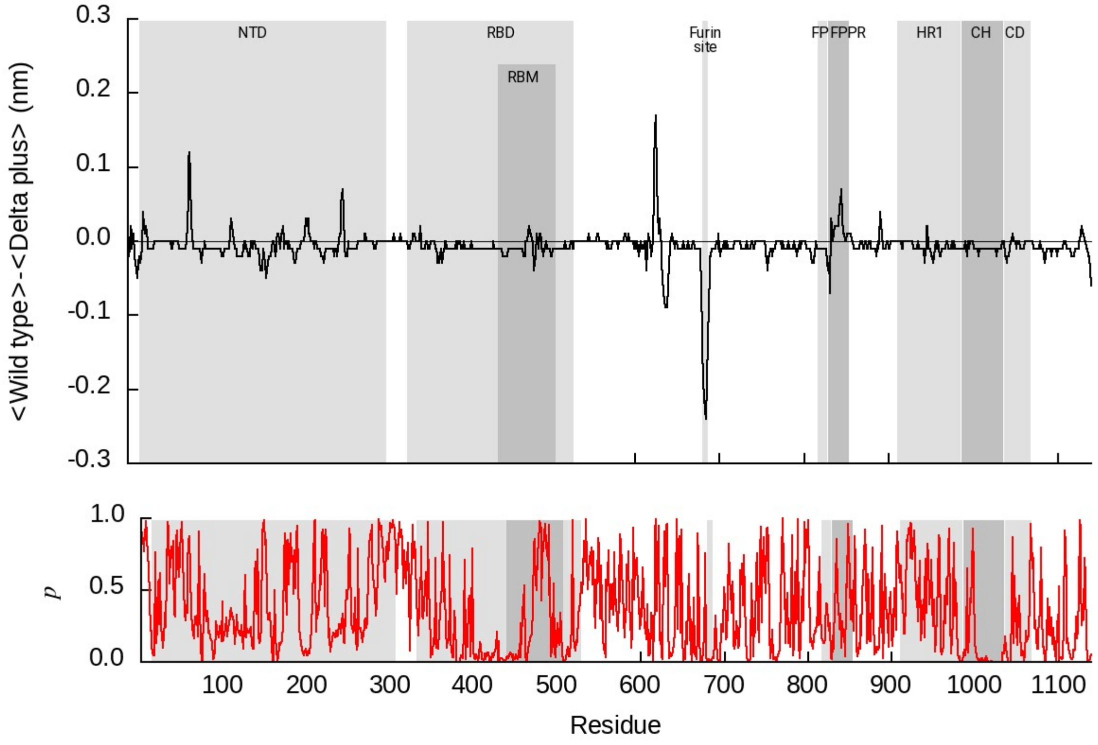

Supplementary Figure 10. Average change in RMSF between the wild-type and Delta plus spike and associated *p*-values (Roy and Laughton, 2010). For more details, see the legend in Supplementary Figure 8. Please zoom in to the image for detailed visualisation.

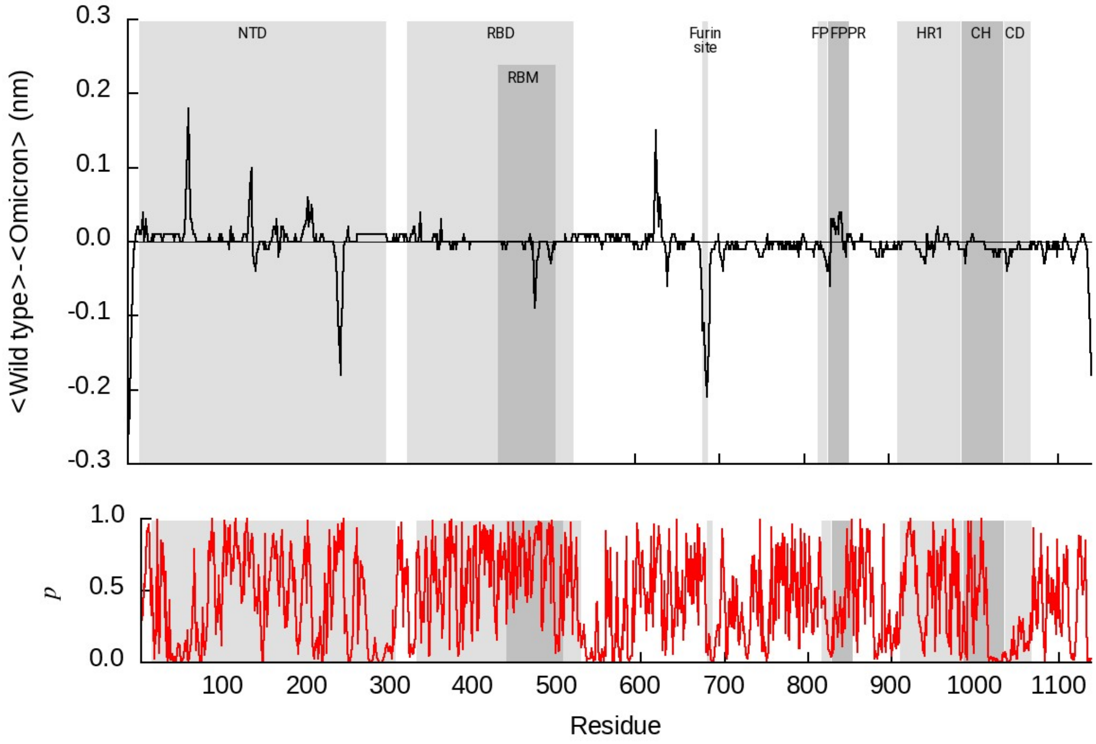

Supplementary Figure 11. Average change in RMSF between the wild-type and Omicron spike and associated *p*-values (Roy and Laughton, 2010). For more details, see the legend in Supplementary Figure 8. Please zoom in to the image for detailed visualisation.

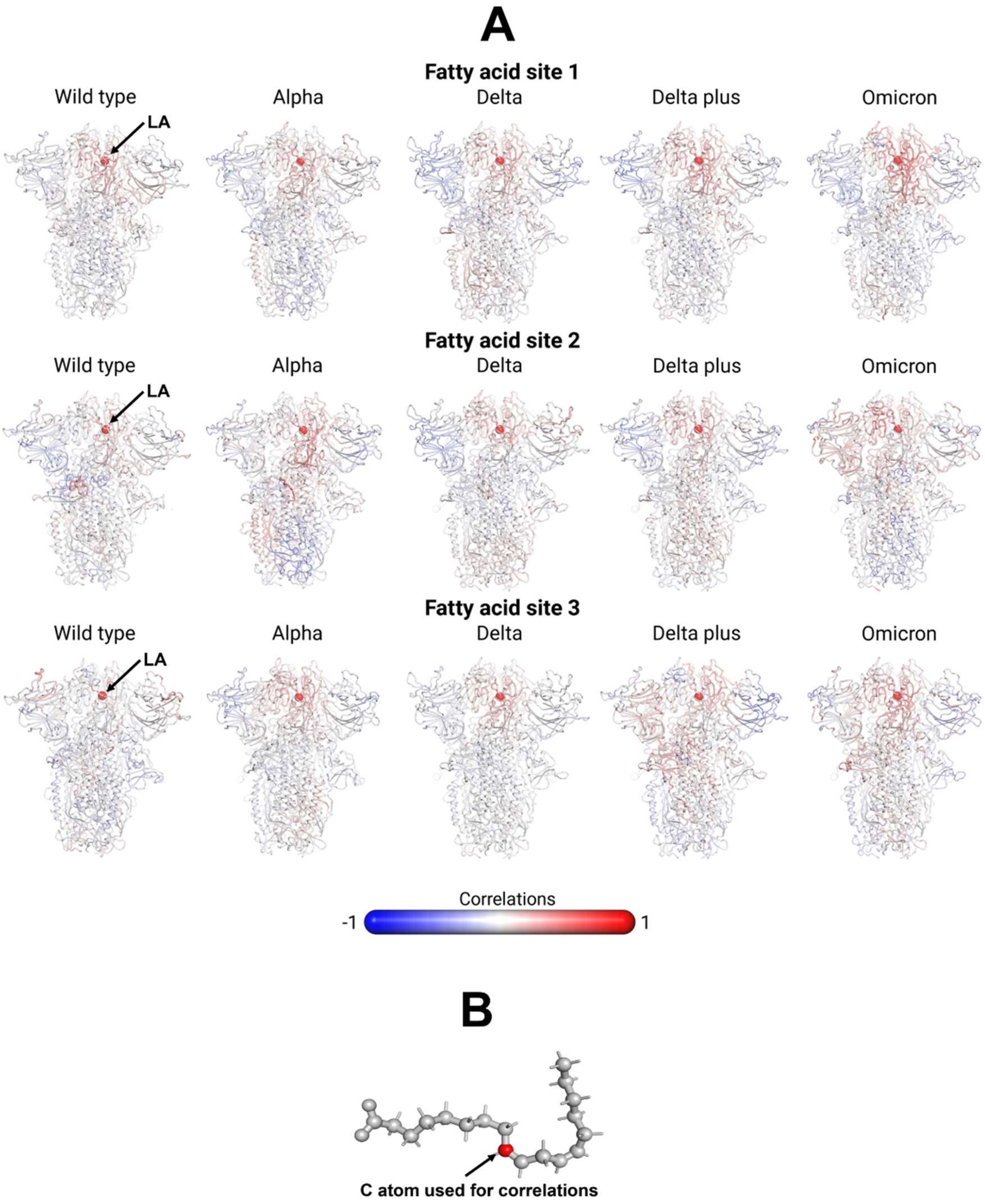

Supplementary Figure 12. **A.** Statistical correlation between LA and all the Cα atoms of the protein for the wild-type, Alpha, Delta, Delta plus and Omicron systems. Correlations are indicated by the colour scale shown between red and blue. The atoms that systematically move along the same direction have a correlation value higher than zero, while those moving in opposite directions have a correlation value lower than 0. The atoms whose movements are uncorrelated have a correlation value of 0. Please zoom in to the image for detailed visualisation of the correlated motions. **B.** Location of the carbon atom of LA used for the calculation of the statistical correlations shown in panel A.

D-NEMD simulations

D-NEMD simulations were performed to study the structural response to LA removal in the unglycosylated, uncleaved wild-type, Alpha, Delta, Delta Plus and Omicron spike from SARS-CoV-2. Eighty-seven nonequilibrium simulations were carried out for each variant, a total of 435 simulations. D-NEMD simulations have successfully been used to study allostery in various biomolecular systems (Abreu et al., 2020; Damas et al., 2011; Galdadas et al., 2021; Oliveira et al., 2019a; Oliveira et al., 2019b), including the SARS-CoV-2 spike (Gupta et al., 2022; Oliveira et al., 2022). Recently, this approach was used to identify the allosteric networks connecting the fatty acid binding site to key functional motifs on the wild-type, D614G (Oliveira et al., 2022) and BriSΔ (a variant containing an eight amino-acid deletion in the furin recognition motif and S1/S2 cleavage site) spikes (Gupta et al., 2022).

Briefly, in the D-NEMD approach, the response of a system to an external perturbation (in this case, LA removal from the fatty acid binding sites) can be directly computed using the Kubo-Onsager relation (Ciccotti, 1991; Ciccotti and Ferrario, 2016; Ciccotti et al., 1979; Oliveira et al., 2021) and by calculating the difference of a given property between the simulations with and without the perturbation. Subtracting the perturbed and unperturbed pairs of simulations at a given time and averaging the results over tens/hundreds of replicates allows not only the identification of the events associated with signal propagation, but also the determination of the statistical significance of the observations (Ciccotti, 1991; Ciccotti and Ferrario, 2016; Ciccotti et al., 1979; Oliveira et al., 2021). Here and similarly to our previous spike work (Gupta et al., 2022; Oliveira et al., 2022), the perturbation was generated by the (instantaneous) removal of the LA molecules from the fatty acid (FA) binding sites. A graphical representation of the procedure used to set up the D-NEMD simulations is shown in the top panel of Supplementary Figure 13.

The starting conformations for the D-NEMD simulations were obtained from the equilibrated part (50-200 ns) of the equilibrium LA-bound simulations of the wild-type, Alpha, Delta, Delta Plus and Omicron systems (Supplementary Figures 3-7). Conformations were taken every five ns, and in each frame, the LA molecules were (instantaneously) deleted from the FA pockets. The resulting apo system was then simulated for 10 ns (Supplementary Figure 13). The simulation conditions for all the nonequilibrium simulations were identical to the equilibrium simulations described in (Shoemark et al., 2022). 87 short (10 ns long) apo D-NEMD simulations were performed for each system. For more details of the setup of the D-NEMD simulations, see refs (Gupta et al., 2022; Oliveira et al., 2022).

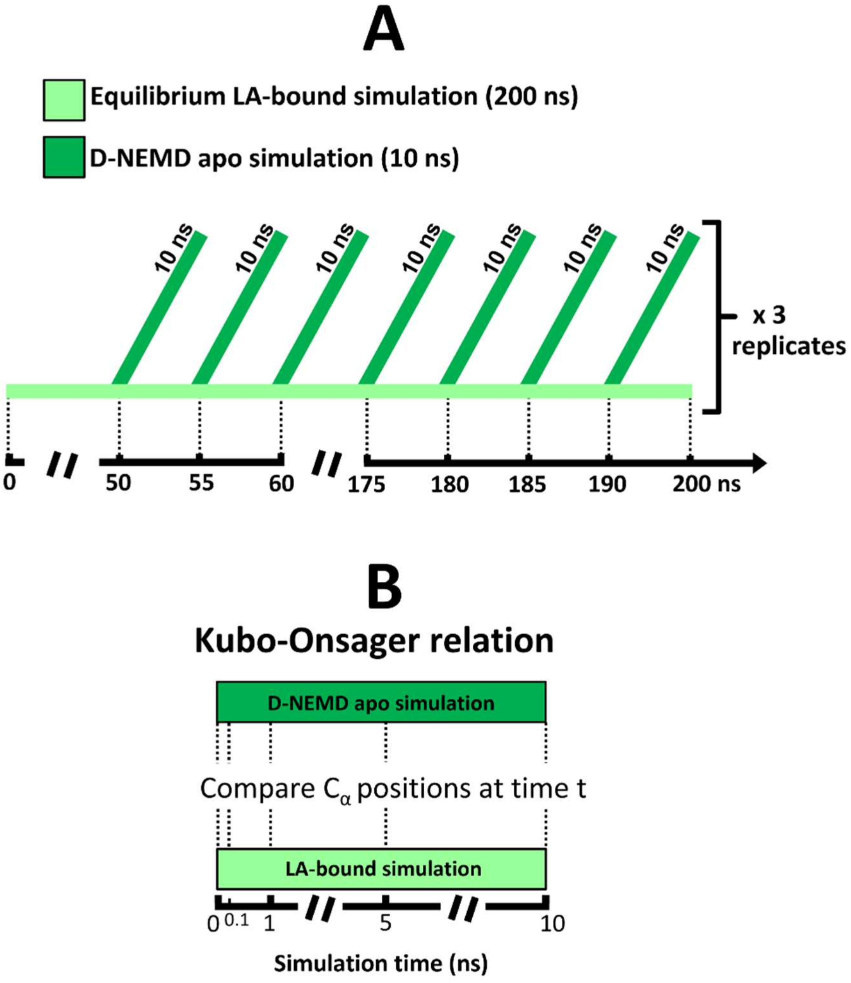

Supplementary Figure 13. Schematic description of the procedure used to set up (**A**) and analyse (**B**) the D-NEMD simulations. For all systems, namely the wild-type, Alpha, Delta, Delta Plus and Omicron spike, 3 equilibrium MD simulations, 200 ns each, were performed for the unglycosylated and uncleaved (no cleavage at the S1/S2 interface) locked spike. These equilibrium simulations (light green rectangles) were then used to generate starting structures for the short apo D-NEMD simulations (dark green rectangles). From the equilibrated part of each LA-bound simulation (from 50-200 ns), conformations were extracted every five nanoseconds, and the perturbation was introduced. Each short D-NEMD simulation was simulated for 10 ns. The Kubo-Onsager (Ciccotti, 1991; Ciccotti and Ferrario, 2016; Ciccotti et al., 1979; Oliveira et al., 2021) approach was used to extract the response of the system to LA annihilation from the FA pockets (bottom panel). For that, for each pair of equilibrium LA-bound and D-NEMD apo simulations, the positional deviations of each Cα at equivalent times (namely 0, 0.1, 1, 5 and 10 ns) were determined and averaged over all the simulations.

The Kubo-Onsager (Ciccotti, 1991; Ciccotti and Ferrario, 2016; Ciccotti et al., 1979; Oliveira et al., 2021) approach was used to extract the response of the spike to LA removal (see the bottom panel in Supplementary Figure 13). For each pair of unperturbed LA-bound equilibrium and perturbed (apo) nonequilibrium simulations, the difference in positions for each Cα was determined at equivalent points in time, namely after 0, 0.1, 1, 3, 5 and 10 ns of simulation (Supplementary Figure 13). The pairwise comparison between the positions of Cα atoms

allows for the direct identification of the most important conformational rearrangements. The Cα-positional deviations between the equilibrium and nonequilibrium trajectories at each point in time were averaged over all simulations and over the three chains forming the trimer. The statistical significance of the structural changes identified here is demonstrated by the low standard error of the averages (Supplementary Figures 16-20).

Supplementary Figures

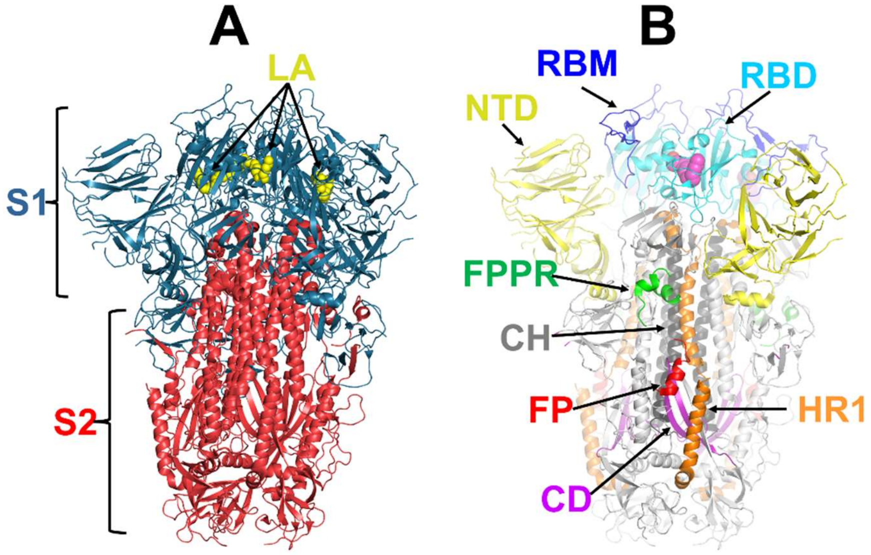

Supplementary Figure 14. A. Structure of the complex of the ectodomain of the SARS-CoV-2 spike trimer with linoleic acid (Toelzer et al., 2020). The S1 and S2 subunits are coloured blue and red, respectively. Linoleic acid (LA) molecules are highlighted with yellow spheres. **B.** Structure of the ectodomain of the spike trimer (Toelzer et al., 2020) with some relevant structural motifs highlighted: N-terminal domain (NTD)-yellow; receptor-binding domain (RBD)- cyan; receptor binding motif (RBM)- blue; fusion peptide (FP)- red; fusion-peptide proximal region (FPPR)- green; heptad repeat 1 (HR1)- orange; central helix (CH)- grey; connector domain (CD)- purple. LA is shown with magenta spheres. These structures show the ectodomain of the spike trimer in the locked conformation.

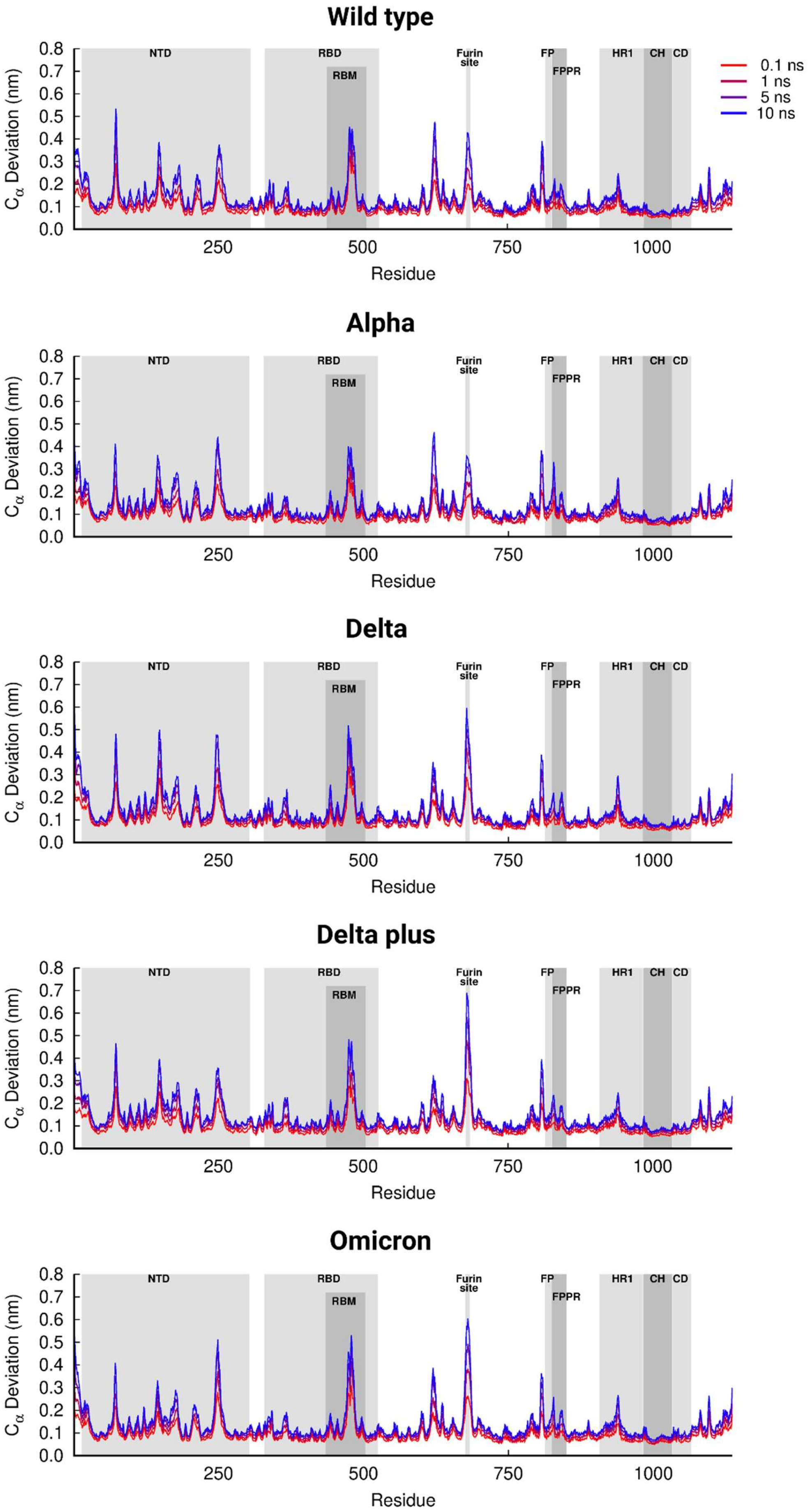
Wild type
Alpha
Delta
Delta plus
Omicron
Cα Deviation (nm)
Residue
NTD
RBD
RBM
Furin site
FP
FPPR
HR1
CH
CD
0.1 ns
1 ns
5 ns
10 ns
0.0
0.1
0.2
0.3
0.4
0.5
0.6
0.7
0.8
250
500
750
1000

Supplementary Figure 15. Average Cα-positional deviations in the 10 ns after LA removal from the FA sites in the wild-type, Alpha, Delta, Delta Plus and Omicron spikes. The average deviations were determined using the Kubo-Onsager approach (Ciccotti, 1991; Ciccotti and Ferrario, 2016; Ciccotti et al., 1979; Oliveira et al., 2021) for the pairwise comparison between the D-NEMD apo and equilibrium LA-bound simulations. The averages were calculated over the three chains of the trimer and over 87 pairs of simulations. The positions of some important structural motifs are highlighted in grey, namely the N-terminal domain (NTD), receptor-binding domain (RBD), receptor-binding motif (RBM), fusion peptide (FP), fusion-peptide proximal region (FPPR), heptad repeat 1 (HR1), central helix (CH) and connector domain (CD). Please zoom in to the image for detailed visualisation.

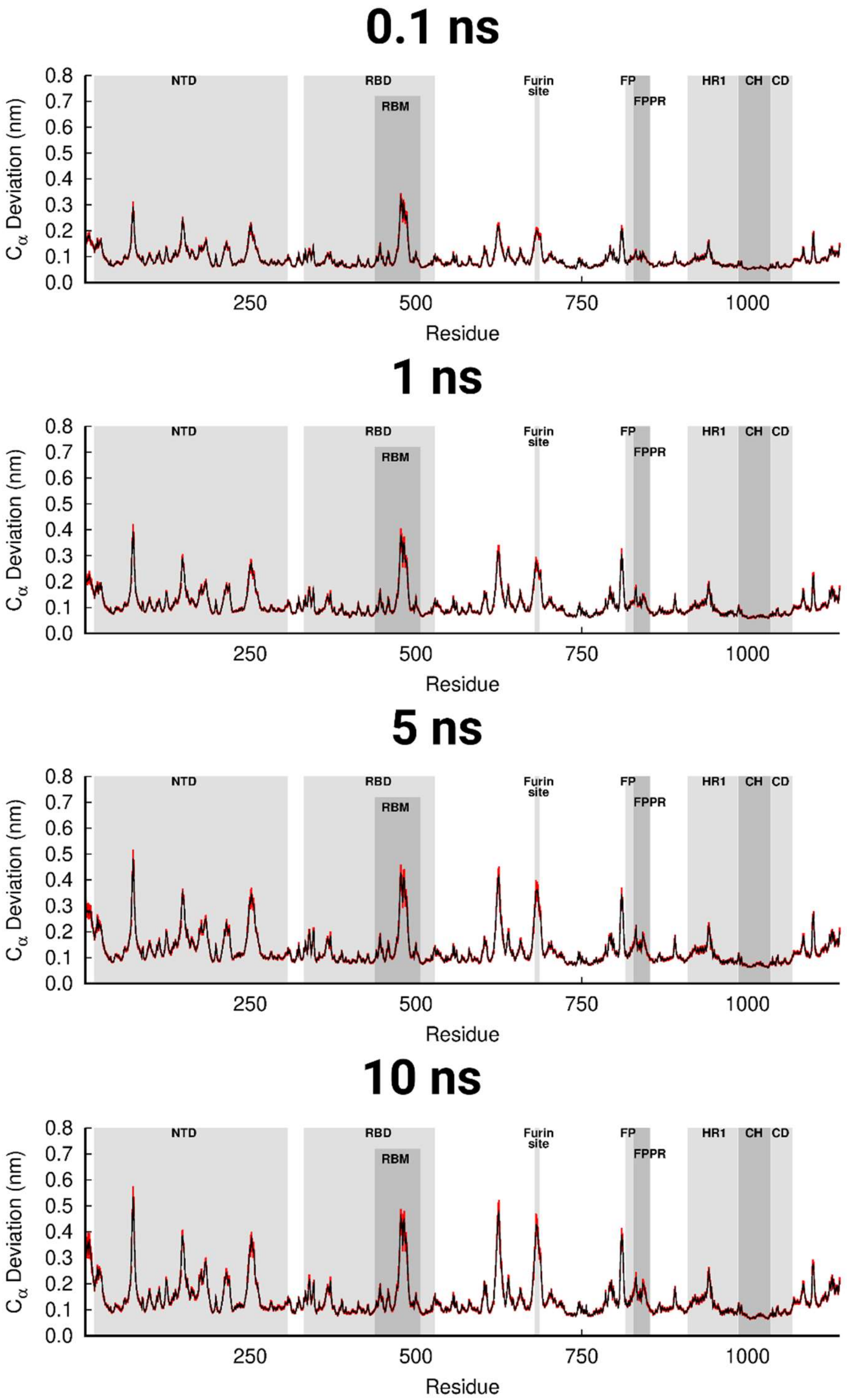

Supplementary Figure 16. Wild-type spike: average Cα-positional deviations and corresponding standard errors in the 0.1, 1, 5 and 10 ns after LA removal from the FA sites. The average deviations were determined using the Kubo-Onsager approach (Ciccotti, 1991; Ciccotti and Ferrario, 2016; Ciccotti et al., 1979; Oliveira et al., 2021) for the pairwise comparison between the nonequilibrium apo and equilibrium LA-bound simulations. The averages were calculated over the three chains of the trimer and over 87 pairs of simulations. The vertical red lines represent the standard error of the mean. The positions of some important structural motifs are highlighted in grey, namely the N-terminal domain (NTD), receptor-binding domain (RBD), receptor-binding motif (RBM), fusion peptide (FP), fusion-peptide

proximal region (FPPR), heptad repeat 1 (HR1), central helix (CH), connector domain (CD). Please zoom in to the image for detailed visualisation.

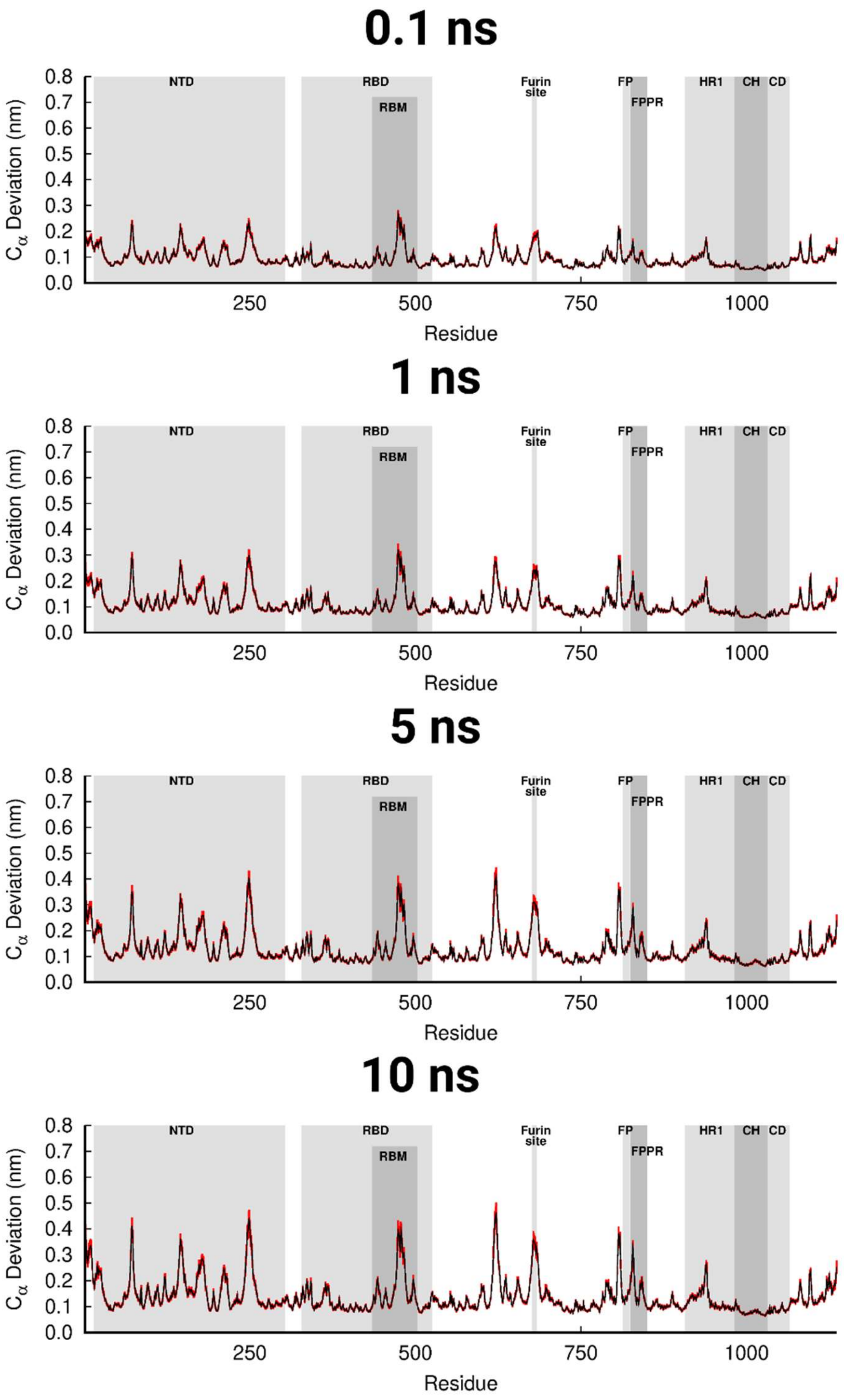

Supplementary Figure 17. Alpha spike: average Cα-positional deviations and corresponding standard errors in the 0.1, 1, 5 and 10 ns after LA removal from the FA sites. For more details, see the legend in Supplementary Figure 16. Please zoom in to the image for detailed visualisation.

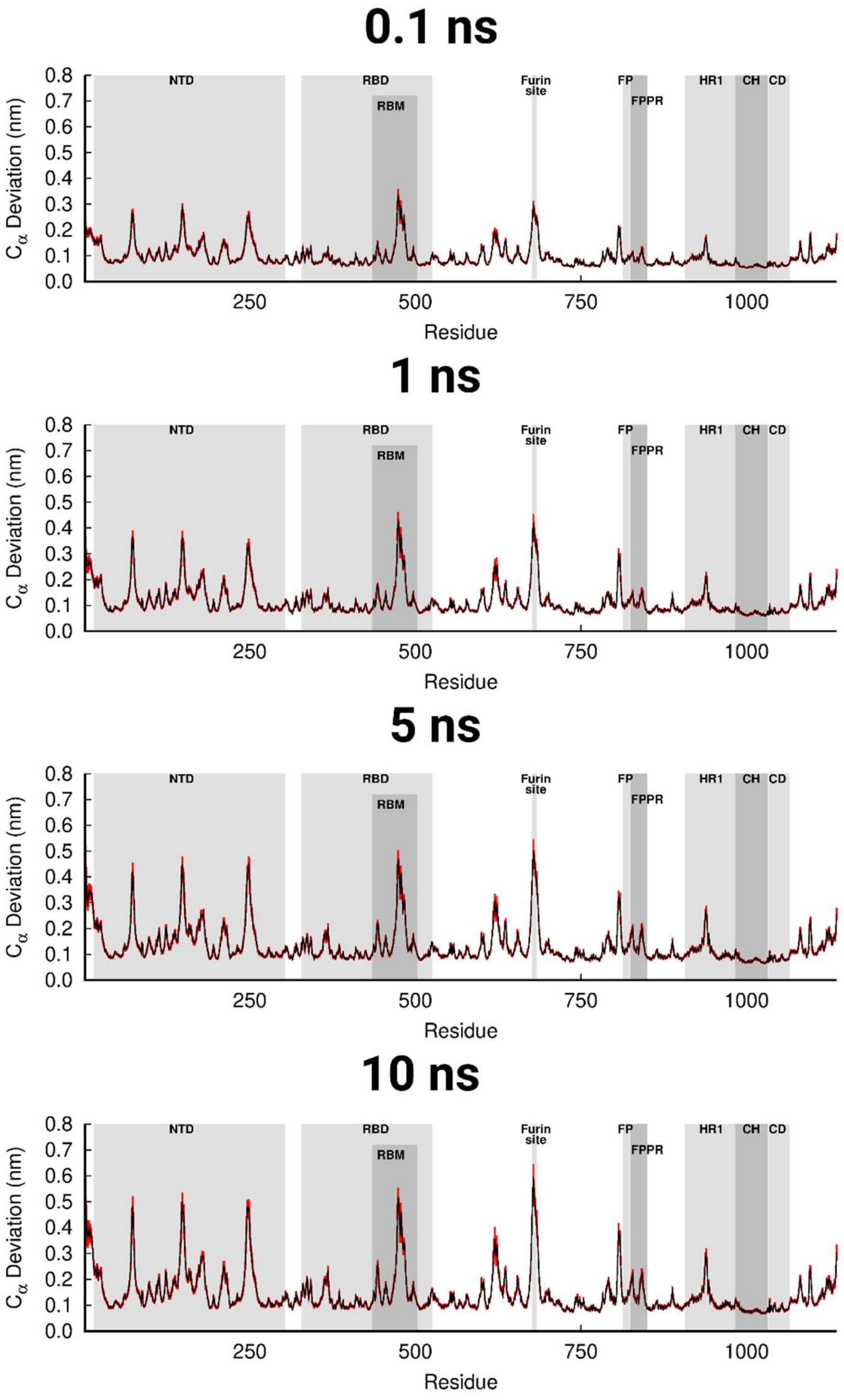

Supplementary Figure 18. Delta spike: average Cα-positional deviations and corresponding standard errors in the 0.1, 1, 5 and 10 ns after LA removal from the FA sites. For more details, see the legend in Supplementary Figure 16. Please zoom in to the image for detailed visualisation.

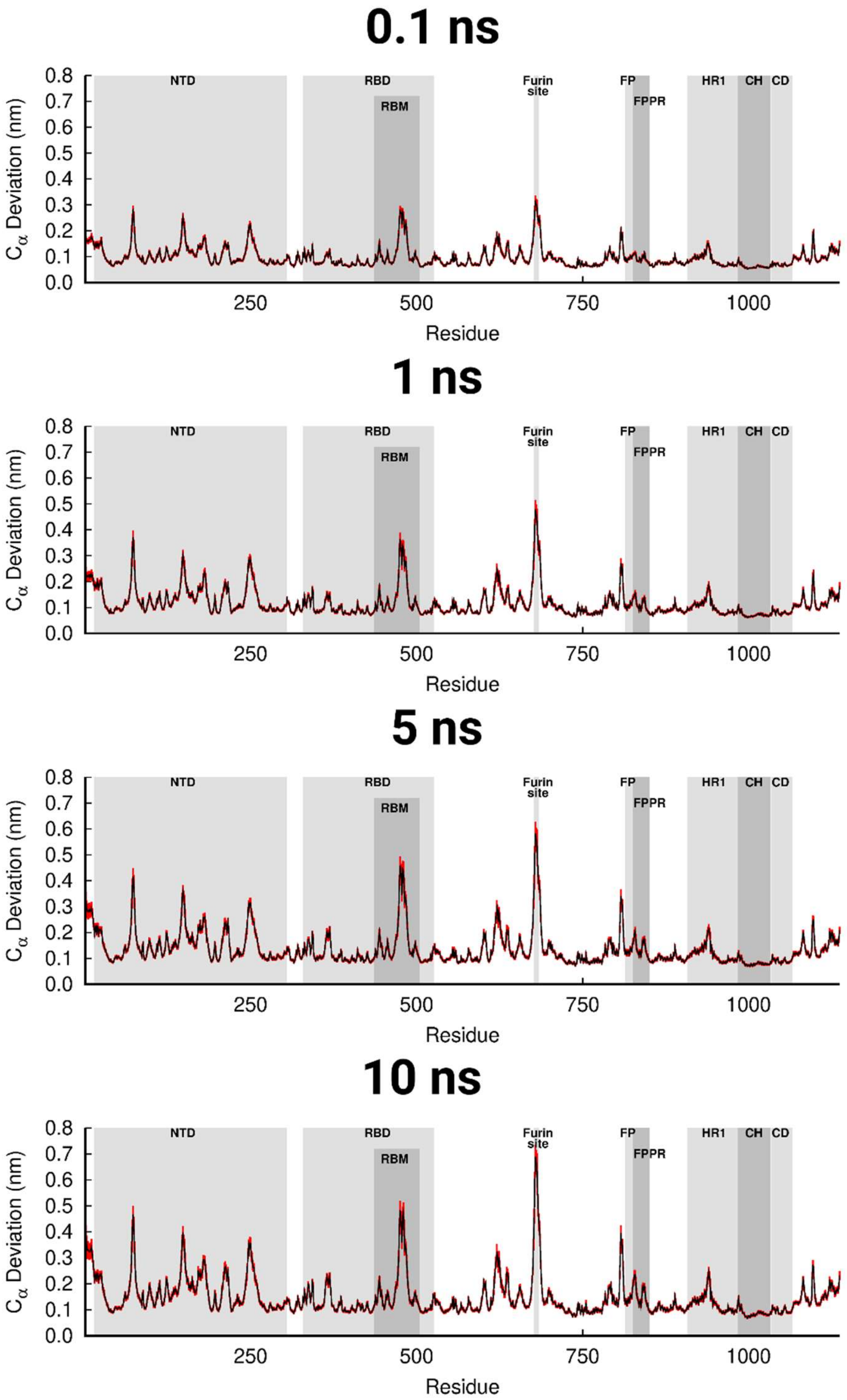

Supplementary Figure 19. Delta plus spike: average Cα-positional deviations (and corresponding standard errors) in the 0.1, 1, 5 and 10 ns after LA removal from the FA sites. For more details, see the legend in Supplementary Figure 16. Please zoom in to the image for detailed visualisation.

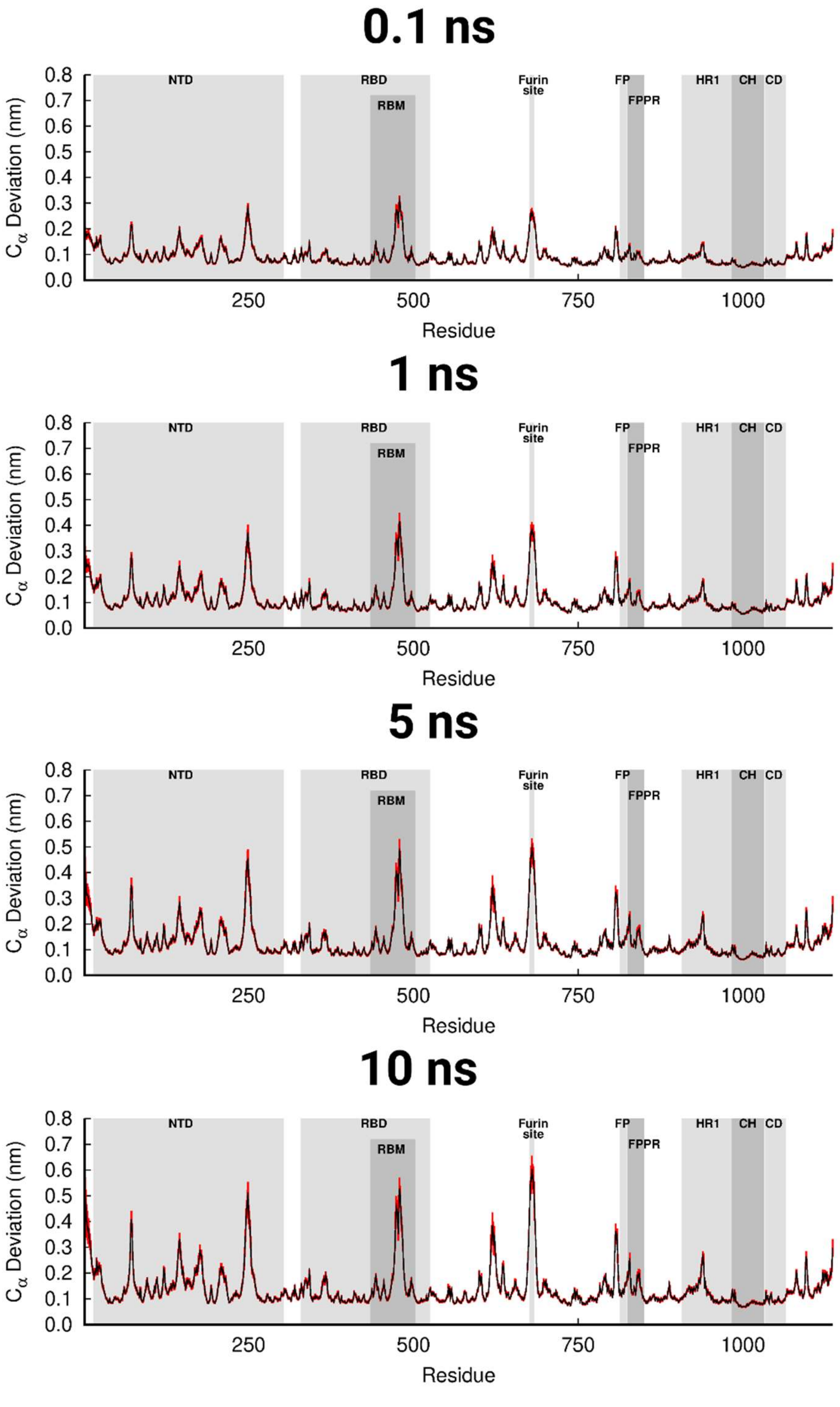

Supplementary Figure 20. Omicron spike: average Cα-positional deviations (and corresponding standard errors) in the 0.1, 1, 5 and 10 ns after LA removal from the FA sites. For more details, see the legend in Supplementary Figure 16. Please zoom in to the image for detailed visualisation.

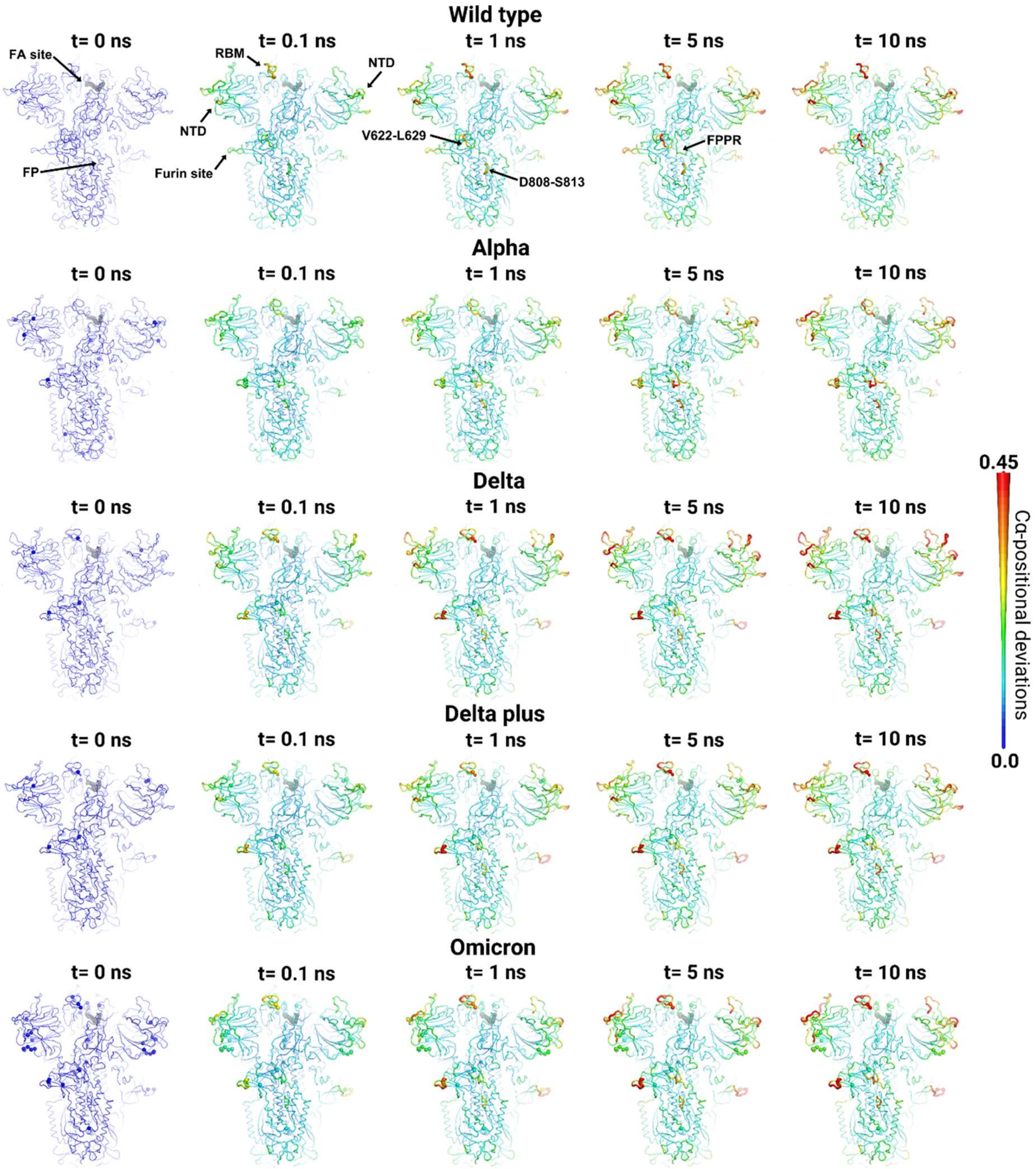

Supplementary Figure 21. Average Cα-positional deviations in the ten nanoseconds following LA removal in the wild-type, Alpha, Delta, Delta Plus and Omicron spike. The Cα deviations between the D-NEMD apo and equilibrium LA-bound simulations at specific times (0, 0.1, 1, 5 and 10 ns) after LA removal were calculated as a function of the residue number. The final deviation values correspond to the average obtained over the three chains of the trimer and over all 87 pairs of simulations (Supplementary Figure 15). The Cα average deviations are mapped onto the starting structure for the LA-bound equilibrium simulations of each variant. Structure colours and cartoon thickness relate to the average Cα-positional deviation values. The dark grey spheres highlight the location of the FA binding site, and other spheres show mutations. Please zoom in to the picture for detailed visualisation.

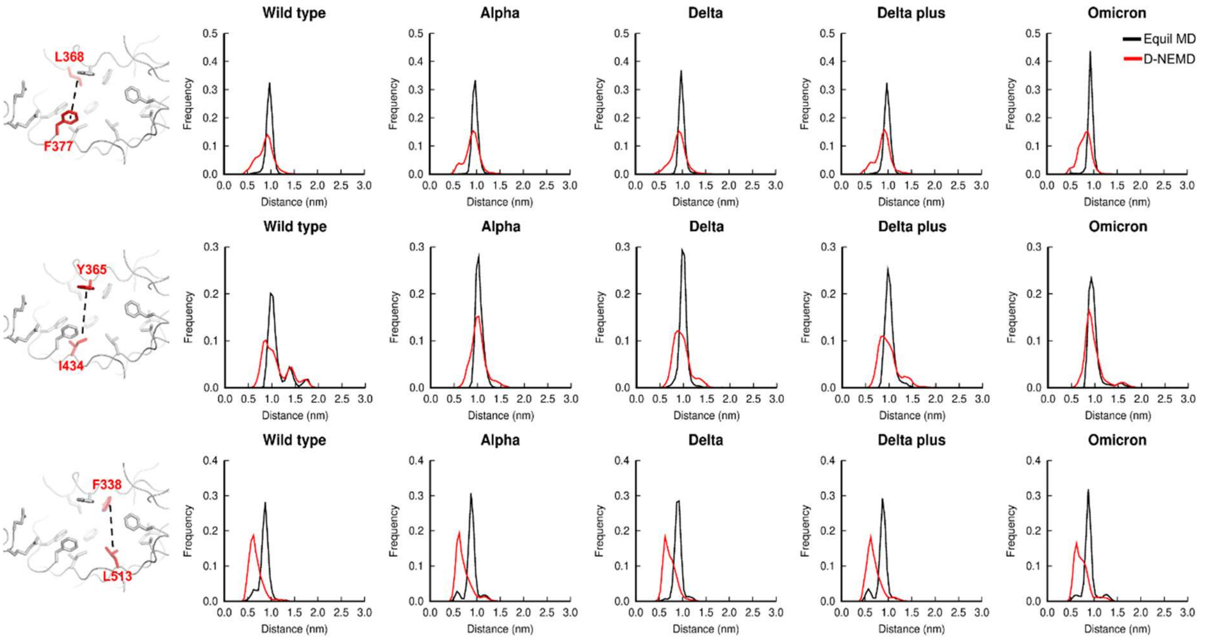

Supplementary Figure 22. Distributions of the distances between L368-F377, Y365-I434 and F338-L513 in the equilibrium FA-bound and D-NEMD apo wild type, Alpha, Delta, Delta Plus and Omicron variant. Overall distribution of the distance between the centre of mass of the sidechains of L368 and F377, Y365 and I434 and F338 and L513 in the equilibrium (black line) and D-NEMD (red line) simulations. Note that the images of the FA site (shown on the left) represent the same orientation as Figure 1B. Please zoom in to the image for detailed visualisation.

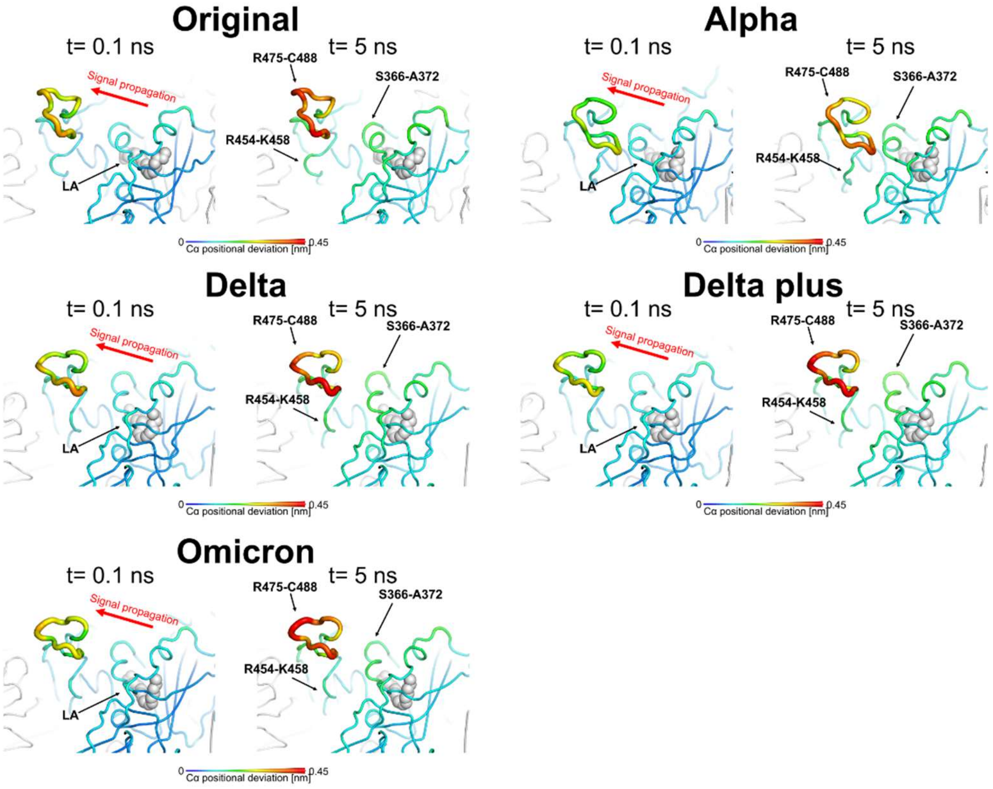

Supplementary Figure 23. Allosteric pathways connecting the FA site to the RBD in the wild type, Alpha, Delta, Delta plus and Omicron spikes. Average Cα-positional deviation at times 0.1 and 5 ns following LA removal from the FA binding pockets. For more details, see the legend in Supplementary Figure 21. Please zoom in to the picture for detailed visualisation.

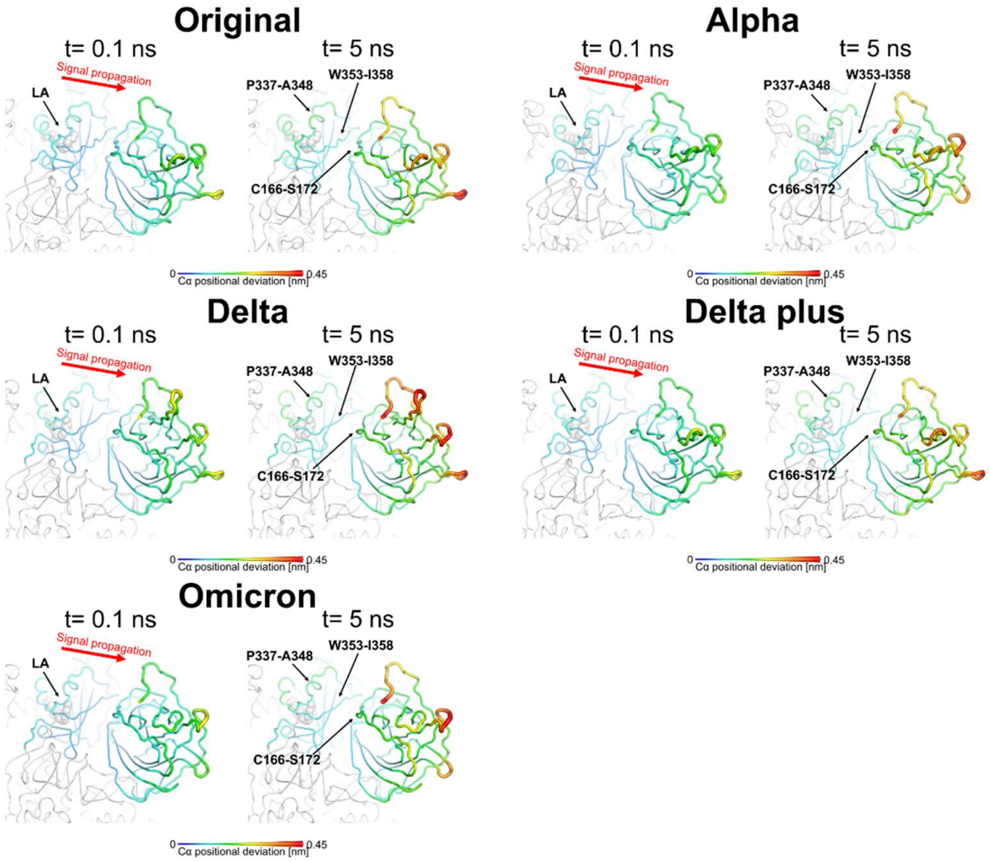

Supplementary Figure 24. Allosteric pathways connecting the FA site to the NTD in the wild type, Alpha, Delta, Delta plus and Omicron. Average Cα-positional deviation at times 0.1 and 5 ns following LA removal from the FA binding pockets. For more details, see the legend in Supplementary Figure 21. Please zoom in to the picture for detailed visualisation.

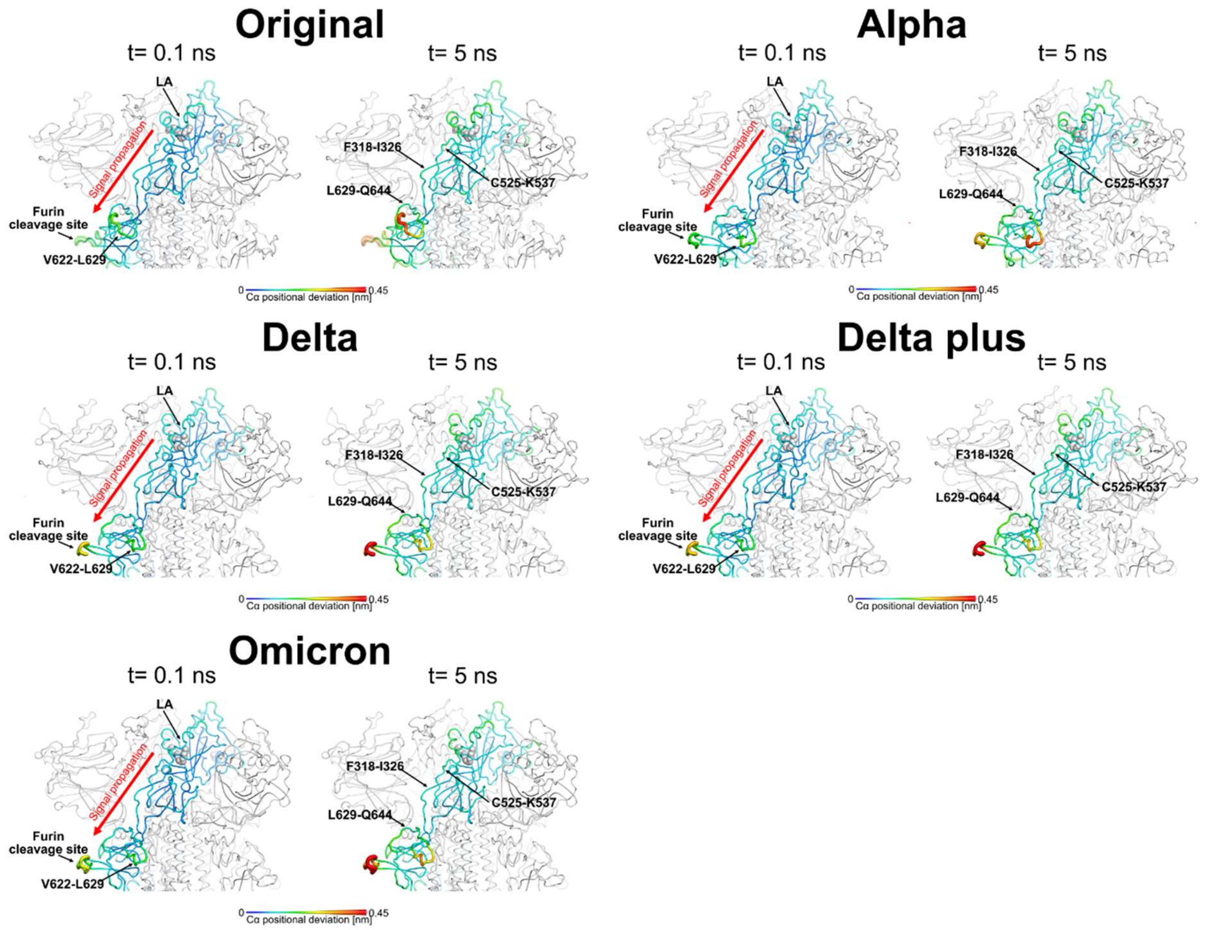

Supplementary Figure 25. Allosteric pathways connecting the FA site to the furin cleavage region in the wild type, Alpha, Delta, Delta plus and Omicron. Average Cα-positional deviation at times 0.1 and 5 ns following LA removal from the FA binding pockets. For more details, see the legend in Supplementary Figure 21. Please zoom in to the picture for detailed visualisation.

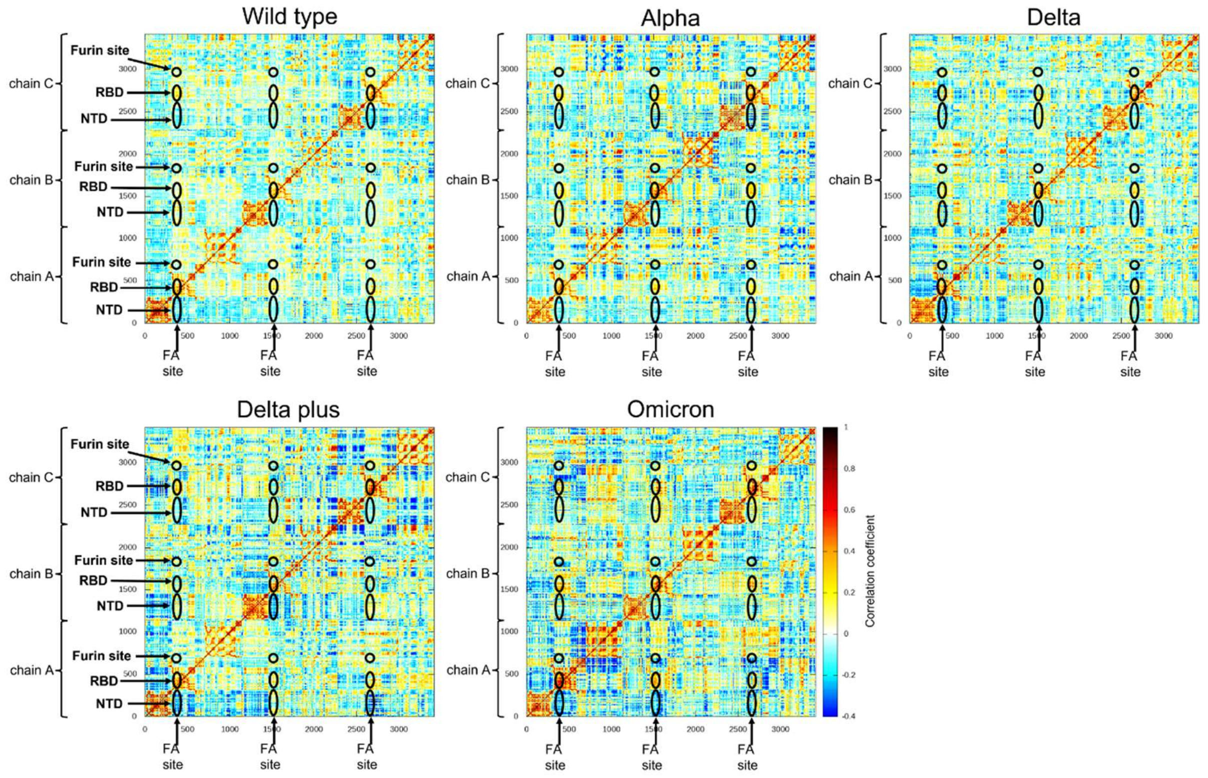

Supplementary Figure 26. Cross-correlation maps computed for the LA-free D-NEMD trajectories. The correlations were calculated for all Cα atoms over the 87 D-NEMD simulations performed for each system. Atoms that systematically move in the same direction have a correlation value higher than zero while those moving in opposite directions have a correlation value lower than 0. White regions indicate no correlation. Yellow, orange and red colours indicate low, moderate and significant positive correlations, while cyan and dark blue represent moderate and significant negative correlations. Please zoom in to the image for detailed visualisation.

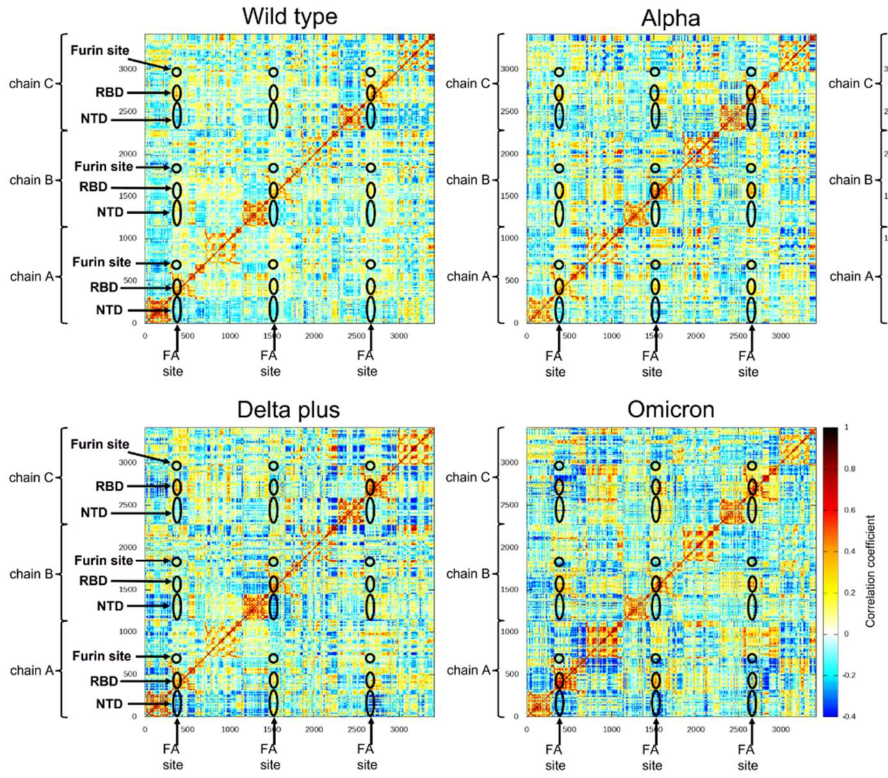

Supplementary Figure 27. Cross-correlation maps computed for LA-bound equilibrium trajectories. The correlations were calculated for all Cα atoms over the equilibrated part of the trajectories (50-200 ns) and over the 3 replica simulations for each system. Atoms that systematically move in the same direction have a correlation value higher than zero while those moving in opposite directions have a correlation value lower than 0. White regions indicate no correlation. Yellow, orange and red colours indicate low, moderate and significant positive correlations while cyan and dark blue represent moderate and significant negative correlations. Please zoom in to the image for detailed visualisation.

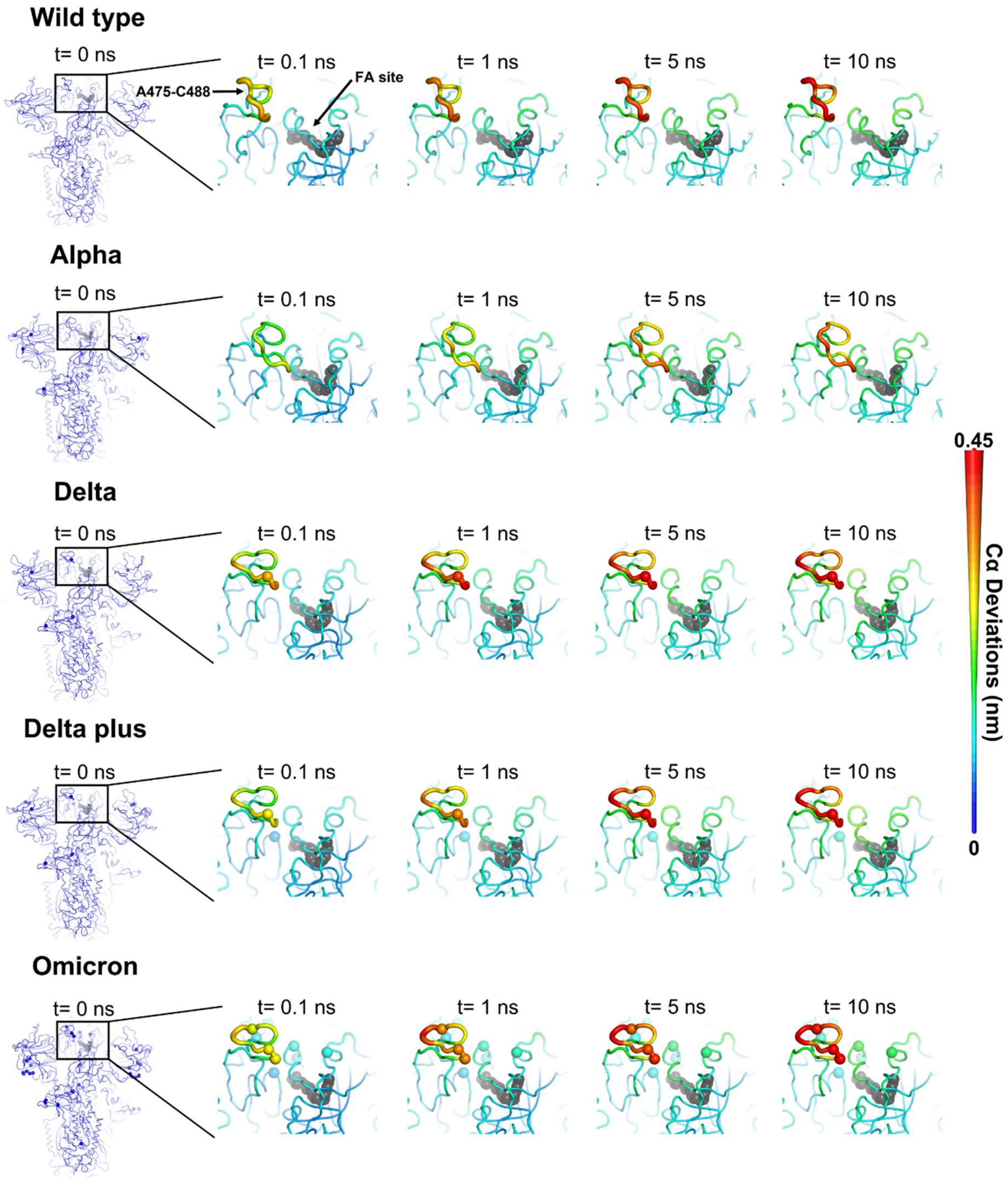

Supplementary Figure 28. Time evolution of the structural response of the RBD. Cα average deviations 0, 0.1, 1, 5 and 10 after LA removal are mapped onto the starting structure for equilibrium simulations of each variant. The structure colours indicate the average Cα-positional deviation values (indicated in the scale on the right). The dark grey spheres highlight the FA binding site and other spheres show positions of mutations. Please zoom in to the picture for detailed visualisation.

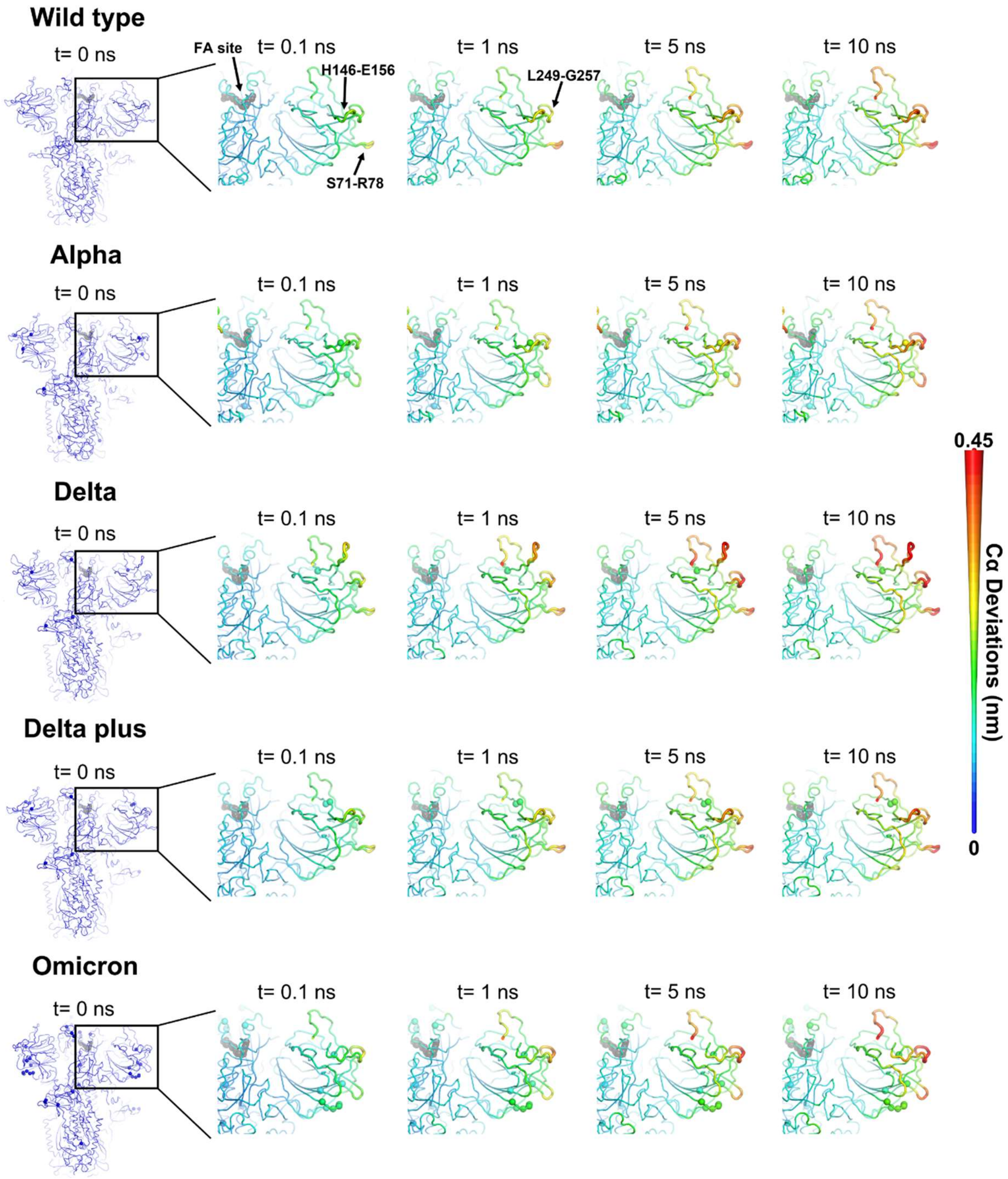

Supplementary Figure 29. Time evolution of the structural response of the NTD. The Cα average deviations 0, 0.1, 1, 5 and 10 after LA removal are mapped onto the starting structures for equilibrium simulations of each variant. For more details, see Supplementary Figure 28 legend. Please zoom in to the picture for detailed visualisation.

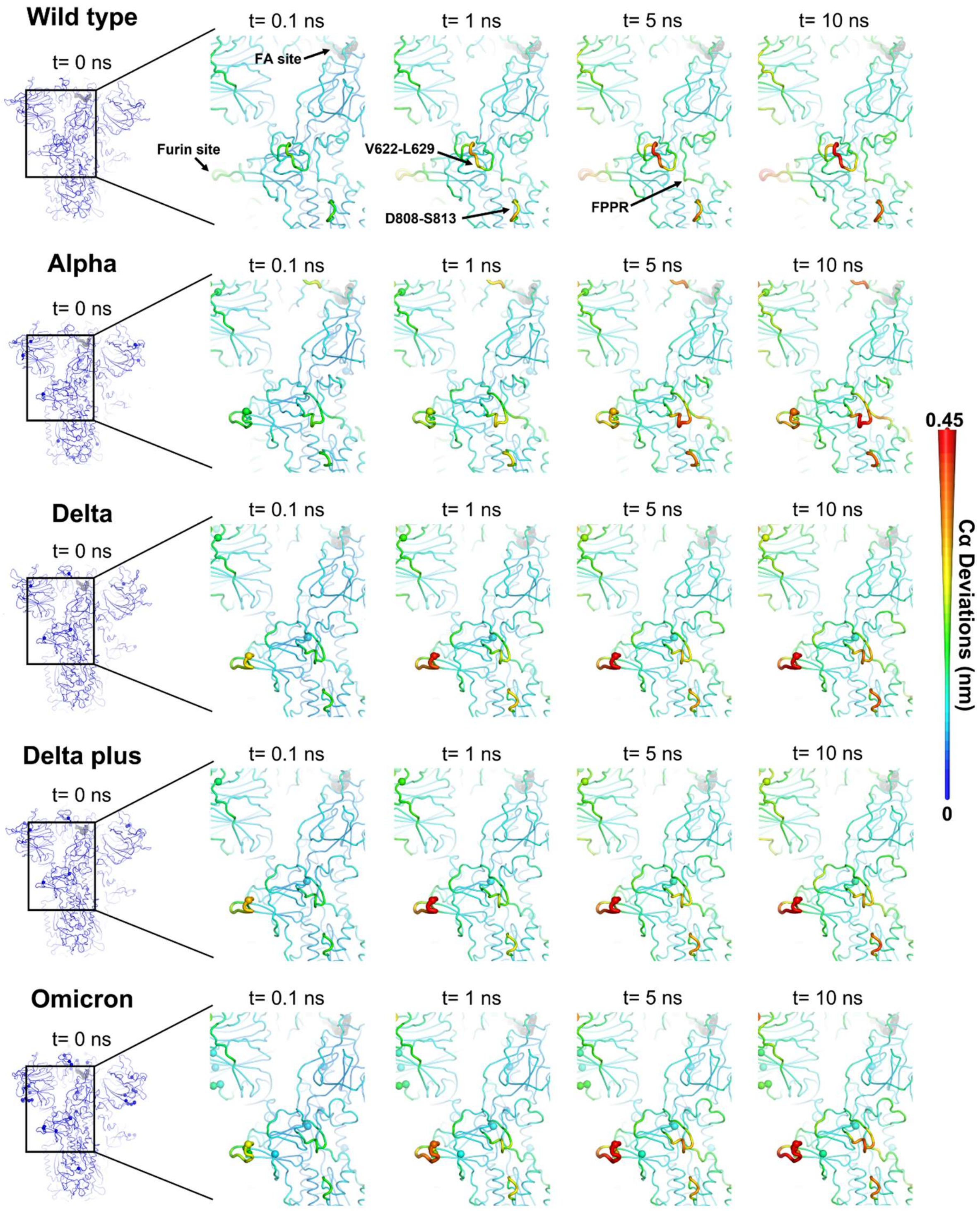

Supplementary Figure 30. Time evolution of the structural response of the furin cleavage site and FP-surrounding regions. The Cα average deviations 0, 0.1, 1, 5 and 10 after LA removal are mapped onto the starting structures for equilibrium simulations of each variant. For more details, see Supplementary Figure 28 legend. Please zoom in to the picture for detailed visualisation.

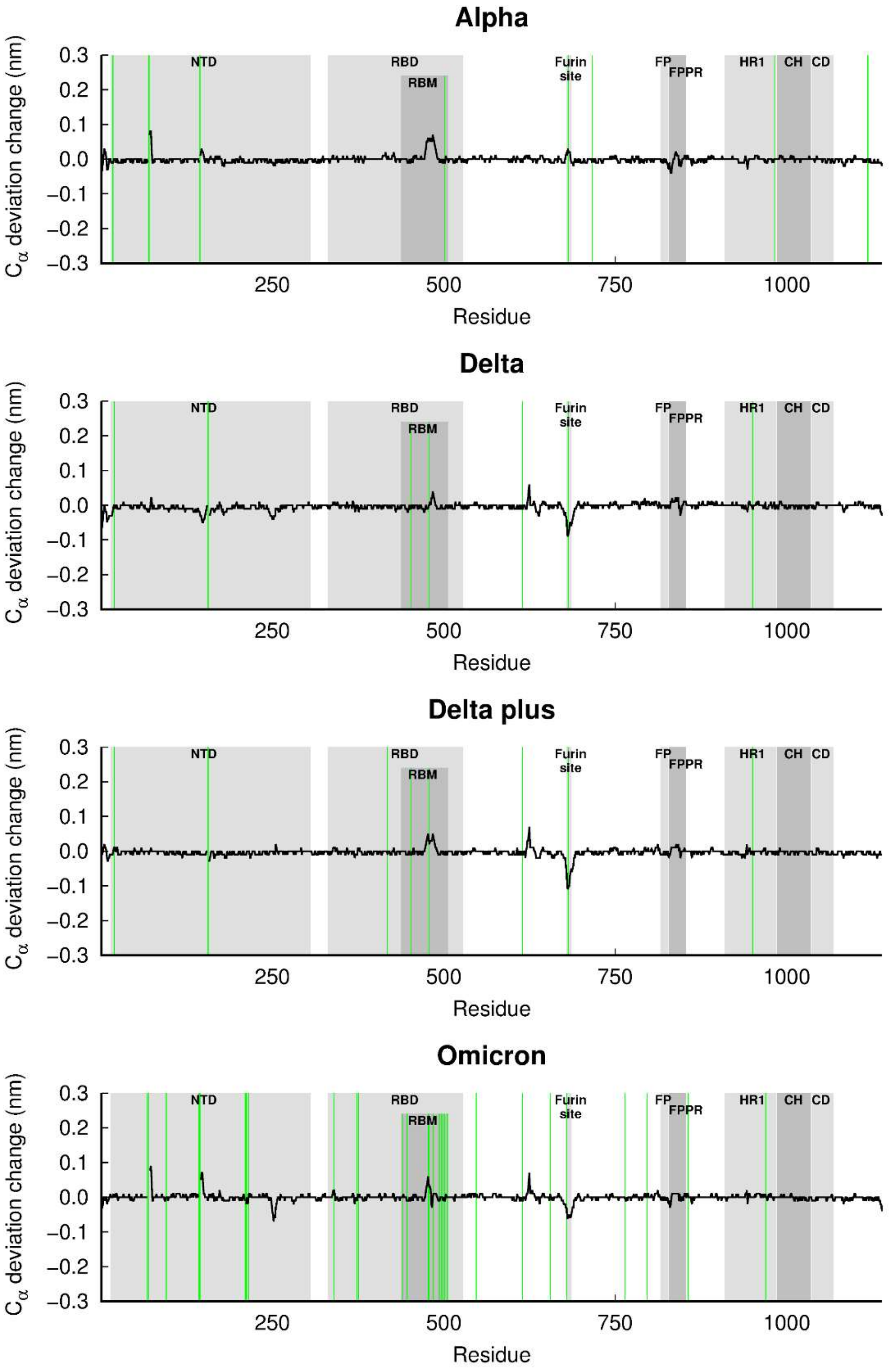

Supplementary Figure 31. Difference in the structural response between the wild type and Alpha, Delta, Delta plus and Omicron variants in t=0.1 ns after LA removal from the FA sites. The positions of some important structural motifs are highlighted in grey, namely the N-terminal domain (NTD), receptor-binding domain (RBD), receptor-binding motif (RBM), fusion peptide (FP), fusion-peptide proximal region (FPPR), heptad repeat 1 (HR1), central

helix (CH) and connector domain (CD). The green vertical lines pinpoint the location of the mutations occurring in each variant. Please zoom in to the image for detailed visualisation.

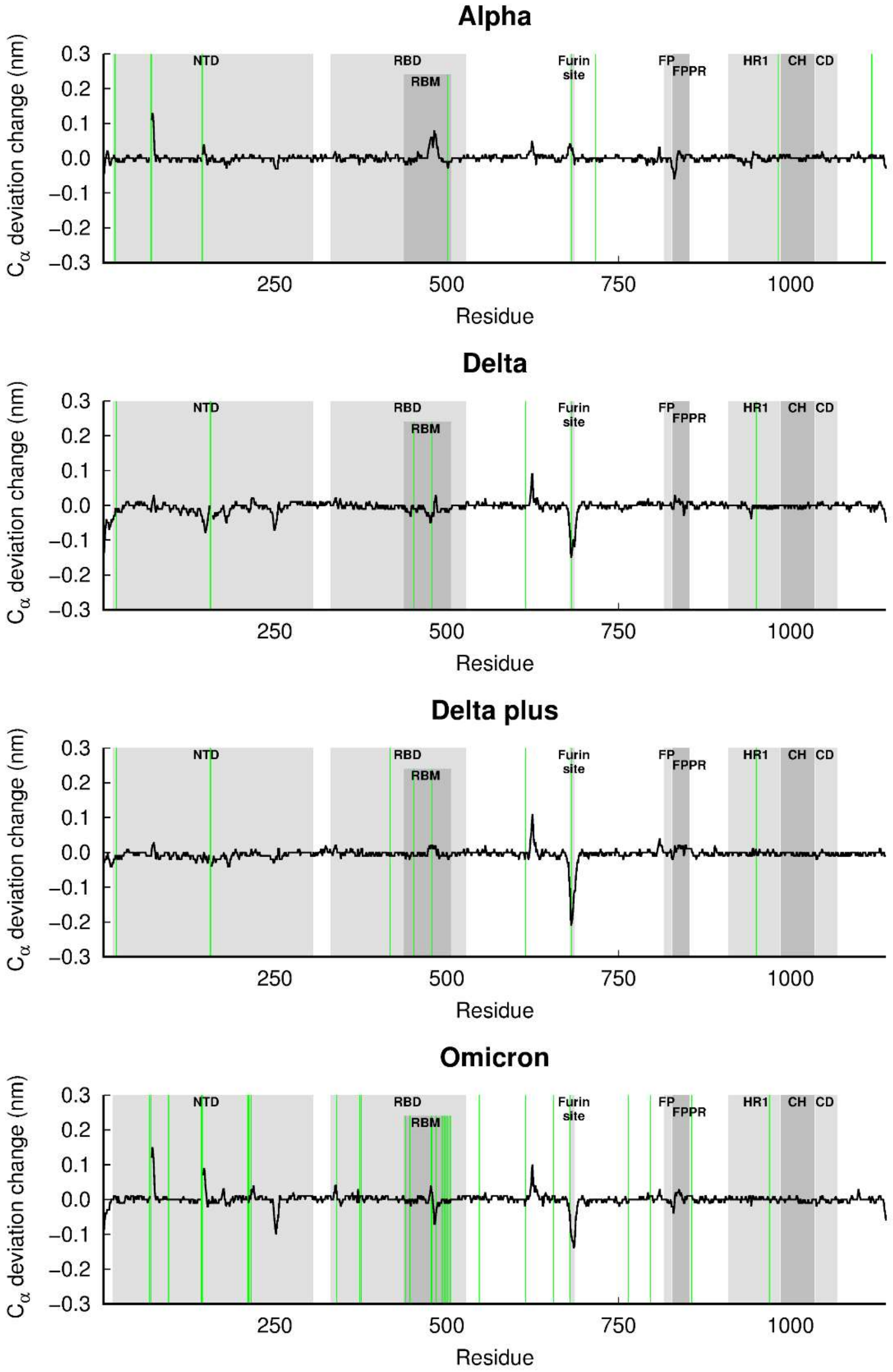

Supplementary Figure 32. Difference in the structural response between the wild type and Alpha, Delta, Delta plus and Omicron variants in the t=1 ns after LA removal from the FA sites. For more details, see Supplementary Figure 31 legend. Please zoom in to the image for detailed visualisation.

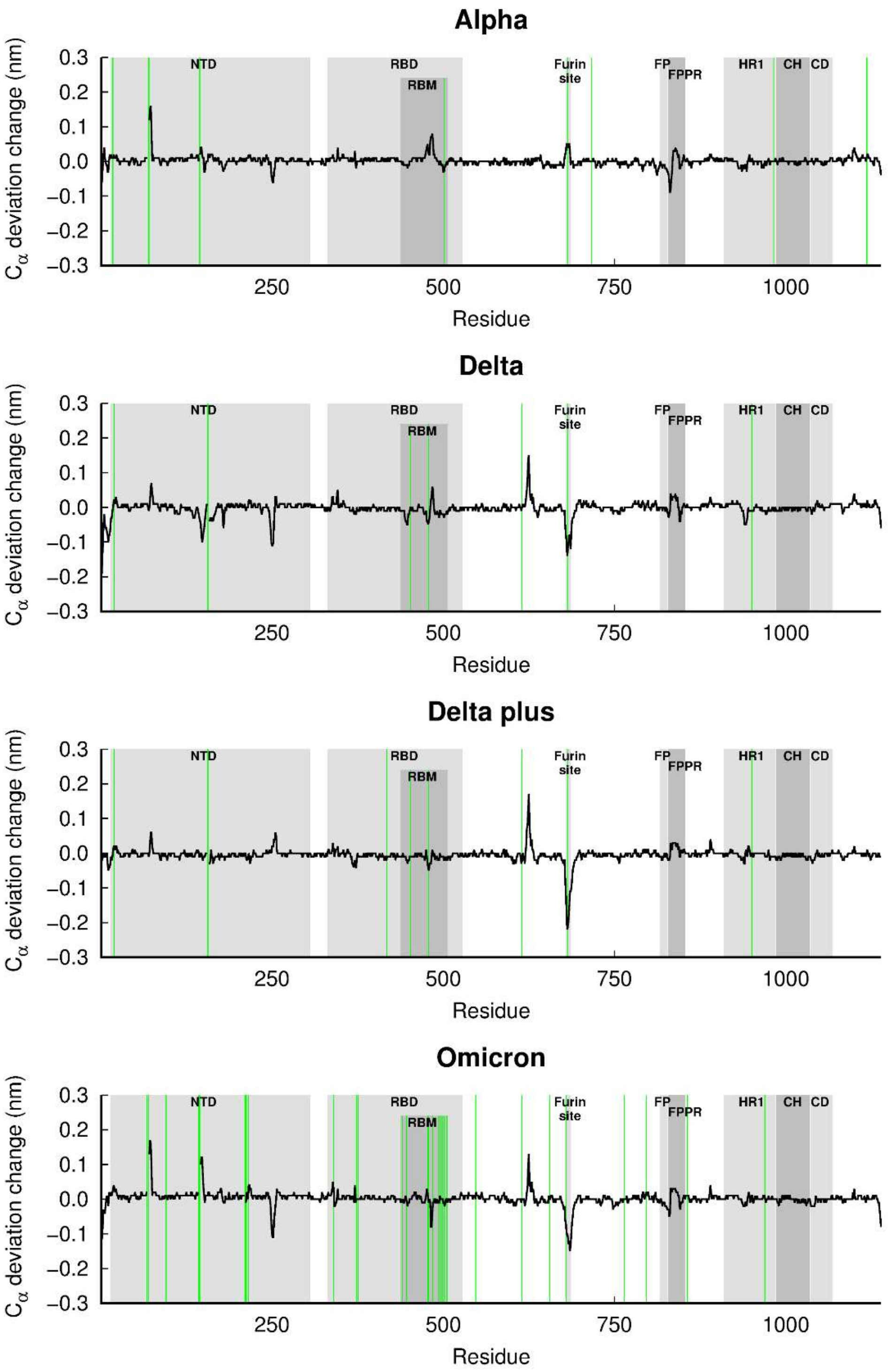

Supplementary Figure 33. Difference in the structural response between the wild type and Alpha, Delta, Delta plus and Omicron variants in the t=5 ns after LA removal from the FA sites. For more details, see Supplementary Figure 31 legend. Please zoom in to the image for detailed visualisation.

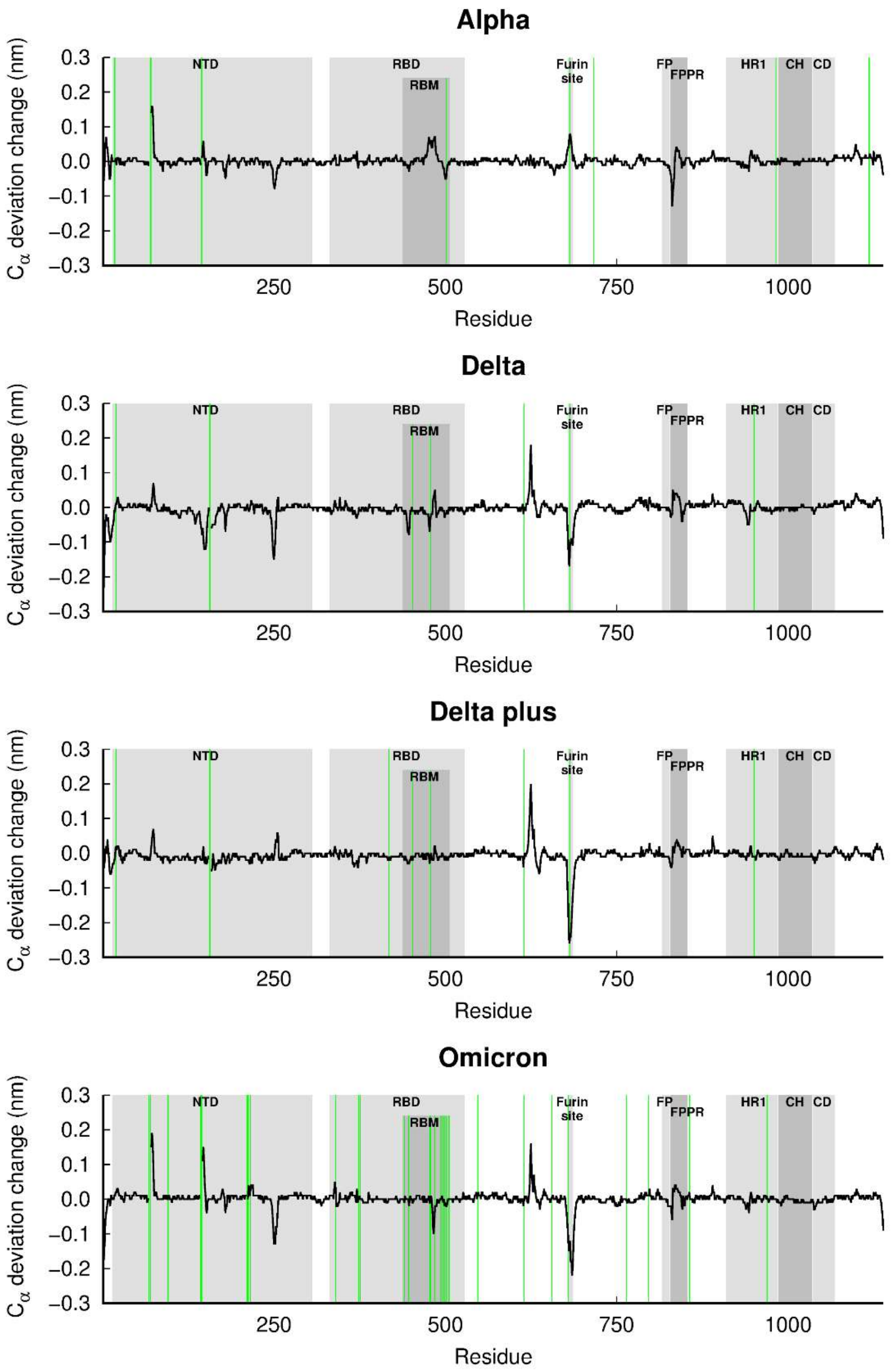

Supplementary Figure 34. Difference in the structural response between the wild type and Alpha, Delta, Delta plus and Omicron variants in the t=10 ns after LA removal from the FA sites. For more details, see Supplementary Figure 31 legend. Please zoom in to the image for detailed visualisation.

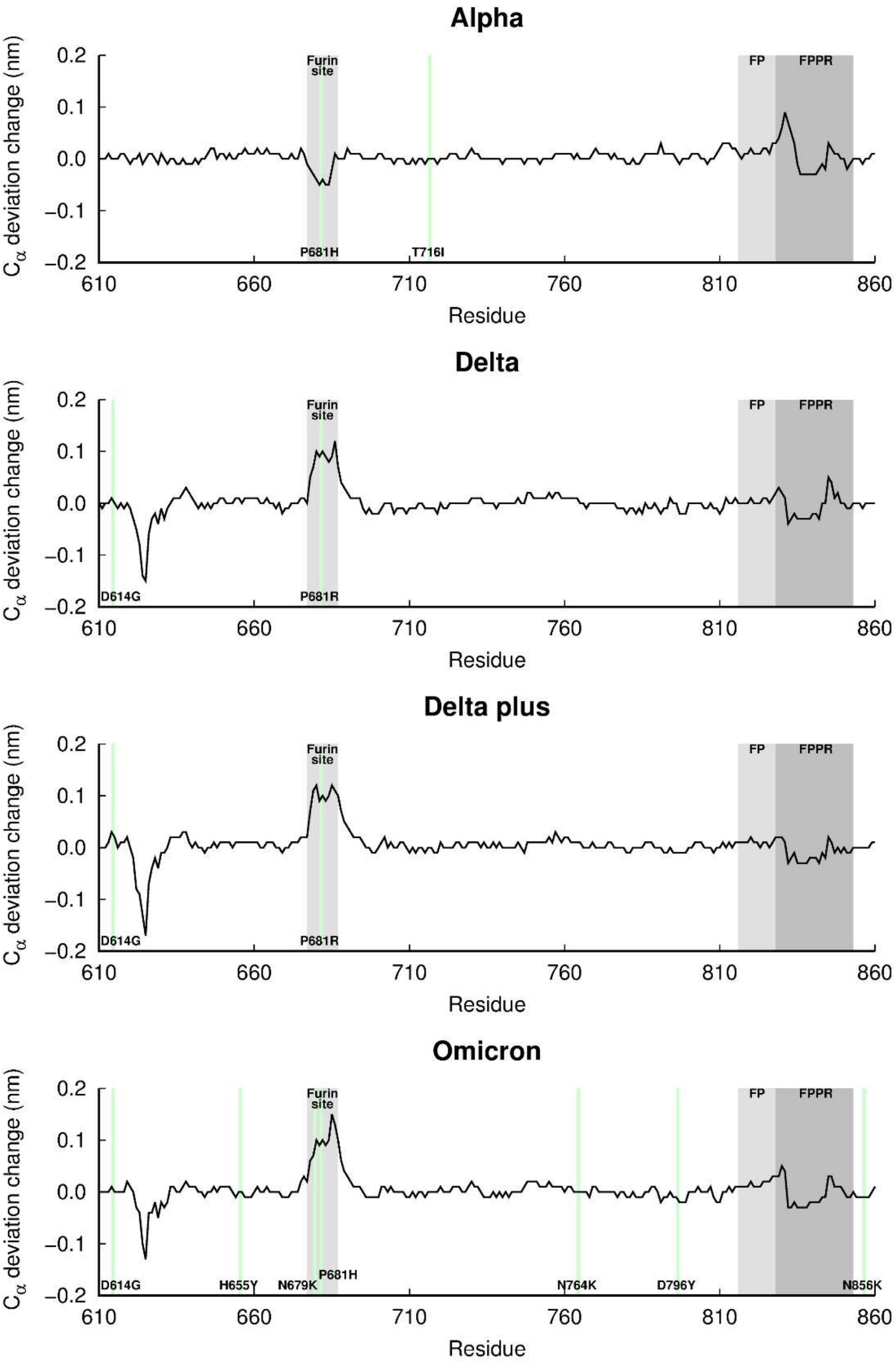

Supplementary Figure 35. Difference in the structural response of the furin cleavage site and FP-surrounding regions between the wild type and Alpha, Delta, Delta plus and Omicron variants in the t=10 ns after LA removal from the FA sites. The positions of the furin cleavage site (located at the S1/S2 interface), fusion peptide (FP) and fusion-peptide proximal region (FPPR) are highlighted in grey. The green vertical lines show the locations of mutations in each variant.

References

Abraham, M.J., Murtola, T., Schulz, R., et al. (2015). GROMACS: High performance molecular simulations through multi-level parallelism from laptops to supercomputers. SoftwareX 1-2, 19-25.

Abreu, B., Lopes, E.F., Oliveira, A.S.F., et al. (2020). F508del disturbs the dynamics of the nucleotide binding domains of CFTR before and after ATP hydrolysis. Proteins 88, 113-126.

Bangaru, S., Ozorowski, G., Turner, H.L., et al. (2020). Structural analysis of full-length SARS-CoV-2 spike protein from an advanced vaccine candidate. Science 370, 1089-1094.

Ciccotti, G., (1991). Molecular dynamics simulation of non equilibrium phenomena and rare dynamical events, in: Meyer M, P.V. (Ed.), Computer simulation in material science. Kluwer Academic Publishers, pp. 119–137.

Ciccotti, G., Ferrario, M., (2016). Non-equilibrium by molecular dynamics: a dynamical approach. Mol Simul 42, 1385-1400.

Ciccotti, G., Jacucci, G., McDonald, I.R., (1979). Thought-experiments by molecular dynamics. J Stat Phys 21, 1-21.

Damas, J.M., Oliveira, A.S.F., Baptista, A.M., et al. (2011). Structural consequences of ATP hydrolysis on the ABC transporter NBD dimer: molecular dynamics studies of HlyB. Protein Sci 20, 1220-1230.

Galdadas, I., Qu, S., Oliveira, A.S.F., et al. (2021). Allosteric communication in class A β-lactamases occurs via cooperative coupling of loop dynamics. eLife 10, e66567.

Garton, M., Laughton, C., (2013). A comprehensive model for the recognition of human telomeres by TRF1. J Mol Biol 425, 2910-2921.

Gobeil, S.M., Henderson, R., Stalls, V., et al. (2022). Structural diversity of the SARS-CoV-2 Omicron spike. Mol Cell 82, 2050-2068.e2056.

Gupta, K., Toelzer, C., Williamson, M.K., et al. (2022). Structural insights in cell-type specific evolution of intra-host diversity by SARS-CoV-2. Nat Commun 13, 222.

Kabsch, W., Sander, C., (1983). Dictionary of protein secondary structure: pattern recognition of hydrogen-bonded and geometrical features. Biopolymers 22, 2577-2637.

Ng, H.W., Laughton, C.A., Doughty, S.W., (2013). Molecular dynamics simulations of the adenosine A2a receptor: structural stability, sampling, and convergence. J Chem Inf Model 53, 1168-1178.

Oliveira, A.S.F., Ciccotti, G., Haider, S., et al. (2021). Dynamical nonequilibrium molecular dynamics reveals the structural basis for allostery and signal propagation in biomolecular systems. Eur Phys J B 94, 144.

Oliveira, A.S.F., Edsall, C., Woods, C., et al. (2019a). A general mechanism for signal propagation in the nicotinic acetylcholine receptor family. J Am Chem Soc 141, 19953–19958.

Oliveira, A.S.F., Shoemark, D.K., Avila Ibarra, A., et al. (2022). The fatty acid site is coupled to functional motifs in the SARS-CoV-2 spike protein and modulates spike allosteric behaviour. Comput Struct Biotechnol J 20, 139–147.

Oliveira, A.S.F., Shoemark, D.K., Campello, H.R., et al. (2019b). Identification of the initial steps in signal transduction in the α4β2 nicotinic receptor: insights from equilibrium and nonequilibrium simulations. Structure 27, 1171-1183.

Roy, J., Laughton, C.A., (2010). Long-timescale molecular-dynamics simulations of the major urinary protein provide atomistic interpretations of the unusual thermodynamics of ligand binding. Biophys J 99, 218-226.

Shoemark, D.K., Colenso, C.K., Toelzer, C., et al. (2021). Molecular simulations suggest vitamins, retinoids and steroids as ligands of the free fatty acid pocket of the SARS-CoV-2 spike protein. Angew Chem Int Ed Engl 60, 7098-7110.

Shoemark, D.K., Oliveira, A.S.F., Davidson, A.D., et al. (2022). Molecular dynamics of spike variants in the closed conformation; their RBD interfaces, fatty acid binding and furin cleavage sites. bioRxiv 10.1101/2022.05.06.490927.

Stalls, V., Lindenberger, J., Gobeil, S.M., et al. (2022). Cryo-EM structures of SARS-CoV-2 Omicron BA.2 spike. Cell Rep 39, 111009.

Toelzer, C., Gupta, K., Yadav, S., et al. (2020). Free fatty acid binding pocket in the locked structure of SARS-CoV-2 spike protein. Science 370, 725-730.

Zhang, J., Cai, Y., Lavine, C.L., et al. (2022). Structural and functional impact by SARS-CoV-2 Omicron spike mutations. Cell Rep 39, 110729.
