# Supplementary material for: SARS-CoV-2 spike variants differ in their allosteric responses to linoleic acid
Source: J Mol Cell Biol. 2023 Mar 29;15(3):mjad021. doi: 10.1093/jmcb/mjad021 (PMC10563148; doi:10.1093/jmcb/mjad021)
Supplement: mjad021_Supplemental_Files [file mjad021_supplemental_files.zip › Supplementary_tables.pdf]

Supplementary Table S1. Average Cα-positional deviations in the 0.1 ns after LA removal

	Deviation after 0.1 ns (nm)				
**Cα atom**	**Wild type**	**Alpha**	**Delta**	**Delta plus**	**Omicron**
1	0.20	0.24	0.27	0.20	0.23
2	0.16	0.19	0.22	0.17	0.19
3	0.16	0.17	0.20	0.16	0.18
4	0.17	0.16	0.19	0.16	0.17
5	0.18	0.15	0.18	0.16	0.18
6	0.17	0.15	0.18	0.16	0.18
7	0.18	0.16	0.20	0.17	0.18
8	0.17	0.17	0.19	0.17	0.17
9	0.15	0.18	0.20	0.17	0.17
10	0.15	0.18	0.19	0.18	0.16
11	0.15	0.17	0.19	0.17	0.16
12	0.15	0.15	0.18	0.17	0.15
13	0.14	0.14	0.17	0.15	0.14
14	0.14	0.14	0.17	0.15	0.13
15	0.12	0.13	0.15	0.13	0.12
16	0.13	0.14	0.16	0.14	0.13
17	0.14	0.15	0.16	0.15	0.14
18	0.14	0.15	0.15	0.14	0.13
19	0.16	0.16	0.16	0.15	0.16
20	0.16	0.16	0.16	0.15	0.15
21	0.14	0.14	0.15	0.14	0.14
22	0.15	0.16	0.15	0.15	0.14
23	0.16	0.16	0.16	0.15	0.15
24	0.16	0.16	0.17	0.15	0.16
25	0.16	0.17	0.17	0.17	0.17
26	0.14	0.15	0.15	0.15	0.14
27	0.12	0.13	0.13	0.13	0.12
28	0.11	0.12	0.12	0.12	0.11
29	0.10	0.11	0.10	0.11	0.10
30	0.10	0.10	0.10	0.10	0.10
31	0.09	0.10	0.09	0.09	0.09
32	0.09	0.10	0.09	0.09	0.09
33	0.09	0.09	0.08	0.09	0.08
34	0.08	0.09	0.08	0.08	0.08
35	0.08	0.08	0.08	0.08	0.08
36	0.07	0.08	0.07	0.08	0.07
37	0.07	0.07	0.07	0.07	0.07
38	0.07	0.07	0.07	0.07	0.07
39	0.08	0.08	0.08	0.08	0.08
40	0.07	0.07	0.07	0.07	0.07
41	0.07	0.07	0.07	0.07	0.06
42	0.07	0.07	0.07	0.07	0.07
43	0.06	0.07	0.07	0.07	0.07
44	0.07	0.07	0.07	0.07	0.07
45	0.07	0.07	0.08	0.07	0.07

46	0.07	0.08	0.08	0.08	0.08
47	0.07	0.08	0.08	0.08	0.08
48	0.08	0.08	0.08	0.08	0.09
49	0.08	0.08	0.08	0.07	0.08
50	0.08	0.08	0.08	0.08	0.08
51	0.08	0.08	0.08	0.08	0.08
52	0.08	0.08	0.08	0.08	0.07
53	0.07	0.08	0.08	0.08	0.07
54	0.07	0.07	0.07	0.07	0.07
55	0.07	0.07	0.07	0.07	0.07
56	0.07	0.08	0.08	0.08	0.07
57	0.08	0.08	0.08	0.08	0.08
58	0.08	0.08	0.08	0.08	0.08
59	0.10	0.10	0.09	0.09	0.09
60	0.10	0.11	0.10	0.10	0.10
61	0.10	0.11	0.10	0.10	0.10
62	0.09	0.10	0.09	0.09	0.09
63	0.09	0.10	0.10	0.10	0.09
64	0.10	0.10	0.10	0.10	0.10
65	0.09	0.10	0.10	0.09	0.09
66	0.10	0.11	0.11	0.10	0.10
67	0.11	0.11	0.12	0.11	0.11
68	0.12	0.13	0.14	0.12	0.12
69	0.14	n.a.	0.15	0.14	n.a.
70	0.16	n.a.	0.18	0.17	n.a.
71	0.21	0.14	0.22	0.21	0.13
72	0.25	0.17	0.26	0.25	0.17
73	0.29	0.21	0.27	0.28	0.20
74	0.26	0.23	0.24	0.26	0.21
75	0.20	0.21	0.21	0.20	0.21
76	0.16	0.17	0.17	0.16	0.17
77	0.14	0.14	0.14	0.14	0.14
78	0.12	0.13	0.13	0.12	0.12
79	0.11	0.11	0.12	0.11	0.11
80	0.10	0.10	0.11	0.10	0.10
81	0.10	0.10	0.11	0.10	0.09
82	0.10	0.10	0.11	0.10	0.10
83	0.09	0.09	0.10	0.10	0.09
84	0.09	0.09	0.09	0.09	0.09
85	0.09	0.09	0.09	0.09	0.09
86	0.08	0.08	0.09	0.08	0.08
87	0.10	0.10	0.10	0.10	0.10
88	0.09	0.10	0.09	0.09	0.09
89	0.08	0.08	0.08	0.08	0.07
90	0.07	0.08	0.07	0.07	0.07
91	0.07	0.07	0.07	0.07	0.07
92	0.07	0.07	0.08	0.07	0.07
93	0.07	0.08	0.08	0.08	0.08
94	0.08	0.09	0.09	0.09	0.09
95	0.09	0.10	0.10	0.10	0.09

96	0.10	0.10	0.11	0.10	0.10
97	0.11	0.12	0.12	0.12	0.11
98	0.11	0.12	0.12	0.12	0.12
99	0.10	0.11	0.12	0.11	0.10
100	0.10	0.10	0.11	0.10	0.10
101	0.09	0.09	0.10	0.09	0.09
102	0.09	0.09	0.10	0.09	0.09
103	0.09	0.09	0.09	0.09	0.08
104	0.07	0.08	0.08	0.08	0.07
105	0.08	0.08	0.08	0.08	0.07
106	0.08	0.08	0.08	0.08	0.07
107	0.08	0.08	0.09	0.09	0.08
108	0.09	0.09	0.10	0.10	0.09
109	0.10	0.10	0.11	0.11	0.10
110	0.10	0.10	0.10	0.10	0.09
111	0.11	0.11	0.11	0.11	0.10
112	0.12	0.12	0.12	0.12	0.11
113	0.12	0.12	0.13	0.13	0.11
114	0.10	0.10	0.11	0.10	0.09
115	0.09	0.09	0.10	0.09	0.08
116	0.08	0.08	0.09	0.09	0.08
117	0.07	0.08	0.08	0.08	0.07
118	0.07	0.08	0.08	0.08	0.07
119	0.07	0.08	0.08	0.09	0.07
120	0.09	0.09	0.09	0.09	0.08
121	0.09	0.10	0.10	0.10	0.09
122	0.11	0.11	0.12	0.11	0.11
123	0.13	0.13	0.15	0.14	0.13
124	0.13	0.13	0.14	0.13	0.12
125	0.11	0.11	0.12	0.12	0.11
126	0.09	0.10	0.10	0.10	0.10
127	0.09	0.09	0.10	0.10	0.09
128	0.08	0.08	0.09	0.09	0.08
129	0.08	0.08	0.09	0.09	0.08
130	0.08	0.08	0.09	0.09	0.08
131	0.09	0.09	0.10	0.10	0.08
132	0.09	0.09	0.10	0.10	0.09
133	0.10	0.10	0.11	0.10	0.09
134	0.11	0.11	0.12	0.11	0.10
135	0.10	0.10	0.12	0.11	0.10
136	0.11	0.11	0.13	0.12	0.10
137	0.12	0.13	0.14	0.12	0.11
138	0.11	0.11	0.12	0.11	0.10
139	0.10	0.10	0.12	0.10	0.10
140	0.10	0.10	0.12	0.11	0.10
141	0.11	0.11	0.12	0.11	0.10
142	0.12	0.12	0.13	0.12	0.12
143	0.12	0.13	0.14	0.13	n.a.
144	0.14	n.a.	0.16	0.14	n.a.
145	0.15	0.14	0.18	0.16	n.a.

146	0.18	0.16	0.21	0.19	0.13
147	0.22	0.19	0.26	0.23	0.15
148	0.24	0.22	0.29	0.25	0.17
149	0.22	0.20	0.27	0.23	0.18
150	0.21	0.20	0.25	0.22	0.20
151	0.18	0.18	0.22	0.19	0.17
152	0.15	0.16	0.18	0.16	0.15
153	0.14	0.14	0.16	0.15	0.14
154	0.13	0.13	0.14	0.13	0.13
155	0.14	0.14	0.14	0.14	0.14
156	0.12	0.13	n.a.	n.a.	0.13
157	0.11	0.12	n.a.	n.a.	0.11
158	0.11	0.11	n.a.	0.14	0.10
159	0.10	0.10	0.13	0.12	0.10
160	0.11	0.10	0.13	0.12	0.10
161	0.12	0.12	0.13	0.12	0.12
162	0.12	0.12	0.13	0.12	0.12
163	0.12	0.12	0.13	0.12	0.11
164	0.12	0.12	0.13	0.12	0.11
165	0.10	0.11	0.12	0.12	0.11
166	0.09	0.10	0.11	0.11	0.10
167	0.10	0.09	0.11	0.11	0.10
168	0.09	0.09	0.10	0.10	0.09
169	0.09	0.09	0.10	0.10	0.10
170	0.10	0.09	0.10	0.10	0.10
171	0.10	0.10	0.11	0.11	0.10
172	0.11	0.11	0.12	0.12	0.11
173	0.14	0.13	0.13	0.14	0.12
174	0.13	0.13	0.13	0.13	0.12
175	0.13	0.13	0.13	0.14	0.13
176	0.14	0.14	0.15	0.14	0.14
177	0.12	0.14	0.14	0.13	0.13
178	0.13	0.14	0.14	0.14	0.13
179	0.13	0.15	0.16	0.14	0.14
180	0.14	0.16	0.16	0.15	0.15
181	0.15	0.15	0.17	0.17	0.16
182	0.15	0.16	0.17	0.17	0.16
183	0.16	0.17	0.18	0.18	0.17
184	0.16	0.17	0.17	0.16	0.17
185	0.13	0.14	0.14	0.14	0.14
186	0.11	0.12	0.12	0.12	0.12
187	0.11	0.12	0.12	0.12	0.12
188	0.10	0.11	0.11	0.10	0.10
189	0.09	0.10	0.10	0.10	0.09
190	0.08	0.09	0.09	0.09	0.08
191	0.07	0.08	0.08	0.08	0.07
192	0.07	0.07	0.07	0.07	0.07
193	0.06	0.07	0.07	0.07	0.07
194	0.07	0.07	0.07	0.07	0.07
195	0.06	0.07	0.07	0.07	0.07

196	0.07	0.08	0.07	0.08	0.07
197	0.09	0.09	0.09	0.10	0.09
198	0.10	0.10	0.09	0.11	0.10
199	0.09	0.09	0.09	0.10	0.09
200	0.07	0.08	0.08	0.08	0.07
201	0.07	0.07	0.07	0.07	0.07
202	0.06	0.07	0.07	0.07	0.07
203	0.07	0.06	0.07	0.07	0.07
204	0.06	0.06	0.07	0.07	0.07
205	0.07	0.07	0.07	0.08	0.07
206	0.07	0.08	0.08	0.08	0.08
207	0.09	0.09	0.09	0.09	0.09
208	0.10	0.10	0.10	0.10	0.09
209	0.11	0.12	0.12	0.12	0.11
210	0.11	0.12	0.12	0.12	0.12
211	0.13	0.14	0.14	0.14	n.a.
212	0.13	0.14	0.14	0.14	0.15
213	0.15	0.16	0.16	0.15	0.15
214	0.15	0.16	0.16	0.15	0.17
215	0.14	0.15	0.15	0.14	0.15
P insertion	n.a.	n.a.	n.a.	n.a.	0.14
E insertion	n.a.	n.a.	n.a.	n.a.	0.14
D insertion	n.a.	n.a.	n.a.	n.a.	0.12
216	0.13	0.13	0.14	0.13	0.12
217	0.13	0.14	0.14	0.14	0.12
218	0.13	0.14	0.14	0.15	0.13
219	0.13	0.14	0.13	0.14	0.12
220	0.10	0.11	0.10	0.11	0.10
221	0.09	0.10	0.09	0.09	0.09
222	0.08	0.09	0.08	0.08	0.08
223	0.07	0.07	0.07	0.07	0.07
224	0.07	0.07	0.07	0.07	0.07
225	0.07	0.07	0.07	0.08	0.07
226	0.07	0.07	0.08	0.08	0.07
227	0.07	0.08	0.08	0.08	0.07
228	0.07	0.08	0.08	0.08	0.07
229	0.07	0.07	0.08	0.08	0.07
230	0.07	0.07	0.08	0.08	0.07
231	0.08	0.08	0.07	0.08	0.07
232	0.08	0.08	0.08	0.09	0.08
233	0.08	0.08	0.08	0.09	0.08
234	0.08	0.09	0.09	0.09	0.08
235	0.08	0.08	0.08	0.09	0.08
236	0.09	0.09	0.09	0.09	0.08
237	0.09	0.09	0.09	0.09	0.08
238	0.08	0.08	0.09	0.08	0.08
239	0.08	0.09	0.09	0.08	0.08
240	0.08	0.08	0.09	0.09	0.08
241	0.09	0.09	0.10	0.09	0.08
242	0.10	0.10	0.10	0.10	0.09

243	0.10	0.11	0.11	0.11	0.10
244	0.11	0.12	0.12	0.12	0.11
245	0.12	0.13	0.14	0.13	0.13
246	0.14	0.14	0.16	0.14	0.14
247	0.16	0.17	0.19	0.16	0.17
248	0.19	0.19	0.22	0.19	0.19
249	0.21	0.22	0.24	0.21	0.23
250	0.20	0.21	0.24	0.20	0.24
251	0.22	0.23	0.26	0.22	0.27
252	0.21	0.23	0.25	0.22	0.28
253	0.19	0.20	0.22	0.19	0.24
254	0.17	0.19	0.20	0.18	0.23
255	0.18	0.18	0.19	0.16	0.21
256	0.17	0.18	0.18	0.16	0.19
257	0.16	0.17	0.17	0.16	0.17
258	0.14	0.14	0.15	0.14	0.14
259	0.13	0.14	0.15	0.14	0.13
260	0.13	0.13	0.14	0.13	0.13
261	0.12	0.13	0.14	0.12	0.13
262	0.12	0.12	0.12	0.12	0.11
263	0.10	0.11	0.11	0.10	0.10
264	0.10	0.11	0.11	0.10	0.10
265	0.09	0.09	0.09	0.09	0.09
266	0.08	0.09	0.09	0.09	0.08
267	0.08	0.09	0.08	0.08	0.08
268	0.08	0.09	0.09	0.09	0.09
269	0.08	0.08	0.08	0.08	0.08
270	0.07	0.08	0.08	0.08	0.07
271	0.08	0.08	0.08	0.08	0.08
272	0.08	0.08	0.08	0.09	0.08
273	0.07	0.08	0.07	0.07	0.07
274	0.07	0.08	0.07	0.07	0.07
275	0.07	0.08	0.07	0.07	0.07
276	0.07	0.07	0.07	0.07	0.07
277	0.07	0.07	0.07	0.07	0.07
278	0.07	0.07	0.07	0.07	0.07
279	0.07	0.07	0.07	0.07	0.08
280	0.08	0.08	0.09	0.08	0.09
281	0.08	0.09	0.10	0.09	0.10
282	0.09	0.09	0.10	0.09	0.10
283	0.08	0.08	0.09	0.08	0.09
284	0.07	0.08	0.08	0.08	0.08
285	0.07	0.07	0.08	0.07	0.07
286	0.08	0.08	0.08	0.08	0.08
287	0.08	0.08	0.08	0.08	0.08
288	0.07	0.08	0.07	0.07	0.07
289	0.07	0.08	0.07	0.07	0.07
290	0.07	0.08	0.07	0.07	0.07
291	0.07	0.08	0.07	0.07	0.07
292	0.09	0.09	0.09	0.08	0.08

293	0.08	0.09	0.08	0.09	0.08
294	0.08	0.08	0.08	0.08	0.07
295	0.07	0.08	0.08	0.07	0.07
296	0.07	0.08	0.07	0.07	0.07
297	0.07	0.08	0.07	0.07	0.07
298	0.07	0.08	0.07	0.07	0.07
299	0.07	0.07	0.07	0.07	0.07
300	0.07	0.08	0.07	0.07	0.07
301	0.07	0.08	0.07	0.07	0.07
302	0.08	0.08	0.08	0.08	0.08
303	0.09	0.09	0.08	0.09	0.08
304	0.09	0.09	0.09	0.09	0.08
305	0.09	0.09	0.09	0.09	0.09
306	0.09	0.09	0.09	0.09	0.09
307	0.10	0.10	0.10	0.10	0.10
308	0.09	0.09	0.09	0.10	0.09
309	0.09	0.10	0.10	0.10	0.09
310	0.09	0.09	0.10	0.10	0.09
311	0.08	0.09	0.09	0.09	0.08
312	0.07	0.07	0.07	0.07	0.07
313	0.07	0.07	0.07	0.07	0.07
314	0.06	0.07	0.07	0.07	0.07
315	0.06	0.07	0.07	0.07	0.07
316	0.06	0.07	0.07	0.07	0.07
317	0.06	0.06	0.06	0.06	0.06
318	0.06	0.07	0.07	0.07	0.07
319	0.07	0.08	0.07	0.07	0.07
320	0.08	0.08	0.08	0.08	0.09
321	0.09	0.10	0.10	0.10	0.10
322	0.09	0.09	0.09	0.09	0.09
323	0.11	0.11	0.11	0.12	0.11
324	0.10	0.09	0.10	0.10	0.09
325	0.09	0.10	0.09	0.10	0.09
326	0.08	0.08	0.08	0.08	0.07
327	0.08	0.08	0.08	0.08	0.07
328	0.07	0.07	0.07	0.07	0.07
329	0.07	0.07	0.07	0.07	0.07
330	0.08	0.08	0.08	0.08	0.08
331	0.10	0.10	0.11	0.11	0.10
332	0.10	0.10	0.10	0.10	0.10
333	0.12	0.13	0.13	0.13	0.12
334	0.10	0.11	0.11	0.11	0.10
335	0.09	0.09	0.09	0.09	0.09
336	0.08	0.08	0.08	0.09	0.08
337	0.10	0.10	0.10	0.10	0.10
338	0.11	0.10	0.11	0.11	0.10
339	0.13	0.12	0.12	0.12	0.11
340	0.12	0.12	0.12	0.11	0.10
341	0.11	0.11	0.10	0.10	0.10
342	0.09	0.09	0.09	0.09	0.10

343	0.11	0.11	0.11	0.11	0.11
344	0.12	0.11	0.12	0.11	0.12
345	0.14	0.14	0.13	0.14	0.14
346	0.11	0.12	0.11	0.12	0.12
347	0.08	0.08	0.08	0.08	0.09
348	0.07	0.07	0.08	0.07	0.08
349	0.07	0.07	0.07	0.07	0.07
350	0.06	0.06	0.07	0.07	0.07
351	0.07	0.07	0.07	0.07	0.07
352	0.07	0.07	0.07	0.07	0.08
353	0.07	0.06	0.07	0.07	0.07
354	0.07	0.07	0.07	0.07	0.07
355	0.07	0.07	0.07	0.07	0.07
356	0.07	0.07	0.07	0.08	0.07
357	0.07	0.07	0.08	0.08	0.07
358	0.07	0.07	0.07	0.07	0.08
359	0.08	0.07	0.07	0.07	0.08
360	0.08	0.08	0.08	0.08	0.08
361	0.07	0.07	0.08	0.08	0.07
362	0.08	0.08	0.08	0.08	0.08
363	0.09	0.09	0.09	0.09	0.09
364	0.10	0.10	0.10	0.10	0.10
365	0.10	0.10	0.10	0.10	0.10
366	0.10	0.10	0.11	0.11	0.10
367	0.11	0.11	0.10	0.11	0.10
368	0.11	0.10	0.10	0.11	0.10
369	0.10	0.09	0.10	0.10	0.10
370	0.10	0.10	0.11	0.12	0.12
371	0.11	0.12	0.13	0.12	0.11
372	0.10	0.10	0.10	0.11	0.11
373	0.09	0.09	0.09	0.10	0.09
374	0.07	0.07	0.08	0.08	0.08
375	0.08	0.07	0.08	0.07	0.07
376	0.08	0.09	0.10	0.08	0.08
377	0.09	0.09	0.10	0.08	0.08
378	0.07	0.07	0.08	0.07	0.07
379	0.07	0.06	0.07	0.07	0.07
380	0.07	0.07	0.07	0.07	0.07
381	0.07	0.07	0.08	0.08	0.07
382	0.06	0.07	0.07	0.07	0.07
383	0.06	0.07	0.07	0.07	0.07
384	0.07	0.07	0.08	0.08	0.08
385	0.08	0.07	0.08	0.08	0.08
386	0.07	0.07	0.08	0.08	0.07
387	0.07	0.07	0.08	0.08	0.08
388	0.08	0.08	0.09	0.09	0.08
389	0.08	0.08	0.08	0.08	0.07
390	0.07	0.07	0.07	0.07	0.07
391	0.07	0.07	0.07	0.07	0.06
392	0.06	0.06	0.06	0.07	0.06

393	0.07	0.07	0.07	0.07	0.07
394	0.07	0.07	0.07	0.07	0.07
395	0.06	0.06	0.07	0.07	0.06
396	0.06	0.06	0.07	0.07	0.06
397	0.06	0.06	0.07	0.07	0.06
398	0.06	0.06	0.06	0.06	0.06
399	0.06	0.06	0.06	0.06	0.06
400	0.06	0.06	0.06	0.06	0.06
401	0.06	0.06	0.06	0.06	0.06
402	0.06	0.06	0.06	0.06	0.06
403	0.06	0.06	0.07	0.07	0.06
404	0.07	0.07	0.07	0.07	0.07
405	0.07	0.07	0.08	0.08	0.07
406	0.07	0.07	0.07	0.07	0.06
407	0.07	0.07	0.07	0.07	0.06
408	0.07	0.07	0.07	0.07	0.07
409	0.06	0.06	0.07	0.07	0.07
410	0.06	0.06	0.07	0.07	0.06
411	0.07	0.06	0.07	0.07	0.07
412	0.08	0.07	0.09	0.08	0.08
413	0.10	0.09	0.10	0.10	0.10
414	0.09	0.08	0.09	0.09	0.08
415	0.09	0.07	0.09	0.09	0.08
416	0.08	0.07	0.08	0.08	0.08
417	0.07	0.07	0.07	0.07	0.07
418	0.06	0.06	0.07	0.07	0.06
419	0.07	0.06	0.07	0.07	0.07
420	0.07	0.07	0.08	0.07	0.07
421	0.07	0.07	0.07	0.07	0.07
422	0.06	0.06	0.07	0.07	0.06
423	0.06	0.06	0.06	0.07	0.06
424	0.06	0.06	0.07	0.07	0.06
425	0.07	0.06	0.07	0.07	0.07
426	0.08	0.07	0.08	0.08	0.07
427	0.09	0.07	0.09	0.08	0.08
428	0.09	0.08	0.09	0.09	0.09
429	0.07	0.07	0.08	0.08	0.08
430	0.06	0.07	0.08	0.07	0.07
431	0.06	0.06	0.07	0.07	0.06
432	0.06	0.06	0.07	0.06	0.06
433	0.06	0.06	0.06	0.06	0.06
434	0.06	0.06	0.07	0.06	0.06
435	0.06	0.06	0.07	0.06	0.06
436	0.06	0.07	0.07	0.07	0.07
437	0.07	0.07	0.07	0.07	0.07
438	0.07	0.07	0.08	0.07	0.07
439	0.08	0.08	0.08	0.08	0.08
440	0.10	0.10	0.10	0.10	0.10
441	0.10	0.10	0.10	0.10	0.09
442	0.08	0.08	0.08	0.08	0.08

443	0.09	0.09	0.09	0.09	0.09
444	0.11	0.11	0.12	0.11	0.11
445	0.13	0.13	0.14	0.14	0.13
446	0.14	0.14	0.15	0.15	0.15
447	0.11	0.12	0.13	0.12	0.12
448	0.09	0.10	0.11	0.10	0.10
449	0.10	0.11	0.11	0.10	0.11
450	0.10	0.10	0.10	0.10	0.10
451	0.07	0.08	0.08	0.08	0.08
452	0.07	0.08	0.08	0.08	0.08
453	0.07	0.07	0.07	0.08	0.07
454	0.07	0.07	0.07	0.07	0.07
455	0.07	0.08	0.08	0.08	0.08
456	0.10	0.09	0.10	0.09	0.09
457	0.09	0.09	0.10	0.10	0.09
458	0.12	0.11	0.12	0.12	0.12
459	0.11	0.10	0.11	0.11	0.10
460	0.09	0.08	0.09	0.09	0.09
461	0.08	0.08	0.08	0.08	0.08
462	0.07	0.07	0.08	0.08	0.08
463	0.07	0.07	0.08	0.08	0.07
464	0.07	0.07	0.07	0.07	0.07
465	0.07	0.07	0.08	0.07	0.07
466	0.07	0.07	0.07	0.08	0.07
467	0.08	0.07	0.08	0.08	0.08
468	0.08	0.08	0.09	0.09	0.08
469	0.09	0.09	0.10	0.10	0.10
470	0.10	0.10	0.11	0.10	0.11
471	0.12	0.12	0.14	0.12	0.13
472	0.12	0.12	0.14	0.12	0.12
473	0.14	0.12	0.15	0.13	0.14
474	0.18	0.15	0.18	0.16	0.16
475	0.22	0.17	0.23	0.19	0.19
476	0.28	0.23	0.29	0.24	0.24
477	0.33	0.27	0.34	0.28	0.27
478	0.31	0.25	0.31	0.27	0.27
479	0.29	0.23	0.29	0.27	0.26
480	0.25	0.20	0.24	0.22	0.22
481	0.30	0.24	0.30	0.27	0.28
482	0.29	0.23	0.27	0.26	0.31
483	0.26	0.21	0.24	0.23	0.29
484	0.25	0.18	0.21	0.20	0.25
485	0.26	0.20	0.23	0.22	0.25
486	0.26	0.21	0.24	0.23	0.25
487	0.22	0.18	0.21	0.20	0.21
488	0.17	0.14	0.17	0.16	0.16
489	0.14	0.12	0.14	0.13	0.13
490	0.11	0.10	0.12	0.11	0.11
491	0.09	0.09	0.10	0.09	0.09
492	0.08	0.08	0.09	0.09	0.09

493	0.08	0.08	0.09	0.09	0.09
494	0.08	0.09	0.09	0.09	0.09
495	0.08	0.08	0.09	0.09	0.08
496	0.09	0.10	0.09	0.09	0.09
497	0.08	0.08	0.08	0.08	0.08
498	0.09	0.10	0.10	0.09	0.09
499	0.10	0.11	0.11	0.11	0.11
500	0.12	0.12	0.13	0.12	0.12
501	0.09	0.10	0.10	0.10	0.10
502	0.09	0.10	0.10	0.10	0.10
503	0.09	0.08	0.09	0.09	0.08
504	0.08	0.08	0.08	0.09	0.08
505	0.07	0.07	0.08	0.08	0.07
506	0.07	0.07	0.07	0.07	0.07
507	0.06	0.07	0.07	0.06	0.06
508	0.06	0.06	0.06	0.06	0.06
509	0.06	0.06	0.06	0.06	0.06
510	0.06	0.06	0.07	0.06	0.06
511	0.06	0.06	0.06	0.06	0.06
512	0.06	0.06	0.06	0.06	0.06
513	0.06	0.06	0.07	0.07	0.06
514	0.06	0.07	0.07	0.07	0.06
515	0.06	0.07	0.07	0.07	0.07
516	0.06	0.07	0.07	0.07	0.07
517	0.07	0.07	0.06	0.07	0.06
518	0.07	0.07	0.07	0.07	0.07
519	0.07	0.08	0.07	0.08	0.07
520	0.07	0.07	0.07	0.08	0.07
521	0.07	0.07	0.07	0.07	0.07
522	0.07	0.07	0.07	0.07	0.07
523	0.08	0.07	0.07	0.08	0.08
524	0.07	0.07	0.07	0.07	0.07
525	0.07	0.07	0.07	0.07	0.07
526	0.08	0.09	0.09	0.09	0.08
527	0.10	0.10	0.10	0.10	0.09
528	0.09	0.09	0.10	0.10	0.09
529	0.10	0.11	0.11	0.11	0.10
530	0.09	0.09	0.09	0.09	0.08
531	0.08	0.09	0.09	0.09	0.08
532	0.10	0.10	0.10	0.10	0.10
533	0.09	0.10	0.10	0.10	0.09
534	0.09	0.10	0.10	0.09	0.09
535	0.09	0.09	0.09	0.09	0.08
536	0.09	0.09	0.10	0.09	0.09
537	0.09	0.09	0.09	0.09	0.08
538	0.08	0.08	0.08	0.08	0.07
539	0.07	0.07	0.07	0.08	0.07
540	0.07	0.07	0.07	0.07	0.07
541	0.07	0.07	0.07	0.07	0.06
542	0.07	0.07	0.07	0.07	0.06

543	0.07	0.06	0.07	0.06	0.06
544	0.07	0.07	0.07	0.07	0.07
545	0.08	0.07	0.07	0.07	0.07
546	0.07	0.07	0.07	0.07	0.06
547	0.07	0.07	0.07	0.07	0.07
548	0.08	0.08	0.07	0.08	0.07
549	0.07	0.07	0.07	0.07	0.07
550	0.07	0.07	0.07	0.07	0.07
551	0.07	0.07	0.07	0.07	0.07
552	0.07	0.07	0.08	0.07	0.07
553	0.08	0.07	0.08	0.08	0.07
554	0.09	0.08	0.09	0.09	0.08
555	0.09	0.08	0.09	0.09	0.08
556	0.11	0.11	0.11	0.11	0.10
557	0.09	0.09	0.09	0.09	0.09
558	0.10	0.10	0.10	0.11	0.10
559	0.08	0.08	0.09	0.08	0.08
560	0.08	0.09	0.09	0.09	0.09
561	0.10	0.10	0.10	0.10	0.10
562	0.08	0.08	0.09	0.09	0.08
563	0.07	0.07	0.08	0.07	0.07
564	0.06	0.06	0.07	0.07	0.06
565	0.06	0.06	0.07	0.06	0.06
566	0.07	0.07	0.07	0.07	0.07
567	0.06	0.07	0.07	0.07	0.06
568	0.07	0.07	0.07	0.07	0.07
569	0.08	0.07	0.08	0.08	0.08
570	0.07	0.07	0.08	0.07	0.07
571	0.07	0.07	0.07	0.07	0.07
572	0.07	0.07	0.07	0.07	0.06
573	0.07	0.07	0.07	0.07	0.06
574	0.07	0.07	0.07	0.07	0.06
575	0.07	0.06	0.07	0.06	0.06
576	0.07	0.06	0.07	0.07	0.06
577	0.07	0.07	0.07	0.07	0.07
578	0.07	0.07	0.08	0.08	0.07
579	0.08	0.08	0.09	0.09	0.08
580	0.10	0.10	0.11	0.11	0.10
581	0.10	0.10	0.10	0.10	0.09
582	0.09	0.09	0.10	0.09	0.09
583	0.09	0.09	0.09	0.09	0.08
584	0.07	0.07	0.08	0.08	0.07
585	0.07	0.07	0.08	0.07	0.07
586	0.07	0.07	0.07	0.07	0.07
587	0.07	0.07	0.07	0.07	0.07
588	0.07	0.07	0.07	0.07	0.07
589	0.07	0.07	0.07	0.07	0.07
590	0.07	0.07	0.07	0.07	0.07
591	0.07	0.07	0.07	0.07	0.07
592	0.07	0.07	0.07	0.07	0.07

593	0.07	0.07	0.07	0.07	0.07
594	0.07	0.07	0.07	0.07	0.07
595	0.06	0.07	0.06	0.07	0.06
596	0.06	0.06	0.07	0.07	0.06
597	0.06	0.07	0.07	0.07	0.06
598	0.07	0.07	0.07	0.08	0.07
599	0.08	0.08	0.09	0.09	0.08
600	0.09	0.10	0.10	0.10	0.10
601	0.10	0.10	0.10	0.10	0.10
602	0.11	0.10	0.11	0.11	0.11
603	0.13	0.13	0.14	0.14	0.14
604	0.12	0.12	0.13	0.13	0.13
605	0.11	0.12	0.12	0.12	0.12
606	0.12	0.12	0.13	0.13	0.13
607	0.11	0.10	0.11	0.10	0.10
608	0.08	0.08	0.09	0.09	0.08
609	0.07	0.07	0.08	0.08	0.07
610	0.07	0.07	0.07	0.07	0.07
611	0.06	0.06	0.07	0.07	0.07
612	0.06	0.07	0.07	0.07	0.07
613	0.07	0.07	0.07	0.07	0.07
614	0.07	0.07	0.08	0.08	0.08
615	0.07	0.08	0.08	0.08	0.08
616	0.09	0.09	0.09	0.09	0.09
617	0.09	0.09	0.09	0.09	0.09
618	0.11	0.10	0.11	0.11	0.11
619	0.12	0.12	0.13	0.14	0.13
620	0.14	0.13	0.15	0.14	0.14
621	0.17	0.16	0.18	0.16	0.16
622	0.19	0.18	0.18	0.16	0.18
623	0.21	0.21	0.19	0.18	0.19
624	0.22	0.21	0.17	0.17	0.17
625	0.22	0.21	0.16	0.15	0.15
626	0.19	0.19	0.19	0.18	0.18
627	0.17	0.17	0.17	0.16	0.15
628	0.15	0.15	0.15	0.14	0.14
629	0.14	0.14	0.14	0.13	0.12
630	0.13	0.14	0.13	0.12	0.12
631	0.12	0.12	0.11	0.11	0.11
632	0.11	0.11	0.10	0.11	0.10
633	0.10	0.10	0.10	0.10	0.10
634	0.09	0.09	0.10	0.10	0.09
635	0.08	0.09	0.10	0.10	0.09
636	0.09	0.10	0.11	0.11	0.09
637	0.11	0.11	0.13	0.13	0.11
638	0.12	0.12	0.15	0.14	0.12
639	0.13	0.13	0.16	0.15	0.14
640	0.13	0.13	0.15	0.15	0.15
641	0.11	0.10	0.11	0.12	0.11
642	0.10	0.09	0.10	0.10	0.10

643	0.09	0.08	0.09	0.09	0.09
644	0.09	0.08	0.08	0.08	0.08
645	0.09	0.09	0.08	0.08	0.08
646	0.09	0.09	0.08	0.09	0.08
647	0.08	0.09	0.08	0.08	0.08
648	0.08	0.08	0.07	0.08	0.08
649	0.07	0.07	0.07	0.07	0.07
650	0.07	0.07	0.07	0.07	0.07
651	0.08	0.08	0.08	0.08	0.08
652	0.09	0.09	0.09	0.10	0.09
653	0.09	0.09	0.10	0.09	0.09
654	0.09	0.10	0.10	0.10	0.10
655	0.10	0.10	0.10	0.10	0.09
656	0.10	0.11	0.10	0.11	0.10
657	0.13	0.14	0.13	0.14	0.13
658	0.12	0.13	0.13	0.14	0.13
659	0.11	0.12	0.11	0.12	0.11
660	0.10	0.11	0.11	0.12	0.11
661	0.10	0.11	0.10	0.12	0.10
662	0.09	0.09	0.09	0.10	0.09
663	0.09	0.09	0.09	0.10	0.09
664	0.08	0.08	0.08	0.09	0.08
665	0.08	0.08	0.08	0.08	0.08
666	0.07	0.08	0.08	0.08	0.07
667	0.08	0.07	0.07	0.08	0.07
668	0.08	0.08	0.08	0.08	0.07
669	0.08	0.08	0.07	0.08	0.08
670	0.07	0.07	0.07	0.07	0.07
671	0.07	0.07	0.07	0.07	0.07
672	0.07	0.08	0.08	0.08	0.08
673	0.08	0.09	0.09	0.09	0.09
674	0.09	0.10	0.10	0.10	0.10
675	0.10	0.11	0.12	0.12	0.12
676	0.12	0.13	0.15	0.14	0.14
677	0.13	0.13	0.15	0.15	0.15
678	0.15	0.14	0.18	0.19	0.18
679	0.17	0.15	0.21	0.23	0.20
680	0.18	0.17	0.26	0.28	0.24
681	0.20	0.17	0.29	0.31	0.26
682	0.20	0.18	0.28	0.30	0.25
683	0.20	0.18	0.27	0.30	0.26
684	0.20	0.18	0.25	0.27	0.25
685	0.18	0.18	0.23	0.24	0.24
686	0.18	0.19	0.24	0.23	0.23
687	0.18	0.19	0.23	0.24	0.22
688	0.18	0.19	0.22	0.23	0.21
689	0.14	0.16	0.17	0.18	0.17
690	0.12	0.13	0.14	0.14	0.13
691	0.10	0.10	0.11	0.12	0.11
692	0.09	0.09	0.10	0.09	0.09

693	0.08	0.09	0.09	0.09	0.09
694	0.08	0.08	0.08	0.08	0.08
695	0.08	0.09	0.08	0.09	0.08
696	0.09	0.09	0.08	0.09	0.08
697	0.09	0.09	0.08	0.09	0.08
698	0.09	0.10	0.09	0.10	0.09
699	0.09	0.09	0.09	0.10	0.09
700	0.10	0.11	0.11	0.11	0.11
701	0.11	0.12	0.11	0.12	0.12
702	0.11	0.12	0.11	0.11	0.12
703	0.10	0.11	0.11	0.11	0.11
704	0.11	0.11	0.11	0.12	0.12
705	0.10	0.10	0.09	0.10	0.10
706	0.10	0.11	0.10	0.10	0.10
707	0.09	0.10	0.09	0.09	0.09
708	0.09	0.10	0.09	0.09	0.09
709	0.09	0.10	0.09	0.09	0.09
710	0.10	0.10	0.09	0.09	0.09
711	0.09	0.09	0.09	0.09	0.09
712	0.08	0.08	0.08	0.08	0.08
713	0.08	0.08	0.08	0.08	0.08
714	0.08	0.08	0.08	0.08	0.08
715	0.08	0.08	0.08	0.08	0.08
716	0.08	0.09	0.09	0.09	0.09
717	0.09	0.09	0.09	0.09	0.09
718	0.08	0.08	0.08	0.09	0.08
719	0.08	0.09	0.09	0.09	0.08
720	0.08	0.08	0.08	0.08	0.08
721	0.09	0.09	0.09	0.09	0.09
722	0.07	0.08	0.08	0.08	0.08
723	0.08	0.09	0.08	0.08	0.08
724	0.07	0.08	0.07	0.08	0.07
725	0.06	0.07	0.07	0.07	0.07
726	0.06	0.06	0.06	0.07	0.06
727	0.06	0.06	0.06	0.06	0.06
728	0.06	0.06	0.06	0.06	0.06
729	0.06	0.06	0.06	0.06	0.06
730	0.06	0.07	0.06	0.07	0.06
731	0.06	0.07	0.06	0.07	0.06
732	0.06	0.07	0.07	0.07	0.06
733	0.06	0.06	0.06	0.06	0.06
734	0.06	0.06	0.06	0.06	0.06
735	0.06	0.06	0.06	0.06	0.06
736	0.06	0.06	0.06	0.06	0.06
737	0.06	0.06	0.06	0.06	0.05
738	0.06	0.06	0.06	0.06	0.06
739	0.06	0.06	0.06	0.06	0.06
740	0.06	0.06	0.06	0.06	0.06
741	0.05	0.05	0.06	0.06	0.06
742	0.06	0.06	0.06	0.06	0.06

743	0.07	0.06	0.06	0.06	0.06
744	0.08	0.08	0.08	0.08	0.08
745	0.09	0.09	0.09	0.09	0.08
746	0.08	0.07	0.08	0.07	0.07
747	0.09	0.09	0.09	0.09	0.09
748	0.09	0.08	0.08	0.08	0.09
749	0.07	0.07	0.07	0.06	0.07
750	0.08	0.07	0.07	0.07	0.08
751	0.09	0.08	0.08	0.08	0.09
752	0.07	0.07	0.07	0.07	0.07
753	0.07	0.07	0.07	0.06	0.07
754	0.07	0.08	0.07	0.07	0.07
755	0.06	0.07	0.07	0.07	0.07
756	0.06	0.07	0.07	0.07	0.06
757	0.07	0.08	0.08	0.08	0.07
758	0.07	0.07	0.07	0.07	0.07
759	0.06	0.06	0.06	0.06	0.06
760	0.06	0.06	0.06	0.07	0.06
761	0.06	0.06	0.07	0.07	0.06
762	0.06	0.06	0.06	0.06	0.06
763	0.06	0.06	0.06	0.06	0.06
764	0.06	0.06	0.06	0.06	0.06
765	0.06	0.06	0.06	0.06	0.07
766	0.06	0.07	0.06	0.06	0.07
767	0.06	0.06	0.06	0.06	0.06
768	0.06	0.07	0.06	0.07	0.07
769	0.07	0.07	0.07	0.07	0.07
770	0.06	0.07	0.06	0.06	0.06
771	0.07	0.07	0.07	0.07	0.07
772	0.07	0.08	0.07	0.07	0.07
773	0.06	0.07	0.06	0.07	0.07
774	0.06	0.06	0.06	0.06	0.06
775	0.07	0.06	0.06	0.07	0.07
776	0.07	0.07	0.06	0.07	0.07
777	0.07	0.07	0.06	0.06	0.06
778	0.06	0.07	0.06	0.07	0.06
779	0.07	0.07	0.06	0.07	0.07
780	0.07	0.07	0.06	0.07	0.07
781	0.07	0.06	0.06	0.07	0.06
782	0.07	0.07	0.07	0.07	0.07
783	0.08	0.08	0.07	0.08	0.08
784	0.08	0.07	0.08	0.08	0.08
785	0.08	0.09	0.09	0.09	0.09
786	0.10	0.11	0.11	0.11	0.10
787	0.09	0.09	0.09	0.10	0.09
788	0.09	0.09	0.08	0.09	0.09
789	0.08	0.09	0.08	0.09	0.09
790	0.10	0.10	0.09	0.11	0.10
791	0.11	0.12	0.10	0.11	0.11
792	0.12	0.13	0.12	0.12	0.12

793	0.14	0.14	0.12	0.13	0.13
794	0.12	0.12	0.11	0.11	0.11
795	0.12	0.12	0.12	0.12	0.12
796	0.10	0.11	0.10	0.10	0.10
797	0.10	0.10	0.09	0.10	0.09
798	0.12	0.12	0.11	0.11	0.11
799	0.11	0.11	0.10	0.11	0.11
800	0.09	0.09	0.09	0.09	0.09
801	0.09	0.09	0.09	0.09	0.09
802	0.08	0.09	0.08	0.08	0.08
803	0.10	0.10	0.09	0.09	0.09
804	0.09	0.09	0.09	0.09	0.09
805	0.08	0.08	0.07	0.08	0.08
806	0.08	0.09	0.08	0.09	0.09
807	0.10	0.10	0.10	0.10	0.10
808	0.14	0.14	0.13	0.14	0.13
809	0.19	0.19	0.18	0.18	0.18
810	0.21	0.21	0.20	0.20	0.20
811	0.18	0.18	0.18	0.17	0.17
812	0.20	0.20	0.20	0.18	0.18
813	0.14	0.14	0.15	0.14	0.14
814	0.12	0.12	0.11	0.11	0.11
815	0.09	0.09	0.09	0.09	0.09
816	0.08	0.08	0.08	0.09	0.08
817	0.08	0.09	0.09	0.09	0.09
818	0.08	0.08	0.08	0.09	0.08
819	0.07	0.08	0.07	0.08	0.07
820	0.08	0.09	0.08	0.09	0.09
821	0.09	0.09	0.09	0.09	0.09
822	0.08	0.08	0.08	0.09	0.08
823	0.08	0.09	0.09	0.09	0.09
824	0.10	0.10	0.10	0.10	0.10
825	0.09	0.10	0.10	0.10	0.10
826	0.09	0.10	0.09	0.10	0.09
827	0.10	0.12	0.10	0.10	0.11
828	0.09	0.12	0.10	0.11	0.11
829	0.10	0.11	0.10	0.11	0.11
830	0.10	0.13	0.11	0.11	0.13
831	0.12	0.16	0.11	0.12	0.14
832	0.12	0.16	0.11	0.11	0.12
833	0.10	0.12	0.08	0.09	0.09
834	0.09	0.10	0.08	0.08	0.08
835	0.09	0.09	0.08	0.08	0.08
836	0.09	0.09	0.08	0.08	0.08
837	0.09	0.08	0.08	0.08	0.08
838	0.11	0.09	0.09	0.10	0.10
839	0.11	0.09	0.09	0.09	0.10
840	0.09	0.08	0.08	0.08	0.08
841	0.10	0.09	0.08	0.09	0.09
842	0.12	0.11	0.10	0.10	0.11

843	0.10	0.12	0.11	0.10	0.11
844	0.11	0.11	0.11	0.10	0.10
845	0.10	0.12	0.13	0.12	0.11
846	0.10	0.12	0.12	0.11	0.11
847	0.09	0.10	0.10	0.09	0.09
848	0.09	0.09	0.09	0.09	0.09
849	0.08	0.08	0.08	0.08	0.09
850	0.08	0.08	0.07	0.08	0.08
851	0.08	0.07	0.07	0.07	0.07
852	0.08	0.07	0.07	0.08	0.07
853	0.07	0.07	0.06	0.07	0.07
854	0.07	0.07	0.07	0.07	0.07
855	0.07	0.06	0.07	0.07	0.06
856	0.07	0.06	0.07	0.07	0.06
857	0.07	0.07	0.07	0.07	0.07
858	0.06	0.06	0.06	0.06	0.06
859	0.06	0.06	0.06	0.06	0.06
860	0.06	0.06	0.06	0.06	0.06
861	0.06	0.07	0.06	0.07	0.07
862	0.06	0.08	0.07	0.08	0.08
863	0.07	0.07	0.07	0.07	0.07
864	0.07	0.07	0.07	0.07	0.07
865	0.07	0.08	0.07	0.08	0.08
866	0.08	0.09	0.08	0.08	0.08
867	0.08	0.09	0.09	0.08	0.08
868	0.08	0.09	0.09	0.08	0.08
869	0.07	0.08	0.07	0.08	0.07
870	0.07	0.07	0.07	0.07	0.07
871	0.07	0.08	0.08	0.08	0.07
872	0.08	0.08	0.07	0.08	0.07
873	0.07	0.07	0.07	0.07	0.07
874	0.07	0.07	0.07	0.07	0.07
875	0.07	0.08	0.07	0.08	0.07
876	0.07	0.07	0.07	0.08	0.07
877	0.07	0.07	0.06	0.07	0.07
878	0.07	0.07	0.06	0.07	0.07
879	0.07	0.08	0.07	0.08	0.07
880	0.07	0.08	0.07	0.07	0.07
881	0.07	0.07	0.07	0.07	0.07
882	0.07	0.07	0.07	0.07	0.07
883	0.07	0.08	0.07	0.07	0.07
884	0.07	0.07	0.07	0.07	0.07
885	0.08	0.08	0.07	0.08	0.08
886	0.08	0.07	0.07	0.08	0.08
887	0.07	0.07	0.07	0.08	0.07
888	0.08	0.08	0.07	0.08	0.08
889	0.09	0.09	0.09	0.09	0.09
890	0.10	0.09	0.09	0.09	0.09
891	0.12	0.11	0.11	0.11	0.11
892	0.10	0.10	0.10	0.09	0.10

893	0.09	0.08	0.08	0.09	0.09
894	0.08	0.08	0.08	0.08	0.08
895	0.08	0.08	0.08	0.08	0.08
896	0.07	0.07	0.07	0.07	0.07
897	0.08	0.08	0.07	0.08	0.07
898	0.08	0.08	0.07	0.08	0.07
899	0.08	0.08	0.08	0.08	0.08
900	0.07	0.07	0.07	0.07	0.08
901	0.07	0.07	0.07	0.07	0.07
902	0.07	0.07	0.07	0.07	0.07
903	0.07	0.07	0.07	0.07	0.07
904	0.07	0.07	0.07	0.07	0.07
905	0.06	0.06	0.06	0.07	0.07
906	0.07	0.06	0.06	0.07	0.07
907	0.07	0.07	0.07	0.07	0.07
908	0.07	0.07	0.07	0.08	0.07
909	0.07	0.07	0.07	0.08	0.07
910	0.08	0.08	0.08	0.09	0.08
911	0.07	0.07	0.07	0.08	0.07
912	0.07	0.07	0.07	0.08	0.08
913	0.07	0.07	0.07	0.08	0.08
914	0.08	0.08	0.08	0.09	0.08
915	0.08	0.08	0.08	0.09	0.08
916	0.08	0.08	0.08	0.08	0.08
917	0.09	0.09	0.09	0.09	0.09
918	0.09	0.09	0.09	0.10	0.09
919	0.09	0.09	0.09	0.10	0.09
920	0.09	0.09	0.09	0.10	0.09
921	0.11	0.10	0.10	0.11	0.10
922	0.10	0.10	0.10	0.10	0.10
923	0.09	0.09	0.09	0.09	0.09
924	0.09	0.09	0.09	0.09	0.09
925	0.10	0.10	0.10	0.10	0.10
926	0.09	0.09	0.09	0.10	0.09
927	0.09	0.09	0.08	0.09	0.08
928	0.09	0.09	0.09	0.10	0.09
929	0.10	0.10	0.10	0.10	0.09
930	0.09	0.10	0.09	0.10	0.09
931	0.09	0.10	0.09	0.10	0.09
932	0.10	0.11	0.10	0.11	0.10
933	0.10	0.11	0.10	0.11	0.10
934	0.09	0.10	0.09	0.10	0.09
935	0.09	0.10	0.10	0.11	0.09
936	0.11	0.12	0.11	0.12	0.11
937	0.11	0.12	0.11	0.12	0.11
938	0.10	0.11	0.11	0.11	0.10
939	0.12	0.12	0.13	0.14	0.12
940	0.14	0.13	0.14	0.15	0.13
941	0.15	0.15	0.16	0.15	0.14
942	0.16	0.17	0.17	0.14	0.14

943	0.12	0.15	0.14	0.13	0.14
944	0.12	0.13	0.12	0.12	0.12
945	0.10	0.10	0.09	0.10	0.09
946	0.11	0.11	0.10	0.10	0.10
947	0.09	0.09	0.09	0.09	0.09
948	0.07	0.07	0.07	0.08	0.07
949	0.08	0.08	0.08	0.08	0.08
950	0.08	0.08	0.09	0.08	0.08
951	0.07	0.07	0.08	0.08	0.07
952	0.07	0.07	0.07	0.08	0.07
953	0.08	0.08	0.08	0.08	0.08
954	0.08	0.07	0.08	0.08	0.07
955	0.07	0.06	0.07	0.07	0.07
956	0.08	0.07	0.07	0.08	0.07
957	0.08	0.07	0.08	0.08	0.07
958	0.07	0.07	0.06	0.07	0.06
959	0.07	0.06	0.06	0.07	0.06
960	0.07	0.07	0.07	0.07	0.06
961	0.07	0.07	0.07	0.07	0.06
962	0.06	0.06	0.06	0.06	0.06
963	0.06	0.06	0.06	0.07	0.06
964	0.07	0.07	0.07	0.07	0.06
965	0.06	0.06	0.06	0.06	0.06
966	0.06	0.06	0.07	0.06	0.06
967	0.07	0.07	0.07	0.06	0.06
968	0.06	0.07	0.07	0.06	0.06
969	0.07	0.07	0.06	0.06	0.06
970	0.06	0.07	0.07	0.07	0.06
971	0.07	0.08	0.08	0.08	0.07
972	0.06	0.07	0.07	0.07	0.07
973	0.07	0.07	0.08	0.07	0.07
974	0.07	0.07	0.07	0.07	0.06
975	0.07	0.07	0.07	0.07	0.07
976	0.07	0.07	0.07	0.07	0.06
977	0.07	0.07	0.06	0.07	0.06
978	0.07	0.07	0.07	0.07	0.07
979	0.06	0.07	0.07	0.07	0.06
980	0.06	0.06	0.06	0.06	0.06
981	0.06	0.07	0.06	0.07	0.07
982	0.07	0.07	0.06	0.07	0.07
983	0.06	0.06	0.07	0.07	0.06
984	0.06	0.06	0.07	0.07	0.06
985	0.07	0.07	0.07	0.07	0.07
986	0.08	0.08	0.08	0.08	0.08
987	0.08	0.08	0.09	0.09	0.08
988	0.07	0.07	0.08	0.08	0.07
989	0.07	0.07	0.07	0.07	0.07
990	0.08	0.07	0.07	0.07	0.08
991	0.08	0.07	0.07	0.08	0.08
992	0.07	0.06	0.06	0.07	0.06

993	0.06	0.06	0.06	0.06	0.06
994	0.06	0.05	0.06	0.06	0.06
995	0.06	0.06	0.06	0.06	0.06
996	0.06	0.06	0.06	0.06	0.05
997	0.05	0.06	0.06	0.06	0.05
998	0.06	0.06	0.06	0.06	0.06
999	0.06	0.06	0.06	0.06	0.06
1000	0.05	0.05	0.06	0.05	0.05
1001	0.05	0.05	0.06	0.05	0.05
1002	0.05	0.05	0.06	0.06	0.05
1003	0.05	0.05	0.05	0.06	0.05
1004	0.05	0.05	0.05	0.06	0.05
1005	0.05	0.05	0.06	0.06	0.05
1006	0.06	0.05	0.06	0.06	0.05
1007	0.06	0.05	0.06	0.06	0.05
1008	0.06	0.06	0.06	0.06	0.06
1009	0.06	0.06	0.06	0.06	0.06
1010	0.06	0.05	0.06	0.06	0.06
1011	0.05	0.05	0.06	0.06	0.06
1012	0.05	0.06	0.06	0.06	0.06
1013	0.06	0.06	0.06	0.06	0.06
1014	0.06	0.06	0.06	0.06	0.06
1015	0.06	0.06	0.06	0.06	0.06
1016	0.06	0.06	0.07	0.07	0.07
1017	0.06	0.06	0.07	0.07	0.07
1018	0.06	0.06	0.06	0.06	0.06
1019	0.06	0.06	0.06	0.06	0.06
1020	0.06	0.07	0.07	0.06	0.07
1021	0.06	0.06	0.07	0.06	0.06
1022	0.06	0.06	0.06	0.06	0.06
1023	0.06	0.06	0.06	0.06	0.07
1024	0.06	0.06	0.06	0.06	0.06
1025	0.06	0.06	0.06	0.06	0.06
1026	0.06	0.06	0.06	0.06	0.06
1027	0.05	0.05	0.06	0.06	0.06
1028	0.06	0.06	0.06	0.06	0.06
1029	0.05	0.05	0.06	0.06	0.06
1030	0.05	0.05	0.06	0.06	0.06
1031	0.05	0.05	0.05	0.06	0.06
1032	0.05	0.05	0.06	0.06	0.06
1033	0.06	0.05	0.06	0.06	0.06
1034	0.06	0.06	0.06	0.06	0.06
1035	0.07	0.07	0.07	0.07	0.07
1036	0.06	0.06	0.06	0.06	0.06
1037	0.06	0.06	0.07	0.06	0.07
1038	0.07	0.07	0.07	0.08	0.08
1039	0.06	0.06	0.06	0.07	0.07
1040	0.06	0.06	0.07	0.07	0.07
1041	0.06	0.07	0.07	0.07	0.07
1042	0.06	0.06	0.07	0.07	0.07

1043	0.06	0.06	0.06	0.07	0.06
1044	0.08	0.07	0.07	0.07	0.08
1045	0.07	0.07	0.07	0.07	0.08
1046	0.08	0.08	0.07	0.07	0.08
1047	0.07	0.07	0.07	0.07	0.07
1048	0.06	0.06	0.06	0.07	0.06
1049	0.06	0.06	0.06	0.06	0.06
1050	0.06	0.06	0.06	0.06	0.06
1051	0.06	0.06	0.06	0.06	0.06
1052	0.06	0.07	0.06	0.07	0.06
1053	0.06	0.06	0.06	0.06	0.06
1054	0.06	0.06	0.06	0.06	0.06
1055	0.07	0.07	0.07	0.07	0.07
1056	0.07	0.07	0.07	0.07	0.07
1057	0.07	0.08	0.07	0.07	0.07
1058	0.06	0.07	0.07	0.07	0.07
1059	0.06	0.07	0.06	0.07	0.07
1060	0.06	0.06	0.06	0.06	0.06
1061	0.06	0.06	0.06	0.07	0.06
1062	0.06	0.06	0.06	0.07	0.06
1063	0.06	0.06	0.06	0.07	0.06
1064	0.06	0.07	0.06	0.07	0.06
1065	0.06	0.07	0.06	0.07	0.06
1066	0.07	0.07	0.07	0.07	0.06
1067	0.07	0.07	0.07	0.07	0.07
1068	0.07	0.08	0.07	0.08	0.08
1069	0.08	0.09	0.08	0.09	0.09
1070	0.09	0.10	0.09	0.10	0.10
1071	0.09	0.09	0.09	0.10	0.09
1072	0.09	0.09	0.09	0.10	0.09
1073	0.09	0.09	0.09	0.10	0.09
1074	0.09	0.09	0.09	0.10	0.09
1075	0.09	0.09	0.09	0.09	0.09
1076	0.09	0.10	0.09	0.09	0.09
1077	0.09	0.09	0.08	0.09	0.09
1078	0.09	0.09	0.09	0.09	0.09
1079	0.09	0.09	0.09	0.09	0.09
1080	0.09	0.09	0.09	0.09	0.09
1081	0.09	0.10	0.09	0.10	0.10
1082	0.10	0.11	0.11	0.11	0.10
1083	0.10	0.12	0.12	0.12	0.12
1084	0.13	0.15	0.15	0.14	0.14
1085	0.13	0.15	0.14	0.14	0.14
1086	0.11	0.12	0.12	0.12	0.11
1087	0.10	0.10	0.10	0.10	0.10
1088	0.08	0.09	0.09	0.09	0.09
1089	0.08	0.08	0.08	0.09	0.08
1090	0.08	0.08	0.08	0.08	0.09
1091	0.08	0.08	0.08	0.09	0.09
1092	0.08	0.08	0.08	0.09	0.09

1093	0.08	0.08	0.08	0.08	0.08
1094	0.07	0.08	0.07	0.07	0.08
1095	0.08	0.08	0.08	0.08	0.08
1096	0.08	0.09	0.08	0.09	0.09
1097	0.10	0.10	0.10	0.10	0.10
1098	0.13	0.13	0.13	0.14	0.13
1099	0.18	0.17	0.18	0.19	0.17
1100	0.19	0.18	0.18	0.20	0.18
1101	0.13	0.13	0.13	0.14	0.13
1102	0.10	0.10	0.10	0.10	0.10
1103	0.09	0.09	0.09	0.09	0.09
1104	0.08	0.08	0.08	0.08	0.08
1105	0.08	0.08	0.07	0.08	0.08
1106	0.08	0.08	0.07	0.08	0.08
1107	0.08	0.08	0.07	0.08	0.08
1108	0.08	0.08	0.08	0.08	0.08
1109	0.08	0.08	0.08	0.08	0.08
1110	0.08	0.08	0.08	0.09	0.08
1111	0.09	0.09	0.08	0.09	0.09
1112	0.09	0.10	0.09	0.10	0.10
1113	0.09	0.09	0.09	0.10	0.09
1114	0.09	0.10	0.10	0.10	0.10
1115	0.09	0.09	0.09	0.10	0.09
1116	0.09	0.10	0.09	0.10	0.10
1117	0.10	0.10	0.10	0.10	0.10
1118	0.10	0.10	0.10	0.10	0.10
1119	0.09	0.09	0.09	0.09	0.09
1120	0.08	0.09	0.09	0.09	0.09
1121	0.09	0.09	0.09	0.09	0.09
1122	0.10	0.10	0.10	0.10	0.10
1123	0.10	0.11	0.10	0.11	0.10
1124	0.12	0.13	0.13	0.13	0.13
1125	0.12	0.13	0.13	0.13	0.12
1126	0.11	0.12	0.12	0.12	0.12
1127	0.13	0.14	0.14	0.14	0.14
1128	0.14	0.14	0.14	0.14	0.14
1129	0.11	0.12	0.12	0.12	0.12
1130	0.12	0.13	0.12	0.13	0.12
1131	0.12	0.13	0.12	0.13	0.12
1132	0.10	0.11	0.11	0.12	0.11
1133	0.11	0.11	0.11	0.12	0.11
1134	0.12	0.13	0.12	0.13	0.12
1135	0.11	0.12	0.12	0.12	0.12
1136	0.12	0.13	0.12	0.12	0.13
1137	0.10	0.11	0.12	0.11	0.12
1138	0.11	0.12	0.13	0.12	0.13
1139	0.14	0.16	0.17	0.15	0.18

Supplementary Table S2. Average Cα-positional deviations in the 1 ns after LA removal

	Deviation after 1 ns (nm)				
Cα atom	Wild type	Alpha	Delta	Delta plus	Omicron
1	0.27	0.32	0.42	0.28	0.37
2	0.21	0.25	0.33	0.24	0.28
3	0.20	0.21	0.28	0.22	0.25
4	0.20	0.20	0.26	0.22	0.24
5	0.21	0.20	0.25	0.22	0.25
6	0.21	0.19	0.26	0.23	0.24
7	0.22	0.20	0.27	0.22	0.25
8	0.21	0.20	0.26	0.23	0.24
9	0.20	0.21	0.27	0.22	0.23
10	0.20	0.22	0.26	0.23	0.21
11	0.18	0.20	0.24	0.22	0.19
12	0.18	0.19	0.22	0.22	0.18
13	0.16	0.16	0.21	0.20	0.16
14	0.16	0.16	0.20	0.19	0.16
15	0.14	0.14	0.18	0.17	0.14
16	0.16	0.15	0.19	0.18	0.15
17	0.17	0.17	0.20	0.19	0.16
18	0.17	0.17	0.18	0.18	0.16
19	0.19	0.20	0.20	0.20	0.19
20	0.19	0.19	0.20	0.20	0.18
21	0.16	0.17	0.18	0.18	0.17
22	0.18	0.19	0.19	0.19	0.17
23	0.19	0.19	0.20	0.20	0.19
24	0.18	0.19	0.20	0.20	0.19
25	0.19	0.20	0.21	0.20	0.20
26	0.17	0.18	0.19	0.18	0.18
27	0.14	0.15	0.16	0.16	0.15
28	0.13	0.14	0.14	0.14	0.14
29	0.12	0.12	0.12	0.13	0.13
30	0.11	0.12	0.12	0.12	0.12
31	0.10	0.11	0.10	0.10	0.11
32	0.10	0.10	0.10	0.10	0.10
33	0.10	0.10	0.10	0.10	0.09
34	0.10	0.10	0.09	0.10	0.09
35	0.10	0.09	0.09	0.09	0.09
36	0.09	0.09	0.08	0.09	0.09
37	0.09	0.08	0.08	0.08	0.08
38	0.08	0.08	0.08	0.08	0.08
39	0.09	0.09	0.09	0.09	0.09
40	0.08	0.08	0.08	0.08	0.08
41	0.08	0.08	0.08	0.08	0.07
42	0.08	0.08	0.08	0.08	0.07
43	0.08	0.08	0.08	0.08	0.07
44	0.08	0.08	0.08	0.08	0.07
45	0.08	0.09	0.09	0.09	0.09
46	0.10	0.10	0.10	0.10	0.09
47	0.09	0.10	0.09	0.10	0.09
48	0.10	0.10	0.09	0.10	0.09

49	0.09	0.09	0.09	0.10	0.09
50	0.10	0.10	0.09	0.10	0.09
51	0.10	0.09	0.09	0.10	0.09
52	0.09	0.09	0.09	0.10	0.09
53	0.09	0.09	0.09	0.09	0.08
54	0.09	0.08	0.08	0.09	0.08
55	0.09	0.08	0.08	0.09	0.08
56	0.09	0.09	0.08	0.09	0.08
57	0.09	0.09	0.09	0.09	0.09
58	0.09	0.09	0.09	0.09	0.09
59	0.11	0.11	0.11	0.11	0.10
60	0.12	0.12	0.11	0.12	0.12
61	0.12	0.12	0.12	0.12	0.12
62	0.11	0.11	0.11	0.11	0.11
63	0.11	0.11	0.11	0.12	0.11
64	0.11	0.12	0.11	0.12	0.11
65	0.11	0.12	0.12	0.12	0.12
66	0.12	0.13	0.13	0.13	0.12
67	0.13	0.13	0.14	0.14	0.13
68	0.15	0.15	0.16	0.16	0.14
69	0.17	n.a.	0.18	0.18	n.a.
70	0.20	n.a.	0.22	0.21	n.a.
71	0.28	0.17	0.28	0.27	0.16
72	0.35	0.22	0.34	0.33	0.20
73	0.39	0.27	0.37	0.37	0.26
74	0.37	0.29	0.34	0.34	0.27
75	0.28	0.27	0.27	0.27	0.25
76	0.21	0.20	0.22	0.21	0.20
77	0.16	0.17	0.18	0.18	0.16
78	0.15	0.15	0.16	0.16	0.15
79	0.14	0.13	0.14	0.14	0.13
80	0.12	0.12	0.13	0.13	0.12
81	0.11	0.11	0.12	0.13	0.11
82	0.11	0.12	0.12	0.13	0.12
83	0.10	0.11	0.11	0.12	0.10
84	0.10	0.10	0.11	0.11	0.10
85	0.10	0.10	0.11	0.11	0.10
86	0.09	0.09	0.10	0.10	0.09
87	0.12	0.12	0.12	0.13	0.12
88	0.12	0.12	0.11	0.13	0.11
89	0.09	0.09	0.09	0.10	0.09
90	0.09	0.08	0.08	0.09	0.08
91	0.08	0.08	0.08	0.08	0.08
92	0.09	0.08	0.09	0.09	0.08
93	0.09	0.09	0.09	0.09	0.09
94	0.10	0.10	0.10	0.10	0.10
95	0.11	0.11	0.11	0.12	0.11
96	0.12	0.12	0.12	0.12	0.12
97	0.13	0.14	0.14	0.15	0.13
98	0.13	0.14	0.15	0.15	0.13

99	0.12	0.13	0.13	0.14	0.12
100	0.12	0.12	0.13	0.13	0.12
101	0.10	0.10	0.11	0.11	0.10
102	0.10	0.10	0.11	0.11	0.10
103	0.10	0.10	0.10	0.10	0.10
104	0.09	0.09	0.09	0.09	0.09
105	0.09	0.09	0.09	0.09	0.09
106	0.09	0.09	0.09	0.09	0.09
107	0.09	0.10	0.10	0.10	0.09
108	0.10	0.11	0.11	0.12	0.10
109	0.12	0.12	0.13	0.13	0.12
110	0.11	0.11	0.12	0.12	0.11
111	0.12	0.12	0.14	0.14	0.12
112	0.13	0.14	0.15	0.15	0.13
113	0.13	0.14	0.16	0.15	0.14
114	0.11	0.12	0.13	0.13	0.11
115	0.10	0.10	0.11	0.11	0.10
116	0.09	0.09	0.10	0.10	0.09
117	0.09	0.09	0.09	0.09	0.08
118	0.09	0.09	0.10	0.10	0.09
119	0.09	0.09	0.10	0.09	0.09
120	0.10	0.10	0.11	0.11	0.10
121	0.11	0.11	0.12	0.12	0.11
122	0.13	0.13	0.15	0.13	0.13
123	0.16	0.16	0.18	0.16	0.16
124	0.15	0.15	0.18	0.16	0.15
125	0.13	0.13	0.15	0.14	0.13
126	0.11	0.12	0.12	0.12	0.12
127	0.10	0.11	0.12	0.12	0.11
128	0.10	0.10	0.11	0.11	0.10
129	0.10	0.10	0.11	0.11	0.10
130	0.09	0.09	0.10	0.10	0.09
131	0.10	0.10	0.11	0.11	0.10
132	0.10	0.11	0.12	0.12	0.11
133	0.10	0.11	0.12	0.12	0.11
134	0.12	0.12	0.14	0.14	0.12
135	0.11	0.12	0.14	0.14	0.12
136	0.12	0.13	0.15	0.15	0.13
137	0.13	0.14	0.16	0.15	0.14
138	0.13	0.12	0.14	0.14	0.13
139	0.12	0.12	0.13	0.13	0.12
140	0.12	0.12	0.13	0.13	0.12
141	0.12	0.12	0.13	0.13	0.12
142	0.14	0.14	0.16	0.15	0.14
143	0.14	0.15	0.17	0.16	n.a.
144	0.16	n.a.	0.19	0.18	n.a.
145	0.18	0.17	0.23	0.21	n.a.
146	0.23	0.20	0.27	0.24	0.16
147	0.28	0.24	0.33	0.28	0.19
148	0.29	0.27	0.36	0.30	0.21

149	0.26	0.25	0.34	0.28	0.22
150	0.26	0.25	0.33	0.27	0.24
151	0.21	0.22	0.27	0.23	0.21
152	0.17	0.19	0.22	0.20	0.19
153	0.16	0.17	0.19	0.19	0.18
154	0.15	0.15	0.17	0.16	0.15
155	0.16	0.16	0.16	0.17	0.17
156	0.14	0.15	n.a.	n.a.	0.15
157	0.13	0.13	n.a.	n.a.	0.14
158	0.12	0.12	n.a.	0.16	0.13
159	0.12	0.11	0.15	0.15	0.11
160	0.12	0.12	0.15	0.15	0.12
161	0.13	0.14	0.17	0.14	0.14
162	0.13	0.14	0.16	0.15	0.14
163	0.13	0.13	0.15	0.15	0.13
164	0.13	0.14	0.16	0.16	0.13
165	0.12	0.13	0.15	0.14	0.13
166	0.11	0.12	0.13	0.13	0.12
167	0.11	0.12	0.14	0.13	0.12
168	0.10	0.11	0.12	0.12	0.11
169	0.11	0.12	0.13	0.13	0.11
170	0.11	0.12	0.13	0.13	0.11
171	0.12	0.13	0.14	0.14	0.12
172	0.14	0.14	0.15	0.15	0.14
173	0.16	0.17	0.17	0.17	0.15
174	0.16	0.16	0.17	0.17	0.14
175	0.17	0.17	0.17	0.18	0.14
176	0.18	0.18	0.19	0.18	0.15
177	0.15	0.17	0.18	0.17	0.15
178	0.16	0.17	0.19	0.17	0.16
179	0.16	0.18	0.21	0.17	0.18
180	0.17	0.20	0.22	0.19	0.18
181	0.19	0.20	0.22	0.22	0.20
182	0.19	0.20	0.22	0.23	0.20
183	0.20	0.21	0.23	0.24	0.21
184	0.18	0.20	0.20	0.22	0.20
185	0.16	0.17	0.17	0.18	0.17
186	0.13	0.14	0.14	0.15	0.13
187	0.13	0.13	0.14	0.14	0.13
188	0.12	0.12	0.12	0.13	0.12
189	0.11	0.11	0.11	0.11	0.10
190	0.09	0.10	0.10	0.10	0.10
191	0.09	0.09	0.09	0.09	0.09
192	0.08	0.08	0.08	0.08	0.08
193	0.08	0.07	0.07	0.07	0.07
194	0.08	0.07	0.07	0.08	0.07
195	0.08	0.08	0.08	0.08	0.07
196	0.08	0.08	0.08	0.09	0.08
197	0.10	0.10	0.10	0.11	0.10
198	0.12	0.13	0.12	0.14	0.11

199	0.11	0.11	0.11	0.12	0.10
200	0.09	0.09	0.09	0.09	0.08
201	0.08	0.08	0.08	0.08	0.08
202	0.07	0.07	0.08	0.08	0.08
203	0.08	0.07	0.08	0.08	0.07
204	0.08	0.07	0.08	0.08	0.07
205	0.08	0.08	0.09	0.08	0.08
206	0.09	0.09	0.09	0.09	0.09
207	0.10	0.10	0.10	0.11	0.10
208	0.12	0.12	0.12	0.12	0.11
209	0.13	0.13	0.14	0.14	0.13
210	0.14	0.14	0.15	0.15	0.14
211	0.16	0.16	0.17	0.17	n.a.
212	0.16	0.16	0.18	0.17	0.17
213	0.18	0.18	0.20	0.19	0.17
214	0.19	0.18	0.20	0.20	0.18
215	0.18	0.17	0.19	0.18	0.18
P insertion	n.a.	n.a.	n.a.	n.a.	0.17
E insertion	n.a.	n.a.	n.a.	n.a.	0.17
D insertion	n.a.	n.a.	n.a.	n.a.	0.15
216	0.17	0.16	0.15	0.16	0.14
217	0.17	0.17	0.15	0.17	0.15
218	0.18	0.18	0.16	0.19	0.15
219	0.17	0.16	0.15	0.17	0.13
220	0.13	0.13	0.12	0.13	0.11
221	0.11	0.11	0.11	0.11	0.10
222	0.10	0.10	0.09	0.09	0.10
223	0.08	0.08	0.08	0.08	0.08
224	0.08	0.08	0.08	0.08	0.08
225	0.08	0.08	0.08	0.09	0.08
226	0.09	0.09	0.09	0.09	0.08
227	0.09	0.09	0.10	0.09	0.09
228	0.09	0.08	0.09	0.09	0.08
229	0.09	0.09	0.09	0.10	0.08
230	0.09	0.09	0.09	0.10	0.08
231	0.09	0.09	0.09	0.10	0.08
232	0.09	0.10	0.10	0.10	0.09
233	0.09	0.10	0.10	0.10	0.09
234	0.10	0.10	0.10	0.11	0.10
235	0.09	0.09	0.10	0.10	0.09
236	0.10	0.10	0.10	0.11	0.10
237	0.10	0.10	0.10	0.11	0.09
238	0.09	0.09	0.09	0.10	0.09
239	0.09	0.09	0.10	0.10	0.09
240	0.09	0.09	0.10	0.10	0.09
241	0.10	0.10	0.11	0.11	0.10
242	0.11	0.11	0.12	0.12	0.11
243	0.12	0.12	0.13	0.13	0.12
244	0.13	0.14	0.15	0.15	0.14
245	0.15	0.15	0.17	0.17	0.15

246	0.17	0.17	0.19	0.19	0.17
247	0.20	0.20	0.23	0.22	0.20
248	0.23	0.23	0.28	0.26	0.25
249	0.25	0.27	0.32	0.28	0.30
250	0.25	0.27	0.32	0.27	0.32
251	0.27	0.30	0.33	0.29	0.36
252	0.27	0.30	0.32	0.28	0.37
253	0.24	0.27	0.28	0.26	0.31
254	0.23	0.26	0.25	0.24	0.29
255	0.23	0.24	0.23	0.22	0.28
256	0.23	0.22	0.22	0.22	0.25
257	0.20	0.20	0.21	0.22	0.19
258	0.17	0.17	0.18	0.19	0.16
259	0.16	0.16	0.18	0.18	0.16
260	0.15	0.15	0.17	0.17	0.15
261	0.15	0.15	0.16	0.16	0.15
262	0.14	0.14	0.15	0.15	0.14
263	0.12	0.13	0.13	0.13	0.12
264	0.12	0.12	0.12	0.12	0.12
265	0.10	0.10	0.10	0.11	0.10
266	0.10	0.10	0.10	0.10	0.10
267	0.09	0.09	0.09	0.10	0.09
268	0.09	0.10	0.10	0.10	0.10
269	0.09	0.09	0.09	0.10	0.09
270	0.09	0.09	0.09	0.09	0.09
271	0.10	0.09	0.10	0.10	0.09
272	0.10	0.10	0.10	0.10	0.09
273	0.09	0.09	0.08	0.09	0.09
274	0.09	0.09	0.08	0.09	0.08
275	0.09	0.09	0.08	0.09	0.08
276	0.09	0.08	0.08	0.09	0.08
277	0.09	0.08	0.08	0.09	0.07
278	0.09	0.09	0.08	0.09	0.08
279	0.09	0.09	0.08	0.09	0.09
280	0.10	0.10	0.09	0.10	0.10
281	0.11	0.11	0.10	0.11	0.11
282	0.11	0.11	0.10	0.11	0.11
283	0.10	0.10	0.10	0.10	0.10
284	0.10	0.09	0.09	0.09	0.09
285	0.09	0.09	0.08	0.09	0.08
286	0.10	0.10	0.09	0.09	0.09
287	0.09	0.09	0.09	0.10	0.09
288	0.09	0.08	0.08	0.09	0.08
289	0.09	0.09	0.08	0.09	0.08
290	0.09	0.09	0.08	0.09	0.08
291	0.09	0.09	0.09	0.09	0.08
292	0.10	0.10	0.10	0.10	0.09
293	0.10	0.10	0.10	0.10	0.09
294	0.09	0.09	0.09	0.09	0.09
295	0.09	0.09	0.09	0.09	0.08

296	0.09	0.09	0.09	0.09	0.08
297	0.09	0.08	0.08	0.09	0.08
298	0.09	0.09	0.08	0.09	0.08
299	0.09	0.09	0.09	0.09	0.08
300	0.09	0.09	0.09	0.09	0.08
301	0.09	0.09	0.08	0.09	0.08
302	0.10	0.10	0.09	0.10	0.09
303	0.10	0.11	0.10	0.11	0.09
304	0.10	0.11	0.10	0.11	0.09
305	0.10	0.11	0.10	0.11	0.10
306	0.11	0.10	0.10	0.11	0.10
307	0.12	0.12	0.12	0.13	0.12
308	0.11	0.11	0.11	0.12	0.11
309	0.12	0.11	0.11	0.12	0.11
310	0.11	0.11	0.11	0.12	0.11
311	0.10	0.10	0.10	0.10	0.10
312	0.08	0.09	0.09	0.09	0.09
313	0.08	0.08	0.08	0.08	0.08
314	0.08	0.08	0.08	0.08	0.08
315	0.08	0.08	0.08	0.08	0.08
316	0.08	0.08	0.07	0.08	0.08
317	0.08	0.07	0.07	0.07	0.07
318	0.08	0.07	0.08	0.08	0.08
319	0.08	0.09	0.08	0.09	0.09
320	0.10	0.10	0.09	0.09	0.10
321	0.11	0.11	0.11	0.11	0.11
322	0.10	0.10	0.10	0.10	0.10
323	0.13	0.12	0.13	0.12	0.13
324	0.12	0.11	0.11	0.10	0.11
325	0.11	0.11	0.11	0.10	0.10
326	0.10	0.10	0.09	0.09	0.09
327	0.09	0.09	0.08	0.09	0.09
328	0.09	0.09	0.08	0.09	0.08
329	0.09	0.09	0.08	0.09	0.08
330	0.10	0.10	0.09	0.09	0.09
331	0.12	0.12	0.12	0.12	0.11
332	0.11	0.11	0.11	0.11	0.11
333	0.14	0.14	0.14	0.14	0.14
334	0.12	0.12	0.12	0.13	0.12
335	0.11	0.11	0.11	0.11	0.11
336	0.11	0.10	0.10	0.10	0.09
337	0.13	0.12	0.12	0.12	0.11
338	0.16	0.14	0.14	0.14	0.12
339	0.17	0.16	0.15	0.15	0.13
340	0.15	0.16	0.14	0.14	0.13
341	0.13	0.13	0.13	0.13	0.13
342	0.11	0.11	0.12	0.11	0.11
343	0.13	0.12	0.13	0.13	0.13
344	0.14	0.13	0.13	0.14	0.14
345	0.17	0.17	0.15	0.17	0.18

346	0.14	0.15	0.13	0.16	0.16
347	0.09	0.10	0.09	0.09	0.10
348	0.08	0.09	0.09	0.09	0.09
349	0.08	0.08	0.08	0.08	0.08
350	0.07	0.08	0.08	0.08	0.07
351	0.08	0.08	0.08	0.08	0.08
352	0.08	0.09	0.09	0.09	0.08
353	0.08	0.08	0.08	0.08	0.07
354	0.09	0.09	0.08	0.08	0.08
355	0.08	0.08	0.08	0.08	0.07
356	0.09	0.09	0.09	0.09	0.08
357	0.09	0.09	0.09	0.09	0.08
358	0.09	0.09	0.09	0.09	0.09
359	0.09	0.09	0.09	0.09	0.09
360	0.10	0.09	0.09	0.09	0.09
361	0.10	0.09	0.09	0.09	0.09
362	0.10	0.10	0.10	0.10	0.09
363	0.11	0.10	0.11	0.10	0.09
364	0.12	0.12	0.13	0.12	0.12
365	0.13	0.12	0.14	0.13	0.13
366	0.14	0.13	0.14	0.14	0.13
367	0.14	0.14	0.14	0.15	0.13
368	0.13	0.13	0.12	0.14	0.12
369	0.12	0.12	0.12	0.13	0.12
370	0.13	0.13	0.14	0.14	0.14
371	0.16	0.15	0.16	0.15	0.13
372	0.12	0.13	0.13	0.13	0.13
373	0.10	0.12	0.11	0.11	0.10
374	0.09	0.09	0.09	0.09	0.08
375	0.09	0.09	0.10	0.09	0.08
376	0.10	0.10	0.10	0.09	0.09
377	0.10	0.10	0.11	0.09	0.09
378	0.08	0.08	0.09	0.09	0.08
379	0.07	0.08	0.08	0.08	0.07
380	0.07	0.08	0.08	0.08	0.08
381	0.08	0.09	0.09	0.09	0.08
382	0.07	0.07	0.08	0.08	0.07
383	0.08	0.07	0.07	0.08	0.07
384	0.09	0.08	0.09	0.10	0.08
385	0.09	0.09	0.09	0.10	0.09
386	0.09	0.09	0.09	0.10	0.08
387	0.10	0.09	0.09	0.10	0.09
388	0.11	0.10	0.10	0.11	0.10
389	0.10	0.09	0.10	0.11	0.09
390	0.08	0.08	0.08	0.09	0.08
391	0.08	0.07	0.08	0.08	0.07
392	0.07	0.07	0.08	0.07	0.07
393	0.08	0.08	0.09	0.08	0.08
394	0.08	0.08	0.09	0.09	0.08
395	0.07	0.08	0.08	0.08	0.07

396	0.07	0.07	0.08	0.08	0.07
397	0.08	0.07	0.08	0.08	0.07
398	0.08	0.08	0.07	0.08	0.07
399	0.07	0.08	0.07	0.08	0.07
400	0.06	0.07	0.07	0.07	0.07
401	0.07	0.07	0.07	0.07	0.07
402	0.07	0.07	0.07	0.07	0.07
403	0.07	0.08	0.08	0.08	0.07
404	0.08	0.09	0.08	0.09	0.08
405	0.08	0.09	0.09	0.09	0.08
406	0.08	0.08	0.08	0.08	0.07
407	0.08	0.08	0.08	0.08	0.07
408	0.08	0.08	0.09	0.08	0.08
409	0.08	0.08	0.08	0.08	0.07
410	0.07	0.08	0.08	0.07	0.07
411	0.08	0.08	0.09	0.08	0.07
412	0.10	0.08	0.10	0.10	0.09
413	0.11	0.10	0.12	0.12	0.10
414	0.10	0.09	0.10	0.10	0.09
415	0.09	0.09	0.10	0.10	0.09
416	0.09	0.09	0.10	0.09	0.09
417	0.08	0.08	0.09	0.09	0.08
418	0.07	0.08	0.08	0.08	0.07
419	0.08	0.08	0.08	0.08	0.08
420	0.08	0.08	0.09	0.09	0.08
421	0.08	0.08	0.09	0.09	0.08
422	0.07	0.07	0.08	0.08	0.07
423	0.07	0.07	0.08	0.08	0.07
424	0.07	0.07	0.08	0.08	0.07
425	0.08	0.08	0.08	0.08	0.08
426	0.09	0.08	0.09	0.09	0.09
427	0.10	0.09	0.10	0.10	0.09
428	0.10	0.09	0.10	0.10	0.10
429	0.09	0.08	0.09	0.09	0.09
430	0.07	0.08	0.08	0.08	0.08
431	0.07	0.07	0.07	0.07	0.07
432	0.07	0.07	0.07	0.07	0.07
433	0.07	0.07	0.07	0.07	0.07
434	0.07	0.07	0.07	0.07	0.07
435	0.07	0.07	0.08	0.08	0.07
436	0.08	0.08	0.08	0.08	0.07
437	0.08	0.08	0.09	0.08	0.07
438	0.08	0.09	0.09	0.09	0.08
439	0.09	0.10	0.09	0.09	0.09
440	0.10	0.12	0.12	0.12	0.11
441	0.10	0.11	0.11	0.11	0.11
442	0.09	0.10	0.10	0.09	0.09
443	0.09	0.11	0.11	0.10	0.10
444	0.12	0.13	0.14	0.13	0.13
445	0.15	0.15	0.17	0.16	0.15

446	0.16	0.16	0.18	0.18	0.16
447	0.13	0.14	0.16	0.14	0.14
448	0.10	0.12	0.13	0.11	0.12
449	0.12	0.12	0.12	0.12	0.12
450	0.11	0.12	0.12	0.11	0.11
451	0.09	0.09	0.09	0.09	0.09
452	0.08	0.09	0.09	0.09	0.09
453	0.07	0.08	0.08	0.08	0.08
454	0.08	0.08	0.09	0.09	0.08
455	0.09	0.09	0.10	0.10	0.09
456	0.10	0.11	0.12	0.11	0.11
457	0.10	0.10	0.12	0.11	0.11
458	0.14	0.12	0.15	0.14	0.13
459	0.13	0.12	0.13	0.13	0.12
460	0.11	0.10	0.11	0.11	0.10
461	0.09	0.09	0.10	0.10	0.09
462	0.09	0.09	0.10	0.10	0.08
463	0.09	0.08	0.09	0.09	0.08
464	0.08	0.08	0.09	0.09	0.08
465	0.08	0.08	0.09	0.09	0.08
466	0.09	0.09	0.09	0.09	0.08
467	0.09	0.09	0.10	0.10	0.09
468	0.10	0.10	0.11	0.10	0.10
469	0.11	0.11	0.13	0.12	0.11
470	0.12	0.12	0.14	0.13	0.13
471	0.14	0.14	0.17	0.15	0.15
472	0.14	0.14	0.17	0.15	0.15
473	0.16	0.14	0.18	0.15	0.17
474	0.20	0.17	0.23	0.19	0.20
475	0.25	0.21	0.28	0.23	0.24
476	0.33	0.28	0.38	0.32	0.31
477	0.38	0.32	0.43	0.36	0.34
478	0.36	0.30	0.40	0.34	0.34
479	0.34	0.28	0.36	0.32	0.32
480	0.27	0.24	0.30	0.26	0.28
481	0.35	0.30	0.38	0.34	0.38
482	0.35	0.27	0.34	0.33	0.42
483	0.31	0.25	0.30	0.30	0.38
484	0.28	0.21	0.25	0.26	0.33
485	0.28	0.23	0.26	0.28	0.31
486	0.27	0.24	0.28	0.28	0.29
487	0.24	0.21	0.26	0.23	0.25
488	0.18	0.17	0.20	0.18	0.19
489	0.14	0.14	0.16	0.16	0.16
490	0.12	0.12	0.14	0.13	0.13
491	0.10	0.10	0.12	0.11	0.11
492	0.09	0.09	0.10	0.10	0.10
493	0.09	0.10	0.10	0.10	0.09
494	0.10	0.10	0.11	0.10	0.10
495	0.09	0.10	0.10	0.09	0.09

496	0.10	0.11	0.11	0.11	0.10
497	0.09	0.10	0.10	0.10	0.10
498	0.10	0.11	0.12	0.11	0.11
499	0.12	0.13	0.13	0.13	0.12
500	0.14	0.15	0.15	0.14	0.14
501	0.10	0.12	0.12	0.11	0.11
502	0.10	0.13	0.12	0.12	0.11
503	0.09	0.10	0.11	0.11	0.10
504	0.09	0.10	0.10	0.10	0.09
505	0.08	0.09	0.09	0.09	0.09
506	0.08	0.09	0.09	0.09	0.08
507	0.07	0.08	0.08	0.08	0.07
508	0.07	0.07	0.07	0.07	0.07
509	0.07	0.07	0.07	0.07	0.07
510	0.07	0.07	0.07	0.07	0.07
511	0.07	0.07	0.07	0.07	0.07
512	0.07	0.07	0.07	0.07	0.07
513	0.07	0.07	0.07	0.08	0.07
514	0.07	0.07	0.07	0.08	0.07
515	0.08	0.08	0.08	0.09	0.08
516	0.08	0.08	0.08	0.08	0.08
517	0.08	0.08	0.08	0.08	0.07
518	0.08	0.08	0.08	0.08	0.07
519	0.08	0.09	0.09	0.09	0.08
520	0.08	0.09	0.09	0.08	0.08
521	0.08	0.08	0.08	0.08	0.08
522	0.08	0.08	0.09	0.08	0.08
523	0.09	0.09	0.10	0.09	0.08
524	0.08	0.08	0.08	0.08	0.07
525	0.09	0.08	0.09	0.08	0.08
526	0.10	0.10	0.11	0.10	0.09
527	0.11	0.11	0.12	0.12	0.11
528	0.12	0.12	0.12	0.12	0.11
529	0.13	0.13	0.12	0.13	0.12
530	0.11	0.10	0.10	0.10	0.10
531	0.11	0.10	0.10	0.10	0.10
532	0.12	0.12	0.12	0.12	0.11
533	0.11	0.11	0.11	0.11	0.10
534	0.11	0.11	0.11	0.11	0.10
535	0.10	0.10	0.10	0.10	0.10
536	0.10	0.10	0.10	0.11	0.11
537	0.10	0.10	0.10	0.10	0.10
538	0.09	0.09	0.09	0.09	0.09
539	0.09	0.08	0.09	0.09	0.08
540	0.09	0.09	0.09	0.09	0.08
541	0.08	0.08	0.09	0.08	0.08
542	0.08	0.08	0.08	0.08	0.07
543	0.07	0.08	0.07	0.07	0.07
544	0.08	0.08	0.07	0.08	0.07
545	0.09	0.09	0.08	0.08	0.08

546	0.08	0.08	0.07	0.08	0.07
547	0.08	0.09	0.08	0.09	0.08
548	0.09	0.09	0.09	0.09	0.08
549	0.08	0.08	0.08	0.09	0.08
550	0.08	0.08	0.08	0.08	0.08
551	0.08	0.08	0.08	0.08	0.08
552	0.09	0.08	0.08	0.08	0.08
553	0.09	0.09	0.09	0.09	0.09
554	0.10	0.10	0.10	0.10	0.10
555	0.10	0.10	0.10	0.10	0.09
556	0.14	0.13	0.12	0.12	0.12
557	0.11	0.10	0.10	0.10	0.10
558	0.12	0.12	0.12	0.12	0.12
559	0.09	0.09	0.09	0.10	0.09
560	0.10	0.10	0.10	0.10	0.10
561	0.11	0.11	0.11	0.12	0.12
562	0.10	0.09	0.10	0.10	0.10
563	0.08	0.08	0.08	0.09	0.08
564	0.07	0.08	0.07	0.08	0.07
565	0.07	0.07	0.07	0.07	0.07
566	0.08	0.07	0.08	0.08	0.07
567	0.08	0.08	0.08	0.08	0.07
568	0.08	0.08	0.09	0.08	0.07
569	0.10	0.09	0.09	0.09	0.09
570	0.09	0.08	0.09	0.09	0.08
571	0.09	0.08	0.09	0.09	0.08
572	0.09	0.08	0.09	0.09	0.08
573	0.08	0.08	0.08	0.09	0.07
574	0.08	0.08	0.08	0.08	0.07
575	0.07	0.07	0.07	0.08	0.07
576	0.07	0.07	0.08	0.08	0.07
577	0.08	0.08	0.08	0.08	0.08
578	0.09	0.09	0.09	0.09	0.08
579	0.10	0.10	0.09	0.10	0.09
580	0.11	0.12	0.12	0.12	0.11
581	0.11	0.12	0.11	0.12	0.11
582	0.10	0.10	0.10	0.11	0.10
583	0.11	0.10	0.10	0.11	0.10
584	0.09	0.09	0.09	0.09	0.08
585	0.09	0.08	0.08	0.09	0.08
586	0.08	0.08	0.08	0.08	0.07
587	0.08	0.08	0.08	0.08	0.07
588	0.09	0.08	0.08	0.08	0.08
589	0.08	0.08	0.08	0.08	0.08
590	0.08	0.08	0.08	0.08	0.09
591	0.08	0.08	0.08	0.08	0.08
592	0.08	0.07	0.08	0.08	0.08
593	0.09	0.09	0.08	0.09	0.08
594	0.08	0.08	0.08	0.08	0.08
595	0.08	0.07	0.07	0.07	0.07

596	0.07	0.07	0.08	0.08	0.07
597	0.08	0.08	0.08	0.08	0.08
598	0.08	0.08	0.09	0.09	0.09
599	0.10	0.10	0.10	0.10	0.10
600	0.11	0.11	0.12	0.12	0.11
601	0.12	0.11	0.12	0.13	0.12
602	0.13	0.13	0.13	0.14	0.13
603	0.16	0.15	0.16	0.17	0.17
604	0.15	0.14	0.15	0.16	0.15
605	0.14	0.13	0.14	0.16	0.14
606	0.15	0.14	0.16	0.16	0.15
607	0.13	0.12	0.13	0.13	0.13
608	0.10	0.10	0.11	0.10	0.10
609	0.09	0.09	0.10	0.09	0.09
610	0.08	0.08	0.09	0.08	0.08
611	0.08	0.08	0.08	0.08	0.08
612	0.08	0.08	0.08	0.08	0.08
613	0.08	0.08	0.08	0.09	0.08
614	0.08	0.08	0.09	0.10	0.10
615	0.09	0.09	0.09	0.09	0.09
616	0.10	0.10	0.10	0.10	0.10
617	0.11	0.10	0.11	0.11	0.10
618	0.13	0.12	0.13	0.14	0.12
619	0.15	0.14	0.15	0.17	0.15
620	0.18	0.16	0.18	0.19	0.17
621	0.22	0.20	0.21	0.21	0.21
622	0.26	0.24	0.23	0.22	0.23
623	0.30	0.28	0.25	0.25	0.26
624	0.32	0.27	0.23	0.23	0.23
625	0.31	0.27	0.22	0.20	0.21
626	0.28	0.25	0.26	0.22	0.24
627	0.24	0.23	0.23	0.21	0.20
628	0.20	0.19	0.19	0.18	0.18
629	0.19	0.18	0.17	0.16	0.15
630	0.16	0.18	0.16	0.16	0.14
631	0.15	0.15	0.13	0.14	0.12
632	0.14	0.13	0.12	0.14	0.12
633	0.13	0.12	0.12	0.14	0.12
634	0.11	0.10	0.11	0.12	0.10
635	0.10	0.10	0.11	0.12	0.10
636	0.11	0.11	0.12	0.13	0.11
637	0.14	0.13	0.14	0.15	0.14
638	0.16	0.15	0.17	0.16	0.16
639	0.18	0.17	0.19	0.17	0.17
640	0.17	0.17	0.19	0.18	0.19
641	0.13	0.13	0.13	0.14	0.14
642	0.12	0.11	0.12	0.12	0.11
643	0.11	0.11	0.11	0.11	0.10
644	0.11	0.10	0.10	0.10	0.09
645	0.10	0.10	0.10	0.10	0.09

646	0.11	0.11	0.10	0.11	0.10
647	0.10	0.10	0.10	0.10	0.10
648	0.09	0.09	0.09	0.09	0.09
649	0.09	0.08	0.09	0.09	0.08
650	0.08	0.08	0.09	0.09	0.09
651	0.09	0.09	0.10	0.10	0.09
652	0.10	0.10	0.11	0.11	0.10
653	0.11	0.10	0.11	0.12	0.11
654	0.12	0.11	0.12	0.13	0.11
655	0.12	0.12	0.12	0.13	0.11
656	0.13	0.13	0.13	0.13	0.12
657	0.16	0.16	0.16	0.16	0.15
658	0.15	0.16	0.16	0.16	0.16
659	0.13	0.14	0.15	0.14	0.13
660	0.12	0.13	0.13	0.14	0.13
661	0.12	0.13	0.13	0.14	0.12
662	0.11	0.11	0.11	0.12	0.11
663	0.11	0.11	0.11	0.12	0.11
664	0.09	0.10	0.10	0.11	0.10
665	0.09	0.10	0.10	0.10	0.09
666	0.09	0.10	0.09	0.10	0.09
667	0.09	0.09	0.09	0.09	0.09
668	0.09	0.09	0.09	0.09	0.09
669	0.10	0.09	0.09	0.09	0.09
670	0.09	0.09	0.09	0.09	0.09
671	0.09	0.09	0.09	0.09	0.09
672	0.10	0.10	0.10	0.10	0.10
673	0.11	0.11	0.11	0.12	0.10
674	0.12	0.12	0.12	0.13	0.12
675	0.14	0.13	0.15	0.16	0.14
676	0.16	0.15	0.17	0.19	0.18
677	0.18	0.15	0.20	0.21	0.19
678	0.20	0.17	0.25	0.27	0.23
679	0.23	0.19	0.30	0.33	0.27
680	0.26	0.22	0.37	0.41	0.34
681	0.27	0.24	0.42	0.48	0.37
682	0.26	0.24	0.40	0.46	0.37
683	0.26	0.23	0.38	0.45	0.38
684	0.25	0.24	0.35	0.41	0.37
685	0.23	0.22	0.33	0.35	0.37
686	0.22	0.24	0.34	0.33	0.35
687	0.23	0.23	0.32	0.34	0.32
688	0.23	0.23	0.29	0.30	0.28
689	0.18	0.18	0.22	0.23	0.22
690	0.15	0.15	0.17	0.18	0.17
691	0.12	0.13	0.14	0.15	0.13
692	0.11	0.11	0.11	0.12	0.11
693	0.10	0.10	0.11	0.11	0.10
694	0.10	0.10	0.10	0.10	0.10
695	0.10	0.10	0.10	0.11	0.10

696	0.10	0.10	0.10	0.10	0.10
697	0.11	0.10	0.10	0.10	0.10
698	0.12	0.11	0.11	0.12	0.11
699	0.11	0.11	0.10	0.11	0.11
700	0.13	0.13	0.12	0.14	0.13
701	0.13	0.13	0.13	0.14	0.14
702	0.13	0.13	0.13	0.13	0.14
703	0.12	0.13	0.13	0.13	0.14
704	0.13	0.14	0.14	0.14	0.14
705	0.12	0.12	0.12	0.12	0.12
706	0.11	0.12	0.12	0.13	0.13
707	0.10	0.11	0.10	0.11	0.11
708	0.11	0.11	0.10	0.11	0.11
709	0.11	0.11	0.10	0.11	0.11
710	0.11	0.11	0.10	0.11	0.11
711	0.10	0.11	0.10	0.11	0.11
712	0.09	0.09	0.09	0.09	0.09
713	0.09	0.09	0.09	0.10	0.09
714	0.09	0.09	0.09	0.09	0.09
715	0.09	0.09	0.09	0.10	0.09
716	0.10	0.10	0.10	0.10	0.10
717	0.10	0.10	0.10	0.10	0.10
718	0.09	0.10	0.09	0.10	0.10
719	0.10	0.10	0.10	0.10	0.10
720	0.09	0.09	0.10	0.10	0.09
721	0.10	0.10	0.10	0.11	0.10
722	0.09	0.10	0.09	0.09	0.09
723	0.09	0.10	0.09	0.09	0.09
724	0.09	0.09	0.08	0.08	0.09
725	0.08	0.08	0.08	0.08	0.08
726	0.07	0.08	0.08	0.08	0.08
727	0.07	0.07	0.07	0.07	0.07
728	0.07	0.07	0.07	0.08	0.08
729	0.07	0.07	0.07	0.08	0.07
730	0.07	0.07	0.08	0.08	0.07
731	0.08	0.07	0.08	0.08	0.08
732	0.07	0.07	0.08	0.08	0.08
733	0.07	0.07	0.08	0.07	0.07
734	0.07	0.07	0.07	0.07	0.07
735	0.07	0.07	0.07	0.07	0.07
736	0.07	0.07	0.07	0.07	0.06
737	0.06	0.06	0.07	0.07	0.06
738	0.07	0.07	0.07	0.07	0.07
739	0.07	0.07	0.07	0.08	0.07
740	0.07	0.07	0.07	0.07	0.07
741	0.06	0.06	0.06	0.06	0.06
742	0.07	0.07	0.07	0.07	0.07
743	0.07	0.07	0.07	0.07	0.07
744	0.09	0.09	0.09	0.09	0.10
745	0.10	0.10	0.11	0.11	0.10

746	0.10	0.09	0.09	0.09	0.09
747	0.11	0.10	0.10	0.10	0.11
748	0.10	0.09	0.10	0.10	0.11
749	0.08	0.08	0.08	0.08	0.09
750	0.09	0.08	0.09	0.09	0.10
751	0.09	0.09	0.10	0.10	0.11
752	0.08	0.08	0.09	0.09	0.09
753	0.07	0.07	0.08	0.08	0.08
754	0.08	0.08	0.09	0.09	0.09
755	0.07	0.07	0.09	0.08	0.08
756	0.07	0.07	0.08	0.08	0.07
757	0.08	0.09	0.09	0.10	0.08
758	0.08	0.08	0.09	0.09	0.08
759	0.07	0.07	0.07	0.07	0.07
760	0.07	0.07	0.07	0.07	0.06
761	0.07	0.07	0.07	0.08	0.07
762	0.06	0.06	0.07	0.07	0.07
763	0.06	0.06	0.07	0.07	0.07
764	0.06	0.06	0.07	0.07	0.07
765	0.07	0.07	0.07	0.08	0.07
766	0.07	0.07	0.07	0.08	0.07
767	0.07	0.07	0.07	0.07	0.07
768	0.07	0.07	0.08	0.08	0.07
769	0.08	0.08	0.08	0.08	0.08
770	0.07	0.08	0.07	0.07	0.07
771	0.08	0.09	0.08	0.08	0.08
772	0.09	0.09	0.09	0.09	0.09
773	0.08	0.09	0.08	0.08	0.08
774	0.07	0.08	0.07	0.07	0.07
775	0.08	0.08	0.08	0.08	0.08
776	0.08	0.08	0.08	0.08	0.08
777	0.07	0.08	0.07	0.08	0.07
778	0.08	0.07	0.07	0.08	0.08
779	0.08	0.08	0.08	0.09	0.08
780	0.08	0.07	0.07	0.08	0.08
781	0.08	0.07	0.07	0.07	0.07
782	0.08	0.08	0.08	0.09	0.08
783	0.09	0.09	0.08	0.10	0.09
784	0.10	0.09	0.09	0.10	0.09
785	0.10	0.10	0.10	0.10	0.10
786	0.13	0.13	0.13	0.13	0.12
787	0.11	0.11	0.10	0.11	0.11
788	0.10	0.10	0.09	0.10	0.10
789	0.10	0.10	0.10	0.10	0.10
790	0.12	0.13	0.12	0.12	0.12
791	0.14	0.16	0.13	0.14	0.14
792	0.15	0.17	0.14	0.15	0.14
793	0.17	0.17	0.15	0.16	0.15
794	0.14	0.15	0.14	0.14	0.14
795	0.15	0.16	0.15	0.14	0.14

796	0.13	0.14	0.13	0.13	0.12
797	0.12	0.12	0.11	0.12	0.11
798	0.14	0.14	0.14	0.14	0.13
799	0.13	0.13	0.12	0.13	0.12
800	0.10	0.11	0.10	0.11	0.10
801	0.10	0.12	0.10	0.11	0.11
802	0.10	0.10	0.10	0.10	0.10
803	0.11	0.12	0.11	0.12	0.11
804	0.10	0.11	0.10	0.11	0.10
805	0.09	0.10	0.09	0.10	0.09
806	0.10	0.11	0.10	0.10	0.10
807	0.13	0.13	0.12	0.12	0.12
808	0.19	0.18	0.18	0.17	0.18
809	0.27	0.24	0.26	0.24	0.25
810	0.31	0.28	0.30	0.27	0.28
811	0.26	0.26	0.27	0.24	0.25
812	0.27	0.28	0.28	0.25	0.26
813	0.20	0.21	0.20	0.18	0.20
814	0.15	0.15	0.14	0.14	0.15
815	0.11	0.11	0.11	0.11	0.11
816	0.10	0.10	0.10	0.10	0.10
817	0.11	0.11	0.11	0.11	0.11
818	0.10	0.10	0.10	0.10	0.10
819	0.09	0.09	0.09	0.09	0.09
820	0.10	0.11	0.10	0.11	0.10
821	0.10	0.11	0.10	0.11	0.11
822	0.10	0.10	0.10	0.10	0.10
823	0.10	0.10	0.10	0.10	0.11
824	0.12	0.12	0.12	0.12	0.13
825	0.12	0.12	0.12	0.12	0.13
826	0.12	0.12	0.11	0.11	0.12
827	0.12	0.14	0.12	0.13	0.14
828	0.12	0.15	0.13	0.14	0.14
829	0.12	0.15	0.13	0.14	0.14
830	0.13	0.17	0.14	0.14	0.17
831	0.16	0.22	0.15	0.16	0.18
832	0.17	0.22	0.14	0.15	0.16
833	0.13	0.17	0.11	0.12	0.11
834	0.11	0.14	0.10	0.11	0.10
835	0.11	0.11	0.10	0.10	0.09
836	0.11	0.10	0.10	0.10	0.09
837	0.11	0.09	0.10	0.09	0.09
838	0.13	0.11	0.11	0.11	0.10
839	0.12	0.10	0.11	0.10	0.10
840	0.10	0.09	0.10	0.09	0.09
841	0.12	0.11	0.11	0.10	0.10
842	0.14	0.14	0.13	0.13	0.13
843	0.14	0.14	0.13	0.13	0.14
844	0.14	0.13	0.14	0.12	0.14
845	0.13	0.14	0.16	0.14	0.14

846	0.13	0.13	0.15	0.13	0.14
847	0.12	0.12	0.12	0.10	0.11
848	0.11	0.10	0.11	0.09	0.11
849	0.10	0.09	0.11	0.09	0.10
850	0.10	0.09	0.09	0.09	0.09
851	0.09	0.08	0.08	0.08	0.08
852	0.09	0.08	0.08	0.08	0.08
853	0.08	0.07	0.07	0.07	0.07
854	0.08	0.07	0.07	0.07	0.07
855	0.08	0.08	0.08	0.07	0.07
856	0.08	0.07	0.07	0.07	0.07
857	0.08	0.08	0.07	0.08	0.07
858	0.07	0.07	0.07	0.07	0.07
859	0.07	0.07	0.07	0.08	0.07
860	0.07	0.07	0.07	0.08	0.07
861	0.07	0.08	0.08	0.08	0.08
862	0.08	0.09	0.09	0.09	0.08
863	0.07	0.08	0.08	0.09	0.08
864	0.08	0.08	0.08	0.09	0.08
865	0.08	0.09	0.09	0.09	0.09
866	0.09	0.10	0.10	0.10	0.10
867	0.09	0.10	0.10	0.10	0.10
868	0.10	0.10	0.10	0.10	0.10
869	0.09	0.09	0.09	0.09	0.08
870	0.08	0.08	0.08	0.08	0.08
871	0.09	0.09	0.09	0.09	0.09
872	0.10	0.09	0.09	0.09	0.09
873	0.08	0.08	0.08	0.08	0.08
874	0.08	0.08	0.07	0.08	0.08
875	0.08	0.09	0.08	0.09	0.09
876	0.09	0.09	0.08	0.09	0.08
877	0.08	0.08	0.07	0.08	0.07
878	0.08	0.08	0.08	0.08	0.08
879	0.08	0.09	0.09	0.09	0.08
880	0.09	0.09	0.09	0.09	0.09
881	0.08	0.08	0.08	0.09	0.08
882	0.08	0.08	0.08	0.09	0.09
883	0.08	0.09	0.08	0.09	0.09
884	0.08	0.08	0.08	0.09	0.09
885	0.09	0.09	0.08	0.10	0.09
886	0.09	0.08	0.08	0.09	0.09
887	0.08	0.08	0.08	0.09	0.09
888	0.09	0.09	0.08	0.10	0.09
889	0.10	0.10	0.10	0.11	0.10
890	0.12	0.11	0.11	0.10	0.11
891	0.14	0.13	0.14	0.13	0.13
892	0.12	0.12	0.12	0.11	0.12
893	0.10	0.10	0.10	0.10	0.10
894	0.09	0.09	0.09	0.09	0.09
895	0.09	0.09	0.09	0.09	0.09

896	0.08	0.09	0.08	0.09	0.09
897	0.09	0.09	0.09	0.09	0.09
898	0.09	0.09	0.08	0.09	0.09
899	0.09	0.09	0.09	0.10	0.10
900	0.09	0.08	0.08	0.09	0.09
901	0.08	0.08	0.07	0.08	0.08
902	0.08	0.08	0.08	0.09	0.08
903	0.08	0.08	0.08	0.09	0.08
904	0.07	0.07	0.08	0.08	0.08
905	0.07	0.07	0.07	0.08	0.07
906	0.08	0.07	0.07	0.08	0.07
907	0.08	0.07	0.08	0.08	0.08
908	0.08	0.08	0.08	0.08	0.08
909	0.08	0.08	0.09	0.09	0.08
910	0.09	0.09	0.09	0.10	0.09
911	0.09	0.08	0.09	0.09	0.08
912	0.09	0.08	0.09	0.09	0.08
913	0.09	0.09	0.09	0.09	0.09
914	0.10	0.09	0.10	0.10	0.10
915	0.10	0.09	0.10	0.10	0.09
916	0.09	0.09	0.09	0.10	0.09
917	0.10	0.10	0.10	0.10	0.10
918	0.11	0.10	0.11	0.11	0.11
919	0.11	0.11	0.11	0.11	0.11
920	0.11	0.11	0.11	0.11	0.11
921	0.12	0.12	0.12	0.12	0.12
922	0.12	0.12	0.12	0.12	0.11
923	0.10	0.10	0.10	0.10	0.10
924	0.10	0.11	0.11	0.11	0.10
925	0.11	0.11	0.11	0.12	0.11
926	0.11	0.11	0.11	0.11	0.11
927	0.10	0.10	0.10	0.10	0.10
928	0.10	0.11	0.11	0.11	0.11
929	0.12	0.12	0.11	0.12	0.11
930	0.11	0.12	0.10	0.11	0.11
931	0.11	0.12	0.11	0.11	0.11
932	0.12	0.14	0.12	0.12	0.12
933	0.12	0.14	0.12	0.12	0.12
934	0.11	0.12	0.10	0.11	0.11
935	0.12	0.12	0.12	0.12	0.11
936	0.13	0.14	0.13	0.14	0.13
937	0.13	0.14	0.14	0.13	0.13
938	0.12	0.13	0.13	0.13	0.13
939	0.14	0.15	0.15	0.15	0.14
940	0.16	0.17	0.17	0.17	0.15
941	0.18	0.19	0.20	0.18	0.17
942	0.19	0.20	0.21	0.19	0.18
943	0.16	0.19	0.20	0.17	0.18
944	0.15	0.15	0.17	0.16	0.15
945	0.12	0.12	0.12	0.11	0.11

946	0.14	0.12	0.14	0.13	0.13
947	0.11	0.10	0.12	0.12	0.11
948	0.09	0.08	0.09	0.09	0.09
949	0.10	0.09	0.10	0.10	0.10
950	0.11	0.10	0.11	0.10	0.10
951	0.09	0.08	0.10	0.10	0.09
952	0.09	0.08	0.09	0.09	0.09
953	0.09	0.09	0.10	0.10	0.09
954	0.09	0.09	0.09	0.09	0.09
955	0.09	0.08	0.09	0.08	0.08
956	0.09	0.09	0.09	0.09	0.09
957	0.08	0.08	0.09	0.08	0.08
958	0.08	0.08	0.08	0.08	0.07
959	0.07	0.08	0.08	0.07	0.07
960	0.08	0.08	0.08	0.08	0.07
961	0.07	0.08	0.08	0.08	0.07
962	0.07	0.07	0.07	0.07	0.07
963	0.07	0.08	0.07	0.07	0.07
964	0.07	0.08	0.08	0.08	0.07
965	0.07	0.08	0.07	0.07	0.07
966	0.07	0.08	0.08	0.07	0.07
967	0.08	0.08	0.09	0.07	0.07
968	0.07	0.08	0.08	0.07	0.07
969	0.08	0.07	0.08	0.08	0.08
970	0.07	0.07	0.08	0.08	0.07
971	0.08	0.09	0.09	0.09	0.09
972	0.08	0.08	0.08	0.08	0.08
973	0.08	0.08	0.09	0.08	0.08
974	0.08	0.08	0.09	0.08	0.08
975	0.09	0.08	0.09	0.08	0.08
976	0.08	0.08	0.09	0.08	0.08
977	0.08	0.07	0.08	0.08	0.07
978	0.08	0.08	0.08	0.09	0.08
979	0.07	0.07	0.08	0.08	0.07
980	0.07	0.07	0.07	0.07	0.07
981	0.07	0.07	0.07	0.08	0.08
982	0.07	0.08	0.08	0.08	0.08
983	0.07	0.07	0.07	0.08	0.08
984	0.07	0.07	0.07	0.08	0.07
985	0.08	0.07	0.08	0.09	0.08
986	0.10	0.09	0.10	0.10	0.09
987	0.10	0.10	0.10	0.11	0.10
988	0.09	0.09	0.09	0.09	0.09
989	0.08	0.07	0.08	0.08	0.08
990	0.09	0.08	0.09	0.09	0.09
991	0.09	0.08	0.09	0.09	0.09
992	0.07	0.07	0.08	0.08	0.08
993	0.07	0.07	0.07	0.07	0.07
994	0.07	0.06	0.07	0.07	0.07
995	0.07	0.06	0.07	0.07	0.07

996	0.07	0.06	0.07	0.07	0.07
997	0.06	0.06	0.07	0.06	0.06
998	0.07	0.06	0.07	0.07	0.06
999	0.07	0.06	0.07	0.07	0.06
1000	0.06	0.06	0.06	0.06	0.06
1001	0.06	0.06	0.07	0.06	0.06
1002	0.06	0.06	0.06	0.07	0.06
1003	0.06	0.06	0.06	0.06	0.06
1004	0.06	0.06	0.06	0.06	0.06
1005	0.06	0.06	0.06	0.07	0.06
1006	0.06	0.06	0.07	0.07	0.06
1007	0.06	0.06	0.06	0.07	0.06
1008	0.06	0.06	0.06	0.07	0.06
1009	0.07	0.07	0.07	0.07	0.06
1010	0.07	0.07	0.07	0.07	0.06
1011	0.06	0.06	0.07	0.07	0.06
1012	0.06	0.06	0.07	0.07	0.07
1013	0.06	0.07	0.07	0.07	0.07
1014	0.07	0.07	0.07	0.07	0.07
1015	0.07	0.07	0.07	0.07	0.07
1016	0.07	0.08	0.08	0.07	0.08
1017	0.07	0.07	0.08	0.08	0.08
1018	0.06	0.07	0.07	0.07	0.07
1019	0.07	0.07	0.07	0.07	0.07
1020	0.07	0.07	0.08	0.08	0.08
1021	0.07	0.07	0.07	0.07	0.07
1022	0.06	0.07	0.07	0.07	0.07
1023	0.07	0.07	0.07	0.08	0.08
1024	0.07	0.06	0.07	0.07	0.08
1025	0.06	0.06	0.06	0.07	0.07
1026	0.07	0.06	0.06	0.07	0.07
1027	0.06	0.06	0.06	0.07	0.07
1028	0.06	0.06	0.07	0.07	0.07
1029	0.06	0.06	0.06	0.07	0.07
1030	0.06	0.06	0.06	0.07	0.07
1031	0.06	0.06	0.06	0.07	0.07
1032	0.06	0.06	0.06	0.07	0.06
1033	0.07	0.06	0.07	0.07	0.07
1034	0.07	0.07	0.07	0.07	0.07
1035	0.07	0.07	0.07	0.08	0.07
1036	0.07	0.06	0.07	0.07	0.07
1037	0.07	0.07	0.07	0.08	0.07
1038	0.08	0.08	0.09	0.10	0.10
1039	0.07	0.06	0.07	0.09	0.08
1040	0.08	0.07	0.07	0.09	0.09
1041	0.08	0.08	0.08	0.09	0.09
1042	0.07	0.07	0.07	0.08	0.08
1043	0.07	0.07	0.07	0.07	0.07
1044	0.09	0.08	0.08	0.09	0.08
1045	0.09	0.09	0.09	0.09	0.09

1046	0.10	0.08	0.09	0.09	0.09
1047	0.08	0.07	0.07	0.08	0.07
1048	0.07	0.06	0.07	0.07	0.07
1049	0.07	0.07	0.07	0.07	0.07
1050	0.07	0.07	0.07	0.07	0.07
1051	0.07	0.07	0.07	0.07	0.07
1052	0.07	0.08	0.07	0.08	0.08
1053	0.07	0.07	0.07	0.07	0.07
1054	0.07	0.07	0.07	0.08	0.08
1055	0.08	0.08	0.08	0.08	0.08
1056	0.08	0.09	0.09	0.08	0.09
1057	0.08	0.09	0.09	0.09	0.09
1058	0.07	0.08	0.08	0.08	0.08
1059	0.07	0.07	0.08	0.08	0.08
1060	0.07	0.07	0.07	0.07	0.07
1061	0.07	0.07	0.07	0.08	0.07
1062	0.07	0.07	0.07	0.07	0.07
1063	0.07	0.07	0.07	0.08	0.07
1064	0.07	0.07	0.07	0.08	0.07
1065	0.07	0.07	0.07	0.08	0.07
1066	0.08	0.07	0.07	0.08	0.07
1067	0.08	0.08	0.08	0.08	0.08
1068	0.09	0.09	0.09	0.10	0.09
1069	0.10	0.10	0.09	0.10	0.10
1070	0.10	0.11	0.10	0.11	0.11
1071	0.10	0.11	0.10	0.11	0.11
1072	0.10	0.10	0.10	0.11	0.10
1073	0.10	0.10	0.10	0.11	0.11
1074	0.10	0.10	0.10	0.10	0.10
1075	0.10	0.10	0.10	0.11	0.10
1076	0.10	0.10	0.10	0.11	0.11
1077	0.10	0.10	0.09	0.10	0.10
1078	0.10	0.10	0.10	0.11	0.10
1079	0.10	0.10	0.10	0.11	0.10
1080	0.10	0.10	0.10	0.11	0.10
1081	0.11	0.11	0.11	0.11	0.11
1082	0.12	0.12	0.13	0.13	0.12
1083	0.13	0.14	0.14	0.14	0.14
1084	0.17	0.17	0.17	0.17	0.18
1085	0.16	0.16	0.17	0.17	0.17
1086	0.13	0.14	0.15	0.14	0.14
1087	0.12	0.12	0.12	0.12	0.12
1088	0.10	0.10	0.11	0.11	0.10
1089	0.10	0.09	0.10	0.10	0.09
1090	0.10	0.09	0.10	0.10	0.09
1091	0.10	0.09	0.10	0.10	0.10
1092	0.10	0.09	0.10	0.10	0.10
1093	0.09	0.09	0.09	0.09	0.09
1094	0.09	0.09	0.09	0.09	0.09
1095	0.09	0.09	0.09	0.10	0.09

1096	0.10	0.10	0.09	0.10	0.10
1097	0.11	0.11	0.11	0.12	0.11
1098	0.17	0.16	0.17	0.17	0.16
1099	0.22	0.20	0.21	0.22	0.19
1100	0.22	0.22	0.21	0.23	0.20
1101	0.16	0.15	0.15	0.17	0.15
1102	0.12	0.12	0.11	0.12	0.12
1103	0.10	0.10	0.10	0.11	0.10
1104	0.09	0.09	0.09	0.10	0.09
1105	0.09	0.09	0.09	0.10	0.09
1106	0.09	0.09	0.09	0.09	0.09
1107	0.09	0.10	0.09	0.09	0.09
1108	0.09	0.10	0.09	0.09	0.09
1109	0.09	0.09	0.09	0.09	0.09
1110	0.09	0.09	0.09	0.10	0.09
1111	0.10	0.10	0.10	0.11	0.10
1112	0.11	0.11	0.11	0.12	0.11
1113	0.10	0.10	0.11	0.11	0.10
1114	0.11	0.11	0.11	0.11	0.11
1115	0.11	0.10	0.11	0.11	0.10
1116	0.11	0.11	0.12	0.11	0.11
1117	0.11	0.11	0.13	0.12	0.12
1118	0.12	0.11	0.13	0.12	0.12
1119	0.11	0.10	0.11	0.11	0.10
1120	0.10	0.10	0.10	0.10	0.10
1121	0.11	0.10	0.11	0.11	0.11
1122	0.11	0.11	0.13	0.12	0.12
1123	0.12	0.12	0.13	0.13	0.13
1124	0.16	0.15	0.16	0.16	0.17
1125	0.15	0.15	0.16	0.15	0.15
1126	0.14	0.14	0.15	0.14	0.14
1127	0.17	0.17	0.17	0.17	0.17
1128	0.17	0.17	0.17	0.17	0.17
1129	0.15	0.15	0.14	0.15	0.14
1130	0.16	0.16	0.15	0.15	0.15
1131	0.16	0.15	0.15	0.15	0.15
1132	0.13	0.13	0.13	0.13	0.13
1133	0.13	0.13	0.13	0.14	0.13
1134	0.14	0.14	0.15	0.14	0.14
1135	0.14	0.13	0.15	0.14	0.14
1136	0.14	0.14	0.15	0.14	0.15
1137	0.12	0.14	0.14	0.13	0.15
1138	0.13	0.15	0.16	0.14	0.17
1139	0.17	0.20	0.22	0.18	0.23

Supplementary Table S3. Average Cα-positional deviations in the 5 ns after LA removal

	**Deviation after 5 ns (nm)**				
**Cα atom**	**Wild type**	**Alpha**	**Delta**	**Delta plus**	**Omicron**
1	0.38	0.45	0.60	0.38	0.50

2	0.30	0.35	0.46	0.32	0.41
3	0.28	0.29	0.39	0.29	0.35
4	0.28	0.26	0.33	0.28	0.31
5	0.28	0.24	0.30	0.28	0.32
6	0.27	0.26	0.32	0.28	0.31
7	0.28	0.28	0.34	0.29	0.30
8	0.28	0.29	0.34	0.28	0.28
9	0.28	0.29	0.35	0.28	0.27
10	0.26	0.29	0.34	0.29	0.26
11	0.23	0.26	0.33	0.28	0.25
12	0.23	0.23	0.31	0.27	0.21
13	0.20	0.18	0.28	0.24	0.19
14	0.19	0.18	0.25	0.22	0.18
15	0.18	0.16	0.21	0.19	0.16
16	0.19	0.18	0.22	0.21	0.18
17	0.21	0.20	0.22	0.22	0.19
18	0.21	0.20	0.21	0.20	0.18
19	0.25	0.23	0.23	0.23	0.21
20	0.24	0.23	0.22	0.22	0.21
21	0.21	0.20	0.20	0.20	0.19
22	0.23	0.22	0.20	0.21	0.20
23	0.23	0.21	0.21	0.21	0.21
24	0.21	0.21	0.20	0.21	0.20
25	0.22	0.21	0.22	0.21	0.21
26	0.20	0.20	0.20	0.20	0.20
27	0.17	0.17	0.17	0.17	0.16
28	0.15	0.16	0.15	0.15	0.14
29	0.14	0.14	0.14	0.15	0.13
30	0.13	0.13	0.13	0.14	0.13
31	0.12	0.12	0.11	0.13	0.12
32	0.12	0.12	0.12	0.13	0.11
33	0.11	0.11	0.11	0.12	0.10
34	0.11	0.10	0.10	0.11	0.10
35	0.10	0.10	0.10	0.11	0.10
36	0.10	0.10	0.09	0.10	0.09
37	0.09	0.09	0.09	0.09	0.09
38	0.09	0.09	0.09	0.09	0.09
39	0.10	0.10	0.09	0.10	0.09
40	0.09	0.09	0.09	0.09	0.09
41	0.08	0.09	0.09	0.09	0.09
42	0.08	0.09	0.09	0.09	0.08
43	0.08	0.08	0.09	0.08	0.08
44	0.09	0.09	0.09	0.09	0.08
45	0.10	0.09	0.10	0.10	0.09
46	0.11	0.10	0.11	0.10	0.10
47	0.11	0.10	0.11	0.10	0.10
48	0.11	0.11	0.11	0.11	0.10
49	0.10	0.11	0.10	0.10	0.09
50	0.11	0.11	0.10	0.10	0.09
51	0.11	0.10	0.10	0.11	0.10

52	0.10	0.10	0.09	0.10	0.10
53	0.10	0.10	0.10	0.10	0.09
54	0.09	0.10	0.09	0.10	0.09
55	0.09	0.10	0.09	0.10	0.09
56	0.09	0.10	0.09	0.10	0.09
57	0.10	0.11	0.10	0.11	0.09
58	0.10	0.11	0.10	0.11	0.10
59	0.13	0.13	0.12	0.13	0.11
60	0.14	0.14	0.13	0.14	0.12
61	0.14	0.14	0.14	0.15	0.13
62	0.13	0.13	0.12	0.13	0.12
63	0.13	0.13	0.12	0.13	0.12
64	0.13	0.13	0.12	0.13	0.12
65	0.14	0.13	0.13	0.14	0.13
66	0.15	0.14	0.14	0.15	0.14
67	0.16	0.15	0.15	0.16	0.14
68	0.18	0.17	0.17	0.18	0.16
69	0.20	n.a.	0.20	0.20	n.a.
70	0.23	n.a.	0.24	0.24	n.a.
71	0.31	0.19	0.31	0.31	0.18
72	0.41	0.26	0.39	0.38	0.24
73	0.48	0.32	0.42	0.42	0.32
74	0.45	0.35	0.38	0.39	0.35
75	0.33	0.33	0.29	0.31	0.32
76	0.26	0.24	0.23	0.25	0.24
77	0.20	0.20	0.19	0.20	0.18
78	0.18	0.17	0.17	0.18	0.16
79	0.16	0.15	0.15	0.16	0.14
80	0.15	0.14	0.14	0.15	0.13
81	0.14	0.13	0.13	0.14	0.13
82	0.14	0.13	0.13	0.14	0.13
83	0.13	0.12	0.12	0.13	0.12
84	0.12	0.11	0.12	0.12	0.11
85	0.12	0.11	0.12	0.12	0.11
86	0.10	0.10	0.11	0.11	0.10
87	0.13	0.14	0.14	0.15	0.13
88	0.14	0.15	0.13	0.16	0.14
89	0.10	0.10	0.10	0.10	0.10
90	0.10	0.09	0.09	0.10	0.09
91	0.09	0.09	0.09	0.10	0.09
92	0.10	0.09	0.09	0.10	0.09
93	0.10	0.10	0.10	0.11	0.10
94	0.12	0.12	0.11	0.12	0.11
95	0.13	0.13	0.12	0.14	0.12
96	0.14	0.14	0.14	0.15	0.13
97	0.15	0.16	0.16	0.17	0.15
98	0.16	0.17	0.16	0.17	0.16
99	0.15	0.15	0.15	0.15	0.14
100	0.14	0.14	0.14	0.14	0.13
101	0.13	0.12	0.12	0.13	0.12

102	0.12	0.12	0.12	0.13	0.12
103	0.12	0.11	0.12	0.12	0.11
104	0.11	0.10	0.10	0.11	0.10
105	0.11	0.10	0.11	0.11	0.10
106	0.10	0.10	0.10	0.10	0.10
107	0.11	0.10	0.11	0.11	0.10
108	0.12	0.12	0.14	0.13	0.12
109	0.14	0.14	0.15	0.15	0.13
110	0.13	0.12	0.14	0.14	0.12
111	0.15	0.14	0.16	0.16	0.13
112	0.16	0.15	0.18	0.17	0.15
113	0.17	0.16	0.19	0.18	0.16
114	0.14	0.13	0.16	0.15	0.13
115	0.12	0.12	0.14	0.14	0.12
116	0.11	0.10	0.12	0.12	0.10
117	0.11	0.10	0.10	0.11	0.10
118	0.11	0.10	0.11	0.11	0.10
119	0.11	0.10	0.11	0.11	0.10
120	0.12	0.12	0.12	0.12	0.11
121	0.14	0.14	0.14	0.14	0.13
122	0.16	0.16	0.16	0.15	0.15
123	0.20	0.19	0.20	0.20	0.19
124	0.19	0.19	0.20	0.20	0.18
125	0.17	0.16	0.17	0.17	0.15
126	0.14	0.14	0.14	0.15	0.13
127	0.13	0.12	0.14	0.14	0.12
128	0.12	0.11	0.12	0.12	0.11
129	0.12	0.11	0.12	0.13	0.11
130	0.12	0.11	0.12	0.12	0.11
131	0.13	0.12	0.14	0.14	0.12
132	0.14	0.12	0.15	0.14	0.12
133	0.14	0.12	0.15	0.14	0.12
134	0.15	0.14	0.17	0.16	0.14
135	0.14	0.13	0.17	0.15	0.13
136	0.15	0.14	0.18	0.16	0.14
137	0.16	0.15	0.19	0.17	0.15
138	0.15	0.14	0.17	0.15	0.15
139	0.14	0.13	0.15	0.14	0.13
140	0.15	0.13	0.15	0.14	0.13
141	0.15	0.14	0.15	0.15	0.14
142	0.18	0.16	0.19	0.17	0.16
143	0.18	0.18	0.20	0.18	n.a.
144	0.20	n.a.	0.23	0.21	n.a.
145	0.23	0.21	0.27	0.24	n.a.
146	0.28	0.24	0.33	0.28	0.18
147	0.33	0.29	0.41	0.33	0.21
148	0.35	0.33	0.45	0.36	0.23
149	0.32	0.31	0.41	0.34	0.25
150	0.32	0.31	0.39	0.33	0.29
151	0.26	0.29	0.32	0.27	0.25

152	0.22	0.25	0.26	0.23	0.23
153	0.21	0.22	0.23	0.21	0.20
154	0.19	0.19	0.19	0.19	0.18
155	0.20	0.20	0.19	0.20	0.19
156	0.18	0.18	n.a.	n.a.	0.17
157	0.17	0.15	n.a.	n.a.	0.15
158	0.16	0.14	n.a.	0.19	0.14
159	0.14	0.13	0.18	0.17	0.13
160	0.15	0.13	0.18	0.17	0.14
161	0.17	0.15	0.21	0.16	0.16
162	0.17	0.15	0.20	0.18	0.16
163	0.16	0.14	0.19	0.17	0.15
164	0.16	0.15	0.20	0.19	0.15
165	0.15	0.15	0.18	0.17	0.15
166	0.14	0.13	0.16	0.16	0.14
167	0.14	0.13	0.16	0.15	0.14
168	0.13	0.13	0.14	0.14	0.12
169	0.14	0.13	0.14	0.15	0.13
170	0.14	0.14	0.14	0.15	0.14
171	0.16	0.15	0.15	0.17	0.14
172	0.18	0.17	0.17	0.18	0.16
173	0.20	0.21	0.20	0.22	0.18
174	0.20	0.20	0.20	0.21	0.18
175	0.21	0.21	0.21	0.22	0.18
176	0.23	0.23	0.22	0.23	0.20
177	0.20	0.21	0.20	0.21	0.19
178	0.20	0.22	0.22	0.21	0.20
179	0.20	0.23	0.26	0.21	0.21
180	0.22	0.24	0.26	0.23	0.23
181	0.24	0.26	0.25	0.26	0.25
182	0.25	0.26	0.25	0.26	0.24
183	0.25	0.26	0.26	0.26	0.25
184	0.23	0.23	0.22	0.24	0.23
185	0.19	0.19	0.19	0.20	0.19
186	0.16	0.16	0.15	0.17	0.16
187	0.17	0.16	0.16	0.17	0.15
188	0.14	0.14	0.14	0.15	0.13
189	0.12	0.12	0.12	0.13	0.12
190	0.11	0.11	0.10	0.12	0.11
191	0.10	0.10	0.09	0.10	0.10
192	0.09	0.09	0.09	0.09	0.09
193	0.09	0.08	0.08	0.09	0.08
194	0.09	0.08	0.09	0.09	0.08
195	0.09	0.09	0.09	0.10	0.09
196	0.10	0.10	0.10	0.10	0.09
197	0.12	0.12	0.11	0.13	0.11
198	0.14	0.15	0.13	0.15	0.13
199	0.13	0.13	0.12	0.14	0.11
200	0.10	0.10	0.10	0.11	0.09
201	0.09	0.09	0.09	0.10	0.09

202	0.09	0.09	0.09	0.09	0.09
203	0.09	0.08	0.08	0.09	0.08
204	0.09	0.08	0.08	0.09	0.08
205	0.09	0.09	0.09	0.10	0.09
206	0.11	0.10	0.10	0.11	0.10
207	0.12	0.12	0.12	0.13	0.11
208	0.14	0.13	0.14	0.14	0.13
209	0.16	0.16	0.16	0.17	0.15
210	0.17	0.17	0.16	0.17	0.16
211	0.20	0.19	0.20	0.20	n.a.
212	0.19	0.19	0.20	0.20	0.20
213	0.22	0.21	0.22	0.24	0.20
214	0.23	0.22	0.23	0.24	0.22
215	0.21	0.20	0.21	0.23	0.21
P insertion	n.a.	n.a.	n.a.	n.a.	0.20
E insertion	n.a.	n.a.	n.a.	n.a.	0.20
D insertion	n.a.	n.a.	n.a.	n.a.	0.18
216	0.20	0.18	0.18	0.20	0.16
217	0.21	0.20	0.19	0.22	0.17
218	0.21	0.19	0.20	0.24	0.18
219	0.19	0.18	0.17	0.21	0.16
220	0.14	0.14	0.14	0.15	0.13
221	0.13	0.12	0.13	0.14	0.12
222	0.10	0.10	0.10	0.11	0.10
223	0.10	0.09	0.09	0.09	0.09
224	0.10	0.09	0.09	0.10	0.09
225	0.10	0.09	0.09	0.10	0.09
226	0.10	0.10	0.10	0.10	0.09
227	0.10	0.10	0.10	0.11	0.10
228	0.10	0.10	0.10	0.11	0.09
229	0.10	0.10	0.10	0.11	0.10
230	0.11	0.10	0.10	0.12	0.10
231	0.11	0.10	0.10	0.12	0.10
232	0.12	0.11	0.11	0.13	0.11
233	0.11	0.11	0.11	0.12	0.11
234	0.12	0.13	0.13	0.13	0.11
235	0.10	0.11	0.12	0.11	0.10
236	0.11	0.11	0.12	0.12	0.11
237	0.12	0.11	0.12	0.12	0.11
238	0.11	0.10	0.11	0.11	0.10
239	0.11	0.11	0.11	0.11	0.10
240	0.11	0.11	0.11	0.11	0.11
241	0.12	0.11	0.12	0.12	0.11
242	0.14	0.12	0.13	0.13	0.12
243	0.15	0.14	0.15	0.15	0.14
244	0.17	0.16	0.17	0.17	0.15
245	0.18	0.19	0.19	0.18	0.18
246	0.21	0.21	0.22	0.20	0.20
247	0.24	0.25	0.28	0.24	0.24
248	0.29	0.29	0.35	0.28	0.31

249	0.31	0.35	0.41	0.31	0.38
250	0.31	0.36	0.42	0.29	0.41
251	0.34	0.40	0.45	0.32	0.45
252	0.34	0.40	0.44	0.32	0.45
253	0.32	0.35	0.36	0.28	0.38
254	0.31	0.33	0.31	0.27	0.35
255	0.31	0.31	0.28	0.25	0.33
256	0.29	0.28	0.26	0.24	0.29
257	0.25	0.24	0.24	0.24	0.22
258	0.21	0.21	0.21	0.21	0.19
259	0.20	0.20	0.20	0.20	0.18
260	0.19	0.18	0.19	0.19	0.17
261	0.19	0.18	0.18	0.19	0.17
262	0.17	0.16	0.16	0.17	0.16
263	0.15	0.14	0.14	0.15	0.14
264	0.14	0.13	0.13	0.14	0.13
265	0.12	0.12	0.11	0.13	0.12
266	0.12	0.11	0.11	0.12	0.11
267	0.11	0.11	0.10	0.11	0.10
268	0.11	0.11	0.10	0.11	0.10
269	0.10	0.10	0.10	0.11	0.10
270	0.10	0.10	0.10	0.10	0.09
271	0.10	0.10	0.10	0.11	0.10
272	0.10	0.11	0.10	0.11	0.10
273	0.10	0.10	0.09	0.10	0.09
274	0.10	0.10	0.09	0.10	0.09
275	0.10	0.10	0.09	0.10	0.09
276	0.10	0.10	0.09	0.10	0.09
277	0.10	0.09	0.09	0.09	0.09
278	0.10	0.10	0.10	0.10	0.09
279	0.10	0.09	0.10	0.10	0.09
280	0.11	0.10	0.11	0.11	0.10
281	0.12	0.11	0.12	0.12	0.11
282	0.12	0.12	0.12	0.12	0.10
283	0.11	0.11	0.11	0.11	0.10
284	0.10	0.10	0.10	0.10	0.09
285	0.10	0.10	0.09	0.10	0.09
286	0.10	0.10	0.10	0.10	0.10
287	0.10	0.10	0.10	0.10	0.09
288	0.10	0.09	0.09	0.10	0.09
289	0.10	0.10	0.09	0.10	0.09
290	0.10	0.09	0.09	0.10	0.09
291	0.10	0.10	0.09	0.10	0.09
292	0.11	0.11	0.10	0.11	0.10
293	0.11	0.10	0.11	0.11	0.10
294	0.10	0.10	0.10	0.11	0.10
295	0.10	0.10	0.10	0.11	0.09
296	0.10	0.10	0.10	0.10	0.09
297	0.10	0.10	0.09	0.10	0.09
298	0.10	0.10	0.09	0.10	0.09

299	0.10	0.10	0.10	0.10	0.09
300	0.10	0.10	0.09	0.10	0.09
301	0.10	0.10	0.09	0.10	0.09
302	0.11	0.12	0.10	0.12	0.10
303	0.11	0.12	0.11	0.12	0.11
304	0.12	0.12	0.11	0.12	0.10
305	0.12	0.12	0.11	0.12	0.11
306	0.12	0.11	0.11	0.12	0.11
307	0.13	0.13	0.13	0.14	0.13
308	0.12	0.12	0.12	0.13	0.12
309	0.13	0.14	0.12	0.14	0.12
310	0.12	0.12	0.12	0.13	0.12
311	0.11	0.11	0.11	0.12	0.11
312	0.10	0.10	0.09	0.10	0.10
313	0.09	0.09	0.09	0.10	0.09
314	0.09	0.09	0.09	0.09	0.09
315	0.09	0.09	0.09	0.10	0.09
316	0.09	0.09	0.09	0.09	0.09
317	0.08	0.08	0.08	0.08	0.08
318	0.09	0.09	0.09	0.09	0.09
319	0.10	0.10	0.10	0.10	0.10
320	0.11	0.11	0.11	0.11	0.12
321	0.12	0.12	0.12	0.13	0.12
322	0.11	0.12	0.11	0.12	0.11
323	0.15	0.14	0.14	0.15	0.13
324	0.13	0.13	0.13	0.13	0.12
325	0.12	0.12	0.12	0.12	0.11
326	0.10	0.11	0.10	0.11	0.10
327	0.09	0.10	0.10	0.10	0.09
328	0.09	0.10	0.10	0.10	0.09
329	0.10	0.09	0.10	0.10	0.09
330	0.10	0.10	0.11	0.11	0.10
331	0.13	0.13	0.14	0.13	0.12
332	0.13	0.12	0.13	0.12	0.11
333	0.16	0.16	0.16	0.15	0.15
334	0.14	0.14	0.15	0.14	0.13
335	0.13	0.12	0.14	0.13	0.12
336	0.12	0.11	0.12	0.12	0.11
337	0.14	0.13	0.13	0.14	0.12
338	0.18	0.17	0.15	0.17	0.15
339	0.20	0.18	0.17	0.17	0.15
340	0.18	0.17	0.17	0.17	0.15
341	0.14	0.14	0.13	0.15	0.16
342	0.12	0.12	0.11	0.12	0.14
343	0.14	0.14	0.13	0.13	0.15
344	0.15	0.15	0.14	0.14	0.16
345	0.20	0.19	0.16	0.19	0.19
346	0.20	0.16	0.15	0.18	0.17
347	0.11	0.10	0.11	0.12	0.11
348	0.10	0.09	0.10	0.10	0.10

349	0.09	0.09	0.09	0.10	0.09
350	0.09	0.08	0.08	0.09	0.09
351	0.10	0.09	0.09	0.10	0.10
352	0.10	0.09	0.10	0.10	0.10
353	0.10	0.08	0.09	0.10	0.09
354	0.11	0.09	0.09	0.10	0.10
355	0.10	0.09	0.09	0.10	0.09
356	0.10	0.09	0.09	0.11	0.10
357	0.10	0.09	0.10	0.11	0.10
358	0.10	0.09	0.10	0.10	0.10
359	0.10	0.09	0.10	0.10	0.09
360	0.11	0.10	0.11	0.10	0.10
361	0.11	0.10	0.11	0.10	0.10
362	0.11	0.11	0.11	0.11	0.10
363	0.12	0.12	0.12	0.12	0.11
364	0.14	0.14	0.15	0.14	0.14
365	0.16	0.15	0.17	0.17	0.16
366	0.17	0.16	0.18	0.18	0.16
367	0.17	0.17	0.18	0.20	0.17
368	0.16	0.16	0.16	0.19	0.15
369	0.14	0.13	0.15	0.18	0.14
370	0.16	0.16	0.18	0.19	0.17
371	0.19	0.16	0.20	0.21	0.15
372	0.14	0.15	0.16	0.18	0.15
373	0.11	0.13	0.13	0.15	0.11
374	0.10	0.10	0.10	0.11	0.10
375	0.10	0.10	0.11	0.10	0.10
376	0.10	0.10	0.11	0.11	0.10
377	0.10	0.10	0.11	0.11	0.10
378	0.09	0.09	0.09	0.10	0.09
379	0.08	0.08	0.09	0.09	0.08
380	0.09	0.08	0.09	0.10	0.08
381	0.09	0.09	0.09	0.10	0.09
382	0.08	0.08	0.08	0.09	0.08
383	0.08	0.09	0.09	0.09	0.08
384	0.10	0.09	0.10	0.10	0.10
385	0.10	0.10	0.11	0.11	0.10
386	0.10	0.09	0.10	0.11	0.09
387	0.11	0.10	0.11	0.11	0.10
388	0.12	0.12	0.13	0.13	0.11
389	0.11	0.10	0.12	0.12	0.10
390	0.09	0.09	0.09	0.09	0.09
391	0.09	0.08	0.09	0.09	0.08
392	0.09	0.08	0.09	0.09	0.08
393	0.09	0.09	0.10	0.10	0.09
394	0.10	0.10	0.11	0.10	0.10
395	0.09	0.08	0.09	0.09	0.09
396	0.08	0.08	0.08	0.09	0.08
397	0.09	0.08	0.09	0.09	0.08
398	0.09	0.08	0.08	0.09	0.08

399	0.09	0.09	0.09	0.09	0.08
400	0.08	0.07	0.08	0.08	0.08
401	0.08	0.07	0.08	0.08	0.08
402	0.08	0.07	0.08	0.09	0.08
403	0.08	0.08	0.09	0.09	0.08
404	0.09	0.09	0.10	0.10	0.09
405	0.09	0.09	0.10	0.11	0.09
406	0.09	0.09	0.09	0.10	0.09
407	0.09	0.09	0.09	0.09	0.08
408	0.09	0.09	0.09	0.10	0.09
409	0.08	0.08	0.09	0.09	0.08
410	0.08	0.07	0.08	0.09	0.08
411	0.09	0.08	0.09	0.09	0.09
412	0.10	0.10	0.10	0.11	0.10
413	0.12	0.11	0.12	0.13	0.12
414	0.10	0.10	0.11	0.12	0.11
415	0.10	0.10	0.11	0.11	0.10
416	0.10	0.09	0.11	0.11	0.10
417	0.09	0.09	0.10	0.11	0.09
418	0.08	0.08	0.09	0.09	0.08
419	0.09	0.08	0.09	0.10	0.08
420	0.10	0.09	0.10	0.11	0.10
421	0.09	0.09	0.09	0.10	0.09
422	0.08	0.08	0.09	0.09	0.08
423	0.08	0.07	0.09	0.09	0.08
424	0.09	0.08	0.09	0.09	0.08
425	0.09	0.08	0.09	0.09	0.08
426	0.10	0.09	0.10	0.10	0.09
427	0.11	0.10	0.11	0.12	0.10
428	0.11	0.10	0.11	0.12	0.11
429	0.10	0.09	0.10	0.10	0.09
430	0.08	0.08	0.09	0.10	0.09
431	0.08	0.08	0.09	0.09	0.08
432	0.08	0.07	0.08	0.09	0.08
433	0.08	0.07	0.08	0.08	0.08
434	0.08	0.08	0.08	0.09	0.08
435	0.08	0.08	0.09	0.09	0.08
436	0.09	0.09	0.09	0.10	0.09
437	0.09	0.09	0.10	0.10	0.09
438	0.09	0.09	0.10	0.10	0.10
439	0.10	0.10	0.11	0.11	0.10
440	0.12	0.13	0.13	0.13	0.13
441	0.12	0.12	0.13	0.12	0.12
442	0.10	0.11	0.11	0.11	0.11
443	0.11	0.12	0.12	0.12	0.12
444	0.14	0.15	0.17	0.15	0.14
445	0.17	0.18	0.21	0.18	0.16
446	0.18	0.19	0.22	0.20	0.18
447	0.15	0.16	0.20	0.17	0.16
448	0.12	0.14	0.17	0.15	0.14

449	0.13	0.14	0.15	0.15	0.14
450	0.12	0.13	0.14	0.14	0.13
451	0.10	0.10	0.11	0.11	0.10
452	0.10	0.10	0.10	0.11	0.10
453	0.09	0.09	0.09	0.10	0.09
454	0.09	0.09	0.10	0.10	0.09
455	0.11	0.10	0.11	0.12	0.10
456	0.13	0.12	0.14	0.14	0.12
457	0.13	0.12	0.14	0.14	0.12
458	0.16	0.15	0.17	0.17	0.14
459	0.15	0.14	0.16	0.15	0.13
460	0.13	0.12	0.14	0.13	0.11
461	0.10	0.10	0.11	0.11	0.10
462	0.10	0.10	0.11	0.11	0.10
463	0.10	0.09	0.10	0.10	0.09
464	0.10	0.09	0.09	0.10	0.09
465	0.10	0.09	0.10	0.10	0.09
466	0.10	0.09	0.10	0.11	0.10
467	0.11	0.10	0.11	0.11	0.10
468	0.12	0.12	0.12	0.12	0.12
469	0.13	0.13	0.13	0.14	0.14
470	0.15	0.14	0.15	0.16	0.16
471	0.17	0.16	0.18	0.18	0.18
472	0.17	0.16	0.19	0.18	0.18
473	0.18	0.17	0.20	0.20	0.19
474	0.23	0.21	0.25	0.24	0.23
475	0.29	0.26	0.30	0.28	0.28
476	0.38	0.34	0.42	0.39	0.35
477	0.43	0.38	0.47	0.46	0.41
478	0.39	0.36	0.44	0.43	0.39
479	0.36	0.34	0.40	0.41	0.37
480	0.31	0.29	0.34	0.34	0.31
481	0.41	0.35	0.41	0.44	0.42
482	0.41	0.34	0.39	0.44	0.49
483	0.37	0.30	0.34	0.38	0.45
484	0.33	0.25	0.27	0.32	0.37
485	0.34	0.28	0.29	0.34	0.35
486	0.32	0.30	0.31	0.33	0.33
487	0.28	0.26	0.29	0.28	0.28
488	0.21	0.20	0.23	0.23	0.21
489	0.17	0.16	0.19	0.18	0.18
490	0.14	0.13	0.16	0.15	0.15
491	0.12	0.12	0.13	0.13	0.12
492	0.10	0.10	0.12	0.12	0.11
493	0.10	0.10	0.12	0.12	0.11
494	0.10	0.11	0.13	0.12	0.11
495	0.11	0.11	0.12	0.11	0.10
496	0.12	0.12	0.13	0.13	0.12
497	0.10	0.11	0.12	0.11	0.10
498	0.12	0.13	0.14	0.13	0.12

499	0.14	0.15	0.16	0.15	0.14
500	0.15	0.18	0.17	0.16	0.16
501	0.12	0.15	0.15	0.14	0.13
502	0.12	0.14	0.14	0.14	0.14
503	0.11	0.12	0.13	0.12	0.12
504	0.10	0.11	0.12	0.12	0.10
505	0.10	0.10	0.11	0.11	0.10
506	0.08	0.09	0.10	0.10	0.09
507	0.08	0.08	0.09	0.09	0.08
508	0.08	0.08	0.09	0.09	0.08
509	0.08	0.07	0.08	0.09	0.08
510	0.07	0.07	0.08	0.09	0.08
511	0.08	0.07	0.08	0.09	0.08
512	0.08	0.07	0.08	0.09	0.08
513	0.08	0.08	0.08	0.09	0.08
514	0.09	0.08	0.08	0.09	0.08
515	0.09	0.09	0.09	0.09	0.09
516	0.09	0.09	0.10	0.09	0.09
517	0.09	0.09	0.09	0.09	0.08
518	0.09	0.10	0.09	0.09	0.09
519	0.09	0.10	0.10	0.09	0.09
520	0.09	0.09	0.10	0.09	0.09
521	0.09	0.09	0.10	0.09	0.09
522	0.09	0.09	0.10	0.10	0.09
523	0.10	0.10	0.11	0.11	0.10
524	0.09	0.09	0.10	0.10	0.09
525	0.10	0.09	0.10	0.10	0.09
526	0.12	0.11	0.12	0.12	0.10
527	0.13	0.13	0.14	0.14	0.12
528	0.14	0.14	0.14	0.14	0.13
529	0.14	0.14	0.14	0.15	0.13
530	0.11	0.12	0.12	0.12	0.11
531	0.11	0.12	0.11	0.12	0.11
532	0.13	0.13	0.13	0.13	0.12
533	0.12	0.12	0.12	0.12	0.11
534	0.12	0.12	0.12	0.13	0.11
535	0.11	0.12	0.11	0.12	0.10
536	0.11	0.12	0.12	0.12	0.11
537	0.12	0.12	0.12	0.12	0.10
538	0.10	0.10	0.10	0.11	0.09
539	0.10	0.10	0.10	0.10	0.09
540	0.10	0.10	0.10	0.10	0.09
541	0.09	0.09	0.09	0.09	0.08
542	0.09	0.09	0.09	0.09	0.08
543	0.08	0.09	0.09	0.09	0.08
544	0.09	0.09	0.09	0.09	0.08
545	0.10	0.10	0.10	0.09	0.09
546	0.09	0.09	0.09	0.09	0.08
547	0.09	0.10	0.10	0.10	0.09
548	0.10	0.10	0.09	0.10	0.08

549	0.09	0.10	0.09	0.10	0.08
550	0.09	0.10	0.09	0.10	0.08
551	0.09	0.10	0.10	0.10	0.08
552	0.09	0.09	0.10	0.10	0.09
553	0.10	0.10	0.10	0.10	0.09
554	0.11	0.11	0.11	0.11	0.11
555	0.11	0.11	0.11	0.11	0.10
556	0.15	0.15	0.14	0.14	0.13
557	0.12	0.12	0.11	0.11	0.11
558	0.13	0.13	0.13	0.14	0.13
559	0.10	0.10	0.11	0.10	0.10
560	0.11	0.11	0.12	0.11	0.11
561	0.12	0.12	0.13	0.12	0.13
562	0.11	0.11	0.11	0.11	0.11
563	0.09	0.09	0.09	0.09	0.09
564	0.08	0.08	0.09	0.09	0.08
565	0.08	0.08	0.08	0.08	0.08
566	0.08	0.08	0.09	0.09	0.08
567	0.08	0.08	0.09	0.09	0.08
568	0.09	0.10	0.10	0.09	0.08
569	0.10	0.11	0.11	0.10	0.09
570	0.10	0.10	0.11	0.10	0.09
571	0.10	0.10	0.10	0.09	0.09
572	0.10	0.10	0.10	0.10	0.09
573	0.09	0.10	0.09	0.09	0.08
574	0.09	0.09	0.09	0.09	0.08
575	0.09	0.08	0.09	0.09	0.08
576	0.08	0.08	0.09	0.09	0.08
577	0.09	0.09	0.10	0.09	0.09
578	0.09	0.09	0.10	0.10	0.09
579	0.10	0.11	0.11	0.11	0.10
580	0.13	0.13	0.13	0.13	0.12
581	0.12	0.13	0.13	0.13	0.12
582	0.11	0.12	0.12	0.12	0.12
583	0.11	0.12	0.12	0.12	0.12
584	0.09	0.09	0.10	0.10	0.09
585	0.09	0.09	0.10	0.10	0.09
586	0.09	0.09	0.09	0.09	0.08
587	0.09	0.09	0.09	0.09	0.08
588	0.10	0.09	0.09	0.09	0.09
589	0.10	0.09	0.09	0.09	0.08
590	0.09	0.10	0.09	0.10	0.09
591	0.09	0.09	0.09	0.10	0.09
592	0.10	0.09	0.09	0.09	0.09
593	0.09	0.10	0.10	0.10	0.10
594	0.09	0.09	0.09	0.09	0.09
595	0.08	0.08	0.08	0.09	0.08
596	0.08	0.08	0.09	0.09	0.09
597	0.08	0.09	0.09	0.09	0.09
598	0.09	0.10	0.10	0.10	0.10

599	0.11	0.11	0.10	0.12	0.11
600	0.12	0.13	0.12	0.14	0.13
601	0.13	0.13	0.13	0.14	0.13
602	0.14	0.14	0.14	0.17	0.15
603	0.17	0.17	0.17	0.20	0.19
604	0.16	0.16	0.16	0.18	0.17
605	0.15	0.16	0.15	0.17	0.16
606	0.16	0.17	0.17	0.19	0.18
607	0.14	0.14	0.14	0.15	0.15
608	0.11	0.11	0.11	0.12	0.11
609	0.10	0.10	0.10	0.10	0.10
610	0.09	0.09	0.09	0.09	0.09
611	0.09	0.09	0.08	0.09	0.09
612	0.09	0.09	0.09	0.09	0.09
613	0.09	0.10	0.09	0.10	0.09
614	0.10	0.10	0.11	0.13	0.11
615	0.10	0.10	0.10	0.12	0.10
616	0.12	0.12	0.11	0.12	0.12
617	0.12	0.13	0.12	0.13	0.12
618	0.15	0.16	0.14	0.16	0.15
619	0.18	0.18	0.18	0.21	0.20
620	0.22	0.21	0.21	0.22	0.23
621	0.28	0.28	0.25	0.26	0.28
622	0.33	0.33	0.28	0.26	0.31
623	0.38	0.39	0.30	0.30	0.35
624	0.41	0.40	0.27	0.28	0.31
625	0.41	0.41	0.26	0.24	0.28
626	0.35	0.36	0.29	0.27	0.31
627	0.30	0.31	0.27	0.25	0.26
628	0.25	0.25	0.23	0.23	0.23
629	0.24	0.23	0.20	0.20	0.19
630	0.20	0.21	0.19	0.19	0.18
631	0.18	0.18	0.15	0.17	0.15
632	0.16	0.15	0.15	0.16	0.14
633	0.14	0.14	0.15	0.16	0.15
634	0.12	0.12	0.13	0.14	0.13
635	0.12	0.11	0.13	0.14	0.12
636	0.13	0.12	0.14	0.15	0.13
637	0.15	0.15	0.17	0.18	0.16
638	0.18	0.17	0.21	0.21	0.20
639	0.20	0.19	0.22	0.21	0.21
640	0.20	0.19	0.21	0.21	0.21
641	0.15	0.15	0.15	0.16	0.16
642	0.14	0.12	0.13	0.14	0.14
643	0.12	0.12	0.12	0.13	0.12
644	0.12	0.12	0.11	0.12	0.11
645	0.11	0.12	0.11	0.11	0.11
646	0.12	0.14	0.11	0.12	0.12
647	0.11	0.13	0.11	0.12	0.12
648	0.10	0.11	0.10	0.10	0.10

649	0.09	0.10	0.10	0.10	0.10
650	0.09	0.10	0.10	0.10	0.10
651	0.10	0.10	0.10	0.11	0.11
652	0.11	0.11	0.12	0.12	0.12
653	0.11	0.12	0.12	0.12	0.12
654	0.13	0.13	0.14	0.14	0.12
655	0.13	0.14	0.14	0.14	0.13
656	0.14	0.15	0.14	0.15	0.14
657	0.17	0.19	0.18	0.19	0.16
658	0.17	0.18	0.18	0.18	0.16
659	0.15	0.16	0.16	0.16	0.14
660	0.14	0.15	0.15	0.15	0.14
661	0.13	0.15	0.14	0.14	0.14
662	0.12	0.13	0.12	0.12	0.12
663	0.12	0.13	0.12	0.12	0.12
664	0.10	0.12	0.11	0.12	0.11
665	0.10	0.11	0.10	0.11	0.10
666	0.10	0.11	0.10	0.11	0.10
667	0.10	0.11	0.10	0.10	0.10
668	0.10	0.11	0.10	0.10	0.10
669	0.10	0.11	0.09	0.10	0.10
670	0.09	0.10	0.09	0.10	0.09
671	0.10	0.10	0.09	0.10	0.09
672	0.10	0.11	0.10	0.11	0.10
673	0.11	0.12	0.12	0.13	0.12
674	0.12	0.13	0.13	0.14	0.13
675	0.14	0.16	0.15	0.17	0.16
676	0.18	0.19	0.19	0.20	0.21
677	0.21	0.20	0.22	0.23	0.23
678	0.24	0.22	0.29	0.31	0.30
679	0.28	0.25	0.36	0.40	0.35
680	0.33	0.28	0.43	0.50	0.43
681	0.36	0.31	0.50	0.58	0.46
682	0.35	0.31	0.47	0.56	0.46
683	0.36	0.31	0.46	0.55	0.49
684	0.35	0.30	0.43	0.50	0.47
685	0.31	0.28	0.39	0.42	0.46
686	0.28	0.29	0.40	0.39	0.41
687	0.28	0.28	0.35	0.38	0.38
688	0.27	0.27	0.31	0.35	0.34
689	0.21	0.21	0.24	0.26	0.26
690	0.16	0.18	0.19	0.20	0.19
691	0.14	0.15	0.16	0.17	0.16
692	0.11	0.12	0.12	0.13	0.12
693	0.11	0.12	0.12	0.13	0.12
694	0.10	0.11	0.11	0.12	0.11
695	0.11	0.12	0.10	0.12	0.11
696	0.11	0.12	0.10	0.12	0.11
697	0.12	0.12	0.11	0.12	0.11
698	0.13	0.13	0.11	0.13	0.12

699	0.13	0.13	0.11	0.12	0.12
700	0.15	0.16	0.13	0.15	0.14
701	0.15	0.16	0.14	0.16	0.16
702	0.14	0.14	0.14	0.16	0.15
703	0.14	0.14	0.14	0.14	0.14
704	0.15	0.15	0.15	0.16	0.15
705	0.14	0.13	0.12	0.14	0.14
706	0.14	0.14	0.13	0.15	0.14
707	0.12	0.12	0.11	0.13	0.12
708	0.12	0.12	0.11	0.13	0.12
709	0.13	0.12	0.11	0.13	0.12
710	0.12	0.12	0.12	0.12	0.12
711	0.12	0.12	0.11	0.12	0.12
712	0.11	0.10	0.10	0.10	0.10
713	0.10	0.10	0.10	0.10	0.10
714	0.10	0.10	0.10	0.10	0.10
715	0.10	0.10	0.10	0.10	0.10
716	0.11	0.11	0.11	0.11	0.11
717	0.11	0.11	0.10	0.11	0.11
718	0.11	0.11	0.10	0.10	0.11
719	0.12	0.11	0.10	0.12	0.12
720	0.10	0.10	0.10	0.11	0.10
721	0.11	0.11	0.10	0.11	0.11
722	0.10	0.10	0.09	0.10	0.10
723	0.10	0.12	0.10	0.10	0.10
724	0.09	0.10	0.09	0.10	0.10
725	0.08	0.09	0.08	0.09	0.09
726	0.08	0.08	0.08	0.09	0.09
727	0.08	0.08	0.08	0.08	0.08
728	0.08	0.08	0.08	0.08	0.08
729	0.07	0.08	0.07	0.08	0.08
730	0.07	0.09	0.08	0.08	0.09
731	0.08	0.09	0.09	0.09	0.09
732	0.08	0.09	0.09	0.09	0.09
733	0.08	0.09	0.09	0.08	0.08
734	0.08	0.08	0.08	0.09	0.07
735	0.08	0.08	0.08	0.08	0.07
736	0.08	0.08	0.08	0.08	0.07
737	0.07	0.08	0.07	0.08	0.07
738	0.08	0.08	0.08	0.08	0.08
739	0.08	0.08	0.08	0.09	0.08
740	0.08	0.07	0.08	0.08	0.07
741	0.07	0.07	0.07	0.07	0.07
742	0.08	0.08	0.08	0.08	0.07
743	0.08	0.08	0.08	0.08	0.08
744	0.10	0.10	0.10	0.10	0.10
745	0.11	0.11	0.12	0.12	0.11
746	0.10	0.10	0.10	0.10	0.10
747	0.12	0.11	0.12	0.12	0.13
748	0.10	0.10	0.12	0.11	0.13

749	0.08	0.08	0.09	0.09	0.10
750	0.09	0.09	0.10	0.10	0.11
751	0.10	0.10	0.11	0.11	0.12
752	0.09	0.08	0.10	0.10	0.10
753	0.08	0.08	0.09	0.09	0.09
754	0.09	0.09	0.10	0.10	0.10
755	0.08	0.09	0.10	0.10	0.10
756	0.08	0.08	0.09	0.09	0.09
757	0.09	0.10	0.11	0.12	0.10
758	0.08	0.10	0.10	0.10	0.09
759	0.08	0.08	0.08	0.08	0.08
760	0.07	0.08	0.08	0.08	0.07
761	0.08	0.08	0.08	0.09	0.08
762	0.07	0.08	0.08	0.08	0.08
763	0.07	0.07	0.08	0.08	0.07
764	0.07	0.08	0.08	0.08	0.07
765	0.08	0.08	0.08	0.09	0.08
766	0.08	0.08	0.08	0.08	0.08
767	0.08	0.08	0.08	0.08	0.08
768	0.09	0.10	0.09	0.09	0.08
769	0.09	0.10	0.09	0.10	0.10
770	0.08	0.10	0.08	0.08	0.09
771	0.09	0.10	0.09	0.09	0.09
772	0.10	0.11	0.10	0.10	0.10
773	0.09	0.10	0.09	0.09	0.09
774	0.08	0.09	0.08	0.09	0.08
775	0.09	0.09	0.09	0.10	0.09
776	0.09	0.10	0.09	0.10	0.09
777	0.09	0.09	0.08	0.09	0.08
778	0.09	0.09	0.08	0.09	0.08
779	0.09	0.09	0.08	0.09	0.09
780	0.10	0.09	0.08	0.09	0.09
781	0.09	0.08	0.08	0.08	0.08
782	0.09	0.09	0.08	0.09	0.09
783	0.10	0.10	0.09	0.11	0.10
784	0.11	0.10	0.10	0.11	0.10
785	0.12	0.12	0.10	0.12	0.11
786	0.14	0.15	0.14	0.15	0.14
787	0.12	0.13	0.11	0.13	0.12
788	0.11	0.12	0.11	0.12	0.12
789	0.11	0.12	0.11	0.11	0.12
790	0.14	0.15	0.13	0.14	0.14
791	0.17	0.20	0.15	0.17	0.15
792	0.18	0.20	0.17	0.17	0.16
793	0.18	0.19	0.17	0.17	0.17
794	0.16	0.17	0.17	0.16	0.16
795	0.18	0.19	0.18	0.17	0.17
796	0.16	0.16	0.16	0.15	0.14
797	0.13	0.13	0.12	0.13	0.12
798	0.16	0.16	0.15	0.15	0.14

799	0.15	0.15	0.13	0.14	0.13
800	0.12	0.12	0.12	0.12	0.11
801	0.12	0.13	0.12	0.12	0.12
802	0.11	0.12	0.11	0.12	0.11
803	0.12	0.13	0.13	0.13	0.12
804	0.12	0.12	0.12	0.13	0.12
805	0.11	0.11	0.10	0.11	0.10
806	0.12	0.11	0.11	0.12	0.12
807	0.14	0.14	0.14	0.14	0.15
808	0.22	0.23	0.21	0.21	0.21
809	0.30	0.32	0.29	0.30	0.29
810	0.35	0.36	0.33	0.34	0.33
811	0.30	0.33	0.29	0.31	0.30
812	0.31	0.35	0.32	0.31	0.31
813	0.23	0.26	0.23	0.24	0.24
814	0.17	0.19	0.17	0.17	0.17
815	0.12	0.14	0.13	0.13	0.13
816	0.11	0.12	0.11	0.12	0.12
817	0.12	0.12	0.12	0.13	0.12
818	0.11	0.12	0.11	0.12	0.11
819	0.10	0.11	0.10	0.12	0.11
820	0.11	0.13	0.12	0.13	0.13
821	0.12	0.13	0.12	0.13	0.13
822	0.11	0.12	0.11	0.12	0.12
823	0.12	0.13	0.12	0.12	0.13
824	0.13	0.15	0.14	0.14	0.15
825	0.13	0.15	0.14	0.14	0.15
826	0.13	0.15	0.13	0.13	0.15
827	0.14	0.17	0.15	0.15	0.17
828	0.15	0.18	0.17	0.17	0.18
829	0.15	0.19	0.18	0.17	0.18
830	0.17	0.23	0.19	0.19	0.22
831	0.19	0.28	0.20	0.20	0.23
832	0.21	0.29	0.17	0.18	0.18
833	0.16	0.21	0.13	0.14	0.13
834	0.14	0.17	0.12	0.13	0.12
835	0.15	0.14	0.12	0.12	0.12
836	0.15	0.12	0.12	0.12	0.12
837	0.14	0.11	0.11	0.11	0.11
838	0.16	0.13	0.13	0.13	0.13
839	0.16	0.12	0.12	0.13	0.13
840	0.13	0.10	0.11	0.11	0.11
841	0.15	0.12	0.13	0.13	0.13
842	0.19	0.17	0.16	0.16	0.17
843	0.18	0.17	0.18	0.17	0.17
844	0.18	0.16	0.18	0.16	0.17
845	0.16	0.18	0.20	0.17	0.18
846	0.15	0.17	0.19	0.16	0.18
847	0.14	0.15	0.15	0.13	0.15
848	0.12	0.13	0.14	0.12	0.13

849	0.12	0.12	0.12	0.11	0.13
850	0.11	0.11	0.11	0.11	0.11
851	0.11	0.09	0.10	0.10	0.10
852	0.11	0.10	0.10	0.10	0.10
853	0.09	0.09	0.09	0.09	0.09
854	0.09	0.09	0.09	0.09	0.08
855	0.09	0.09	0.09	0.09	0.08
856	0.09	0.08	0.08	0.09	0.08
857	0.09	0.09	0.09	0.09	0.08
858	0.08	0.08	0.08	0.08	0.07
859	0.08	0.09	0.08	0.09	0.08
860	0.08	0.09	0.08	0.09	0.09
861	0.09	0.09	0.09	0.10	0.09
862	0.09	0.11	0.10	0.10	0.10
863	0.09	0.10	0.09	0.09	0.09
864	0.09	0.09	0.09	0.09	0.09
865	0.10	0.10	0.10	0.10	0.10
866	0.11	0.11	0.11	0.11	0.10
867	0.11	0.11	0.11	0.12	0.11
868	0.12	0.12	0.12	0.11	0.11
869	0.10	0.10	0.10	0.10	0.10
870	0.09	0.09	0.09	0.10	0.10
871	0.10	0.10	0.10	0.11	0.10
872	0.10	0.10	0.10	0.10	0.10
873	0.09	0.09	0.09	0.09	0.09
874	0.09	0.09	0.09	0.09	0.09
875	0.09	0.10	0.10	0.10	0.10
876	0.09	0.09	0.09	0.10	0.10
877	0.09	0.09	0.08	0.09	0.09
878	0.09	0.09	0.09	0.09	0.09
879	0.10	0.10	0.09	0.10	0.10
880	0.10	0.10	0.09	0.10	0.10
881	0.09	0.09	0.09	0.09	0.09
882	0.10	0.09	0.09	0.10	0.10
883	0.10	0.10	0.09	0.10	0.10
884	0.10	0.09	0.09	0.09	0.10
885	0.10	0.10	0.10	0.11	0.11
886	0.10	0.09	0.10	0.10	0.10
887	0.10	0.09	0.09	0.09	0.10
888	0.10	0.10	0.09	0.10	0.10
889	0.13	0.11	0.11	0.12	0.11
890	0.16	0.14	0.13	0.12	0.12
891	0.17	0.15	0.15	0.14	0.15
892	0.14	0.13	0.13	0.13	0.14
893	0.12	0.11	0.11	0.11	0.11
894	0.11	0.10	0.10	0.10	0.10
895	0.10	0.10	0.10	0.10	0.10
896	0.10	0.09	0.09	0.10	0.09
897	0.10	0.10	0.09	0.10	0.09
898	0.10	0.10	0.09	0.10	0.09

899	0.11	0.10	0.10	0.11	0.10
900	0.10	0.09	0.09	0.10	0.10
901	0.09	0.09	0.08	0.09	0.09
902	0.09	0.09	0.08	0.09	0.09
903	0.10	0.09	0.09	0.10	0.09
904	0.09	0.09	0.09	0.09	0.09
905	0.08	0.08	0.08	0.08	0.08
906	0.09	0.08	0.08	0.09	0.09
907	0.09	0.09	0.09	0.09	0.09
908	0.09	0.09	0.09	0.09	0.09
909	0.09	0.09	0.09	0.10	0.09
910	0.10	0.10	0.10	0.11	0.10
911	0.10	0.09	0.10	0.10	0.10
912	0.10	0.09	0.10	0.11	0.10
913	0.11	0.10	0.10	0.11	0.10
914	0.12	0.11	0.11	0.12	0.11
915	0.11	0.11	0.11	0.12	0.11
916	0.11	0.10	0.10	0.11	0.10
917	0.12	0.12	0.11	0.12	0.11
918	0.13	0.12	0.12	0.13	0.12
919	0.13	0.13	0.12	0.12	0.12
920	0.13	0.12	0.12	0.12	0.12
921	0.14	0.14	0.13	0.14	0.13
922	0.14	0.13	0.12	0.13	0.13
923	0.12	0.12	0.11	0.12	0.11
924	0.12	0.12	0.11	0.12	0.12
925	0.13	0.13	0.12	0.13	0.12
926	0.13	0.13	0.12	0.13	0.12
927	0.12	0.12	0.11	0.12	0.11
928	0.13	0.13	0.12	0.13	0.12
929	0.14	0.14	0.12	0.14	0.13
930	0.13	0.14	0.12	0.13	0.12
931	0.12	0.14	0.11	0.13	0.12
932	0.14	0.16	0.13	0.15	0.14
933	0.14	0.16	0.13	0.15	0.14
934	0.12	0.14	0.12	0.13	0.12
935	0.13	0.14	0.13	0.15	0.13
936	0.14	0.17	0.15	0.16	0.15
937	0.14	0.16	0.15	0.16	0.15
938	0.13	0.14	0.14	0.14	0.15
939	0.15	0.17	0.18	0.18	0.17
940	0.17	0.19	0.22	0.20	0.19
941	0.21	0.22	0.26	0.21	0.22
942	0.22	0.23	0.27	0.21	0.23
943	0.20	0.23	0.24	0.20	0.22
944	0.18	0.18	0.21	0.19	0.18
945	0.14	0.14	0.14	0.12	0.13
946	0.16	0.15	0.17	0.14	0.14
947	0.14	0.12	0.15	0.13	0.13
948	0.10	0.10	0.11	0.10	0.11

949	0.11	0.11	0.12	0.12	0.11
950	0.12	0.12	0.13	0.12	0.11
951	0.11	0.10	0.12	0.11	0.10
952	0.10	0.10	0.11	0.11	0.11
953	0.10	0.11	0.11	0.11	0.11
954	0.10	0.10	0.10	0.10	0.10
955	0.10	0.10	0.10	0.10	0.09
956	0.10	0.10	0.10	0.10	0.10
957	0.10	0.10	0.09	0.10	0.10
958	0.09	0.09	0.09	0.09	0.08
959	0.08	0.09	0.08	0.08	0.08
960	0.09	0.09	0.09	0.09	0.09
961	0.09	0.09	0.09	0.09	0.09
962	0.08	0.08	0.08	0.08	0.08
963	0.08	0.08	0.09	0.08	0.08
964	0.09	0.09	0.09	0.09	0.09
965	0.08	0.10	0.08	0.08	0.08
966	0.08	0.09	0.09	0.08	0.08
967	0.09	0.10	0.10	0.09	0.08
968	0.08	0.10	0.09	0.08	0.07
969	0.09	0.09	0.10	0.09	0.08
970	0.08	0.08	0.09	0.09	0.08
971	0.09	0.10	0.11	0.10	0.10
972	0.08	0.09	0.10	0.09	0.08
973	0.09	0.09	0.10	0.10	0.09
974	0.09	0.09	0.10	0.09	0.09
975	0.10	0.09	0.10	0.09	0.09
976	0.09	0.09	0.10	0.10	0.09
977	0.08	0.09	0.09	0.10	0.08
978	0.09	0.09	0.10	0.10	0.09
979	0.08	0.08	0.09	0.09	0.08
980	0.08	0.08	0.08	0.09	0.07
981	0.08	0.08	0.09	0.09	0.08
982	0.09	0.09	0.09	0.10	0.09
983	0.08	0.08	0.09	0.09	0.08
984	0.08	0.08	0.09	0.09	0.08
985	0.09	0.08	0.09	0.10	0.09
986	0.10	0.10	0.11	0.12	0.10
987	0.11	0.11	0.12	0.12	0.11
988	0.09	0.10	0.10	0.11	0.10
989	0.08	0.08	0.09	0.09	0.09
990	0.09	0.09	0.10	0.10	0.11
991	0.10	0.09	0.10	0.11	0.10
992	0.08	0.08	0.09	0.10	0.08
993	0.07	0.07	0.08	0.09	0.08
994	0.07	0.07	0.08	0.08	0.07
995	0.08	0.07	0.08	0.09	0.07
996	0.08	0.07	0.08	0.08	0.07
997	0.07	0.07	0.07	0.07	0.07
998	0.07	0.07	0.08	0.08	0.07

999	0.07	0.07	0.07	0.08	0.06
1000	0.07	0.07	0.07	0.07	0.06
1001	0.06	0.06	0.07	0.07	0.06
1002	0.07	0.07	0.07	0.07	0.06
1003	0.07	0.07	0.07	0.07	0.06
1004	0.06	0.07	0.07	0.07	0.06
1005	0.07	0.07	0.07	0.07	0.06
1006	0.07	0.07	0.07	0.08	0.06
1007	0.07	0.07	0.07	0.07	0.07
1008	0.07	0.07	0.07	0.07	0.07
1009	0.07	0.07	0.08	0.08	0.07
1010	0.07	0.07	0.08	0.08	0.07
1011	0.07	0.07	0.07	0.07	0.07
1012	0.07	0.07	0.07	0.08	0.07
1013	0.07	0.08	0.08	0.08	0.08
1014	0.08	0.08	0.08	0.08	0.08
1015	0.08	0.08	0.08	0.08	0.08
1016	0.08	0.09	0.08	0.09	0.08
1017	0.08	0.09	0.09	0.09	0.09
1018	0.07	0.08	0.08	0.08	0.08
1019	0.08	0.08	0.08	0.08	0.08
1020	0.08	0.08	0.08	0.08	0.09
1021	0.07	0.08	0.08	0.08	0.08
1022	0.07	0.07	0.07	0.08	0.08
1023	0.08	0.08	0.08	0.08	0.08
1024	0.07	0.07	0.07	0.08	0.08
1025	0.07	0.07	0.07	0.07	0.07
1026	0.07	0.07	0.07	0.08	0.07
1027	0.07	0.07	0.07	0.08	0.08
1028	0.07	0.06	0.07	0.08	0.08
1029	0.07	0.06	0.07	0.08	0.07
1030	0.06	0.06	0.07	0.08	0.08
1031	0.06	0.06	0.07	0.08	0.07
1032	0.06	0.07	0.07	0.08	0.07
1033	0.07	0.07	0.07	0.08	0.07
1034	0.07	0.07	0.07	0.08	0.07
1035	0.08	0.08	0.08	0.09	0.08
1036	0.07	0.07	0.07	0.08	0.08
1037	0.07	0.07	0.08	0.09	0.09
1038	0.09	0.08	0.11	0.11	0.11
1039	0.07	0.07	0.08	0.10	0.09
1040	0.08	0.08	0.08	0.10	0.10
1041	0.08	0.09	0.09	0.10	0.10
1042	0.07	0.07	0.08	0.09	0.09
1043	0.07	0.07	0.07	0.08	0.08
1044	0.10	0.09	0.09	0.10	0.09
1045	0.10	0.10	0.09	0.10	0.10
1046	0.11	0.10	0.09	0.10	0.11
1047	0.08	0.08	0.08	0.09	0.09
1048	0.08	0.08	0.07	0.08	0.08

1049	0.08	0.08	0.07	0.08	0.08
1050	0.08	0.08	0.08	0.09	0.08
1051	0.07	0.07	0.07	0.08	0.08
1052	0.08	0.09	0.08	0.09	0.08
1053	0.08	0.08	0.07	0.08	0.08
1054	0.08	0.09	0.08	0.09	0.08
1055	0.09	0.10	0.09	0.10	0.09
1056	0.09	0.10	0.10	0.10	0.10
1057	0.10	0.10	0.10	0.10	0.10
1058	0.09	0.09	0.09	0.09	0.09
1059	0.08	0.09	0.08	0.08	0.09
1060	0.08	0.08	0.08	0.08	0.08
1061	0.08	0.08	0.08	0.09	0.08
1062	0.08	0.08	0.08	0.08	0.08
1063	0.08	0.08	0.08	0.08	0.08
1064	0.08	0.08	0.08	0.09	0.08
1065	0.09	0.08	0.08	0.09	0.09
1066	0.09	0.08	0.08	0.09	0.09
1067	0.10	0.09	0.09	0.10	0.09
1068	0.11	0.10	0.10	0.10	0.10
1069	0.11	0.11	0.10	0.11	0.11
1070	0.12	0.12	0.11	0.12	0.12
1071	0.12	0.12	0.11	0.12	0.12
1072	0.12	0.12	0.11	0.12	0.12
1073	0.12	0.12	0.11	0.12	0.12
1074	0.12	0.12	0.11	0.12	0.11
1075	0.12	0.11	0.11	0.12	0.11
1076	0.12	0.12	0.11	0.12	0.12
1077	0.12	0.11	0.11	0.11	0.11
1078	0.12	0.11	0.11	0.12	0.12
1079	0.12	0.11	0.11	0.12	0.12
1080	0.12	0.12	0.11	0.12	0.12
1081	0.13	0.12	0.12	0.14	0.12
1082	0.14	0.14	0.14	0.15	0.14
1083	0.15	0.16	0.17	0.17	0.16
1084	0.18	0.19	0.19	0.20	0.19
1085	0.18	0.18	0.19	0.20	0.18
1086	0.16	0.15	0.16	0.17	0.16
1087	0.13	0.13	0.13	0.14	0.14
1088	0.12	0.12	0.12	0.13	0.12
1089	0.11	0.10	0.11	0.12	0.11
1090	0.11	0.10	0.11	0.12	0.11
1091	0.12	0.11	0.12	0.12	0.12
1092	0.12	0.10	0.12	0.12	0.12
1093	0.11	0.10	0.10	0.11	0.11
1094	0.10	0.09	0.09	0.10	0.10
1095	0.11	0.10	0.10	0.11	0.11
1096	0.12	0.11	0.11	0.12	0.12
1097	0.13	0.13	0.12	0.13	0.13
1098	0.22	0.19	0.20	0.21	0.20

1099	0.25	0.22	0.22	0.25	0.25
1100	0.27	0.23	0.23	0.25	0.24
1101	0.19	0.16	0.17	0.18	0.18
1102	0.14	0.12	0.12	0.14	0.14
1103	0.13	0.11	0.11	0.13	0.13
1104	0.11	0.10	0.10	0.11	0.11
1105	0.11	0.10	0.10	0.11	0.11
1106	0.11	0.11	0.10	0.11	0.10
1107	0.11	0.11	0.10	0.11	0.10
1108	0.11	0.10	0.10	0.11	0.10
1109	0.11	0.11	0.10	0.11	0.10
1110	0.11	0.10	0.11	0.11	0.10
1111	0.12	0.11	0.12	0.13	0.12
1112	0.13	0.11	0.12	0.13	0.13
1113	0.13	0.12	0.12	0.13	0.13
1114	0.13	0.12	0.13	0.14	0.13
1115	0.13	0.12	0.12	0.13	0.12
1116	0.14	0.13	0.13	0.14	0.14
1117	0.14	0.14	0.14	0.15	0.15
1118	0.15	0.14	0.15	0.15	0.15
1119	0.14	0.12	0.12	0.13	0.13
1120	0.13	0.11	0.12	0.13	0.12
1121	0.13	0.12	0.13	0.14	0.13
1122	0.14	0.13	0.13	0.14	0.14
1123	0.15	0.14	0.15	0.16	0.15
1124	0.18	0.18	0.18	0.20	0.18
1125	0.17	0.17	0.17	0.18	0.17
1126	0.16	0.16	0.16	0.17	0.16
1127	0.20	0.20	0.19	0.20	0.19
1128	0.20	0.21	0.20	0.20	0.19
1129	0.17	0.18	0.16	0.17	0.17
1130	0.19	0.18	0.17	0.19	0.18
1131	0.18	0.17	0.16	0.18	0.17
1132	0.15	0.14	0.14	0.16	0.14
1133	0.15	0.14	0.14	0.16	0.15
1134	0.16	0.16	0.16	0.17	0.16
1135	0.16	0.16	0.16	0.16	0.17
1136	0.17	0.17	0.17	0.17	0.18
1137	0.15	0.16	0.16	0.16	0.18
1138	0.16	0.18	0.19	0.17	0.21
1139	0.20	0.24	0.26	0.21	0.28

Supplementary Table S4. Average Cα-positional deviations in the 10 ns after LA removal

	**Deviation after 10 ns (nm)**				
**Cα atom**	**Wild type**	**Alpha**	**Delta**	**Delta plus**	**Omicron**
1	0.42	0.49	0.69	0.46	0.61
2	0.34	0.38	0.53	0.38	0.50
3	0.33	0.33	0.43	0.35	0.45
4	0.34	0.29	0.38	0.33	0.40

5	0.35	0.28	0.37	0.33	0.39
6	0.33	0.29	0.37	0.33	0.36
7	0.36	0.32	0.39	0.32	0.37
8	0.34	0.33	0.37	0.33	0.34
9	0.32	0.33	0.39	0.33	0.32
10	0.29	0.34	0.37	0.34	0.30
11	0.26	0.31	0.36	0.32	0.28
12	0.25	0.26	0.34	0.31	0.24
13	0.22	0.20	0.30	0.27	0.21
14	0.20	0.20	0.26	0.24	0.20
15	0.18	0.18	0.23	0.21	0.18
16	0.20	0.20	0.24	0.23	0.20
17	0.22	0.22	0.23	0.24	0.21
18	0.21	0.21	0.22	0.22	0.20
19	0.26	0.25	0.25	0.24	0.24
20	0.25	0.26	0.24	0.24	0.23
21	0.23	0.22	0.21	0.21	0.20
22	0.25	0.24	0.22	0.23	0.22
23	0.25	0.24	0.23	0.23	0.23
24	0.23	0.23	0.23	0.23	0.22
25	0.23	0.24	0.23	0.24	0.22
26	0.22	0.21	0.21	0.21	0.21
27	0.17	0.18	0.17	0.19	0.17
28	0.16	0.16	0.16	0.18	0.15
29	0.14	0.15	0.15	0.17	0.14
30	0.14	0.14	0.14	0.15	0.14
31	0.12	0.13	0.12	0.14	0.13
32	0.12	0.13	0.12	0.14	0.12
33	0.12	0.12	0.12	0.12	0.10
34	0.11	0.11	0.11	0.12	0.10
35	0.11	0.11	0.11	0.11	0.10
36	0.10	0.10	0.10	0.10	0.09
37	0.10	0.09	0.09	0.10	0.09
38	0.10	0.09	0.09	0.10	0.09
39	0.11	0.10	0.10	0.10	0.10
40	0.09	0.09	0.09	0.09	0.09
41	0.09	0.09	0.09	0.09	0.09
42	0.09	0.09	0.09	0.09	0.09
43	0.09	0.09	0.09	0.09	0.08
44	0.10	0.09	0.10	0.09	0.09
45	0.11	0.10	0.10	0.10	0.10
46	0.12	0.11	0.11	0.11	0.10
47	0.12	0.11	0.11	0.11	0.10
48	0.12	0.11	0.12	0.11	0.10
49	0.11	0.10	0.11	0.10	0.10
50	0.11	0.11	0.11	0.11	0.10
51	0.11	0.11	0.11	0.11	0.10
52	0.11	0.11	0.10	0.11	0.10
53	0.10	0.10	0.10	0.11	0.09
54	0.10	0.10	0.09	0.10	0.09

55	0.10	0.10	0.09	0.10	0.09
56	0.10	0.10	0.10	0.11	0.10
57	0.11	0.11	0.10	0.12	0.10
58	0.11	0.11	0.11	0.12	0.10
59	0.13	0.13	0.13	0.14	0.12
60	0.14	0.14	0.13	0.15	0.13
61	0.14	0.15	0.14	0.16	0.14
62	0.13	0.13	0.13	0.14	0.12
63	0.13	0.14	0.13	0.14	0.12
64	0.14	0.14	0.13	0.15	0.13
65	0.13	0.14	0.14	0.15	0.14
66	0.15	0.15	0.15	0.16	0.15
67	0.16	0.17	0.16	0.18	0.16
68	0.18	0.19	0.19	0.20	0.18
69	0.20	n.a.	0.21	0.22	n.a.
70	0.26	n.a.	0.25	0.26	n.a.
71	0.36	0.22	0.36	0.34	0.21
72	0.46	0.30	0.44	0.42	0.27
73	0.53	0.38	0.48	0.47	0.37
74	0.50	0.41	0.43	0.43	0.41
75	0.38	0.37	0.33	0.34	0.37
76	0.28	0.26	0.26	0.28	0.27
77	0.22	0.21	0.21	0.22	0.20
78	0.19	0.18	0.18	0.19	0.17
79	0.17	0.16	0.17	0.17	0.16
80	0.15	0.15	0.16	0.16	0.15
81	0.14	0.14	0.15	0.15	0.14
82	0.14	0.14	0.15	0.15	0.14
83	0.13	0.13	0.14	0.14	0.13
84	0.12	0.13	0.13	0.13	0.12
85	0.12	0.13	0.13	0.14	0.12
86	0.11	0.11	0.11	0.12	0.11
87	0.14	0.16	0.15	0.16	0.14
88	0.14	0.17	0.15	0.17	0.14
89	0.11	0.11	0.10	0.12	0.10
90	0.10	0.10	0.10	0.11	0.09
91	0.09	0.09	0.10	0.10	0.09
92	0.10	0.10	0.10	0.11	0.09
93	0.11	0.11	0.11	0.12	0.10
94	0.12	0.12	0.13	0.13	0.12
95	0.13	0.14	0.14	0.15	0.13
96	0.15	0.15	0.15	0.16	0.14
97	0.17	0.17	0.17	0.19	0.16
98	0.17	0.18	0.18	0.20	0.17
99	0.15	0.17	0.17	0.17	0.15
100	0.14	0.15	0.15	0.16	0.14
101	0.12	0.13	0.14	0.14	0.13
102	0.12	0.13	0.14	0.14	0.12
103	0.12	0.12	0.13	0.13	0.11
104	0.10	0.11	0.12	0.12	0.10

105	0.11	0.11	0.12	0.12	0.10
106	0.10	0.11	0.11	0.11	0.10
107	0.11	0.12	0.12	0.12	0.11
108	0.13	0.14	0.15	0.14	0.13
109	0.15	0.15	0.17	0.16	0.14
110	0.14	0.14	0.16	0.15	0.13
111	0.16	0.15	0.19	0.18	0.15
112	0.17	0.17	0.20	0.19	0.17
113	0.18	0.17	0.21	0.20	0.18
114	0.15	0.14	0.17	0.17	0.15
115	0.13	0.13	0.14	0.15	0.13
116	0.12	0.11	0.13	0.13	0.11
117	0.11	0.11	0.12	0.12	0.10
118	0.11	0.11	0.12	0.12	0.11
119	0.11	0.11	0.12	0.12	0.11
120	0.12	0.12	0.13	0.14	0.12
121	0.14	0.15	0.15	0.15	0.14
122	0.17	0.17	0.18	0.18	0.17
123	0.21	0.21	0.23	0.22	0.21
124	0.21	0.21	0.22	0.21	0.21
125	0.18	0.18	0.18	0.19	0.17
126	0.15	0.15	0.15	0.17	0.14
127	0.14	0.14	0.14	0.15	0.14
128	0.12	0.13	0.13	0.14	0.12
129	0.12	0.12	0.13	0.14	0.12
130	0.12	0.12	0.13	0.14	0.12
131	0.13	0.13	0.14	0.15	0.13
132	0.14	0.14	0.16	0.16	0.14
133	0.14	0.13	0.16	0.16	0.14
134	0.16	0.15	0.19	0.17	0.15
135	0.14	0.14	0.19	0.17	0.14
136	0.16	0.16	0.20	0.18	0.15
137	0.17	0.17	0.20	0.18	0.16
138	0.16	0.15	0.18	0.17	0.16
139	0.15	0.14	0.17	0.16	0.14
140	0.15	0.15	0.17	0.16	0.15
141	0.16	0.15	0.17	0.17	0.15
142	0.18	0.18	0.21	0.19	0.17
143	0.18	0.19	0.23	0.20	n.a.
144	0.21	n.a.	0.27	0.23	n.a.
145	0.24	0.23	0.32	0.26	n.a.
146	0.31	0.27	0.37	0.31	0.20
147	0.38	0.32	0.47	0.36	0.23
148	0.38	0.36	0.50	0.40	0.28
149	0.34	0.34	0.46	0.37	0.30
150	0.34	0.33	0.46	0.35	0.33
151	0.27	0.31	0.38	0.30	0.30
152	0.23	0.27	0.30	0.26	0.27
153	0.22	0.24	0.25	0.24	0.23
154	0.20	0.20	0.22	0.21	0.20

155	0.21	0.21	0.21	0.22	0.21
156	0.19	0.19	n.a.	n.a.	0.19
157	0.17	0.17	n.a.	n.a.	0.17
158	0.16	0.16	n.a.	0.21	0.16
159	0.14	0.14	0.20	0.19	0.14
160	0.15	0.14	0.20	0.19	0.15
161	0.18	0.17	0.23	0.18	0.17
162	0.18	0.17	0.23	0.20	0.17
163	0.16	0.16	0.21	0.19	0.16
164	0.17	0.16	0.22	0.22	0.17
165	0.16	0.17	0.19	0.20	0.17
166	0.15	0.15	0.17	0.18	0.16
167	0.15	0.15	0.17	0.18	0.16
168	0.14	0.14	0.15	0.17	0.14
169	0.15	0.15	0.15	0.17	0.15
170	0.15	0.15	0.16	0.17	0.15
171	0.16	0.16	0.17	0.19	0.16
172	0.18	0.18	0.18	0.20	0.18
173	0.22	0.22	0.21	0.25	0.21
174	0.22	0.22	0.21	0.23	0.20
175	0.23	0.23	0.23	0.23	0.20
176	0.24	0.26	0.24	0.24	0.22
177	0.21	0.24	0.22	0.22	0.20
178	0.22	0.24	0.24	0.23	0.22
179	0.21	0.26	0.28	0.24	0.25
180	0.24	0.28	0.28	0.27	0.26
181	0.27	0.28	0.29	0.29	0.29
182	0.28	0.28	0.29	0.29	0.27
183	0.28	0.28	0.29	0.29	0.27
184	0.26	0.24	0.25	0.26	0.25
185	0.20	0.21	0.20	0.23	0.21
186	0.17	0.17	0.17	0.19	0.17
187	0.17	0.17	0.17	0.19	0.16
188	0.15	0.15	0.15	0.17	0.14
189	0.13	0.13	0.13	0.14	0.12
190	0.12	0.11	0.12	0.12	0.11
191	0.10	0.10	0.10	0.11	0.10
192	0.10	0.09	0.09	0.10	0.09
193	0.09	0.08	0.09	0.10	0.08
194	0.09	0.09	0.09	0.10	0.08
195	0.10	0.10	0.09	0.10	0.09
196	0.10	0.10	0.10	0.11	0.10
197	0.12	0.13	0.11	0.13	0.12
198	0.14	0.16	0.13	0.16	0.14
199	0.13	0.14	0.12	0.15	0.13
200	0.11	0.11	0.10	0.12	0.11
201	0.10	0.10	0.10	0.11	0.09
202	0.09	0.09	0.09	0.10	0.09
203	0.09	0.08	0.09	0.10	0.09
204	0.09	0.08	0.09	0.10	0.08

205	0.10	0.09	0.10	0.11	0.09
206	0.11	0.10	0.11	0.12	0.10
207	0.12	0.12	0.13	0.13	0.12
208	0.14	0.14	0.14	0.15	0.13
209	0.16	0.17	0.17	0.18	0.15
210	0.17	0.18	0.17	0.19	0.16
211	0.21	0.21	0.20	0.23	n.a.
212	0.21	0.21	0.21	0.23	0.21
213	0.24	0.24	0.25	0.26	0.20
214	0.24	0.24	0.25	0.27	0.22
215	0.23	0.24	0.23	0.26	0.22
P insertion	n.a.	n.a.	n.a.	n.a.	0.21
E insertion	n.a.	n.a.	n.a.	n.a.	0.21
D insertion	n.a.	n.a.	n.a.	n.a.	0.19
216	0.21	0.20	0.20	0.22	0.17
217	0.22	0.22	0.20	0.24	0.18
218	0.22	0.21	0.21	0.25	0.18
219	0.20	0.20	0.19	0.20	0.16
220	0.15	0.15	0.15	0.16	0.14
221	0.13	0.14	0.15	0.14	0.12
222	0.11	0.11	0.11	0.11	0.10
223	0.10	0.09	0.09	0.10	0.09
224	0.10	0.09	0.10	0.10	0.09
225	0.10	0.09	0.10	0.10	0.09
226	0.11	0.10	0.10	0.11	0.10
227	0.11	0.11	0.11	0.12	0.10
228	0.10	0.10	0.10	0.12	0.10
229	0.11	0.12	0.11	0.12	0.10
230	0.12	0.12	0.11	0.13	0.11
231	0.11	0.11	0.11	0.13	0.11
232	0.12	0.12	0.11	0.15	0.12
233	0.12	0.12	0.12	0.14	0.11
234	0.12	0.14	0.14	0.14	0.13
235	0.11	0.12	0.12	0.12	0.12
236	0.12	0.12	0.13	0.13	0.12
237	0.12	0.12	0.12	0.13	0.11
238	0.11	0.11	0.12	0.12	0.11
239	0.12	0.12	0.12	0.12	0.11
240	0.11	0.12	0.13	0.12	0.11
241	0.12	0.13	0.14	0.14	0.12
242	0.14	0.14	0.15	0.15	0.14
243	0.15	0.15	0.16	0.16	0.15
244	0.17	0.17	0.18	0.19	0.17
245	0.19	0.20	0.22	0.20	0.19
246	0.22	0.23	0.25	0.23	0.23
247	0.26	0.28	0.32	0.26	0.28
248	0.30	0.32	0.41	0.32	0.35
249	0.33	0.39	0.47	0.35	0.42
250	0.32	0.39	0.47	0.33	0.45
251	0.36	0.44	0.48	0.36	0.48

252	0.38	0.44	0.47	0.35	0.51
253	0.35	0.40	0.39	0.31	0.43
254	0.33	0.36	0.33	0.30	0.41
255	0.33	0.35	0.31	0.27	0.38
256	0.31	0.32	0.28	0.26	0.32
257	0.25	0.27	0.26	0.26	0.24
258	0.21	0.22	0.22	0.23	0.21
259	0.20	0.21	0.21	0.22	0.20
260	0.19	0.19	0.20	0.21	0.19
261	0.20	0.19	0.20	0.20	0.19
262	0.18	0.17	0.18	0.19	0.17
263	0.15	0.15	0.16	0.17	0.15
264	0.14	0.14	0.14	0.16	0.14
265	0.12	0.12	0.13	0.14	0.12
266	0.12	0.12	0.12	0.13	0.11
267	0.11	0.11	0.11	0.12	0.11
268	0.11	0.12	0.11	0.13	0.11
269	0.11	0.11	0.10	0.12	0.10
270	0.10	0.10	0.10	0.11	0.10
271	0.11	0.11	0.11	0.12	0.10
272	0.11	0.11	0.11	0.12	0.10
273	0.10	0.11	0.10	0.11	0.09
274	0.10	0.11	0.10	0.10	0.09
275	0.10	0.10	0.10	0.10	0.09
276	0.10	0.10	0.10	0.10	0.09
277	0.10	0.10	0.09	0.09	0.09
278	0.10	0.10	0.10	0.10	0.09
279	0.10	0.10	0.10	0.10	0.09
280	0.12	0.11	0.11	0.11	0.10
281	0.13	0.13	0.12	0.12	0.12
282	0.13	0.13	0.12	0.12	0.12
283	0.12	0.11	0.11	0.11	0.10
284	0.11	0.10	0.10	0.11	0.10
285	0.10	0.10	0.10	0.10	0.10
286	0.11	0.11	0.11	0.11	0.10
287	0.11	0.11	0.10	0.10	0.10
288	0.10	0.10	0.10	0.10	0.09
289	0.11	0.10	0.10	0.10	0.09
290	0.10	0.10	0.10	0.10	0.09
291	0.10	0.10	0.10	0.10	0.09
292	0.11	0.12	0.11	0.12	0.11
293	0.11	0.12	0.11	0.12	0.10
294	0.11	0.12	0.11	0.12	0.10
295	0.11	0.11	0.10	0.11	0.10
296	0.11	0.11	0.11	0.11	0.10
297	0.10	0.11	0.10	0.10	0.09
298	0.10	0.11	0.10	0.10	0.10
299	0.11	0.11	0.11	0.11	0.10
300	0.11	0.10	0.10	0.11	0.10
301	0.11	0.11	0.10	0.11	0.10

302	0.12	0.12	0.12	0.12	0.11
303	0.13	0.13	0.13	0.13	0.12
304	0.12	0.12	0.12	0.13	0.12
305	0.12	0.12	0.12	0.13	0.11
306	0.12	0.12	0.12	0.13	0.12
307	0.14	0.13	0.15	0.14	0.14
308	0.13	0.13	0.13	0.14	0.13
309	0.14	0.14	0.14	0.14	0.13
310	0.13	0.13	0.13	0.13	0.13
311	0.12	0.12	0.12	0.12	0.12
312	0.10	0.11	0.10	0.10	0.11
313	0.09	0.10	0.10	0.10	0.10
314	0.09	0.10	0.10	0.10	0.10
315	0.10	0.10	0.10	0.10	0.10
316	0.09	0.10	0.09	0.09	0.10
317	0.09	0.09	0.09	0.09	0.09
318	0.09	0.09	0.09	0.10	0.10
319	0.10	0.11	0.10	0.10	0.12
320	0.11	0.12	0.11	0.12	0.12
321	0.12	0.13	0.12	0.13	0.13
322	0.11	0.12	0.12	0.12	0.12
323	0.14	0.15	0.14	0.15	0.14
324	0.13	0.13	0.13	0.14	0.13
325	0.12	0.13	0.13	0.12	0.12
326	0.11	0.11	0.12	0.12	0.10
327	0.10	0.10	0.11	0.11	0.10
328	0.10	0.10	0.10	0.10	0.10
329	0.10	0.10	0.10	0.10	0.10
330	0.11	0.11	0.12	0.11	0.11
331	0.14	0.14	0.15	0.14	0.13
332	0.14	0.14	0.14	0.13	0.13
333	0.16	0.17	0.18	0.17	0.15
334	0.15	0.15	0.17	0.15	0.14
335	0.14	0.14	0.15	0.14	0.13
336	0.13	0.12	0.14	0.13	0.12
337	0.14	0.14	0.15	0.15	0.14
338	0.19	0.18	0.18	0.18	0.16
339	0.22	0.20	0.20	0.20	0.17
340	0.20	0.19	0.19	0.19	0.17
341	0.15	0.16	0.15	0.15	0.17
342	0.13	0.14	0.13	0.13	0.15
343	0.14	0.15	0.15	0.15	0.16
344	0.15	0.16	0.15	0.16	0.16
345	0.19	0.20	0.19	0.21	0.20
346	0.20	0.18	0.17	0.19	0.18
347	0.12	0.11	0.12	0.12	0.12
348	0.10	0.10	0.11	0.10	0.10
349	0.10	0.10	0.10	0.10	0.10
350	0.09	0.09	0.09	0.10	0.09
351	0.10	0.10	0.10	0.11	0.10

352	0.10	0.10	0.11	0.11	0.10
353	0.10	0.10	0.10	0.10	0.10
354	0.12	0.11	0.10	0.11	0.11
355	0.11	0.10	0.10	0.10	0.10
356	0.11	0.11	0.10	0.11	0.11
357	0.10	0.10	0.10	0.11	0.11
358	0.10	0.10	0.11	0.11	0.10
359	0.11	0.10	0.11	0.11	0.10
360	0.11	0.11	0.12	0.12	0.11
361	0.12	0.11	0.13	0.12	0.10
362	0.12	0.12	0.13	0.12	0.11
363	0.14	0.13	0.14	0.13	0.12
364	0.16	0.15	0.16	0.17	0.14
365	0.17	0.16	0.19	0.20	0.16
366	0.18	0.16	0.20	0.21	0.17
367	0.19	0.18	0.20	0.23	0.18
368	0.18	0.17	0.19	0.21	0.17
369	0.17	0.15	0.19	0.20	0.17
370	0.18	0.18	0.21	0.21	0.19
371	0.21	0.18	0.24	0.23	0.18
372	0.16	0.17	0.18	0.20	0.17
373	0.13	0.15	0.15	0.17	0.13
374	0.11	0.11	0.11	0.12	0.11
375	0.11	0.11	0.12	0.12	0.10
376	0.11	0.11	0.12	0.12	0.11
377	0.11	0.11	0.12	0.11	0.11
378	0.10	0.10	0.10	0.10	0.10
379	0.09	0.09	0.09	0.10	0.09
380	0.09	0.09	0.09	0.10	0.10
381	0.09	0.10	0.10	0.10	0.10
382	0.08	0.09	0.09	0.09	0.09
383	0.09	0.09	0.09	0.10	0.10
384	0.10	0.10	0.10	0.11	0.11
385	0.10	0.11	0.11	0.13	0.11
386	0.10	0.10	0.11	0.12	0.11
387	0.11	0.10	0.12	0.12	0.12
388	0.14	0.13	0.14	0.14	0.12
389	0.13	0.11	0.12	0.13	0.12
390	0.10	0.10	0.10	0.10	0.10
391	0.09	0.09	0.09	0.10	0.09
392	0.09	0.09	0.09	0.09	0.09
393	0.10	0.10	0.10	0.10	0.10
394	0.10	0.11	0.12	0.10	0.11
395	0.09	0.09	0.09	0.09	0.09
396	0.09	0.09	0.09	0.09	0.09
397	0.09	0.09	0.09	0.10	0.09
398	0.09	0.09	0.09	0.09	0.09
399	0.09	0.09	0.09	0.10	0.09
400	0.09	0.08	0.09	0.09	0.08
401	0.09	0.08	0.09	0.09	0.09

402	0.09	0.08	0.09	0.09	0.09
403	0.09	0.09	0.10	0.10	0.09
404	0.10	0.10	0.10	0.11	0.10
405	0.10	0.11	0.10	0.11	0.10
406	0.09	0.10	0.10	0.10	0.09
407	0.09	0.09	0.10	0.10	0.09
408	0.09	0.09	0.10	0.10	0.09
409	0.09	0.09	0.10	0.10	0.09
410	0.09	0.08	0.09	0.09	0.09
411	0.09	0.09	0.10	0.10	0.10
412	0.10	0.10	0.11	0.11	0.10
413	0.12	0.11	0.13	0.13	0.12
414	0.11	0.10	0.12	0.12	0.12
415	0.11	0.10	0.13	0.12	0.12
416	0.10	0.10	0.12	0.12	0.11
417	0.10	0.09	0.11	0.12	0.10
418	0.09	0.08	0.10	0.10	0.09
419	0.09	0.09	0.11	0.11	0.09
420	0.10	0.10	0.12	0.12	0.10
421	0.10	0.10	0.11	0.11	0.10
422	0.09	0.09	0.10	0.10	0.09
423	0.09	0.08	0.10	0.10	0.09
424	0.09	0.09	0.10	0.10	0.09
425	0.09	0.09	0.10	0.10	0.09
426	0.10	0.09	0.10	0.11	0.10
427	0.11	0.10	0.11	0.12	0.11
428	0.12	0.10	0.11	0.13	0.11
429	0.10	0.09	0.10	0.11	0.10
430	0.09	0.09	0.10	0.10	0.09
431	0.08	0.08	0.09	0.09	0.09
432	0.08	0.08	0.08	0.09	0.09
433	0.08	0.08	0.08	0.09	0.09
434	0.09	0.09	0.09	0.09	0.09
435	0.09	0.08	0.09	0.09	0.09
436	0.10	0.09	0.10	0.10	0.10
437	0.10	0.10	0.11	0.11	0.10
438	0.10	0.10	0.11	0.11	0.10
439	0.11	0.12	0.12	0.12	0.11
440	0.13	0.14	0.15	0.15	0.14
441	0.13	0.13	0.14	0.14	0.12
442	0.11	0.12	0.12	0.12	0.11
443	0.12	0.13	0.14	0.14	0.12
444	0.15	0.16	0.19	0.17	0.15
445	0.17	0.19	0.24	0.20	0.17
446	0.19	0.20	0.26	0.22	0.19
447	0.15	0.18	0.23	0.18	0.17
448	0.14	0.15	0.18	0.16	0.14
449	0.15	0.16	0.16	0.17	0.14
450	0.14	0.14	0.15	0.15	0.14
451	0.11	0.11	0.11	0.12	0.11

452	0.10	0.10	0.11	0.12	0.11
453	0.09	0.10	0.10	0.10	0.10
454	0.10	0.10	0.11	0.11	0.10
455	0.12	0.11	0.13	0.13	0.12
456	0.14	0.14	0.16	0.15	0.14
457	0.14	0.14	0.15	0.14	0.14
458	0.17	0.16	0.18	0.17	0.16
459	0.16	0.15	0.17	0.16	0.15
460	0.14	0.13	0.16	0.14	0.13
461	0.12	0.11	0.13	0.12	0.11
462	0.11	0.11	0.12	0.12	0.10
463	0.10	0.10	0.11	0.11	0.10
464	0.10	0.10	0.10	0.11	0.10
465	0.10	0.10	0.11	0.11	0.10
466	0.11	0.10	0.11	0.11	0.11
467	0.12	0.11	0.12	0.12	0.11
468	0.12	0.13	0.14	0.14	0.13
469	0.15	0.14	0.16	0.15	0.14
470	0.16	0.15	0.17	0.18	0.17
471	0.19	0.17	0.21	0.19	0.19
472	0.18	0.17	0.20	0.20	0.18
473	0.20	0.18	0.22	0.20	0.21
474	0.25	0.22	0.27	0.25	0.25
475	0.31	0.26	0.33	0.31	0.31
476	0.41	0.34	0.46	0.42	0.40
477	0.45	0.40	0.52	0.48	0.46
478	0.44	0.38	0.48	0.45	0.45
479	0.42	0.36	0.44	0.42	0.41
480	0.35	0.31	0.37	0.36	0.35
481	0.44	0.40	0.46	0.46	0.47
482	0.45	0.39	0.43	0.47	0.53
483	0.40	0.34	0.37	0.42	0.50
484	0.36	0.29	0.32	0.34	0.42
485	0.37	0.30	0.32	0.35	0.39
486	0.34	0.31	0.34	0.34	0.36
487	0.29	0.27	0.32	0.29	0.31
488	0.23	0.21	0.25	0.24	0.23
489	0.18	0.17	0.20	0.20	0.19
490	0.15	0.15	0.16	0.17	0.16
491	0.13	0.13	0.14	0.14	0.14
492	0.12	0.12	0.12	0.13	0.12
493	0.12	0.12	0.12	0.13	0.12
494	0.12	0.12	0.13	0.13	0.12
495	0.12	0.12	0.12	0.12	0.11
496	0.13	0.14	0.14	0.13	0.13
497	0.11	0.13	0.12	0.12	0.12
498	0.13	0.15	0.14	0.14	0.13
499	0.14	0.18	0.17	0.16	0.16
500	0.16	0.21	0.18	0.17	0.17
501	0.13	0.18	0.15	0.14	0.15

502	0.13	0.16	0.14	0.15	0.15
503	0.12	0.13	0.13	0.13	0.13
504	0.11	0.12	0.13	0.13	0.11
505	0.11	0.11	0.11	0.12	0.11
506	0.10	0.11	0.10	0.11	0.10
507	0.09	0.09	0.10	0.10	0.09
508	0.09	0.09	0.09	0.09	0.09
509	0.09	0.08	0.09	0.09	0.09
510	0.08	0.08	0.08	0.09	0.08
511	0.08	0.08	0.09	0.09	0.09
512	0.08	0.08	0.09	0.09	0.08
513	0.09	0.08	0.09	0.09	0.09
514	0.09	0.09	0.09	0.09	0.09
515	0.10	0.10	0.10	0.10	0.10
516	0.10	0.10	0.10	0.10	0.09
517	0.09	0.09	0.09	0.09	0.09
518	0.09	0.10	0.10	0.10	0.09
519	0.10	0.10	0.10	0.10	0.10
520	0.09	0.10	0.10	0.10	0.09
521	0.09	0.10	0.10	0.10	0.10
522	0.10	0.10	0.10	0.10	0.10
523	0.11	0.11	0.12	0.12	0.11
524	0.10	0.10	0.10	0.10	0.10
525	0.10	0.10	0.11	0.10	0.10
526	0.13	0.13	0.14	0.13	0.11
527	0.14	0.15	0.16	0.15	0.13
528	0.14	0.15	0.16	0.15	0.14
529	0.15	0.15	0.16	0.16	0.14
530	0.12	0.12	0.13	0.13	0.12
531	0.12	0.12	0.12	0.12	0.12
532	0.13	0.14	0.14	0.14	0.12
533	0.12	0.13	0.13	0.13	0.12
534	0.13	0.13	0.13	0.13	0.12
535	0.12	0.12	0.12	0.12	0.11
536	0.12	0.13	0.12	0.13	0.12
537	0.12	0.12	0.12	0.12	0.12
538	0.10	0.11	0.11	0.11	0.10
539	0.10	0.10	0.10	0.10	0.10
540	0.10	0.10	0.10	0.10	0.10
541	0.09	0.10	0.10	0.10	0.10
542	0.09	0.10	0.10	0.09	0.09
543	0.10	0.09	0.09	0.09	0.09
544	0.10	0.10	0.10	0.09	0.09
545	0.11	0.11	0.10	0.10	0.09
546	0.09	0.10	0.09	0.09	0.09
547	0.10	0.11	0.10	0.10	0.09
548	0.11	0.11	0.10	0.10	0.09
549	0.10	0.10	0.10	0.10	0.09
550	0.10	0.10	0.10	0.10	0.09
551	0.10	0.10	0.10	0.10	0.09

552	0.10	0.10	0.10	0.10	0.10
553	0.12	0.11	0.10	0.11	0.10
554	0.13	0.12	0.11	0.12	0.11
555	0.12	0.12	0.12	0.12	0.11
556	0.16	0.15	0.14	0.14	0.15
557	0.13	0.12	0.12	0.12	0.12
558	0.14	0.13	0.14	0.14	0.14
559	0.11	0.11	0.12	0.11	0.10
560	0.12	0.11	0.13	0.12	0.12
561	0.13	0.12	0.14	0.13	0.13
562	0.11	0.11	0.12	0.11	0.11
563	0.10	0.09	0.10	0.09	0.09
564	0.09	0.09	0.10	0.09	0.09
565	0.09	0.08	0.09	0.09	0.08
566	0.09	0.09	0.09	0.09	0.08
567	0.09	0.09	0.09	0.10	0.08
568	0.10	0.10	0.10	0.10	0.09
569	0.12	0.12	0.12	0.12	0.10
570	0.11	0.11	0.12	0.11	0.10
571	0.10	0.10	0.11	0.10	0.10
572	0.11	0.10	0.11	0.11	0.09
573	0.10	0.10	0.10	0.10	0.09
574	0.10	0.09	0.10	0.10	0.09
575	0.10	0.09	0.09	0.09	0.08
576	0.09	0.09	0.09	0.09	0.09
577	0.10	0.10	0.10	0.10	0.09
578	0.10	0.10	0.11	0.11	0.10
579	0.11	0.11	0.11	0.12	0.11
580	0.13	0.14	0.14	0.14	0.12
581	0.13	0.13	0.14	0.13	0.13
582	0.12	0.12	0.13	0.12	0.12
583	0.12	0.12	0.12	0.12	0.12
584	0.11	0.10	0.11	0.10	0.10
585	0.10	0.10	0.10	0.10	0.09
586	0.10	0.10	0.10	0.10	0.09
587	0.10	0.10	0.10	0.10	0.09
588	0.11	0.10	0.10	0.10	0.09
589	0.10	0.10	0.09	0.10	0.09
590	0.10	0.10	0.10	0.10	0.10
591	0.10	0.10	0.10	0.10	0.10
592	0.10	0.09	0.09	0.10	0.10
593	0.10	0.10	0.10	0.11	0.11
594	0.09	0.10	0.09	0.10	0.10
595	0.09	0.09	0.09	0.10	0.09
596	0.09	0.09	0.09	0.09	0.10
597	0.09	0.10	0.09	0.10	0.10
598	0.10	0.11	0.10	0.11	0.11
599	0.12	0.12	0.12	0.12	0.12
600	0.14	0.14	0.14	0.14	0.13
601	0.14	0.14	0.14	0.15	0.14

602	0.16	0.15	0.16	0.17	0.15
603	0.20	0.18	0.20	0.21	0.20
604	0.18	0.17	0.18	0.19	0.18
605	0.17	0.16	0.17	0.18	0.16
606	0.18	0.17	0.19	0.20	0.19
607	0.15	0.15	0.16	0.16	0.15
608	0.12	0.12	0.12	0.13	0.12
609	0.10	0.11	0.11	0.12	0.11
610	0.09	0.10	0.10	0.10	0.10
611	0.09	0.10	0.09	0.10	0.09
612	0.09	0.10	0.09	0.10	0.09
613	0.10	0.10	0.09	0.11	0.10
614	0.10	0.10	0.12	0.14	0.12
615	0.11	0.11	0.11	0.12	0.11
616	0.13	0.13	0.13	0.13	0.12
617	0.13	0.14	0.14	0.14	0.13
618	0.17	0.17	0.16	0.17	0.16
619	0.21	0.20	0.20	0.21	0.21
620	0.25	0.24	0.24	0.24	0.26
621	0.31	0.31	0.28	0.28	0.32
622	0.36	0.37	0.32	0.29	0.35
623	0.42	0.43	0.36	0.32	0.39
624	0.47	0.45	0.32	0.30	0.35
625	0.47	0.46	0.29	0.27	0.31
626	0.41	0.41	0.33	0.30	0.35
627	0.34	0.35	0.31	0.27	0.29
628	0.28	0.29	0.24	0.24	0.26
629	0.27	0.26	0.22	0.20	0.20
630	0.22	0.24	0.21	0.20	0.18
631	0.19	0.20	0.17	0.18	0.16
632	0.16	0.17	0.16	0.18	0.15
633	0.15	0.15	0.16	0.18	0.15
634	0.12	0.12	0.15	0.16	0.13
635	0.12	0.12	0.14	0.16	0.12
636	0.13	0.14	0.16	0.18	0.14
637	0.16	0.17	0.18	0.22	0.17
638	0.19	0.19	0.22	0.24	0.22
639	0.22	0.21	0.23	0.24	0.23
640	0.22	0.21	0.22	0.23	0.22
641	0.17	0.16	0.16	0.17	0.17
642	0.15	0.14	0.14	0.14	0.14
643	0.14	0.13	0.13	0.14	0.13
644	0.14	0.13	0.12	0.12	0.11
645	0.12	0.13	0.12	0.12	0.11
646	0.14	0.15	0.13	0.13	0.12
647	0.13	0.14	0.12	0.13	0.12
648	0.11	0.11	0.10	0.11	0.10
649	0.10	0.10	0.10	0.11	0.10
650	0.10	0.10	0.10	0.11	0.10
651	0.11	0.12	0.11	0.12	0.11

652	0.12	0.13	0.13	0.13	0.12
653	0.12	0.14	0.13	0.14	0.13
654	0.14	0.15	0.14	0.15	0.14
655	0.14	0.15	0.15	0.16	0.14
656	0.15	0.17	0.16	0.16	0.14
657	0.18	0.20	0.20	0.19	0.17
658	0.17	0.20	0.19	0.19	0.17
659	0.15	0.19	0.17	0.17	0.15
660	0.14	0.17	0.15	0.16	0.14
661	0.14	0.16	0.15	0.15	0.14
662	0.13	0.14	0.12	0.13	0.12
663	0.13	0.14	0.13	0.13	0.13
664	0.11	0.13	0.11	0.12	0.12
665	0.11	0.12	0.11	0.12	0.11
666	0.10	0.12	0.10	0.11	0.11
667	0.10	0.12	0.10	0.11	0.11
668	0.11	0.12	0.10	0.11	0.10
669	0.11	0.12	0.10	0.11	0.10
670	0.10	0.11	0.09	0.11	0.10
671	0.10	0.11	0.10	0.11	0.10
672	0.11	0.12	0.11	0.12	0.11
673	0.12	0.14	0.13	0.14	0.12
674	0.14	0.14	0.14	0.15	0.13
675	0.16	0.17	0.17	0.18	0.16
676	0.20	0.20	0.21	0.22	0.22
677	0.23	0.21	0.23	0.25	0.27
678	0.27	0.24	0.32	0.35	0.34
679	0.32	0.28	0.41	0.46	0.42
680	0.37	0.33	0.52	0.59	0.52
681	0.43	0.36	0.60	0.69	0.57
682	0.43	0.35	0.54	0.67	0.55
683	0.42	0.35	0.52	0.66	0.60
684	0.39	0.34	0.48	0.59	0.57
685	0.34	0.31	0.44	0.50	0.56
686	0.32	0.32	0.43	0.44	0.51
687	0.32	0.30	0.39	0.41	0.44
688	0.30	0.29	0.35	0.37	0.38
689	0.23	0.23	0.26	0.27	0.28
690	0.18	0.19	0.20	0.21	0.21
691	0.15	0.17	0.16	0.17	0.16
692	0.12	0.14	0.13	0.14	0.13
693	0.12	0.14	0.13	0.14	0.12
694	0.11	0.12	0.11	0.12	0.11
695	0.12	0.13	0.11	0.13	0.11
696	0.12	0.13	0.11	0.13	0.12
697	0.12	0.13	0.12	0.13	0.12
698	0.13	0.14	0.13	0.14	0.12
699	0.14	0.14	0.12	0.13	0.13
700	0.15	0.17	0.14	0.17	0.15
701	0.16	0.17	0.15	0.17	0.17

702	0.15	0.16	0.15	0.17	0.16
703	0.15	0.15	0.14	0.16	0.15
704	0.17	0.15	0.16	0.16	0.17
705	0.15	0.13	0.13	0.14	0.14
706	0.15	0.14	0.14	0.15	0.15
707	0.13	0.13	0.12	0.13	0.13
708	0.14	0.13	0.12	0.13	0.14
709	0.14	0.13	0.12	0.13	0.13
710	0.14	0.13	0.12	0.13	0.13
711	0.13	0.12	0.12	0.13	0.13
712	0.11	0.11	0.10	0.11	0.11
713	0.11	0.11	0.10	0.11	0.11
714	0.11	0.11	0.10	0.11	0.11
715	0.12	0.11	0.10	0.11	0.11
716	0.12	0.12	0.11	0.12	0.12
717	0.13	0.12	0.12	0.12	0.12
718	0.12	0.11	0.11	0.11	0.12
719	0.13	0.12	0.12	0.12	0.13
720	0.12	0.11	0.11	0.11	0.12
721	0.12	0.11	0.11	0.12	0.12
722	0.11	0.11	0.10	0.11	0.11
723	0.11	0.11	0.10	0.11	0.11
724	0.10	0.10	0.10	0.10	0.10
725	0.09	0.09	0.09	0.09	0.10
726	0.09	0.09	0.09	0.09	0.09
727	0.08	0.08	0.08	0.09	0.09
728	0.08	0.08	0.08	0.09	0.09
729	0.08	0.09	0.08	0.09	0.09
730	0.08	0.09	0.08	0.09	0.09
731	0.09	0.10	0.09	0.10	0.10
732	0.09	0.10	0.10	0.10	0.10
733	0.08	0.09	0.09	0.09	0.09
734	0.08	0.09	0.09	0.09	0.09
735	0.09	0.08	0.09	0.09	0.09
736	0.08	0.08	0.08	0.08	0.08
737	0.08	0.08	0.08	0.07	0.08
738	0.08	0.08	0.08	0.08	0.08
739	0.09	0.09	0.08	0.09	0.09
740	0.08	0.08	0.08	0.08	0.08
741	0.08	0.07	0.07	0.07	0.07
742	0.08	0.09	0.08	0.08	0.08
743	0.09	0.09	0.09	0.08	0.09
744	0.10	0.11	0.11	0.10	0.11
745	0.12	0.12	0.13	0.12	0.11
746	0.11	0.10	0.12	0.10	0.11
747	0.13	0.11	0.13	0.12	0.14
748	0.11	0.11	0.13	0.11	0.13
749	0.09	0.09	0.10	0.09	0.10
750	0.10	0.10	0.11	0.10	0.12
751	0.11	0.10	0.12	0.11	0.13

752	0.09	0.09	0.11	0.10	0.11
753	0.09	0.09	0.10	0.09	0.10
754	0.10	0.10	0.11	0.10	0.11
755	0.09	0.09	0.11	0.10	0.11
756	0.09	0.09	0.10	0.10	0.09
757	0.11	0.11	0.12	0.13	0.10
758	0.09	0.10	0.11	0.10	0.10
759	0.08	0.09	0.09	0.09	0.08
760	0.08	0.09	0.09	0.09	0.08
761	0.08	0.09	0.09	0.09	0.08
762	0.08	0.08	0.09	0.09	0.08
763	0.08	0.08	0.08	0.08	0.08
764	0.08	0.08	0.08	0.09	0.08
765	0.08	0.09	0.09	0.09	0.09
766	0.08	0.09	0.09	0.09	0.09
767	0.08	0.09	0.08	0.09	0.08
768	0.09	0.10	0.09	0.10	0.09
769	0.09	0.11	0.10	0.10	0.10
770	0.08	0.10	0.08	0.09	0.09
771	0.09	0.11	0.09	0.10	0.10
772	0.10	0.12	0.10	0.11	0.11
773	0.09	0.11	0.09	0.10	0.10
774	0.09	0.10	0.08	0.09	0.10
775	0.09	0.10	0.09	0.10	0.10
776	0.10	0.11	0.09	0.10	0.10
777	0.09	0.10	0.08	0.09	0.10
778	0.10	0.10	0.08	0.10	0.10
779	0.10	0.10	0.08	0.10	0.10
780	0.10	0.09	0.08	0.10	0.10
781	0.09	0.08	0.07	0.09	0.09
782	0.10	0.09	0.08	0.10	0.10
783	0.12	0.10	0.09	0.12	0.11
784	0.12	0.10	0.10	0.13	0.11
785	0.12	0.12	0.11	0.13	0.12
786	0.17	0.15	0.14	0.15	0.15
787	0.13	0.13	0.12	0.14	0.13
788	0.13	0.12	0.11	0.12	0.12
789	0.12	0.12	0.11	0.12	0.12
790	0.14	0.15	0.14	0.15	0.14
791	0.18	0.19	0.16	0.18	0.16
792	0.19	0.19	0.17	0.19	0.18
793	0.20	0.19	0.18	0.19	0.18
794	0.18	0.17	0.18	0.17	0.17
795	0.20	0.19	0.20	0.19	0.19
796	0.17	0.16	0.18	0.17	0.16
797	0.14	0.14	0.12	0.13	0.13
798	0.18	0.17	0.15	0.15	0.15
799	0.16	0.16	0.14	0.15	0.15
800	0.13	0.13	0.12	0.12	0.12
801	0.13	0.13	0.13	0.13	0.13

802	0.12	0.12	0.11	0.12	0.12
803	0.14	0.14	0.13	0.14	0.13
804	0.13	0.13	0.12	0.13	0.13
805	0.11	0.11	0.11	0.12	0.11
806	0.12	0.12	0.12	0.13	0.12
807	0.15	0.15	0.15	0.15	0.15
808	0.24	0.24	0.23	0.23	0.22
809	0.34	0.34	0.33	0.34	0.33
810	0.39	0.38	0.39	0.40	0.36
811	0.35	0.35	0.36	0.35	0.34
812	0.37	0.36	0.36	0.35	0.35
813	0.26	0.26	0.27	0.27	0.26
814	0.18	0.18	0.18	0.18	0.18
815	0.14	0.14	0.14	0.14	0.13
816	0.12	0.12	0.12	0.13	0.12
817	0.13	0.13	0.13	0.14	0.13
818	0.12	0.12	0.12	0.12	0.12
819	0.11	0.12	0.11	0.12	0.11
820	0.13	0.13	0.13	0.14	0.13
821	0.13	0.14	0.13	0.14	0.13
822	0.12	0.13	0.12	0.13	0.12
823	0.12	0.13	0.13	0.13	0.13
824	0.15	0.16	0.15	0.16	0.15
825	0.14	0.16	0.15	0.15	0.16
826	0.14	0.15	0.14	0.15	0.16
827	0.16	0.18	0.17	0.18	0.18
828	0.17	0.20	0.18	0.20	0.19
829	0.17	0.21	0.20	0.21	0.19
830	0.19	0.25	0.21	0.23	0.23
831	0.20	0.33	0.22	0.23	0.26
832	0.23	0.32	0.18	0.21	0.20
833	0.18	0.25	0.14	0.17	0.15
834	0.15	0.19	0.13	0.15	0.13
835	0.16	0.15	0.12	0.14	0.12
836	0.16	0.13	0.12	0.13	0.12
837	0.15	0.11	0.11	0.12	0.12
838	0.17	0.13	0.13	0.13	0.14
839	0.16	0.13	0.13	0.14	0.13
840	0.14	0.11	0.11	0.11	0.12
841	0.16	0.13	0.13	0.14	0.14
842	0.20	0.19	0.18	0.18	0.18
843	0.20	0.19	0.19	0.19	0.19
844	0.19	0.18	0.20	0.18	0.18
845	0.18	0.20	0.22	0.19	0.20
846	0.17	0.18	0.21	0.18	0.20
847	0.16	0.16	0.17	0.14	0.15
848	0.13	0.14	0.15	0.13	0.14
849	0.12	0.13	0.13	0.12	0.14
850	0.12	0.12	0.12	0.12	0.12
851	0.11	0.10	0.10	0.10	0.10

852	0.11	0.10	0.10	0.10	0.11
853	0.10	0.09	0.09	0.09	0.10
854	0.10	0.09	0.09	0.09	0.09
855	0.10	0.09	0.09	0.09	0.09
856	0.10	0.09	0.08	0.09	0.08
857	0.09	0.09	0.09	0.09	0.09
858	0.08	0.09	0.08	0.09	0.08
859	0.09	0.09	0.08	0.09	0.09
860	0.09	0.10	0.08	0.10	0.09
861	0.09	0.10	0.09	0.10	0.10
862	0.10	0.12	0.10	0.11	0.10
863	0.09	0.11	0.09	0.10	0.10
864	0.09	0.10	0.09	0.10	0.10
865	0.11	0.11	0.10	0.11	0.10
866	0.12	0.12	0.11	0.12	0.11
867	0.12	0.12	0.11	0.13	0.11
868	0.12	0.12	0.12	0.13	0.12
869	0.11	0.11	0.10	0.11	0.10
870	0.10	0.10	0.09	0.10	0.10
871	0.10	0.11	0.10	0.11	0.10
872	0.11	0.11	0.10	0.11	0.10
873	0.10	0.09	0.09	0.10	0.09
874	0.10	0.10	0.09	0.10	0.09
875	0.10	0.10	0.10	0.11	0.10
876	0.10	0.10	0.10	0.10	0.10
877	0.10	0.09	0.08	0.09	0.09
878	0.10	0.09	0.09	0.10	0.09
879	0.10	0.10	0.10	0.10	0.10
880	0.10	0.10	0.10	0.10	0.10
881	0.10	0.10	0.09	0.10	0.09
882	0.10	0.10	0.09	0.10	0.10
883	0.11	0.10	0.10	0.10	0.10
884	0.10	0.10	0.10	0.10	0.10
885	0.11	0.10	0.10	0.12	0.11
886	0.11	0.10	0.10	0.11	0.10
887	0.11	0.10	0.10	0.10	0.10
888	0.11	0.10	0.10	0.11	0.10
889	0.14	0.12	0.12	0.13	0.12
890	0.17	0.14	0.13	0.12	0.13
891	0.18	0.15	0.16	0.15	0.16
892	0.16	0.14	0.14	0.14	0.14
893	0.12	0.11	0.12	0.12	0.12
894	0.11	0.11	0.10	0.11	0.11
895	0.11	0.11	0.10	0.11	0.11
896	0.11	0.10	0.10	0.10	0.10
897	0.11	0.10	0.10	0.11	0.10
898	0.11	0.10	0.09	0.10	0.10
899	0.11	0.11	0.10	0.11	0.11
900	0.11	0.10	0.10	0.11	0.10
901	0.10	0.09	0.09	0.10	0.10

902	0.10	0.09	0.09	0.10	0.10
903	0.10	0.10	0.09	0.10	0.10
904	0.10	0.09	0.09	0.10	0.10
905	0.09	0.08	0.08	0.09	0.09
906	0.09	0.08	0.09	0.09	0.09
907	0.09	0.09	0.09	0.10	0.10
908	0.09	0.09	0.09	0.10	0.10
909	0.10	0.09	0.10	0.10	0.10
910	0.10	0.10	0.10	0.11	0.10
911	0.11	0.10	0.10	0.11	0.10
912	0.11	0.10	0.10	0.12	0.11
913	0.12	0.11	0.10	0.12	0.11
914	0.13	0.12	0.11	0.12	0.12
915	0.12	0.12	0.11	0.12	0.12
916	0.12	0.12	0.10	0.12	0.11
917	0.13	0.13	0.12	0.13	0.12
918	0.14	0.14	0.12	0.14	0.13
919	0.14	0.14	0.12	0.14	0.13
920	0.14	0.14	0.12	0.13	0.13
921	0.15	0.15	0.14	0.14	0.15
922	0.14	0.14	0.13	0.14	0.14
923	0.13	0.13	0.11	0.12	0.13
924	0.13	0.13	0.12	0.13	0.13
925	0.14	0.14	0.13	0.14	0.14
926	0.13	0.14	0.12	0.13	0.13
927	0.13	0.13	0.12	0.12	0.12
928	0.14	0.14	0.12	0.14	0.14
929	0.14	0.15	0.13	0.15	0.14
930	0.14	0.14	0.12	0.14	0.13
931	0.13	0.14	0.12	0.14	0.13
932	0.15	0.17	0.14	0.16	0.15
933	0.15	0.16	0.14	0.15	0.15
934	0.14	0.14	0.13	0.14	0.14
935	0.14	0.15	0.14	0.15	0.15
936	0.16	0.18	0.17	0.17	0.17
937	0.16	0.17	0.17	0.17	0.17
938	0.15	0.16	0.16	0.16	0.17
939	0.17	0.19	0.19	0.19	0.20
940	0.20	0.21	0.23	0.21	0.22
941	0.23	0.25	0.28	0.23	0.24
942	0.25	0.26	0.30	0.25	0.27
943	0.22	0.25	0.27	0.23	0.26
944	0.20	0.19	0.23	0.20	0.21
945	0.16	0.14	0.15	0.14	0.15
946	0.18	0.15	0.19	0.16	0.16
947	0.15	0.12	0.16	0.15	0.14
948	0.11	0.10	0.12	0.12	0.12
949	0.12	0.11	0.13	0.13	0.13
950	0.13	0.12	0.14	0.14	0.13
951	0.12	0.10	0.12	0.13	0.12

952	0.11	0.11	0.12	0.12	0.12
953	0.11	0.11	0.11	0.13	0.12
954	0.11	0.10	0.10	0.12	0.11
955	0.11	0.09	0.10	0.11	0.10
956	0.12	0.11	0.10	0.11	0.11
957	0.11	0.10	0.10	0.11	0.11
958	0.10	0.10	0.09	0.10	0.09
959	0.09	0.09	0.09	0.10	0.09
960	0.09	0.10	0.09	0.10	0.09
961	0.09	0.10	0.09	0.09	0.09
962	0.08	0.09	0.09	0.09	0.09
963	0.09	0.09	0.09	0.09	0.09
964	0.09	0.10	0.10	0.09	0.09
965	0.09	0.10	0.09	0.09	0.08
966	0.09	0.10	0.09	0.09	0.08
967	0.10	0.11	0.11	0.10	0.09
968	0.09	0.10	0.10	0.09	0.09
969	0.10	0.10	0.10	0.10	0.09
970	0.09	0.09	0.10	0.10	0.09
971	0.10	0.10	0.11	0.11	0.10
972	0.09	0.09	0.10	0.10	0.10
973	0.10	0.10	0.11	0.10	0.10
974	0.10	0.10	0.10	0.10	0.10
975	0.10	0.10	0.11	0.10	0.09
976	0.09	0.10	0.11	0.10	0.09
977	0.09	0.09	0.10	0.09	0.09
978	0.10	0.10	0.11	0.10	0.09
979	0.09	0.09	0.10	0.09	0.09
980	0.08	0.09	0.09	0.09	0.08
981	0.09	0.09	0.09	0.09	0.09
982	0.09	0.10	0.10	0.10	0.09
983	0.09	0.09	0.09	0.10	0.09
984	0.08	0.09	0.09	0.09	0.09
985	0.10	0.09	0.10	0.11	0.10
986	0.11	0.11	0.11	0.13	0.12
987	0.12	0.12	0.12	0.14	0.13
988	0.10	0.11	0.11	0.12	0.11
989	0.09	0.09	0.10	0.10	0.10
990	0.11	0.10	0.11	0.11	0.13
991	0.11	0.10	0.11	0.12	0.12
992	0.09	0.09	0.09	0.10	0.09
993	0.08	0.08	0.09	0.09	0.09
994	0.08	0.08	0.08	0.09	0.08
995	0.09	0.08	0.09	0.09	0.08
996	0.08	0.08	0.08	0.08	0.08
997	0.08	0.07	0.08	0.07	0.07
998	0.08	0.08	0.08	0.07	0.07
999	0.08	0.08	0.08	0.07	0.07
1000	0.07	0.07	0.07	0.07	0.07
1001	0.07	0.07	0.07	0.07	0.07

1002	0.07	0.07	0.08	0.08	0.07
1003	0.07	0.07	0.08	0.08	0.07
1004	0.07	0.07	0.07	0.07	0.07
1005	0.07	0.07	0.07	0.07	0.07
1006	0.07	0.07	0.08	0.08	0.07
1007	0.07	0.07	0.07	0.08	0.07
1008	0.07	0.07	0.07	0.08	0.07
1009	0.07	0.08	0.08	0.08	0.08
1010	0.07	0.08	0.08	0.08	0.08
1011	0.07	0.07	0.07	0.08	0.07
1012	0.07	0.07	0.07	0.08	0.08
1013	0.07	0.08	0.08	0.08	0.08
1014	0.08	0.08	0.08	0.08	0.08
1015	0.08	0.08	0.08	0.09	0.09
1016	0.08	0.09	0.08	0.09	0.09
1017	0.08	0.09	0.08	0.09	0.09
1018	0.08	0.08	0.08	0.09	0.09
1019	0.08	0.08	0.08	0.09	0.09
1020	0.09	0.09	0.08	0.09	0.09
1021	0.08	0.08	0.08	0.09	0.09
1022	0.08	0.07	0.07	0.09	0.08
1023	0.08	0.08	0.08	0.09	0.09
1024	0.08	0.08	0.08	0.09	0.09
1025	0.07	0.07	0.07	0.08	0.08
1026	0.07	0.07	0.07	0.08	0.08
1027	0.07	0.07	0.07	0.08	0.08
1028	0.07	0.07	0.07	0.08	0.08
1029	0.07	0.07	0.07	0.08	0.08
1030	0.07	0.07	0.07	0.08	0.08
1031	0.07	0.06	0.07	0.08	0.08
1032	0.07	0.07	0.07	0.08	0.08
1033	0.07	0.07	0.07	0.08	0.08
1034	0.08	0.08	0.08	0.08	0.08
1035	0.08	0.08	0.09	0.09	0.09
1036	0.08	0.07	0.08	0.09	0.08
1037	0.08	0.08	0.09	0.10	0.09
1038	0.10	0.09	0.12	0.11	0.13
1039	0.07	0.07	0.08	0.10	0.10
1040	0.08	0.08	0.09	0.10	0.10
1041	0.09	0.09	0.09	0.10	0.11
1042	0.08	0.08	0.08	0.10	0.09
1043	0.08	0.08	0.08	0.09	0.09
1044	0.10	0.10	0.10	0.10	0.10
1045	0.11	0.10	0.10	0.11	0.12
1046	0.11	0.10	0.10	0.10	0.12
1047	0.09	0.08	0.09	0.09	0.10
1048	0.08	0.08	0.08	0.08	0.09
1049	0.08	0.08	0.08	0.08	0.09
1050	0.08	0.08	0.08	0.08	0.09
1051	0.08	0.08	0.08	0.08	0.08

1052	0.09	0.09	0.09	0.09	0.09
1053	0.09	0.08	0.08	0.09	0.09
1054	0.09	0.09	0.08	0.09	0.09
1055	0.10	0.10	0.10	0.10	0.09
1056	0.10	0.10	0.10	0.11	0.10
1057	0.10	0.11	0.10	0.11	0.11
1058	0.09	0.10	0.09	0.10	0.10
1059	0.09	0.09	0.08	0.09	0.09
1060	0.08	0.08	0.08	0.09	0.09
1061	0.09	0.09	0.08	0.09	0.09
1062	0.08	0.08	0.08	0.09	0.09
1063	0.09	0.09	0.08	0.09	0.09
1064	0.09	0.09	0.08	0.09	0.09
1065	0.09	0.09	0.09	0.09	0.10
1066	0.10	0.09	0.09	0.09	0.10
1067	0.10	0.10	0.09	0.10	0.10
1068	0.12	0.10	0.10	0.10	0.11
1069	0.12	0.11	0.11	0.11	0.11
1070	0.14	0.13	0.13	0.12	0.13
1071	0.14	0.12	0.12	0.13	0.13
1072	0.13	0.12	0.12	0.12	0.12
1073	0.13	0.12	0.12	0.13	0.13
1074	0.13	0.12	0.12	0.13	0.12
1075	0.13	0.12	0.12	0.13	0.12
1076	0.13	0.12	0.12	0.13	0.13
1077	0.13	0.12	0.11	0.12	0.12
1078	0.13	0.12	0.12	0.13	0.12
1079	0.13	0.12	0.11	0.13	0.13
1080	0.13	0.12	0.12	0.14	0.13
1081	0.15	0.13	0.13	0.15	0.14
1082	0.16	0.15	0.16	0.17	0.15
1083	0.17	0.17	0.18	0.18	0.17
1084	0.20	0.19	0.20	0.22	0.20
1085	0.20	0.19	0.20	0.21	0.19
1086	0.17	0.16	0.17	0.18	0.16
1087	0.15	0.14	0.14	0.16	0.14
1088	0.14	0.12	0.13	0.14	0.13
1089	0.12	0.11	0.12	0.14	0.12
1090	0.12	0.11	0.12	0.14	0.12
1091	0.13	0.12	0.13	0.14	0.13
1092	0.12	0.12	0.12	0.13	0.13
1093	0.12	0.11	0.10	0.12	0.11
1094	0.11	0.10	0.10	0.12	0.11
1095	0.12	0.11	0.11	0.12	0.11
1096	0.13	0.11	0.11	0.13	0.12
1097	0.15	0.13	0.13	0.14	0.14
1098	0.25	0.21	0.22	0.23	0.23
1099	0.28	0.23	0.24	0.27	0.27
1100	0.27	0.24	0.23	0.25	0.24
1101	0.20	0.17	0.17	0.19	0.18

1102	0.16	0.13	0.13	0.15	0.14
1103	0.14	0.12	0.12	0.14	0.13
1104	0.13	0.11	0.11	0.13	0.12
1105	0.12	0.11	0.10	0.12	0.12
1106	0.12	0.11	0.10	0.12	0.11
1107	0.11	0.12	0.10	0.12	0.11
1108	0.12	0.11	0.10	0.12	0.11
1109	0.12	0.11	0.10	0.12	0.11
1110	0.12	0.11	0.10	0.12	0.11
1111	0.13	0.12	0.12	0.13	0.13
1112	0.14	0.13	0.12	0.14	0.14
1113	0.14	0.12	0.13	0.15	0.14
1114	0.15	0.14	0.14	0.15	0.14
1115	0.14	0.13	0.13	0.14	0.13
1116	0.15	0.14	0.14	0.15	0.14
1117	0.15	0.14	0.15	0.16	0.15
1118	0.16	0.15	0.16	0.17	0.15
1119	0.15	0.13	0.14	0.15	0.14
1120	0.14	0.12	0.13	0.14	0.13
1121	0.14	0.13	0.14	0.16	0.14
1122	0.15	0.14	0.15	0.16	0.15
1123	0.16	0.15	0.16	0.18	0.16
1124	0.20	0.21	0.19	0.22	0.20
1125	0.20	0.17	0.18	0.19	0.18
1126	0.18	0.17	0.17	0.18	0.17
1127	0.21	0.20	0.20	0.20	0.20
1128	0.22	0.22	0.20	0.20	0.20
1129	0.19	0.18	0.17	0.18	0.18
1130	0.21	0.19	0.18	0.18	0.20
1131	0.19	0.18	0.17	0.18	0.19
1132	0.17	0.15	0.15	0.16	0.16
1133	0.17	0.16	0.15	0.16	0.16
1134	0.19	0.17	0.17	0.17	0.18
1135	0.18	0.17	0.17	0.17	0.18
1136	0.19	0.19	0.19	0.18	0.20
1137	0.17	0.18	0.19	0.17	0.20
1138	0.18	0.21	0.23	0.19	0.23
1139	0.21	0.25	0.30	0.23	0.30
